# Human amniotic membrane modulates collagen production and deposition in vitro

**DOI:** 10.1038/s41598-024-64364-2

**Published:** 2024-07-10

**Authors:** Sarah E. Moreno, Isioma Enwerem-Lackland, Kristiana Dreaden, Michelle Massee, Thomas J. Koob, John R. Harper

**Affiliations:** 1 MIMEDX Group, Inc., 1775 West Oak Commons Court NE, Marietta, GA 30062 USA; 2 Alkermes, 900 Winter St., Waltham, MA 02451 USA

**Keywords:** Tissues, Mechanisms of disease

## Abstract

Pathological fibrosis is a significant complication of surgical procedures resulting from the accumulation of excess collagen at the site of repair which can compromise the tissue architecture and severely impede the function of the affected tissue. Few prophylactic treatments exist to counteract this process; however, the use of amniotic membrane allografts has demonstrated promising clinical outcomes. This study aimed to identify the underlying mechanism of action by utilizing relevant models that accurately represent the pathophysiology of the disease state. This study employed a pro-fibrotic in vitro system using TGFβ1 stimulation and macromolecular crowding techniques to evaluate the mechanism by which amniotic membrane allografts regulate collagen biosynthesis and deposition. Following treatment with dehydrated human amnion chorion membrane (DHACM), subsequent RNA sequencing and functional enrichment with Reactome pathway analysis indicated that amniotic membranes are indeed capable of regulating genes associated with the composition and function of the extracellular matrix. Furthermore, macromolecular crowding was used in vitro to expand the evaluation to include both the effects of DHACM and a lyophilized human amnion/chorion membrane (LHACM). DHACM and LHACM regulate the TGFβ pathway and myofibroblast differentiation. Additionally, both DHACM and LHACM modulate the production, secretion, and deposition of collagen type I, a primary target for pathological fibrosis. These observations support the hypothesis that amniotic membranes may interrupt pathological fibrosis by regulating collagen biosynthesis and associated pathways.

## Introduction

Upon acute dermal injury, the body responds by initiating regenerative and reparative pathways to restore tissue architecture and function. Collagen remodeling continues for months after wound closure and the tensile strength of the repaired tissue increases to about 80–85% of normal tissue if all processes proceed without any perturbations^1^. However, chronic and persistent pathologic circumstances may lead to an abnormal repair process with excessive accumulation of extracellular cellular matrix (ECM)^2^. The resultant scarring can range in severity from poor cosmetic outcomes to significant restrictions in function, identifying a significant unmet need. Therefore, targeting the molecular mechanisms associated with regulating ECM synthesis, deposition, and degradation could highlight potential therapeutic targets for the development of novel and effective therapies^3,4^.

An ideal clinical treatment would support the healing cascade to enable accelerated recovery and improved quality of healing, much like the fetal response to injury. The fetal dermis can regenerate a non-disrupted collagen matrix that is identical to that of the original tissue, commonly referred to as scarless healing^5^. While attempts have been made to harness this mechanism, modalities aimed at recreating fetal wound healing in adult tissues have not proven successful^6,7^. The difficulty largely stems from the complex mechanism that is not well-understood; however, similar properties have been reported in amniotic membranes. Allografts made from amniotic membrane have been used successfully in the treatment of complex wounds including burns, diabetic foot ulcers, venous ulcers, ocular injuries, and uterine adherences^8–11^. The primary endpoint in these studies is rate of closure; however, observations of reduced scarring and decreased incidence of recurrence may be attributable to amniotic membrane’s ability to regulate ECM architecture, making it a potential candidate for the treatment of fibrotic disease^12–14^.

Evaluating the underlying mechanism of action for the observed clinical outcomes can be difficult with the inherent shortcomings of in vitro cell culture systems. It is necessary to recreate the in vivo cellular environment by mimicking the complex network of the ECM containing fibroblasts that drive ECM deposition and remodeling^15,16^. Standard in vitro models are plagued by sluggish production and deposition of ECM components, severely limiting the ability to recreate hyperactive fibrosis. Limitations of the extracellular post-translational modifications of collagen, namely the slow enzymatic conversion of immature pro-collagen to mature collagen, and the formation of covalent crosslinks, results in minimal amounts of functional mature collagen in vitro^17–19^. Therefore, the data generated from these experiments cannot necessarily be extrapolated to the clinical setting, as the model does not fully represent clinical etiology. Macromolecular crowding (MMC) is a technique which facilitates more representative cell–cell interactions and enzyme–substrate reactions by closely approximating the components of the culture system, thus promoting the deposition of a more complex ECM^20^. This system provides a more physiologically relevant model for assessing anti-fibrotic therapeutics^21–23^.

In this study, two unique PURION® processed amniotic tissue allografts were evaluated for their ability to regulate collagen biosynthesis and deposition. Lyophilized human amnion, intermediate layer, and chorion membrane (LHACM) is a freeze-dried tri-layer allograft; whereas, dehydrated human amnion, chorion membrane (DHACM) is an air-dried bi-layer allograft. Previous experiments demonstrated that this patented and proprietary process retains well known regulatory proteins inherent to amniotic tissues, and preserves the bioactivity to stimulate cellular activities, such as proliferation, migration, and biosynthesis in multiple cell types^24–30^. Moreover, DHACM has been shown to contain anti-fibrotic potential through the regulation of the TGFβ-SMAD pathway leading to the modulation of myofibroblast contraction suggesting a potential impact on the production and deposition of ECM components^31^. However, the limitations of the culture system potentially necessitate the use of a more physiologically relevant model to validate this finding for DHACM and evaluate LHACM. This study aimed to provide an assessment of the effects of LHACM and DHACM treatment in a physiologically relevant in vitro fibrotic model on collagen production, deposition, and maturation in a simulated, pro-fibrotic environment.

## Material and methods

### Human amnion/chorion membrane

Human birth tissue was acquired and processed at previously described^31^. Briefly, human placentas, from Caesarean sections, were donated under informed written consent in compliance with the Food and Drug Administration’s Good Tissue Practice and American Association of Tissue Banks standards. MIMEDX Group is accredited by American Association of Tissue Banks for donor eligibility assessment, informed consent, acquisition, processing, release, storage, and distribution of birth tissue for transplantation and therefore this study did not require institutional approval. All donors were confirmed to be free of infectious diseases, including human immunodeficiency virus (HIV), human T-lymphotropic virus (HTLV), hepatitis B and C, and syphilis. Under controlled conditions, amnion and chorion are separated from the placenta and processed in accordance with the proprietary PURION® Process, in which the amnion and chorion layers are gently cleansed, laminated and dehydrated^32–34^. LHACM (MIMEDX, Marietta, GA) is a lyophilized human allograft composed of laminated amnion, intermediate and chorion layer and DHACM (MIMEDX, Marietta, GA) is a dehydrated human allograft comprised of laminated amnion and chorion. Both are derived from the human amniotic sac.

### Extract preparation

Soluble extracts for cell culture experiments were prepared as previously described^31^. Briefly, 1–3 donors of DHACM and LHACM were minced, combined, and extracted overnight at 40 mg of tissue per milliliter of basal DMEM (Dulbecco’s modified eagle’s medium (DMEM) containing 1% penicillin streptomycin and 1% sodium pyruvate). The resulting extract was clarified and collected in a sterile container, followed by preparation at testing concentrations by dilution in basal DMEM. Three independent extracts of DHACM and LHACM samples were used in each subsequent experiment.

### Cell culture and treatment

Human dermal fibroblasts (HDF) (Thermo Fisher Scientific, Waltham, MA) between passages 5–9 were maintained as described previously in DMEM (Thermo Fisher Scientific, Waltham, MA) supplemented with 10% fetal bovine serum (FBS) (Thermo Fisher Scientific, Waltham, MA), 1% penicillin streptomycin (Fisher Scientific, Waltham, MA) and 1% sodium pyruvate (Thermo Fisher Scientific, Waltham, MA) at 37 °C, 5% CO_2_ until 80% confluent^31^.

To initiate experiments under MMC conditions, cells were seeded in 96 well plates, 6 well plates, 100 mm dishes, and 4 well slides plated at 10,000 cells/cm^2^ for each experiment. After 4 h, adherent cells were washed with basal DMEM, and incubated overnight in DMEM containing 0.4% FBS, 1% penicillin streptomycin, and 1% sodium pyruvate followed by treatment of HDFs for six days. Culture media and treatments were replaced on day 3. All treatments were carried out under MMC conditions in DMEM containing 0.4% FBS, 1% penicillin streptomycin, 1% sodium pyruvate, 1 mM of L-ascorbic acid 2-phospate (Sigma-Aldrich, St. Louis, MO), and a mixture of 37.5 mg/mL Ficoll 70 kDa (Sigma-Aldrich, St. Louis, MO) and 25 mg/ml Ficoll 400 kDa (Sigma-Aldrich, St. Louis, MO). The following control groups were included for each experiment: basal media and 10 ng/mL TGFβ1 (R&D Systems, Minneapolis, MN). Treatments were carried out with LHACM (10 mg/mL, and 1 mg/mL) or DHACM (20 mg/mL, 10 mg/mL, and 1 mg/mL) in the presence of 10 ng/mL TGFβ1. Extracts were prepared as described above.

### RNA sequencing analysis

RNA isolation, library preparation and RNA sequencing was performed by Novogene (Sacramento, CA). Messenger RNA was purified from total RNA followed by library preparation. Libraries were sequenced on NovaSeq 6000, an Illumina platform. Differential expression analysis was conducted using DESeq2 R package (version 1.20.0). The resulting *p* values were adjusted using the Benjamini and Hochberg’s approach for controlling the false discovery rate. Genes with an adjusted *p* value < 0.05 found by DESeq2 and an absolute value of log2 fold change greater than 0.75 were differentially expressed. Reactome Pathway enrichment analysis of differentially expressed genes (DEGs) was done using the Reactome database’s default settings (http://www.reactome.org). This analysis allowed identification of over-represented biological pathways and processes associated with the gene expression changes observed in the dataset. The significance of pathway enrichment was determined based on multiple statistical tests, and pathways with adjusted *p* values below *p* < 0.05 were considered significantly enriched. Data visualization was done utilizing Novogene’s NovoMagic analysis portal.

### Quantitative polymerase chain reaction

RNA and complimentary DNA was prepared utilizing the Cells-2-Ct Kit (Thermo Fisher Scientific, Waltham, MA), per the manufacturer’s protocol. Quantitative polymerase chain reaction amplification for each gene target was performed on a QuantStudio™ 7 Flex Real-Time PCR System (Thermo Fisher Scientific, Waltham, MA) using predesigned TaqMan Gene Expression Assays for *ACTA2* (Hs00426835_g1), *BMP1* (Hs00241807_m1), *LOX* (Hs00942483_m1), *TGM2* (Hs01096681_m1), *COL1A1* (Hs00164004_m1), *CTGF* (Hs00164004_m1), and eukaryotic *18s* (4319413E) purchased from Thermo Fisher Scientific (Waltham, MA). Each replicate sample was analyzed in duplicate. Relative mRNA concentrations of the genes of interest were normalized to the relative mRNA of the housekeeping gene 18S. Differences were calculated with the comparative Ct method for each target gene with the results expressed as a fold increase over the control. For graphical representation, the technical replicate values for each sample were combined.

### Western blotting

Total cellular protein was solubilized in radioimmunoprecipitation assay buffer containing proteinase inhibitors. After removal of cellular debris, cell culture supernatants were concentrated using protein concentrators with a molecular weight cutoff of 3 kDa. Samples of LHACM and DHACM extract were used as control for analysis of cell culture supernatants. The protein concentrations were determined using bicinchoninic acid protein assay reagent. Equal amounts of the proteins were separated on a 4–12% gradient sodium dodecyl sulfate poly-acrylamide gel and transferred to nitrocellulose membranes as previously described^31^. After blocking with 5% non-fat dry milk in 1× Tris Buffered Saline 0.05% Tween 20 for 1 h, membranes were probed with primary antibodies overnight at 4 °C: αSMA (19,245, Cell Signaling Technology, Danvers, MA), phospho-SMAD2 Ser465/467 (3108, Cell Signaling Technology, Danvers, MA), SMAD2 (3103 Cell Signaling Technology, Danvers, MA), phospho-SMAD3 Ser423/425 (ab40854, Abcam, Waltham, MA), SMAD3 (ab40854, Abcam, Waltham, MA), BMP1 (ab38953, Abcam, Waltham, MA), LOX (ab174316, Abcam, Waltham, MA), TGase2 (ab2386, Abcam, Waltham, MA), and GAPDH (ab8245, Abcam, Waltham, MA). Following overnight incubation, anti-mouse or anti-rabbit IgG HRP-conjugated secondary antibodies (Abcam, Cambridge, MA) were used to detect binding of antibodies. Immunoreactive proteins were visualized on the Amersham Imager 600 (GE Healthcare Systems Piscataway, NJ) after exposure to chemi-luminescence reagents (Cell Signaling Technology, Danvers, MA).

### Immunofluorescence

Cells, cultured on 4-well slides, were fixed with 4% paraformaldehyde (Electron Microscopy Science, Hatfield, PA) for 30 min at room temperature. For intracellular analysis, cellular membranes were permeabilized with 0.1% Trition-X-100 for 2 min. Cells were blocked in serum free protein block (Agilent DAKO, Santa Clara, CA) for 1 h at room temperature. Incubation with primary antibody against collagen type I (ab138492, Abcam, Waltham, MA) diluted in Antibody Diluent (Agilent DAKO, Santa Clara, CA) was carried out overnight at 4 °C. For visualization, cells were incubated with Goat anti-Mouse IgG (H + L) Alexa Fluor 488 (A11034, Thermo Fisher Scientific, Waltham, MA) and DAPI 4′, 6′-diamidino-2-phenylindole (H-1500 Vector Laboratories, Burlingame, CA) to identify nuclei. Images were acquired on a Leica microscope fitted with 20× objective using Leica Application Suite Advance Fluorescence software and the Thunder Imager from Leica (Leica Microsystems, Wetzlar, Germany). Semi-quantitative analysis was performed on ten independent fields per treatment condition using the ImageJ software (NIH)^35^. Values for each sample were combined for graphical representation.

### Matrix deposition analysis

At day 6, the matrix deposited at the bottom of the wells together with the cells was washed twice with PBS and stored at − 80 °C. Prior to digestion, the matrix was thawed and dissolved with 0.5 M acetic acid overnight followed by digestion with 0.5 mg/mL Pepsin (Sigma-Aldrich St. Louis, MO), in 0.01 M HCl. Samples were neutralized with 1× LDS sample buffer (Thermo Fisher, Waltham, MA) and were analyzed by SDS-PAGE under reducing conditions. Protein bands were stained with the Silver Stain Kit (Thermo Fisher, Waltham, MA) according to the manufacturer’s instructions. Gel images were obtained with Amersham Imager 600 (GE Healthcare Systems Piscataway, NJ).

### Statistical analysis

All values are reported as mean +/− standard deviation and statistical analyses were performed using GraphPad Prism software (version 10.0.2). *p* values ≤ 0.05 were considered statistically significant. Graphical illustrations and one-way ANOVA with pairwise comparisons were made using a Tukey test.

## Results

### Transcriptional profiling

**Figure 1:**
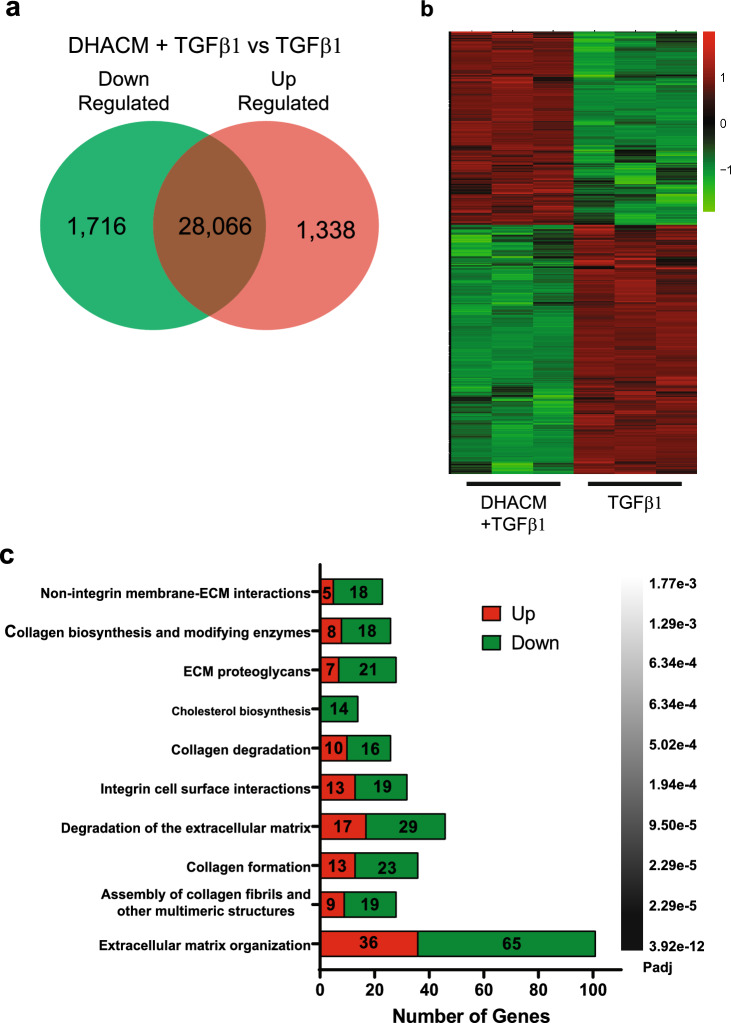
Differential gene expression patterns between DHACM + TGFβ1 and TGFβ1. (**a**) Venn diagram highlighting the DEGs. (**b**) Heatmap of DEGs showing relative expression levels from highest (red) to lowest (green). (**c**) Reactome Pathway analysis of dysregulated genes. Functional annotation of top 10 pathways and genes upregulated (red) or downregulated (green) in each cluster.

DHACM has been previously shown to regulate the TGFβ signaling pathway, leading to a reduced contractile phenotype^31^. This was further investigated in this MMC model using RNA sequencing to assess the gene expression patterns in HDFs and TGFβ1 stimulus with and without DHACM treatment. Treatment of cells with TGFβ1 resulted in 2968 DEGs of which 1565 were down regulated and 1403 were upregulated (Supplementary Fig. S1a). DHACM + TGFβ1 treatment resulted in 3054 DEGs of which 1716 were down regulated and 1338 were upregulated (Fig. 1a). A full list of DEGs is available in Supplementary Tables S1 and S2. Hierarchical clustering demonstrated a clear separation between the two groups (Fig. 1b).

Further refinement and greater biological characterization of DEGs were pursued through functional enrichment analysis utilizing Reactome Pathway analysis. Comparative analysis between the TGFβ1 and basal samples identified the top 10 enriched pathways contained well known pathways involved in the development of fibrosis, including extracellular matrix organization and collagen formation (Supplementary Fig. S1c). The majority of DEGs associated with each Reactome were up regulated by TGFβ1. Interestingly, the top 10 Reactome pathways affected with DHACM treatment include biological pathways associated with regulation of the ECM including, extracellular matrix organization, collagen formation and collagen degradation with a majority of DEGs being down regulated with DHACM treatment (Fig. 1c).

### DHACM and LHACM attenuates the TGFβ1-mediated fibrotic phenotype

**Figure 2:**
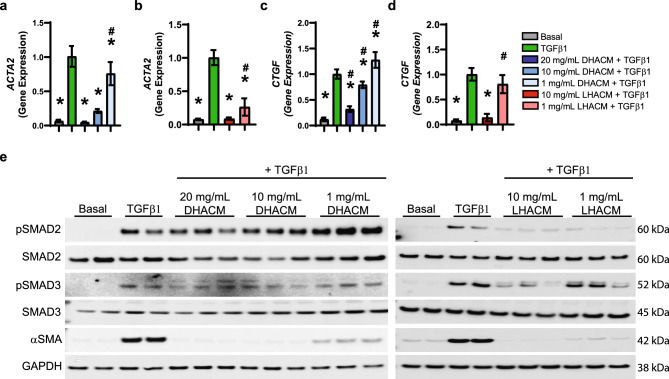
Effect of DHACM and LHACM on TGFβ1 induced fibrotic response. Fold change in gene expression of myofibroblast marker *ACTA2* (**a** and **b**) and fibrotic marker *CTGF* (**c** and **d**). (**e**) Western blot analysis of the phosphorylation of SMAD2 and SMAD3 and αSMA. Full blots available in Supplementary Figure S2. *p < 0.05 versus TGFβ1 control and #*p* < 0.05 versus basal control using one-way ANOVA; n = 3.

Under MMC conditions, with recombinant TGFβ1 stimulation, HDFs increased the expression of phosphorylated SMAD2 and SMAD3 (Fig. 2e), two key transcription factors in the TGFβ signaling cascade. Treatment at 20 and 10 mg/mL DHACM + TGFβ1 reduced the phosphorylation of SMAD2 and SMAD3. The phosphorylation of SMAD2 was decreased by LHACM at 10 and 1 mg/mL + TGFβ1, however, SMAD3 phosphorylation was only impacted at 10 mg/mL LHACM + TGFβ1. Total protein expression was unchanged (Fig. 2e).

As part of the fibrotic process, TGFβ1 governs increased expression of pro-fibrotic genes and myofibroblast differentiation^36–39^. Recombinant TGFβ1 stimulation resulted in an increase in mRNA expression of *CTGF* which is known to contribute to the accumulation of collagen and reduced ECM degradation^40,41^ (Fig. 2c and d). As shown in Fig. 2c, treatment with 20 and 10 mg/mL DHACM + TGFβ1 significantly suppressed the TGFβ1-dependent induction of *CTGF* mRNA. Of note, *CTGF* was significantly suppressed to levels seen in the basal control by treatment with 10 mg/mL LHACM + TGFβ1 (Fig. 2d). A key indicator of fibroblast differentiation into myofibroblasts is the marked increase of αSMA expression^38^. Fibroblasts, under MMC conditions, stimulated with 10 ng/mL of TGFβ1 showed increased mRNA expression of *ACTA2*, the gene coding for αSMA, compared with that of basal control (unstimulated) (Fig. 2a and b). Treatment with DHACM significantly suppressed the TGFβ1-dependent induction of the *ACTA2* mRNA in a dose-dependent manner. Moreover, the TGFβ1-dependent increase of *ACTA2* mRNA was decreased to the basal level at both the 20 and 10 mg/mL doses (Fig. 2a). Similarly, treatment with 10 and 1 mg/mL LHACM + TGFβ1 decreased *ACTA2* expression with 10 mg/mL resulting in mRNA levels similar to the basal level (Fig. 2b). Consequently, western blotting demonstrated DHACM and LHACM significantly inhibited the TGFβ1 induced protein expression of αSMA at all concentrations tested (Fig. 2e).

### DHACM and LHACM regulate collagen biosynthesis and deposition

**Figure 3:**
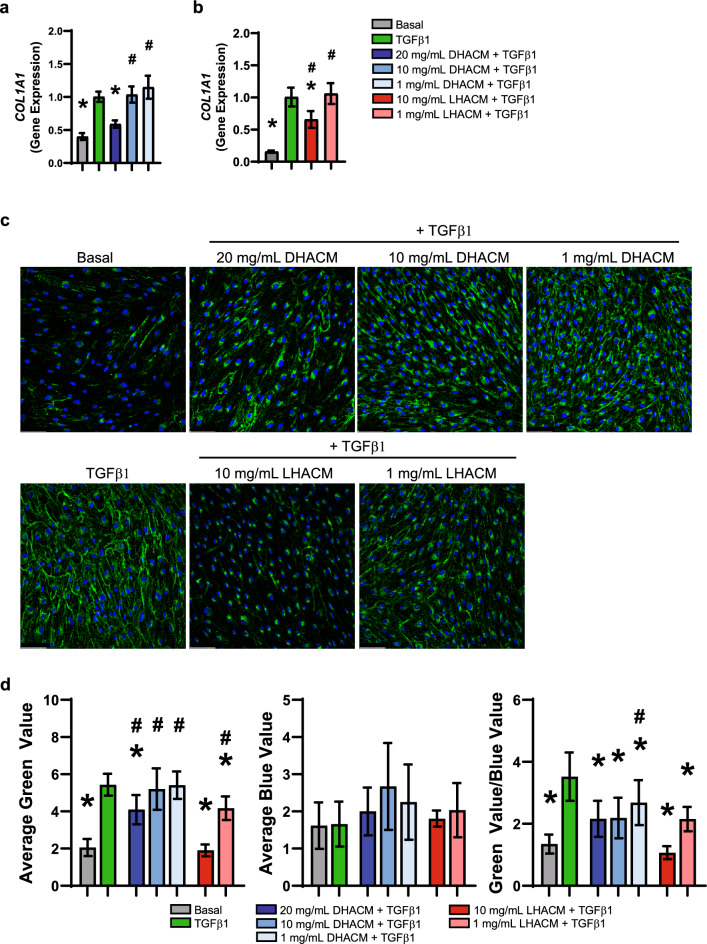
Regulation of collagen type I biosynthesis. Fold change in gene expression of *COL1A1* (**a** and **b**) in HDFs after 6 days in the presence of TGFβ1 cultured with macromolecular crowding and treated with DHACM and LHACM. *p < 0.05 versus TGFβ1 control and #*p* < 0.05 versus basal control using one-way ANOVA; n = 3. (**c**) Immunofluorescence staining of intracellular and extracellular collagen type I in HDFs after 6 days in the presence of TGFβ1 cultured with MMC and treated with DHACM and LHACM. Scale bar = 100 µm. (**d**) Semi-quantitative analysis of IF staining utilizing Image J software. All values represent mean ± SD. *p < 0.05 versus TGFβ1 control and #*p* < 0.05 versus basal control using one-way ANOVA.

A comparison of the control groups demonstrated that HDFs under MMC conditions and stimulated with TGFβ1 resulted in increased gene expression of *COL1A1*, compared with that of basal control. *COL1A1* expression was significantly reduced by treatment with 20 mg/mL of DHACM + TGFβ1 and 10 mg/mL LHACM + TGFβ1 (Fig. 3a and b). Further evaluation of the regulation of collagen synthesis and subsequent secretion was assessed in cells that were permeabilized with 0.1% Triton X-100 for visualization of intracellular and extracellular collagen type I. Immunofluorescence staining confirmed that TGFβ1 stimulation increases the extracellular collagen as suggested by the diffuse, mesh-like pattern. In response to DHACM and LHACM treatment, collagen type I remains predominately localized intracellularly (Fig. 3c). Semi-quantitative analysis demonstrates that there is a marked decrease of collagen type I in response to DHACM and LHACM treatment (Fig. 3d).

**Figure 4:**
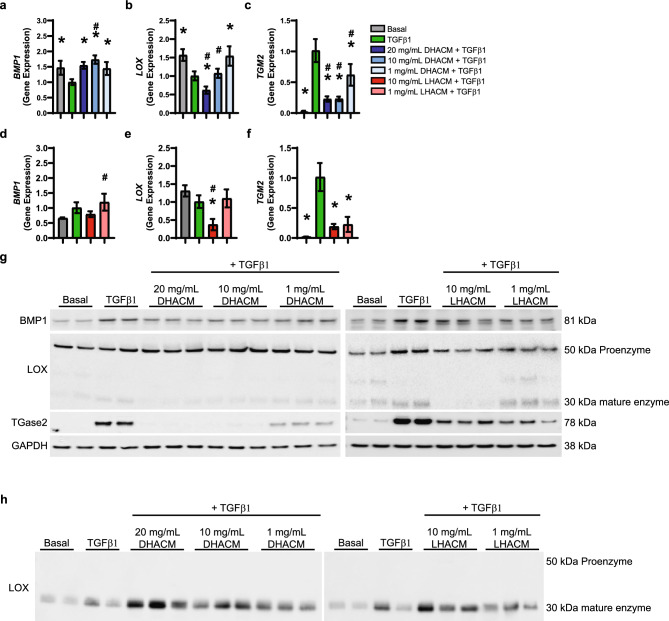
Effect of DHACM and LHACM on collagen modifying enzymes. Fold change in gene expression of *BMP1* (**a** and **d**), *LOX* (**b** and **e**) and *TGM2* (**c** and **f**) in HDFs after 6 days in the presence of TGFβ1 cultured with macromolecular crowding and treated with DHACM and LHACM. *p < 0.05 versus TGFβ1 control and #*p* < 0.05 versus basal control using one-way ANOVA; n = 3. Western blot analysis of BMP1, LOX, TGase2 in cell lysates (**g**) and LOX in cell culture supernatant (**h**). Full blots available in Supplementary Figures S2 and S3.

Collagen matrices feature hierarchical structures that are self-assembled through sequential steps. BMP1 cleaves the C-propeptide of procollagen type I during the assembly of extracellular matrix collagen fibrils^42^. Protein analysis demonstrated that TGFβ1 increased BMP1 protein compared to the basal control. Both DHACM and LHACM reduced the TGFβ1 induced increase in BMP1 (Fig. 4g). These results were confounded by gene expression analysis that shows DHACM + TGFβ1 at concentrations tested and the LHACM + TGFβ1 at 1 mg/mL increased expression of *BMP1* (Fig. 4a and d).

Collagen in the extracellular space is stabilized through intra and intermolecular crosslinks catalyzed by the lysyl oxidase (LOX) and Transglutaminase (TGase) family^43^. Contrary to expectations, *LOX* gene expression was decreased upon stimulation with TGFβ1 in MMC conditions. However, DHACM treatment at 20 mg/mL further down regulated *LOX* expression in the presence of TGFβ1. No differences were observed at 10 mg/mL and 1 mg/mL DHACM + TGFβ1. Similarly, 10 mg/mL LHACM + TGFβ1 down regulated *LOX* expression. No difference was seen with 1 mg/mL LHACM (Fig. 4b and e). LOX is synthesized and processed intracellularly and the resulting proenzyme is secreted into the extracellular space where it undergoes further proteolytical processing to the mature enzyme^44^. The proenzyme was increased intracellularly upon TGFβ1 stimulation. DHACM treatment in the presence of TGFβ1 resulted in only minor changes at the protein level, whereas LHACM significantly reduced the LOX proenzyme. Additionally, TGFβ1 resulted in an increased intracellular accumulation of the mature form of the LOX enzyme. This effect was significantly reduced by both DHACM and LHACM treatment (Fig. 4g). DHACM treatment returned the protein level to that observed in the basal control (Fig. 4g). Expression of *TGM2* was substantially elevated upon TGFβ1 stimulation. LHACM and DHACM treatment in the presence of TGFβ1 reduced the expression of *TGM2* at all concentrations tested (Fig. 4c and f). Analysis of protein levels confirm that LHACM and DHACM decreased the TGFβ1 induced protein level of TGase2. DHACM treatment returned the protein level to that observed in the basal control (Fig. 4g).

The modifications elicited by BMP1, LOX, and TGase2 are catalyzed in the extracellular environment necessitating the evaluation of collagen modifying enzymes in the supernatnt^43^. The extracellular fraction was evaluated using western blotting of cell culture supernatants. The mature form of LOX was increased in the supernatants by TGFβ1 compared to basal. However, treatment with both DHACM (20 mg/mL and 10 mg/mL) and LHACM (10 mg/mL) also significantly increased the amount of mature LOX in the cell culture supernatants (Fig. 4h). Of note, neither the proenzyme nor mature enzyme forms of LOX were detected in the extracts controls. BMP1 was not detected by this method in the cell culture supernatant and assessment of TGase2 was confounded by the presence of this enzyme in the LHACM and DHACM extracts themselves (Supplementary Figure S3).

**Figure 5:**
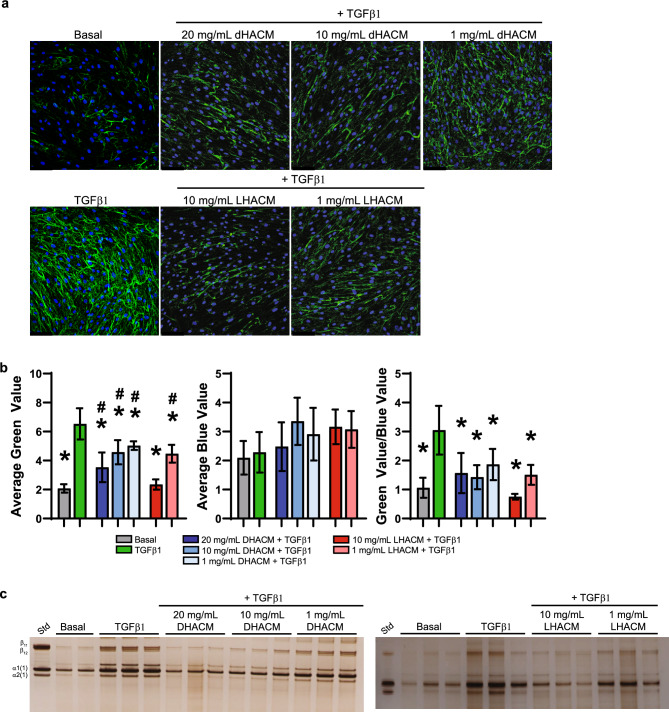
Regulation of collagen deposition in an in vitro MMC model. (**a**) Extracellular immunofluorescence staining of collagen type I in HDFs after 6 days in the presence of TGFβ1 cultured with MMC and treated with DHACM and LHACM. Scale bar = 100 µm. (**b**) Semi-quantitative analysis of IF staining utilizing Image J software. All values represent mean ± SD. *p < 0.05 versus TGFβ1 control and #*p* < 0.05 versus basal control using one-way ANOVA. (**c**) SDS-PAGE analysis of deposited ECM by HDFs after 6 days in the presence of TGFβ1 cultured with macromolecular crowding and treated with DHACM and LHACM.

Collagen type I deposition which was evaluated in cultures of HDFs under MMC conditions. TGFβ1 enhanced extracellular collagen type I deposition as compared to the basal control as assessed by immunofluorescence. As shown in Fig. 5 both DHACM and LHACM prevented the TGFβ1-induced extracellular accumulation of collagen type I in the deposited matrix (Fig. 5a and b). Type 1 collagen deposition was further assessed by pepsin digestion followed by SDS-PAGE. Analysis of solubilized matrices showed that TGFβ1 treatment induced greater collagen type I deposition, however DHACM and LHACM treatment dose dependently reduced the accumulation of collagen type I in the deposited ECM in the presence of TGFβ1 (Fig. 5c).

## Discussion

Despite the remarkable heterogeneity in the etiologic mechanisms responsible for the development of fibrotic diseases and in their clinical manifestations, numerous studies have identified common cellular elements that play a critical role in the regulation of fibrosis^45^. Ultimately, dysregulation of these same elements is responsible for the replacement of normal tissues with compromised or nonfunctional fibrotic tissue. The development of therapeutic strategies that limit the progression of fibrosis without adversely affecting the overall repair process would represent an important technological advancement^46^. The use of amniotic tissue allografts in severe burns and chronic wounds was originally intended to accelerate wound closure; however, an unexpected outcome of treatment was an improved quality of healing^47,48^. In a 30-patient pediatric burn case series, the return to normally functioning skin including dynamic compliance, movement, and color was superior in patients treated with DHACM grafts^47^. In addition to burns, amniotic membranes have also been shown to reduce scar tissue formation and enhance wound healing in diabetic foot ulcers and venous leg ulcers^9,49–51^. The mechanism of action by which amniotic membranes may satisfy this unmet need was explored in this study.

One of the primary cellular mediators of fibrosis is the fibroblast, which when activated becomes a myofibroblast and acquires a contractile phenotype with increased matrix synthesis. This process is largely regulated by the influx of TGFβ into a wound, which has been corroborated by experimental evidence including, cell biological studies, animal model experiments, and clinical evidence^52^. Establishing a bio-similar in vitro model for fibrosis requires appropriate cell types and signals; however, it also is highly dependent upon the organization of the microenvironment. Previous experiments evaluating DHACM utilized standard assays involving a cell monolayer in a matrix-poor system. The controls demonstrated elevated *COL1A* expression with TGFb1 treatment, but failed to capture the pathophysiology and complexity of the disease state^31^. The addition of DHACM counteracted the TGFb1 effect, but without a bio-similar model, the mechanism is regarded as largely speculative. However, the addition of Ficoll to the culture conditions in this study introduced volume exclusion to accelerate the enzymatic conversion of procollagen to insoluble collagen allowing for protein and post-translational modification analysis. This demonstrates the utility of this model as it is more representative of the in vivo disease state and thus, more appropriate for assessing the significance of a potential treatment. This study not only used a more physiologically relevant in vitro model to substantiate the previous data demonstrating an effect of DHACM on collagen type I biosynthesis but additionally investigated how DHACM and LHACM impacts collagen type I biosynthesis and deposition.

Whole mRNA profiles of MMC cultured fibroblasts treated with DHACM + TGFβ1 identified 3054 genes that were differentially expressed. Interestingly, many of the identified genes are related to myofibroblast differentiation, collagen synthesis and matrix regulation, making them potential therapeutic targets for fibrotic indications^37,53–59^. LHACM, like DHACM, is comprised of PURION® processed amnion and chorion membranes, but also retains the intermediate layer^30^. LHACM is expected to exhibit similar properties, but inclusion in this study provided a direct comparison to DHACM. Under MMC conditions, fibroblasts treated with TGFβ1 exhibited expected behavior: increased phosphorylation of SMAD2 and SMAD3, increased *ACTA2* and *CTGF* expression and elevated *COL1A1*. The addition of DHACM and LHACM reversed these effects, indicating their role in the regulation of downstream collagen accumulation and ECM degradation suseptibility^40,41,60^. TGFβ1 induced increased intracellular collagen synthesis and extracellular collagen deposition, which was attenuated with the addition of DHACM and LHACM treatment. Interestingly, visualization of collagen type I in the cell system revealed a retention of intracellular collagen and a marked reduction in extracellular collagen with treatment. In line with published results on other anti-fibrotic modalities, this data suggests that DHACM and LHACM may target gene expression, protein synthesis and secretion mechanisms of collagen to regulate the fibrotic process^61^.

Collagen maturation is facilitated by the extracellular crosslinking events which influence collagen deposition, matrix stability and rate of turnover. Regulation of these activities is important in assessing the prevention and irreversibility of fibrosis, as abnormalities in these crosslinking events have been directly linked to the clinical manifestation of stiffness and persistence in keloid and hypertrophic scars^62,63^. BMP1, LOX and TGase2 are the primary enzymes responsible for post-translational collagen modification and aberrant activity of these specifically have been implicated in fibrotic diseases^69–71^. TGFβ1 is known to be a key regulator of all three enzymes, however, in this study under MMC conditions, TGFβ1 increased *TGM2* expression, but did not increase *BMP1* and *LOX* gene expression ^64–66^. Despite these unexpected results, the protein levels did exhibit intracellular increases in BMP1, LOX and TGase2, validating the model. The discrepancy between gene expression and protein secretion is likely due to the temporal nature of gene regulation. An expanded time point analysis would be necessary to elucidate the impact of stimulation on the timing of gene regulation. The addition of DHACM and LHACM treatment to this system altered the expression of *BMP1* and demonstrated corresponding changes to BMP1 protein levels relative to the TGFβ1 stimulated cells. BMP1 regulation is important as it directly impacts TGFβ signaling and the downstream effects of LOX^67,68^.

The extracellular crosslinking events catalyzed by LOX are critical for the stabilization of extracellular collagen^43^. The absence of LOX-mediated crosslinking in vivo results in weak collagen fibers and fragile collagenous tissues^69^. Consequently, elevated LOX levels play a critical role in scar formation and maintenance^70^. LOX is synthesized as a ~ 50 kDa proenzyme that is secreted and extracellularly processed by BMP1 to a mature active enzyme of ~ 30 kDa^44^. Intracellularly, DHACM and LHACM reduce the expression of the LOX proenzyme. Additionally, a LOX protein band at the molecular weight of the mature enzyme was also detected in the cell lysate or intracellular space of TGFβ1 stimulated cells and was diminished by DHACM and LHACM treatment. Cell culture supernatant from TGFβ1 stimulated HDFs showed an increase in accumulation of the mature LOX enzyme; however, DHACM and LHACM treatment further increased levels found in the cell culture supernatant. The contrasting influence of DHACM and LHACM on *LOX* gene expression and LOX protein levels reflect the inherent complexity of tissue-based biologics. DHACM and LHACM may not only regulate the production of the proenzyme but also play a role in regulating the trafficking of mature LOX into the intracellular space. Even though processed extracellularly, LOX has been shown to have intracellular functions in the cytoplasm and nucleus. Nellaiappan et. al. previously reported that secreted and proteolytically processed-LOX can re-enter cells and concentrate within nuclei^71,72^. DHACM and LHACM treated groups have significantly less mature LOX in the intracellular space, which may correspond to the elevated extracellular levels. Growing evidence implicating LOX as not only a major player in ECM remodeling but of also having intracellular activities that enable cell communication and ECM remodeling supports the hypothesis that LHACM and DHACM may modulate LOX at multiple levels^71^. The rigidity of the ECM and subsequent resistance to turnover is modulated in part by the crosslinking actions of TGase2 which is known to be involved in several diseases^73–75^.TGase2 is a key player in wound healing and inflammation as well as in pathological states such as fibrosis and arthritis^76^. TGFβ1 stimulation of HDFs significantly increased the gene expression and protein level of TGase2 which was subsequently reduced with DHACM and LHACM treatment. This suggests that the resulting collagen matrix may be more susceptible to degradation. Taken together, DHACM and LHACM demonstrate a multi-faceted approach to influence collagen deposition, rigidity, and maturation through the regulation of enzymatic crosslinking actions. This data, in addition to other reports, suggest that by controlling the extent of crosslinking, the matrix is more susceptible to remodeling and less likely to form excessive scar tissue^57,77,78^.

Clinically, fibrosis is recognized as a major health challenge with an incidence of approximately 4968 per 100,000 person-year. The complex pathogenesis of fibrotic diseases complicates the development of safe and effective therapeutics^79^. These data corroborate the earlier findings in the monolayer culture experiments and provides insight into the multi-functional effects of DHACM and LHACM on the regulation of TGFβ1-induced collagen production and deposition, further expanding the potential role of amniotic tissue allografts as modulators of fibrosis. Amniotic tissue allografts have been used for many years and have been safe and well tolerated with no adverse complications offering an advantage over the use of traditional therapeutics with known adverse effects^8–14,80^. While additional preclinical animal models, clinical data and investigating alternative crosslinking pathways will be beneficial in determining how the in vitro characteristics of DHACM and LHACM may translate to clinical efficacy, these data further expand the understanding of the regulatory capabilities of DHACM and LHACM on fibrotic pathways and may solve for a significant unmet clinical need.

### Supplementary Information

Supplementary Information.

## Data Availability

RNA-seq data used in this study are deposited in the Gene Expression Omnibus (GEO, https://www.ncbi.nlm.nih.gov/geo/) under the accession number GSE247237.
