# Supplementary material for: Human amniotic membrane modulates collagen production and deposition in vitro
Source: Sci Rep. 2024 Jul 10;14:15998. doi: 10.1038/s41598-024-64364-2 (PMC11237048; doi:10.1038/s41598-024-64364-2)
Supplement: Supplementary file 1 — Supplementary Information. [file 41598_2024_64364_MOESM1_ESM.pdf]

Human Amniotic Membrane Modulates Collagen Production and Deposition *In Vitro*

Sarah E. Moreno[1, 2], Isioma Enwerem-Lackland Ph.D.[1, 2], Kristiana Dreaden[2, 3], Michelle Massee[1*], Thomas J. Koob Ph.D.[1], and John R. Harper Ph.D.[1]

[1]MiMedx Group, Inc. 1775 West Oak Commons Court NE, Marietta, Georgia 30062
[2]Authors contributed equally to this work
[3] Alkermes 900 Winter St. Waltham, Massachusetts 02451
*Corresponding author: Michelle Massee, mmassee@mimedx.com

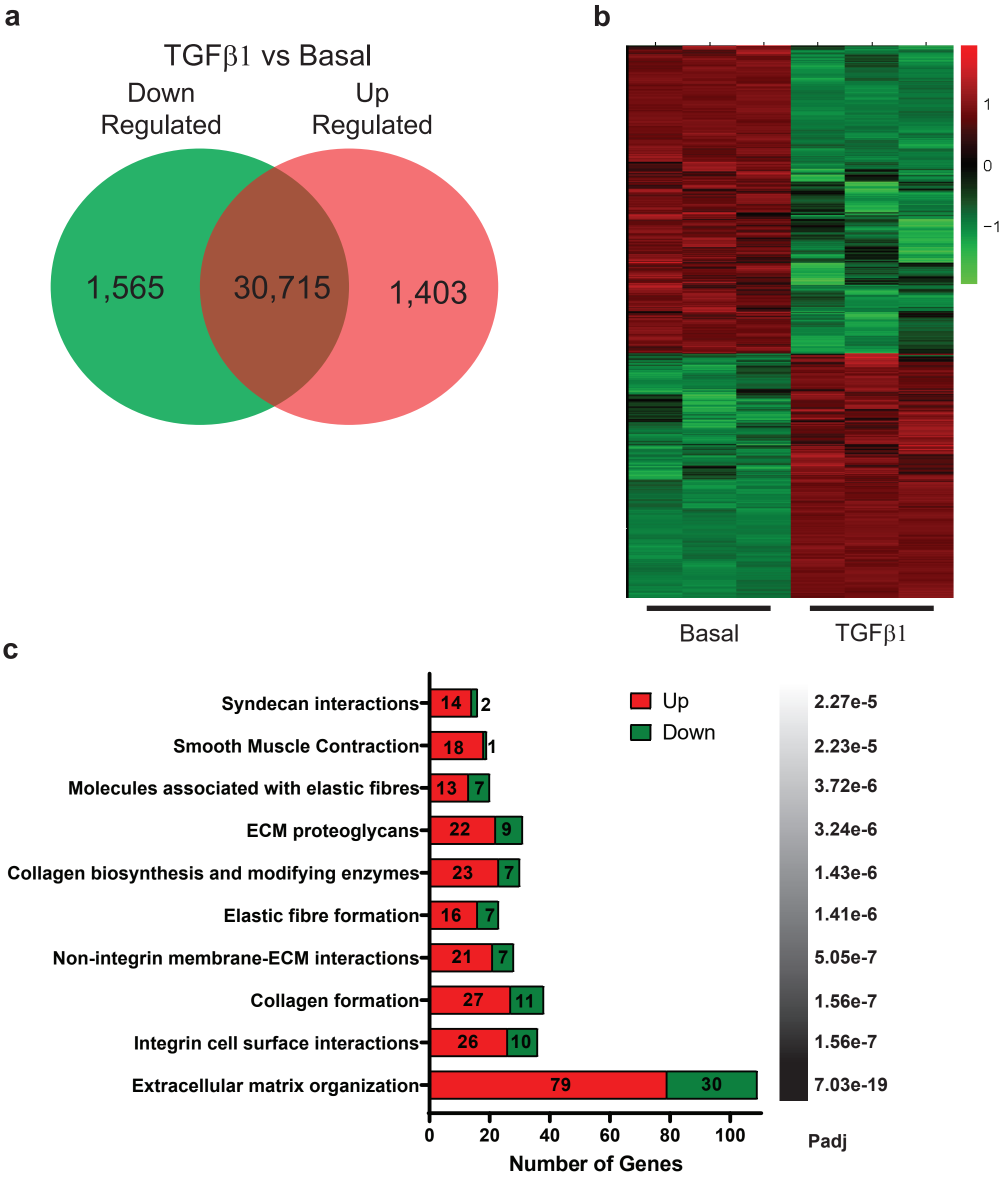

Supplementary Figure S1. Differential gene expression patterns between TGFb and basal. (a) Venn diagram highlighting the DEGs. (b) Heatmap of DEGs showing relative expression levels from highest (red) to lowest (green). (c) Reactome Pathway analysis of dysregulated genes. Functional annotation of the top 10 pathways and genes upregulated (red) or downregulated (green) in each cluster.

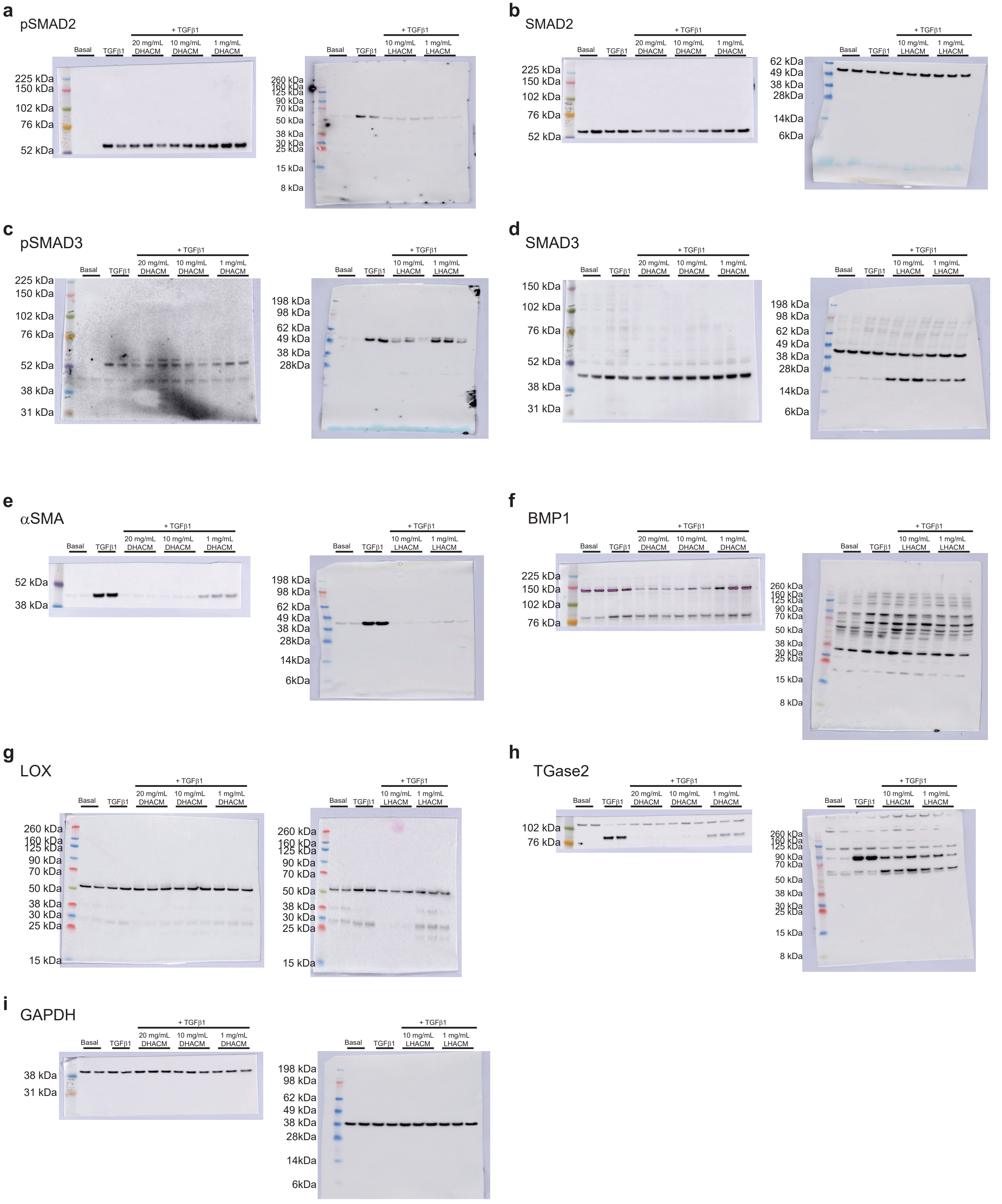

Supplementary Figure S2. Original western blot images. Cell lysates were resolved using SDS-PAGE followed by western blot analysis of (a) pSMAD2, (b) SMAD2, (c) pSMAD3, (d) SMAD3, (e) αSMA, (f) BMP1, (g) LOX, (h) TGase, and (i) GAPDH.

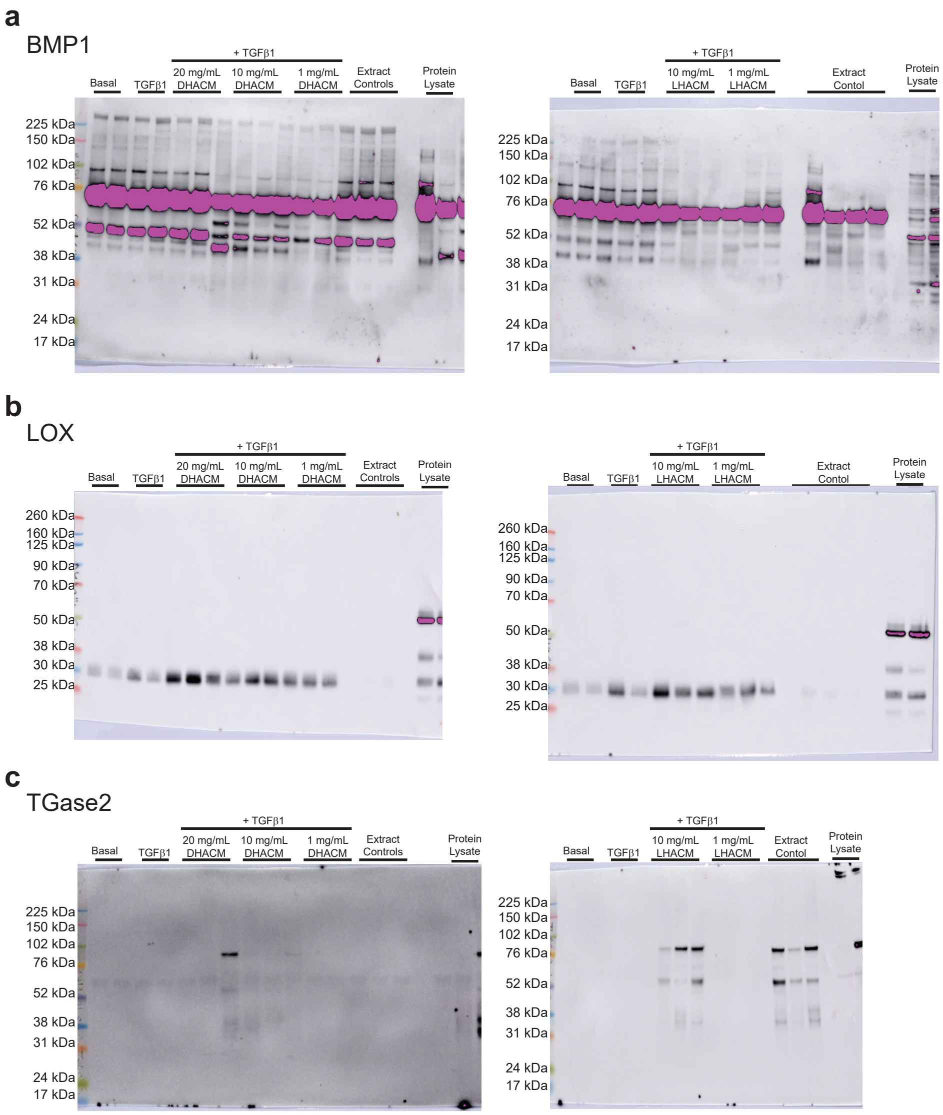

Supplementary Figure S3. Original western blot images. Cell culture supernatants were resolved using SDS-PAGE followed by western blot analysis of (a) BMP1, (b) LOX, and (c) TGase2. LHACM and DHACM extract and cell lysate of TGFβ1 treated cells were used as positive control for each western blot.

Supplementary Table S1: DEG list of the comparision of TGFβ1 to basal groups

gene_id	gene_name	TGFB_AVG	BASAL_AVG	log2FoldChange	pvalue	padj
ENSG00000115414	FN1	1918529.638	793534.0062	1.273634749	0	0
ENSG00000108821	COL1A1	1017878.115	279099.1852	1.866707822	0	0
ENSG00000137801	THBS1	298769.5119	123697.9014	1.272225716	0	0
ENSG00000011465	DCN	27520.83956	123729.218	-2.168540432	0	0
ENSG00000196924	FLNA	176947.0577	65445.44174	1.434913099	0	0
ENSG00000111799	COL12A1	168628.3912	46453.42749	1.859951026	0	0
ENSG00000113140	SPARC	172002.4597	30013.69854	2.518711728	0	0
ENSG00000100345	MYH9	109683.3952	28086.27034	1.965420861	0	0
ENSG00000119681	LTBP2	67314.23767	15857.83417	2.085695117	0	0
ENSG00000122786	CALD1	75828.57848	17365.06874	2.126581019	0	0
ENSG00000084234	APLP2	18705.97486	49207.15774	-1.395365234	0	0
ENSG00000106624	AEBP1	54187.07968	19484.12415	1.475623604	0	0
ENSG00000204262	COL5A2	44291.83655	11648.41274	1.926682993	0	0
ENSG00000120708	TGFBI	52120.58002	15731.02951	1.728257453	0	0
ENSG00000137809	ITGA11	74296.68986	4547.726725	4.029779854	0	0
ENSG00000140416	TPM1	43624.506	5872.882148	2.89226706	0	0
ENSG00000146674	IGFBP3	74659.60643	11220.92003	2.733925809	0	0
ENSG00000118523	CTGF	59383.90743	4207.186726	3.819998589	0	0
ENSG00000169604	ANTXR1	25428.65615	4946.94054	2.362239958	0	0
ENSG00000145730	PAM	7336.210598	29952.02756	-2.029488694	0	0
ENSG00000185567	AHNAK2	10660.47593	39522.68094	-1.890619974	0	0
ENSG00000144810	COL8A1	23026.79696	2627.254155	3.132119805	0	0
ENSG00000130508	PXDN	25033.68452	7694.789424	1.70157938	0	0
ENSG00000172061	LRRC15	14217.70625	2125.045936	2.742519793	0	0
ENSG00000072110	ACTN1	27118.93106	7809.532652	1.796172942	0	0
ENSG00000139926	FRMD6	20195.92737	6574.583737	1.619236968	0	0
ENSG00000164694	FNDC1	17893.52006	781.8385956	4.516210337	0	0
ENSG00000148848	ADAM12	24260.66911	2830.43885	3.099209632	0	0
ENSG00000101335	MYL9	27688.83189	10010.93057	1.467865866	0	0
ENSG00000106366	SERPINE1	19255.17183	4115.891895	2.225854759	0	0
ENSG00000114270	COL7A1	8082.070816	1338.670094	2.592059078	0	0
ENSG00000198467	TPM2	28696.41233	5287.599921	2.440786589	0	0
ENSG00000134531	EMP1	5286.367562	15641.44552	-1.564950467	0	0
ENSG00000107957	SH3PXD2A	23830.63184	4102.377944	2.537737234	0	0
ENSG00000038427	VCAN	26662.7152	7516.065932	1.826975873	0	0
ENSG00000112655	PTK7	17698.13922	5480.246718	1.69160562	0	0
ENSG00000129116	PALLD	27215.19503	4931.991368	2.464672811	0	0
ENSG00000133110	POSTN	20789.8348	693.2086758	4.902222365	0	0
ENSG00000082781	ITGB5	21064.93749	7694.719116	1.452836919	0	0
ENSG00000168477	TNXB	163.33001	30118.71962	-7.52753989	0	0
ENSG00000148926	ADM	1666.396892	8518.207953	-2.354048618	0	0
ENSG00000149591	TAGLN	32655.29056	2421.464725	3.752883442	0	0
ENSG00000183255	PTTG1IP	19158.61897	7135.70029	1.424724631	0	0
ENSG00000013297	CLDN11	75.5129268	6757.761864	-6.486675249	0	0
ENSG00000149451	ADAM33	2934.653322	11973.07009	-2.028912374	0	0
ENSG00000182492	BGN	15774.3316	971.6029174	4.021657616	0	0
ENSG00000107796	ACTA2	31210.04122	1035.110901	4.910860229	0	0
ENSG00000162407	PLPP3	2687.312947	14207.35975	-2.402299906	0	0
ENSG00000154856	APCDD1	378.1660611	4064.078202	-3.424584897	0	0
ENSG00000134871	COL4A2	16072.08465	1647.91452	3.284246663	0	0
ENSG00000123500	COL10A1	18401.52912	114.0253454	7.340150254	0	0
ENSG00000143341	HMCN1	13805.37969	3393.962853	2.023364318	0	0
ENSG00000105664	COMP	23412.56243	4028.577219	2.538559451	0	0
ENSG00000135318	NT5E	3038.093269	9739.258573	-1.680622795	0	0
ENSG00000058668	ATP2B4	3433.721172	13244.12963	-1.947546933	0	0
ENSG00000064666	CNN2	13180.37033	4054.168474	1.700963902	0	0
ENSG00000086062	B4GALT1	9710.503787	1914.242519	2.343900603	0	0
ENSG00000117519	CNN3	10989.08503	3496.396945	1.652552264	0	0
ENSG00000166741	NNMT	11995.91351	2658.247895	2.173507596	0	0
ENSG00000122641	INHBA	7116.524432	736.5036485	3.274685207	0	0
ENSG00000136960	ENPP2	891.9195863	8345.564472	-3.226251583	0	0
ENSG00000049540	ELN	19001.01825	1172.473311	4.018943205	0	0
ENSG00000156804	FBXO32	13837.45631	1063.308178	3.702363298	0	0

ENSG00000133816	MICAL2	10060.38923	2268.093385	2.148303484	0	0
ENSG00000039560	RAI14	6368.516086	1260.138999	2.337248302	0	0
ENSG00000187498	COL4A1	12291.08491	688.8184143	4.154516198	0	0
ENSG00000103489	XYLT1	9187.98764	1512.624345	2.601409224	0	0
ENSG00000115884	SDC1	11746.38277	757.3888237	3.95442678	0	0
ENSG00000139211	AMIGO2	10545.57112	396.27237	4.729052934	0	0
ENSG00000103196	CRISPLD2	7280.112043	862.3441691	3.078522806	0	0
ENSG00000171812	COL8A2	11948.06614	1953.290343	2.612809953	0	0
ENSG00000171617	ENC1	8452.071112	254.233708	5.051420678	0	0
ENSG00000159176	CSRP1	7518.292003	2109.896602	1.832945186	0	0
ENSG00000167779	IGFBP6	1208.232304	9386.531882	-2.957109161	0	0
ENSG00000196923	PDLIM7	8188.924775	1017.974304	3.007385942	0	0
ENSG00000143320	CRABP2	694.5507125	4063.966155	-2.548751794	0	0
ENSG00000118898	PPL	184.7434162	8943.007468	-5.595188265	0	0
ENSG00000183688	RFLNB	6242.523181	770.2607602	3.022572045	0	0
ENSG00000103257	SLC7A5	9180.354996	852.9211859	3.426770931	0	0
ENSG00000176170	SPHK1	5086.295354	822.3215216	2.628252177	0	0
ENSG00000164932	CTHRC1	3808.01779	675.9678626	2.495903605	0	0
ENSG00000124225	PMEPA1	4535.943652	288.8647072	3.976916368	0	0
ENSG00000172348	RCAN2	729.0451188	5827.512714	-2.997564958	0	0
ENSG00000107731	UNC5B	4604.493364	963.7810139	2.254147344	0	0
ENSG00000133687	TMTC1	345.8717746	3369.139652	-3.282183438	0	0
ENSG00000138623	SEMA7A	6198.334208	848.5655006	2.868049693	0	0
ENSG00000049323	LTBP1	3065.954391	348.8437693	3.133824304	0	0
ENSG00000137124	ALDH1B1	6126.432451	383.5993059	4.001031793	0	0
ENSG00000115129	TP53I3	5779.981129	483.174476	3.579292811	0	0
ENSG00000165617	DACT1	3133.60595	84.63077332	5.226157197	0	0
ENSG00000198959	TGM2	7348.590567	57.56093758	7.004917759	0	0
ENSG00000187955	COL14A1	751.5143987	4565.840291	-2.60280958	0	0
ENSG00000265972	TXNIP	288.408078	3135.207674	-3.443041208	0	0
ENSG00000106538	RARRES2	2749.054185	54.80987678	5.633560561	0	0
ENSG00000185585	OLFML2A	154.8718387	4271.863004	-4.787606213	0	0
ENSG00000124212	PTGIS	398.8648292	3595.620388	-3.170914803	0	0
ENSG00000162849	KIF26B	3972.50377	388.5916597	3.350406329	0	0
ENSG00000135074	ADAM19	2451.841563	283.1558948	3.114400224	0	0
ENSG00000070404	FSTL3	3640.031666	535.4419173	2.764424737	0	0
ENSG00000060718	COL11A1	4380.46457	96.15217302	5.518885619	0	0
ENSG00000214517	PPME1	3215.009933	387.9411298	3.047577281	0	0
ENSG00000145681	HAPLN1	4606.668308	41.6879948	6.779144335	0	0
ENSG00000164176	EDIL3	2075.452521	83.10790753	4.651660797	0	0
ENSG00000158008	EXTL1	2592.359375	30.43683832	6.416572438	0	0
ENSG00000096696	DSP	14912.5236	3607.74887	2.047720734	8.18E-308	1.66E-305
ENSG00000182667	NTM	3543.108424	627.9847226	2.496662232	2.84E-304	5.70E-302
ENSG00000105974	CAV1	4974.273796	15883.39579	-1.67472023	9.75E-304	1.94E-301
ENSG00000206190	ATP10A	4587.268355	926.1248195	2.308974439	2.84E-302	5.58E-300
ENSG00000134986	NREP	3406.456458	636.1711401	2.419969187	4.85E-300	9.45E-298
ENSG00000197747	S100A10	1715.495691	9586.452562	-2.481973949	1.39E-298	2.68E-296
ENSG00000069702	TGFBR3	423.6238503	4123.587519	-3.283790496	2.46E-296	4.71E-294
ENSG00000082482	KCNK2	2031.709874	7690.705384	-1.920448758	6.28E-296	1.19E-293
ENSG00000075624	ACTB	147586.6853	61526.61227	1.26229609	1.74E-291	3.28E-289
ENSG00000158270	COLEC12	1175.29008	5472.674163	-2.21853982	1.55E-290	2.89E-288
ENSG00000079308	TNS1	12337.12237	4615.05096	1.418730943	4.87E-290	9.00E-288
ENSG00000130402	ACTN4	22232.66606	8320.669951	1.417764752	3.33E-284	6.10E-282
ENSG00000188783	PRELP	3933.757677	14225.2921	-1.854162702	3.26E-283	5.93E-281
ENSG00000163453	IGFBP7	4686.449516	806.9121053	2.537368728	1.83E-281	3.30E-279
ENSG00000150687	PRSS23	17680.37637	5455.827963	1.696489603	4.95E-279	8.85E-277
ENSG00000118257	NRP2	4125.499518	874.0577892	2.239570997	5.21E-274	9.23E-272
ENSG00000165124	SVEP1	3643.515812	9994.68565	-1.455664489	2.39E-273	4.20E-271
ENSG00000152518	ZFP36L2	1710.941014	7594.504357	-2.150506162	7.62E-271	1.33E-268
ENSG00000130176	CNN1	2219.61377	167.6046397	3.733803466	2.54E-267	4.39E-265
ENSG00000116679	IVNS1ABP	4231.682732	744.2048824	2.508857576	9.47E-265	1.62E-262
ENSG00000149257	SERPINH1	20163.88483	6910.310074	1.544784321	4.18E-264	7.11E-262
ENSG00000163110	PDLIM5	4892.196911	1079.323794	2.182695845	5.32E-261	8.98E-259
ENSG00000176438	SYNE3	204.0127304	2383.402241	-3.543562735	8.69E-259	1.46E-256
ENSG00000166923	GREM1	74989.04837	34388.69262	1.124843986	1.53E-257	2.54E-255
ENSG00000006327	TNFRSF12A	5653.505844	1643.078904	1.783493829	4.78E-257	7.88E-255

ENSG00000122966	CIT	195.0189391	2038.592241	-3.385763175	1.86E-256	3.04E-254
ENSG00000198542	ITGBL1	9130.859485	3406.139524	1.422327498	4.44E-255	7.20E-253
ENSG00000115419	GLS	13634.15359	4228.611923	1.689476345	8.45E-255	1.36E-252
ENSG00000172757	CFL1	17380.56751	7667.514071	1.180714838	1.89E-254	3.02E-252
ENSG00000109610	SOD3	351.0973101	3176.167997	-3.17657015	1.51E-251	2.39E-249
ENSG00000156466	GDF6	1592.51898	31.73616683	5.669636171	7.46E-251	1.17E-248
ENSG00000157227	MMP14	72746.41144	28486.4642	1.352582759	4.23E-247	6.61E-245
ENSG00000026025	VIM	49657.48705	127724.8642	-1.362952879	2.50E-246	3.87E-244
ENSG00000152952	PLOD2	4809.817607	918.5000573	2.390810099	2.82E-244	4.34E-242
ENSG00000157168	NRG1	2920.396771	414.4281075	2.820331532	1.74E-240	2.66E-238
ENSG00000180914	OXTR	2646.666474	457.7896637	2.530162735	3.33E-240	5.05E-238
ENSG00000136826	KLF4	186.3441246	2033.025469	-3.449795673	8.94E-240	1.35E-237
ENSG00000099860	GADD45B	3274.544029	586.1324329	2.48386599	1.91E-238	2.86E-236
ENSG00000004399	PLXND1	8390.374942	3244.204118	1.370440181	3.26E-234	4.85E-232
ENSG00000128016	ZFP36	651.2065083	3528.846166	-2.438766506	1.45E-233	2.14E-231
ENSG00000185008	ROBO2	84.30460073	1577.801355	-4.228860333	5.66E-230	8.29E-228
ENSG00000135842	FAM129A	333.2474657	2325.616237	-2.803405201	2.17E-228	3.16E-226
ENSG00000100644	HIF1A	9214.074458	2811.655541	1.711711524	8.20E-228	1.19E-225
ENSG00000135047	CTSL	3828.922377	9641.713768	-1.332243833	1.44E-226	2.07E-224
ENSG00000026508	CD44	5195.175742	13998.8578	-1.429655294	1.31E-225	1.87E-223
ENSG00000233608	TWIST2	1605.797737	5549.656496	-1.789214891	3.36E-225	4.76E-223
ENSG00000213626	LBH	6794.650793	1955.340574	1.797483994	4.07E-225	5.73E-223
ENSG00000125398	SOX9	1227.236018	36.05744394	5.098239508	3.20E-224	4.48E-222
ENSG00000163431	LMOD1	2515.666948	446.5687617	2.497562372	4.34E-224	6.03E-222
ENSG00000175183	CSRP2	3170.22053	761.5233125	2.057847426	4.64E-224	6.41E-222
ENSG00000177283	FZD8	1455.596387	114.8566103	3.658443744	4.67E-222	6.41E-220
ENSG00000150093	ITGB1	59409.1591	28120.48226	1.079126445	9.91E-221	1.35E-218
ENSG00000101846	STS	825.6513225	4009.917448	-2.280675322	2.12E-220	2.87E-218
ENSG00000182240	BACE2	3813.553006	875.644434	2.123190443	2.92E-219	3.94E-217
ENSG00000140937	CDH11	13498.8595	5666.996206	1.252232762	4.00E-219	5.35E-217
ENSG00000156535	CD109	6817.393297	18526.2325	-1.442324249	3.92E-217	5.21E-215
ENSG00000109861	CTSC	236.7141637	2164.984113	-3.192752419	5.41E-217	7.15E-215
ENSG00000092841	MYL6	24344.85073	10343.66523	1.235152913	1.03E-216	1.35E-214
ENSG00000240583	AQP1	6177.557048	1737.840975	1.830688162	2.46E-213	3.20E-211
ENSG00000166147	FBN1	73513.97004	30675.22879	1.260909984	6.59E-211	8.54E-209
ENSG00000107738	VSIR	455.7852731	2561.142093	-2.491498125	9.96E-210	1.28E-207
ENSG00000041982	TNC	107311.3788	42243.39626	1.344981651	1.05E-209	1.34E-207
ENSG00000154175	ABI3BP	459.5697646	2979.752649	-2.698504985	3.81E-209	4.85E-207
ENSG00000187134	AKR1C1	200.1486213	1728.884491	-3.112115659	2.01E-207	2.55E-205
ENSG00000166825	ANPEP	4959.262441	11673.72439	-1.235302831	1.36E-206	1.71E-204
ENSG00000087245	MMP2	113442.7863	57382.40529	0.983227278	3.01E-204	3.76E-202
ENSG00000169756	LIMS1	4987.445546	1057.524723	2.239649308	3.02E-202	3.76E-200
ENSG00000071575	TRIB2	2061.461609	316.4981554	2.702988182	6.97E-202	8.62E-200
ENSG00000075213	SEMA3A	325.883865	2108.540181	-2.693954355	8.95E-202	1.10E-199
ENSG00000064042	LIMCH1	1809.935443	252.3921736	2.838271521	7.43E-200	9.07E-198
ENSG00000115461	IGFBP5	2890.906511	10546.27522	-1.866647973	4.78E-199	5.80E-197
ENSG00000197043	ANXA6	13889.10192	6107.313542	1.18506827	3.82E-197	4.62E-195
ENSG00000170801	HTRA3	113.7106517	1548.854107	-3.770855881	8.68E-196	1.04E-193
ENSG00000011028	MRC2	23163.8834	10865.94848	1.091991152	5.94E-195	7.10E-193
ENSG00000120594	PLXDC2	2200.234762	364.0612156	2.596801603	1.04E-194	1.23E-192
ENSG00000111817	DSE	6082.340314	1859.375358	1.711210708	3.35E-194	3.95E-192
ENSG00000164574	GALNT10	6000.251763	2211.326476	1.440625529	7.76E-194	9.10E-192
ENSG00000177363	LRRN4CL	1190.166763	4630.273374	-1.960393628	7.79E-194	9.10E-192
ENSG00000165757	JCAD	6199.535626	2321.231182	1.417737717	3.76E-191	4.37E-189
ENSG00000163931	TKT	3228.799349	8284.833657	-1.359612233	2.77E-190	3.20E-188
ENSG00000115306	SPTBN1	5429.44317	11799.54452	-1.120066093	4.58E-190	5.27E-188
ENSG00000113657	DPYSL3	9300.036658	4149.718292	1.164236408	9.00E-190	1.03E-187
ENSG00000121769	FABP3	519.4607927	2806.184441	-2.434646516	2.96E-189	3.36E-187
ENSG00000159216	RUNX1	6050.221959	2187.090761	1.46838699	7.19E-189	8.13E-187
ENSG00000130635	COL5A1	80494.11294	14932.14776	2.430397536	1.04E-188	1.18E-186
ENSG00000135048	CEMIP2	2585.415427	529.8706088	2.288190026	1.49E-186	1.66E-184
ENSG00000179222	MAGED1	6461.791403	2487.409853	1.377041999	2.40E-186	2.68E-184
ENSG00000115594	IL1R1	1129.735237	4188.017414	-1.890408322	9.39E-185	1.04E-182
ENSG00000149596	JPH2	3208.107038	624.4828658	2.358742875	3.86E-184	4.26E-182
ENSG00000164236	ANKRD33B	109.3081145	1305.375794	-3.577587598	7.91E-184	8.67E-182
ENSG00000138448	ITGAV	13188.62776	5339.263864	1.305190803	3.47E-183	3.78E-181

ENSG00000038382	TRIO	10907.65871	4964.971832	1.135026041	2.94E-181	3.19E-179
ENSG00000119280	C1orf198	10124.92966	3802.612816	1.412324464	2.61E-180	2.82E-178
ENSG00000128487	SPECC1	1596.266028	247.8229199	2.68530702	2.68E-180	2.88E-178
ENSG00000223749	MIR503HG	1400.395835	169.8016031	3.047137886	1.73E-179	1.85E-177
ENSG00000154380	ENAH	5699.612764	2172.959876	1.391665344	3.04E-179	3.24E-177
ENSG00000112977	DAP	6035.283402	2359.545047	1.355676153	2.54E-178	2.69E-176
ENSG00000124942	AHNAK	56070.07729	136332.9931	-1.281866298	8.25E-178	8.69E-176
ENSG00000155324	GRAMD2B	3662.669489	1033.467832	1.824452665	3.40E-177	3.56E-175
ENSG00000113494	PRLR	24.08829047	1480.655712	-5.944430786	1.10E-175	1.15E-173
ENSG00000183087	GAS6	50796.4732	21441.41896	1.244179276	4.28E-175	4.44E-173
ENSG00000112769	LAMA4	5368.184716	12372.89004	-1.204691132	7.04E-173	7.27E-171
ENSG00000145358	DDIT4L	38.58018721	1302.502752	-5.080620765	2.51E-172	2.58E-170
ENSG00000099953	MMP11	2827.979941	463.4850336	2.612857734	1.65E-171	1.69E-169
ENSG00000110092	CCND1	13244.68708	26600.3386	-1.006089898	2.51E-171	2.55E-169
ENSG00000104415	WISP1	3337.20495	1003.745947	1.733072469	5.42E-170	5.49E-168
ENSG00000211455	STK38L	2301.053614	491.4977053	2.227708168	9.62E-169	9.70E-167
ENSG00000017483	SLC38A5	1379.288183	184.8506467	2.895151738	1.50E-168	1.50E-166
ENSG00000218336	TENM3	3501.652249	1101.390732	1.667599367	9.65E-168	9.64E-166
ENSG00000134352	IL6ST	5281.979266	13250.15085	-1.326560128	2.67E-166	2.65E-164
ENSG00000164692	COL1A2	534334.2604	305621.5387	0.805999923	3.09E-165	3.05E-163
ENSG00000136048	DRAM1	3650.174802	9282.193441	-1.346341049	6.31E-165	6.21E-163
ENSG00000197965	MPZL1	5073.325946	2009.817456	1.336466559	1.18E-164	1.15E-162
ENSG00000183287	CCBE1	538.9470053	2658.204437	-2.303578117	9.21E-164	8.98E-162
ENSG00000071282	LMCD1	4888.881343	1698.865561	1.525640603	1.31E-163	1.28E-161
ENSG00000104881	PPP1R13L	1667.448426	282.6757366	2.555329814	7.76E-163	7.50E-161
ENSG00000112096	SOD2	7944.393503	2558.818792	1.635022931	2.55E-162	2.46E-160
ENSG00000146648	EGFR	1574.149114	4381.493157	-1.476871164	8.31E-162	7.96E-160
ENSG00000101825	MXRA5	28977.31797	11458.0863	1.338537568	1.63E-161	1.55E-159
ENSG00000137309	HMGA1	907.3400565	3020.472939	-1.734938836	1.61E-159	1.52E-157
ENSG00000106976	DNM1	877.447473	3314.255711	-1.918002112	5.04E-159	4.76E-157
ENSG00000164465	DCBLD1	1670.466744	339.1796993	2.300705298	9.74E-158	9.16E-156
ENSG00000249992	TMEM158	211.460134	1666.986052	-2.981311194	3.24E-157	3.03E-155
ENSG00000213949	ITGA1	3209.059117	989.1828999	1.698252599	1.54E-155	1.44E-153
ENSG00000211448	DIO2	933.4300482	33.25644169	4.816505728	1.91E-155	1.77E-153
ENSG00000167460	TPM4	43640.72724	24836.00608	0.813126941	1.90E-154	1.75E-152
ENSG00000074590	NUAK1	2757.286979	578.1358823	2.249762299	5.94E-154	5.47E-152
ENSG00000163520	FBLN2	9064.844435	19354.141	-1.094364716	1.61E-153	1.47E-151
ENSG00000160932	LY6E	2315.861786	5925.425198	-1.355308416	4.26E-153	3.89E-151
ENSG00000232679	LINC01705	930.9554197	50.02312727	4.230752403	4.55E-152	4.14E-150
ENSG00000134853	PDGFRA	3066.119544	7080.619822	-1.207799181	7.78E-152	7.04E-150
ENSG00000090339	ICAM1	919.5690637	72.32113712	3.674990014	1.52E-151	1.37E-149
ENSG00000159840	ZYX	7969.090468	3295.816075	1.274057055	8.51E-150	7.63E-148
ENSG00000074527	NTN4	97.51548122	1135.3031	-3.539017717	1.45E-148	1.29E-146
ENSG00000140092	FBLN5	10444.87326	5253.965591	0.991393428	1.85E-148	1.64E-146
ENSG00000134198	TSPAN2	830.2386227	18.8549069	5.45424986	5.62E-148	4.98E-146
ENSG00000110651	CD81	6871.303194	14884.89215	-1.115146417	6.59E-148	5.81E-146
ENSG00000103175	WFDC1	1051.127776	12.29688501	6.401570349	1.36E-147	1.19E-145
ENSG00000143196	DPT	4881.541557	1861.787453	1.391336671	9.24E-147	8.08E-145
ENSG00000117298	ECE1	1124.476471	4042.616783	-1.847072409	1.50E-146	1.31E-144
ENSG00000164920	OSR2	251.2156077	1535.613718	-2.612989746	1.83E-146	1.59E-144
ENSG00000117122	MFAP2	4548.968635	1490.5408	1.609997612	1.23E-144	1.06E-142
ENSG00000142552	RCN3	7600.746329	2981.163131	1.350012431	1.76E-144	1.52E-142
ENSG00000127241	MASP1	606.1029993	2413.376901	-1.99368377	3.22E-143	2.76E-141
ENSG00000065534	MYLK	13773.62989	7365.789332	0.903060607	1.51E-142	1.29E-140
ENSG00000132000	PODNL1	1736.525613	346.8093934	2.322666596	3.24E-142	2.75E-140
ENSG00000196155	PLEKHG4	90.7245913	1046.373519	-3.530303867	2.48E-140	2.10E-138
ENSG00000231924	PSG1	159.4336106	1244.542555	-2.966841206	1.24E-139	1.05E-137
ENSG00000175220	ARHGAP1	5691.216398	2537.845229	1.165393307	5.56E-139	4.67E-137
ENSG00000139289	PHLDA1	228.6298513	1470.762482	-2.685111098	1.23E-137	1.03E-135
ENSG00000152049	KCNE4	958.7986429	102.7664046	3.228712332	6.78E-137	5.65E-135
ENSG00000070961	ATP2B1	1979.961817	5731.625757	-1.53345281	8.52E-137	7.07E-135
ENSG00000064205	WISP2	304.0221159	1692.140867	-2.475895101	1.04E-136	8.58E-135
ENSG00000197635	DPP4	9001.091732	18596.37041	-1.046919095	5.29E-136	4.36E-134
ENSG00000174136	RGMB	541.0444592	2179.966761	-2.011088993	1.20E-135	9.89E-134
ENSG00000136153	LMO7	13259.51613	6487.656295	1.031125902	2.75E-135	2.25E-133
ENSG00000196352	CD55	4380.478917	1793.73266	1.288354043	1.01E-134	8.26E-133

ENSG00000103187	COTL1	3433.845106	1119.725473	1.617305641	1.76E-134	1.43E-132
ENSG00000173114	LRRN3	727.06353	16.88970667	5.415439885	7.96E-134	6.43E-132
ENSG00000132561	MATN2	35.15931851	889.4741295	-4.658518778	1.34E-133	1.08E-131
ENSG00000131236	CAP1	7222.844336	3133.254973	1.204432316	1.39E-133	1.11E-131
ENSG00000169018	FEM1B	3878.377425	1408.095935	1.462734736	3.17E-133	2.53E-131
ENSG00000150593	PDCD4	653.0126533	2314.029532	-1.824764137	3.39E-133	2.70E-131
ENSG00000122420	PTGFR	193.891386	1463.869636	-2.918207569	6.66E-133	5.28E-131
ENSG00000081377	CDC14B	1062.473746	3016.970832	-1.505988174	1.68E-132	1.33E-130
ENSG00000151414	NEK7	10170.80464	3955.806599	1.362540132	6.55E-132	5.16E-130
ENSG00000163297	ANTXR2	2391.490219	5378.849451	-1.169035316	1.82E-131	1.43E-129
ENSG00000177425	PAWR	2464.327721	662.7277114	1.896821927	9.98E-131	7.80E-129
ENSG00000152580	IGSF10	22.3254884	957.2716324	-5.416709295	1.74E-129	1.35E-127
ENSG00000143387	CTSK	36944.55436	67531.78498	-0.870193136	1.01E-127	7.85E-126
ENSG00000147224	PRPS1	2012.79566	404.6706247	2.310869525	1.07E-126	8.29E-125
ENSG00000114850	SSR3	7713.059746	2856.174092	1.434229991	2.86E-126	2.20E-124
ENSG00000116132	PRRX1	6201.747312	2708.823547	1.196154219	3.79E-126	2.91E-124
ENSG00000106537	TSPAN13	901.5389785	107.7829713	3.065998384	1.00E-125	7.67E-124
ENSG00000168374	ARF4	8793.910713	4149.902705	1.083701604	1.82E-125	1.38E-123
ENSG00000159251	ACTC1	1788.37761	441.7668964	2.01631252	4.61E-125	3.50E-123
ENSG00000117394	SLC2A1	1634.870185	419.1199405	1.964200254	5.23E-125	3.95E-123
ENSG00000197321	SVIL	837.2316028	2771.602797	-1.72686813	9.42E-124	7.10E-122
ENSG00000010295	IFFO1	915.8451215	2828.885945	-1.627526482	1.92E-123	1.45E-121
ENSG00000075275	CELSR1	760.6415909	11.12338353	6.129016748	2.17E-122	1.62E-120
ENSG00000071127	WDR1	10658.22516	5339.35591	0.997108144	6.21E-122	4.63E-120
ENSG00000229847	EMX2OS	1200.55325	3739.722244	-1.638761311	7.95E-122	5.91E-120
ENSG00000180155	LYNX1	1069.521635	2973.255183	-1.474919857	9.40E-122	6.96E-120
ENSG00000168003	SLC3A2	3395.159061	1240.726317	1.451470506	5.97E-121	4.41E-119
ENSG00000106683	LIMK1	4073.096223	1549.16971	1.396443315	1.11E-120	8.14E-119
ENSG00000154556	SORBS2	707.7962048	17.77350423	5.343436468	1.27E-120	9.28E-119
ENSG00000132205	EMILIN2	277.5602271	1423.215658	-2.35950636	1.82E-120	1.33E-118
ENSG00000145362	ANK2	431.0823065	1732.241955	-2.006986152	3.58E-120	2.60E-118
ENSG00000077549	CAPZB	6990.512103	3590.553854	0.96117263	6.17E-120	4.48E-118
ENSG00000106211	HSPB1	9350.107143	4970.037876	0.911454158	2.53E-119	1.83E-117
ENSG00000116260	QSOX1	5819.096163	11357.86414	-0.96494108	6.44E-119	4.64E-117
ENSG00000137076	TLN1	23363.5958	13214.37471	0.822272598	6.63E-119	4.76E-117
ENSG00000049130	KITLG	888.6559332	2655.775496	-1.579716568	2.03E-118	1.45E-116
ENSG00000167468	GPX4	2408.005595	5674.958679	-1.237237063	3.77E-118	2.69E-116
ENSG00000158859	ADAMTS4	741.1779195	56.74891832	3.705048875	2.12E-117	1.51E-115
ENSG00000031081	ARHGAP31	2245.522446	727.6524332	1.625239353	2.69E-117	1.90E-115
ENSG00000087116	ADAMTS2	5690.845653	2431.101776	1.226235709	5.78E-117	4.08E-115
ENSG00000135269	TES	1887.844993	505.3706453	1.903927433	7.38E-117	5.20E-115
ENSG00000157766	ACAN	255.3498884	1346.526097	-2.398179172	9.33E-117	6.55E-115
ENSG00000205221	VIT	106.8707519	953.5388257	-3.160033879	4.65E-116	3.25E-114
ENSG00000166396	SERPINB7	1148.395059	206.0890907	2.478595977	1.77E-115	1.24E-113
ENSG00000116005	PCYOX1	2537.190293	5910.424485	-1.220079363	1.91E-115	1.33E-113
ENSG00000108518	PFN1	11016.40783	6015.797817	0.872669402	6.20E-115	4.30E-113
ENSG00000171451	DSEL	921.0707812	2630.429762	-1.513657519	1.85E-114	1.27E-112
ENSG00000162458	FBLIM1	1653.801532	405.0444171	2.030930828	2.45E-114	1.68E-112
ENSG00000113273	ARSB	3864.901448	1546.165071	1.320832203	9.05E-114	6.19E-112
ENSG00000147883	CDKN2B	1857.954681	418.9526016	2.153429658	9.59E-114	6.54E-112
ENSG00000122707	RECK	7314.39324	13943.34635	-0.930463808	2.56E-113	1.74E-111
ENSG00000009413	REV3L	1117.046476	3025.955239	-1.437867358	6.49E-113	4.40E-111
ENSG00000127946	HIP1	2572.17956	931.9611663	1.465396269	3.44E-112	2.32E-110
ENSG00000125148	MT2A	2754.039978	699.7145842	1.978729846	3.51E-112	2.36E-110
ENSG00000100106	TRIOBP	1713.26624	4015.414877	-1.229172442	9.31E-112	6.24E-110
ENSG00000132386	SERPINF1	5822.092611	2212.41596	1.395698044	1.94E-111	1.29E-109
ENSG00000123562	MORF4L2	5987.850071	2898.808598	1.046534375	2.22E-111	1.48E-109
ENSG00000105339	DENND3	395.2557543	1579.752909	-1.999150601	1.56E-110	1.04E-108
ENSG00000134516	DOCK2	1065.512817	187.7569405	2.501151584	1.95E-110	1.29E-108
ENSG00000076716	GPC4	711.1887168	69.13206394	3.36658568	2.05E-110	1.35E-108
ENSG00000158186	MRAS	2942.449561	918.1784019	1.677574781	2.67E-110	1.76E-108
ENSG00000117385	P3H1	3775.822981	1615.975543	1.223965124	2.76E-110	1.81E-108
ENSG00000107984	DKK1	642.6302751	2648.974212	-2.043673904	4.77E-110	3.12E-108
ENSG00000040275	SPDL1	1557.65439	364.9612379	2.089997077	1.10E-109	7.17E-108
ENSG00000134013	LOXL2	6154.189033	2599.84275	1.242445749	1.27E-109	8.25E-108
ENSG00000073712	FERMT2	5047.500401	2213.328438	1.190435675	1.50E-109	9.74E-108

ENSG00000154262	ABCA6	117.8899614	1025.081207	-3.123421324	2.03E-109	1.31E-107
ENSG00000164761	TNFRSF11B	2413.210242	6617.37366	-1.455323157	4.57E-109	2.95E-107
ENSG00000121957	GPSM2	374.4891368	1529.842631	-2.030264795	5.76E-109	3.70E-107
ENSG00000111424	VDR	2256.888891	740.0952629	1.609942654	1.16E-108	7.41E-107
ENSG00000114251	WNT5A	8505.945949	3258.447459	1.384632754	1.82E-108	1.16E-106
ENSG00000141696	P3H4	2272.694813	715.5877648	1.666861893	2.92E-108	1.86E-106
ENSG00000062716	VMP1	4214.348606	1630.955197	1.36985324	6.80E-108	4.32E-106
ENSG00000143933	CALM2	12736.5617	7448.419851	0.773915733	8.08E-108	5.12E-106
ENSG00000101236	RNF24	714.6654353	2394.108588	-1.744494123	2.00E-107	1.26E-105
ENSG00000096433	ITPR3	2680.672792	5821.871866	-1.1193303	7.27E-107	4.57E-105
ENSG00000213694	S1PR3	82.2018124	814.2715567	-3.308937184	7.31E-107	4.59E-105
ENSG00000244486	SCARF2	6738.446021	2344.582081	1.522071526	1.44E-106	9.01E-105
ENSG00000154258	ABCA9	74.41031386	783.4766982	-3.395157987	2.42E-106	1.51E-104
ENSG00000071967	CYBRD1	15410.21992	27927.97796	-0.857758631	4.37E-106	2.72E-104
ENSG00000136244	IL6	613.3423096	28.70340122	4.390227166	1.08E-105	6.70E-104
ENSG00000154122	ANKH	3384.415356	1182.491473	1.517320326	1.40E-105	8.68E-104
ENSG00000172935	MRGPRF	767.5172044	2575.237019	-1.745843211	2.35E-105	1.45E-103
ENSG00000178031	ADAMTSL1	795.6996709	2317.695347	-1.542409923	2.69E-105	1.66E-103
ENSG00000137331	IER3	996.4781831	176.7907243	2.490024688	1.11E-104	6.82E-103
ENSG00000182985	CADM1	1257.809367	273.9285242	2.200208281	1.41E-104	8.60E-103
ENSG00000111961	SASH1	1328.737954	3303.276664	-1.313620296	2.40E-104	1.46E-102
ENSG00000171793	CTPS1	1288.185574	311.1032599	2.050499629	4.38E-104	2.66E-102
ENSG00000160789	LMNA	18715.83194	34218.61026	-0.870577534	1.90E-103	1.15E-101
ENSG00000048740	CELF2	415.7196781	1671.635478	-2.007898171	8.25E-103	4.98E-101
ENSG00000111885	MAN1A1	2174.28399	4711.287447	-1.115824423	9.49E-103	5.72E-101
ENSG00000138080	EMILIN1	10318.32511	5045.318725	1.03261207	1.87E-102	1.12E-100
ENSG00000177706	FAM20C	1462.888207	3521.481712	-1.267346132	2.04E-102	1.22E-100
ENSG00000163661	PTX3	84.76208484	3906.084255	-5.52367524	3.25E-102	1.94E-100
ENSG00000115468	EFHD1	601.2734312	16.80128513	5.161414584	1.28E-101	7.63E-100
ENSG00000179051	RCC2	1930.492176	652.1325044	1.565404002	1.46E-101	8.66E-100
ENSG00000170390	DCLK2	1453.932018	198.9315548	2.863051539	2.64E-101	1.56E-99
ENSG00000108829	LRRC59	3528.998553	1554.169225	1.182476314	7.75E-101	4.58E-99
ENSG00000103888	CEMIP	6928.932095	12023.60365	-0.795203542	1.67E-100	9.82E-99
ENSG00000136603	SKIL	2190.135905	694.8807101	1.656159529	2.35E-100	1.38E-98
ENSG00000140945	CDH13	1247.95888	3417.649496	-1.45420064	8.71E-100	5.10E-98
ENSG00000149294	NCAM1	1025.571153	207.2708063	2.304828299	1.43E-99	8.34E-98
ENSG00000143631	FLG	951.0244251	163.04902	2.542300215	2.68E-99	1.56E-97
ENSG00000131435	PDLIM4	1423.998205	341.4646542	2.057482935	3.27E-99	1.90E-97
ENSG00000172986	GXYLT2	1313.919908	218.9165172	2.590964753	1.66E-98	9.63E-97
ENSG00000125753	VASP	2324.751534	789.1100669	1.559657838	2.69E-98	1.55E-96
ENSG00000109654	TRIM2	740.3376139	2180.12731	-1.557661764	4.62E-98	2.66E-96
ENSG00000151388	ADAMTS12	2262.155088	694.7341438	1.699854101	1.61E-97	9.25E-96
ENSG00000174437	ATP2A2	10114.5066	5713.294277	0.824431705	3.41E-97	1.95E-95
ENSG00000110436	SLC1A2	170.589641	1105.163727	-2.698469739	5.81E-97	3.32E-95
ENSG00000148700	ADD3	810.2325624	2554.371065	-1.656172511	8.16E-97	4.65E-95
ENSG00000116871	MAP7D1	5026.636385	2230.361282	1.172670994	1.53E-96	8.69E-95
ENSG00000106034	CPED1	601.8397319	2142.47219	-1.832080257	2.80E-96	1.58E-94
ENSG00000151718	WWC2	3475.458383	1175.164461	1.563836457	3.78E-96	2.14E-94
ENSG00000111913	RIPOR2	200.3206184	1174.238913	-2.548483823	6.23E-96	3.51E-94
ENSG00000134107	BHLHE40	3592.548408	1365.105567	1.39575649	8.89E-96	5.00E-94
ENSG00000143867	OSR1	41.27671952	787.7874198	-4.246543535	1.21E-95	6.78E-94
ENSG00000183010	PYCR1	1293.056698	318.8773822	2.019831903	1.22E-95	6.83E-94
ENSG00000141526	SLC16A3	4307.334686	1958.493101	1.13641901	1.56E-95	8.68E-94
ENSG00000162702	ZNF281	1770.961667	536.4556375	1.725111502	1.64E-95	9.10E-94
ENSG00000145390	USP53	685.2668119	2270.246054	-1.728104733	8.98E-95	4.98E-93
ENSG00000197894	ADH5	3614.116194	6836.460511	-0.919646242	9.88E-95	5.47E-93
ENSG00000168398	BDKRB2	23.39210893	639.3731795	-4.780294751	1.69E-94	9.31E-93
ENSG00000128342	LIF	929.774695	4.649031318	7.656817706	4.92E-94	2.71E-92
ENSG00000134243	SORT1	4121.229279	1918.909912	1.102625455	5.57E-93	3.06E-91
ENSG00000131711	MAP1B	3982.424646	7721.544845	-0.955224008	8.94E-92	4.90E-90
ENSG00000123989	CHPF	11488.42377	5319.328921	1.110569394	2.05E-91	1.12E-89
ENSG00000072682	P4HA2	2342.584118	881.6958892	1.411061489	1.98E-90	1.07E-88
ENSG00000148677	ANKRD1	473.9546552	22.27833415	4.414226624	4.68E-90	2.54E-88
ENSG00000141756	FKBP10	11106.12107	6128.370425	0.857328506	6.03E-90	3.26E-88
ENSG00000144476	ACKR3	1291.181044	350.0708897	1.88304479	5.88E-89	3.17E-87
ENSG00000162704	ARPC5	6132.765414	3159.00128	0.95746566	1.02E-88	5.47E-87

ENSG00000102760	RGCC	339.3209361	1353.253009	-1.995682776	2.44E-88	1.31E-86
ENSG00000013016	EHD3	1334.984419	3191.889564	-1.257352959	2.55E-88	1.37E-86
ENSG00000196562	SULF2	1561.778973	392.1878119	1.998213473	3.08E-88	1.65E-86
ENSG00000275342	PRAG1	511.0923976	40.59232482	3.664782873	5.43E-88	2.89E-86
ENSG00000112473	SLC39A7	4842.198003	2156.036895	1.167606707	5.66E-88	3.01E-86
ENSG00000136010	ALDH1L2	3078.266737	1181.985825	1.381793281	6.17E-88	3.27E-86
ENSG00000135480	KRT7	1455.95192	297.9870244	2.284282098	6.27E-88	3.32E-86
ENSG00000107562	CXCL12	1659.331058	3868.385402	-1.220718508	7.38E-88	3.89E-86
ENSG00000162576	MXRA8	21292.51829	10240.01465	1.055872137	7.51E-87	3.94E-85
ENSG00000162614	NEXN	2029.104166	679.6863789	1.580302992	9.08E-87	4.75E-85
ENSG00000152377	SPOCK1	7377.726116	3771.030274	0.96870199	1.04E-86	5.42E-85
ENSG00000115091	ACTR3	8077.700445	4291.213878	0.912847887	1.15E-86	6.01E-85
ENSG00000084636	COL16A1	9487.383332	5015.867532	0.918772172	1.94E-86	1.01E-84
ENSG00000069869	NEDD4	1619.024467	486.0111641	1.734386365	4.10E-86	2.12E-84
ENSG00000101608	MYL12A	8895.807703	4889.754335	0.863234826	4.50E-86	2.33E-84
ENSG00000107372	ZFAND5	5828.023916	3210.931213	0.860233942	3.92E-85	2.02E-83
ENSG00000180139	ACTA2-AS1	512.6530824	26.38644923	4.301984771	5.10E-85	2.62E-83
ENSG00000131473	ACLY	8317.351428	4851.50314	0.777335903	6.09E-85	3.12E-83
ENSG00000092820	EZR	4520.084867	2158.77974	1.065182941	2.18E-84	1.11E-82
ENSG00000136040	PLXNC1	311.2939484	1396.440255	-2.163811934	2.72E-84	1.39E-82
ENSG00000174945	AMZ1	537.7757268	39.28933078	3.76336846	5.84E-84	2.97E-82
ENSG00000130340	SNX9	3124.101352	6093.380081	-0.96413154	2.69E-82	1.36E-80
ENSG00000177606	JUN	1282.101918	3039.952907	-1.246467722	3.20E-82	1.62E-80
ENSG00000106105	GARS	4178.917252	2017.895991	1.050999938	3.83E-82	1.93E-80
ENSG00000167657	DAPK3	2790.045034	1145.744601	1.283098903	4.00E-82	2.01E-80
ENSG00000100626	GALNT16	158.7694139	855.2783259	-2.428251763	4.17E-82	2.10E-80
ENSG00000003436	TFPI	288.1955701	1295.068131	-2.169835421	4.64E-82	2.32E-80
ENSG00000183801	OLFML1	24.11187668	543.5398898	-4.498461612	7.66E-82	3.82E-80
ENSG00000010810	FYN	587.4859597	1696.408084	-1.529778709	2.05E-81	1.02E-79
ENSG00000102802	MEDAG	48.83118005	580.8986086	-3.5711387	7.85E-81	3.89E-79
ENSG00000277443	MARCKS	14210.9102	8088.717792	0.813245713	1.24E-80	6.15E-79
ENSG00000112658	SRF	2550.599878	989.4378122	1.364661014	1.86E-80	9.16E-79
ENSG00000019144	PHLDB1	3876.331611	1881.840073	1.041913767	2.31E-80	1.14E-78
ENSG00000174099	MSRB3	5682.31544	3156.150766	0.848070346	3.70E-80	1.82E-78
ENSG00000065413	ANKRD44	1093.780008	299.7273627	1.866648523	4.20E-80	2.06E-78
ENSG00000157404	KIT	48.00471156	634.8558249	-3.731778534	5.84E-80	2.86E-78
ENSG00000170962	PDGFD	181.4455579	906.560797	-2.320979633	1.28E-79	6.26E-78
ENSG00000198948	MFAP3L	622.6671275	82.69260309	2.90672344	1.37E-79	6.68E-78
ENSG00000280143	AP000892.3	438.0257931	22.58684245	4.282579894	1.73E-79	8.38E-78
ENSG00000167552	TUBA1A	11659.00171	6448.42519	0.854091376	1.90E-79	9.22E-78
ENSG00000143153	ATP1B1	1414.84186	372.7016324	1.925282299	2.53E-79	1.22E-77
ENSG00000228495	LINC01013	483.1903856	7.307133506	6.045813643	3.11E-79	1.50E-77
ENSG00000197622	CDC42SE1	2294.689043	916.2912484	1.32276046	3.33E-79	1.60E-77
ENSG00000073921	PICALM	6060.802621	3304.004142	0.875600701	3.78E-79	1.81E-77
ENSG00000111186	WNT5B	4369.502617	2150.389066	1.021877442	7.22E-79	3.45E-77
ENSG00000099250	NRP1	3334.642379	5996.8322	-0.846721524	2.01E-78	9.59E-77
ENSG00000155090	KLF10	1145.686531	318.553305	1.843793741	2.05E-78	9.76E-77
ENSG00000159640	ACE	198.7857167	989.7681691	-2.316816518	3.26E-78	1.55E-76
ENSG00000141574	SECTM1	21.27814058	535.8695666	-4.656664799	6.57E-78	3.11E-76
ENSG00000149212	SESN3	122.8159946	731.144618	-2.572859285	6.65E-78	3.14E-76
ENSG00000124104	SNX21	650.9969367	1761.960378	-1.43631037	8.23E-78	3.88E-76
ENSG00000136295	TTYH3	4924.179628	2440.45254	1.012928014	1.32E-77	6.20E-76
ENSG00000166033	HTRA1	22373.11256	13004.51223	0.782610585	2.29E-77	1.08E-75
ENSG00000181804	SLC9A9	50.81861393	573.1450097	-3.494584182	3.57E-77	1.67E-75
ENSG00000101199	ARFGAP1	2998.821015	1305.724119	1.198925416	7.95E-77	3.72E-75
ENSG00000162591	MEGF6	10989.8029	5503.556567	0.997087661	2.95E-76	1.38E-74
ENSG00000107611	CUBN	106.3028416	865.6331367	-3.02683427	3.20E-76	1.49E-74
ENSG00000113328	CCNG1	1237.18125	3613.212477	-1.545913172	4.49E-76	2.09E-74
ENSG00000176014	TUBB6	6328.134639	3178.703497	0.993015557	2.70E-75	1.25E-73
ENSG00000072210	ALDH3A2	372.8953955	1358.049931	-1.865725416	5.44E-75	2.52E-73
ENSG00000196843	ARID5A	441.6057688	1383.112386	-1.64661096	6.20E-75	2.85E-73
ENSG00000161203	AP2M1	7540.731925	4185.842595	0.849404321	7.51E-75	3.45E-73
ENSG00000163083	INHBB	140.0856735	802.4909571	-2.519621898	8.00E-75	3.66E-73
ENSG00000006451	RALA	2985.549555	1318.866751	1.178272921	8.90E-75	4.07E-73
ENSG00000176871	WSB2	2970.402348	1208.473197	1.298424955	2.34E-74	1.07E-72
ENSG00000010404	IDS	1430.687624	3179.125011	-1.151684794	5.03E-74	2.28E-72

ENSG00000162545	CAMK2N1	368.5792146	1372.011961	-1.895733252	6.67E-74	3.02E-72
ENSG00000079931	MOXD1	4623.488677	8007.400354	-0.792143734	7.55E-74	3.42E-72
ENSG00000196154	S100A4	6466.649582	12193.86584	-0.915085539	1.16E-73	5.25E-72
ENSG00000131016	AKAP12	2230.191802	4293.581224	-0.944971815	1.35E-73	6.09E-72
ENSG00000115107	STEAP3	124.6004176	778.5040317	-2.645684396	3.22E-73	1.45E-71
ENSG00000102316	MAGED2	4139.030782	2083.095696	0.990302175	4.76E-73	2.13E-71
ENSG00000116667	C1orf21	192.8542743	937.6366688	-2.281428713	6.11E-73	2.74E-71
ENSG00000082497	SERTAD4	660.7372463	87.60323373	2.916925163	8.44E-73	3.77E-71
ENSG00000183160	TMEM119	11662.11733	6824.798948	0.773452478	2.62E-72	1.17E-70
ENSG00000166794	PPIB	4394.106284	2172.325164	1.015671001	2.73E-72	1.21E-70
ENSG00000140564	FURIN	3454.990995	1674.995184	1.044090207	4.09E-72	1.82E-70
ENSG00000156011	PSD3	873.2444996	2302.339513	-1.399821326	4.20E-72	1.86E-70
ENSG00000174807	CD248	4263.651316	7938.93115	-0.897001083	1.19E-71	5.24E-70
ENSG00000222041	CYTOR	1343.168384	452.7707093	1.568857144	1.61E-71	7.11E-70
ENSG00000115641	FHL2	2235.467164	907.5617777	1.300907666	2.14E-71	9.38E-70
ENSG00000131378	RFTN1	1658.412491	609.6434989	1.445361781	2.46E-71	1.08E-69
ENSG00000188643	S100A16	3267.763171	1654.13938	0.982041368	3.03E-71	1.33E-69
ENSG00000150961	SEC24D	4611.296992	2486.108137	0.891681825	1.50E-70	6.55E-69
ENSG00000076706	MCAM	510.8873444	51.04918009	3.340314067	1.81E-70	7.87E-69
ENSG00000164828	SUN1	1739.5812	3434.785821	-0.981249755	2.38E-70	1.03E-68
ENSG00000160285	LSS	5644.122162	10377.66882	-0.878991704	3.10E-70	1.35E-68
ENSG00000184640	9-Sep	4264.323088	7601.907822	-0.834305779	3.95E-70	1.71E-68
ENSG00000120896	SORBS3	1233.766845	2721.217125	-1.141323794	6.20E-70	2.68E-68
ENSG00000154188	ANGPT1	989.7448295	273.3337857	1.85409905	7.16E-70	3.09E-68
ENSG00000124496	TRERF1	201.1709283	992.6456541	-2.30386722	9.98E-70	4.30E-68
ENSG00000153246	PLA2R1	1229.446014	2714.488086	-1.14331612	4.57E-69	1.96E-67
ENSG00000113070	HBEGF	384.7508493	11.01680241	5.090941921	6.44E-69	2.75E-67
ENSG00000249669	CARMN	1356.727114	414.922329	1.707103527	4.02E-68	1.71E-66
ENSG00000116157	GPX7	1076.883932	326.6977454	1.723031602	1.44E-67	6.10E-66
ENSG00000170873	MTSS1	63.47370816	539.8196189	-3.085866147	4.85E-67	2.05E-65
ENSG00000197971	MBP	457.941973	1360.745618	-1.570325718	1.05E-66	4.43E-65
ENSG00000116191	RALGPS2	709.4284411	1901.511932	-1.421029501	1.74E-66	7.36E-65
ENSG00000131018	SYNE1	6518.861349	3357.112241	0.956736062	1.80E-66	7.59E-65
ENSG00000112208	BAG2	589.4595327	1586.971453	-1.428755944	1.84E-66	7.72E-65
ENSG00000127334	DYRK2	1754.60195	678.229476	1.369753252	3.89E-66	1.63E-64
ENSG00000134324	LPIN1	1227.653935	2604.668757	-1.085368927	4.52E-66	1.89E-64
ENSG00000167191	GPRC5B	94.37182868	591.5816034	-2.647008544	1.29E-65	5.38E-64
ENSG00000198856	OSTC	3187.466414	1402.485466	1.185921525	1.34E-65	5.59E-64
ENSG00000269378	AC022149.1	3086.181891	1410.781962	1.130073673	2.72E-65	1.13E-63
ENSG00000142669	SH3BGRL3	2483.264889	4446.320373	-0.840242633	5.00E-65	2.07E-63
ENSG00000065150	IPO5	4734.774423	8216.414079	-0.794876498	5.67E-65	2.34E-63
ENSG00000143013	LMO4	1185.681417	362.9381086	1.704338417	6.33E-65	2.61E-63
ENSG00000138356	AOX1	772.9880956	1870.493977	-1.274527913	1.09E-64	4.51E-63
ENSG00000149968	MMP3	2352.73063	908.6122463	1.372864855	4.22E-64	1.73E-62
ENSG00000016391	CHDH	73.71318839	595.307927	-3.01035922	5.09E-64	2.09E-62
ENSG00000166949	SMAD3	447.5060933	1332.248908	-1.573045373	6.01E-64	2.46E-62
ENSG00000168297	PXK	970.0051917	2187.072148	-1.172739019	6.61E-64	2.70E-62
ENSG00000153885	KCTD15	688.2563628	132.615644	2.370627477	1.59E-63	6.46E-62
ENSG00000067798	NAV3	2649.871439	1151.586595	1.202682953	1.80E-63	7.31E-62
ENSG00000119408	NEK6	3121.257276	1518.495972	1.039754115	1.84E-63	7.48E-62
ENSG00000173641	HSPB7	2010.916354	818.7005017	1.298314129	3.67E-62	1.48E-60
ENSG00000100196	KDELR3	2596.474993	1240.493479	1.065286507	1.23E-61	4.98E-60
ENSG00000170624	SGCD	6873.946029	3303.796027	1.056349475	1.83E-61	7.37E-60
ENSG00000139567	ACVRL1	1020.522415	2300.770361	-1.172765338	2.14E-61	8.62E-60
ENSG00000204634	TBC1D8	74.43568726	510.94932	-2.778178879	3.75E-61	1.50E-59
ENSG00000095752	IL11	420.467308	38.27192209	3.458635713	4.34E-61	1.74E-59
ENSG00000185565	LSAMP	80.10315947	594.218514	-2.890378447	5.40E-61	2.16E-59
ENSG00000145675	PIK3R1	1031.433315	2342.127832	-1.183127893	5.71E-61	2.28E-59
ENSG00000033100	CHPF2	4198.856027	2286.595215	0.875962671	8.63E-61	3.44E-59
ENSG00000189058	APOD	60.65528748	507.799157	-3.061613281	1.37E-60	5.46E-59
ENSG00000132003	ZSWIM4	581.3037226	72.74207624	2.98475973	1.43E-60	5.67E-59
ENSG00000182158	CREB3L2	3266.481107	1716.038	0.929609135	1.49E-60	5.89E-59
ENSG00000166165	CKB	209.6586587	873.0278324	-2.059527198	2.16E-60	8.55E-59
ENSG00000162745	OLFML2B	1630.501446	632.2947649	1.366977496	2.89E-60	1.14E-58
ENSG00000185504	FAAP100	1486.695707	596.9001837	1.315970353	1.59E-59	6.23E-58
ENSG00000047932	GOPC	2002.426605	880.1827654	1.186439525	2.96E-59	1.16E-57

ENSG00000110237	ARHGEF17	5609.944896	3279.448636	0.774195392	4.62E-59	1.80E-57
ENSG00000149428	HYOU1	2183.511886	1012.376169	1.110076438	6.41E-59	2.49E-57
ENSG00000183671	GPR1	74.63331295	526.4811244	-2.820073371	7.58E-59	2.94E-57
ENSG00000108950	FAM20A	395.403322	1219.858382	-1.624894124	7.92E-59	3.07E-57
ENSG00000148120	C9orf3	1432.595394	538.9543992	1.410498234	1.07E-58	4.12E-57
ENSG00000149256	TENM4	933.7712103	286.3320018	1.703305913	1.28E-58	4.94E-57
ENSG00000104368	PLAT	742.5535865	182.7090334	2.024587698	1.35E-58	5.18E-57
ENSG00000022267	FHL1	472.9620351	1493.543741	-1.658287953	3.30E-58	1.27E-56
ENSG00000153993	SEMA3D	555.3612951	1586.392453	-1.513703075	6.51E-58	2.49E-56
ENSG00000155011	DKK2	385.342245	1265.803063	-1.715280883	1.05E-57	4.02E-56
ENSG00000149380	P4HA3	1057.612858	326.276388	1.699248113	1.17E-57	4.47E-56
ENSG00000130147	SH3BP4	2958.396618	1450.741713	1.027046688	1.52E-57	5.78E-56
ENSG00000129292	PHF20L1	2456.354859	1181.629716	1.056292724	2.04E-57	7.76E-56
ENSG00000166833	NAV2	400.7792065	1179.856497	-1.557815998	4.09E-57	1.55E-55
ENSG00000100439	ABHD4	2125.749863	988.1440839	1.104488005	6.92E-57	2.62E-55
ENSG00000160712	IL6R	62.23414655	480.7440183	-2.951089943	1.26E-56	4.74E-55
ENSG00000128923	MINDY2	2985.504476	1518.192001	0.975271534	1.48E-56	5.58E-55
ENSG00000163697	APBB2	2146.178406	959.3564034	1.1610655	2.18E-56	8.21E-55
ENSG00000167693	NXN	2448.868913	1149.484118	1.089702112	2.33E-56	8.73E-55
ENSG00000054965	FAM168A	2806.105067	1434.658379	0.96771253	2.42E-56	9.05E-55
ENSG00000163378	EOGT	1387.52614	558.6109618	1.312216787	2.56E-56	9.56E-55
ENSG00000134508	CABLES1	163.2417658	743.8611288	-2.188308926	2.76E-56	1.03E-54
ENSG00000108846	ABCC3	1100.141646	367.0855638	1.585324861	2.85E-56	1.06E-54
ENSG00000165434	PGM2L1	1542.293549	614.0147172	1.328281468	6.65E-56	2.47E-54
ENSG00000150636	CCDC102B	14.47244593	365.0468537	-4.661807609	6.88E-56	2.55E-54
ENSG00000123737	EXOSC9	1058.404967	345.4480173	1.617359352	1.28E-55	4.75E-54
ENSG00000181789	COPG1	4618.878912	2565.416019	0.847991422	2.85E-55	1.05E-53
ENSG00000119401	TRIM32	1278.899785	498.4163498	1.360246998	2.86E-55	1.06E-53
ENSG00000104408	EIF3E	1701.673887	3188.101612	-0.905260765	5.07E-55	1.87E-53
ENSG00000052795	FNIP2	1878.521351	855.4175487	1.135141005	1.33E-54	4.89E-53
ENSG00000161202	DVL3	3312.634826	1816.122566	0.867058338	2.56E-54	9.37E-53
ENSG00000146950	SHROOM2	351.5870844	30.14605959	3.52548137	3.31E-54	1.21E-52
ENSG00000139874	SSTR1	203.9382696	808.4094572	-1.987178452	4.16E-54	1.52E-52
ENSG00000136999	NOV	255.1665567	921.4243187	-1.853037645	6.14E-54	2.24E-52
ENSG00000127863	TNFRSF19	1035.285834	2351.349352	-1.183494995	1.20E-53	4.36E-52
ENSG00000151632	AKR1C2	159.4687094	740.9611161	-2.216793483	1.37E-53	4.96E-52
ENSG00000198561	CTNND1	5036.033658	2796.991048	0.848590456	1.90E-53	6.84E-52
ENSG00000138685	FGF2	3038.709245	1469.002288	1.048746847	3.75E-53	1.35E-51
ENSG00000198853	RUSC2	2404.166013	1160.786303	1.049766634	4.59E-53	1.65E-51
ENSG00000107560	RAB11FIP2	1060.10783	2361.329298	-1.154709327	5.22E-53	1.88E-51
ENSG00000107249	GLIS3	776.7024432	212.6516497	1.87224618	5.80E-53	2.08E-51
ENSG00000182054	IDH2	1955.204307	917.9660884	1.090189492	6.45E-53	2.31E-51
ENSG00000168906	MAT2A	2734.824132	4636.326106	-0.761350607	7.67E-53	2.74E-51
ENSG00000133466	C1QTNF6	763.7298506	215.4595765	1.827117119	7.81E-53	2.79E-51
ENSG00000117228	GBP1	1280.484235	400.6991541	1.678501357	1.18E-52	4.21E-51
ENSG00000140682	TGFB1I1	3379.42416	1565.184548	1.110629287	1.78E-52	6.32E-51
ENSG00000131634	TMEM204	1246.290863	417.4729788	1.57673443	3.40E-52	1.21E-50
ENSG00000143473	KCNH1	274.9035675	6.914310725	5.291249836	7.31E-52	2.59E-50
ENSG00000123700	KCNJ2	96.18001599	579.5028242	-2.590290397	7.69E-52	2.72E-50
ENSG00000070371	CLTCL1	991.4258473	305.3611493	1.698856424	1.11E-51	3.91E-50
ENSG00000184371	CSF1	2529.381235	4373.811327	-0.789912277	1.65E-51	5.80E-50
ENSG00000167110	GOLGA2	3944.247474	2328.410531	0.760924723	1.92E-51	6.74E-50
ENSG00000168014	C2CD3	1096.333054	374.710291	1.548513313	2.14E-51	7.50E-50
ENSG00000186815	TPCN1	771.2817848	1741.433018	-1.175838372	2.55E-51	8.90E-50
ENSG00000174738	NR1D2	1072.850581	2519.863948	-1.232172675	3.06E-51	1.07E-49
ENSG00000121898	CPXM2	2570.406905	4356.807039	-0.76098673	4.05E-51	1.41E-49
ENSG00000137831	UACA	3114.921599	1764.597741	0.820168149	4.21E-51	1.46E-49
ENSG00000134363	FST	4104.593624	2228.715599	0.881800724	4.56E-51	1.58E-49
ENSG00000141429	GALNT1	2413.15531	1026.546359	1.23517022	7.22E-51	2.50E-49
ENSG00000198431	TXNRD1	3059.660239	5428.844793	-0.827440467	9.92E-51	3.42E-49
ENSG00000168610	STAT3	3657.405466	2075.322876	0.818394633	1.11E-50	3.84E-49
ENSG00000109686	SH3D19	1741.297007	3320.003151	-0.930805494	1.12E-50	3.84E-49
ENSG00000100219	XBP1	2460.908538	1247.887088	0.978618392	1.37E-50	4.70E-49
ENSG00000183496	MEX3B	593.2839739	125.0810577	2.247779938	1.51E-50	5.18E-49
ENSG00000173674	EIF1AX	1212.761859	2500.334967	-1.043400202	1.56E-50	5.35E-49
ENSG00000105993	DNAJB6	2867.958562	1573.915917	0.864918232	1.61E-50	5.50E-49

ENSG00000189001	SBSN	18.79232896	332.1077799	-4.139969352	1.73E-50	5.91E-49
ENSG00000183876	ARSI	409.5091584	62.92600881	2.697024999	1.94E-50	6.62E-49
ENSG00000198832	SELENOM	2241.080983	1088.100221	1.040933554	2.01E-50	6.84E-49
ENSG00000012171	SEMA3B	158.6428794	656.2548868	-2.046930354	2.54E-50	8.63E-49
ENSG00000272841	AL139393.2	428.8880826	64.62032102	2.724053597	2.72E-50	9.23E-49
ENSG00000166780	C16orf45	590.7553572	1617.221479	-1.45291251	3.39E-50	1.15E-48
ENSG00000113716	HMGXB3	2631.542013	1353.404339	0.959981443	3.43E-50	1.16E-48
ENSG00000179604	CDC42EP4	119.2002313	565.1041417	-2.244768411	3.65E-50	1.23E-48
ENSG00000059573	ALDH18A1	2048.201077	1009.38039	1.020805383	3.95E-50	1.33E-48
ENSG00000144560	VGLL4	1589.649712	678.3055529	1.228010862	4.61E-50	1.55E-48
ENSG00000162630	B3GALT2	638.7766843	163.7311054	1.968436209	6.75E-50	2.26E-48
ENSG00000136240	KDELR2	7064.15838	4189.430225	0.754566261	7.50E-50	2.51E-48
ENSG00000104635	SLC39A14	2983.816974	1513.849067	0.979517139	7.60E-50	2.54E-48
ENSG00000165322	ARHGAP12	1032.354589	2065.650579	-1.000928452	8.55E-50	2.86E-48
ENSG00000107798	LIPA	1731.454206	3091.310776	-0.836370088	9.24E-50	3.08E-48
ENSG00000139132	FGD4	97.57186838	544.1683567	-2.480180801	9.82E-50	3.27E-48
ENSG00000175137	SH3BP5L	1308.318904	540.7339089	1.273343139	1.11E-49	3.70E-48
ENSG00000174564	IL20RB	41.65129391	378.8839047	-3.18447645	1.25E-49	4.13E-48
ENSG00000148175	STOM	1879.236879	3556.188528	-0.919631141	1.75E-49	5.78E-48
ENSG00000113580	NR3C1	1821.978055	3669.227671	-1.009998851	1.96E-49	6.47E-48
ENSG00000173848	NET1	781.7919206	226.8041214	1.787484807	2.05E-49	6.77E-48
ENSG00000182534	MXRA7	7821.246915	4200.770733	0.897056421	2.15E-49	7.07E-48
ENSG00000095303	PTGS1	263.0767793	871.9861998	-1.727382418	2.27E-49	7.46E-48
ENSG00000101224	CDC25B	870.3242295	1936.1579	-1.153954546	3.24E-49	1.06E-47
ENSG00000158470	B4GALT5	2745.100293	1448.765016	0.922399502	5.18E-49	1.69E-47
ENSG00000171223	JUNB	2122.282997	956.7473481	1.149424944	6.47E-49	2.11E-47
ENSG00000180340	FZD2	900.5060792	295.4391688	1.604859701	6.80E-49	2.22E-47
ENSG00000157020	SEC13	2939.345633	1560.066784	0.913942286	9.13E-49	2.97E-47
ENSG00000008517	IL32	325.1609833	26.32007933	3.643634848	1.09E-48	3.53E-47
ENSG00000242221	PSG2	40.5800273	380.8614264	-3.231873773	3.08E-48	9.98E-47
ENSG00000001617	SEMA3F	70.64711069	455.7139903	-2.686974581	3.31E-48	1.07E-46
ENSG00000157483	MYO1E	3656.258567	1955.938703	0.902680996	3.52E-48	1.14E-46
ENSG00000113758	DBN1	4354.514461	2490.722377	0.805644716	3.64E-48	1.17E-46
ENSG00000169641	LUZP1	2778.801468	1534.027085	0.857250667	6.63E-48	2.12E-46
ENSG00000010278	CD9	474.6199236	1298.125503	-1.45183947	7.04E-48	2.25E-46
ENSG00000143819	EPHX1	838.7055483	1804.433386	-1.104739598	1.01E-47	3.22E-46
ENSG00000013293	SLC7A14	2.866486713	766.1012218	-8.093464889	1.54E-47	4.90E-46
ENSG00000124766	SOX4	1247.992396	455.2305614	1.453608484	2.27E-47	7.20E-46
ENSG00000127314	RAP1B	4552.321258	2646.746555	0.782331889	2.76E-47	8.75E-46
ENSG00000051108	HERPUD1	2380.319585	1255.550284	0.922624012	3.31E-47	1.05E-45
ENSG00000001461	NIPAL3	1223.244938	2466.915973	-1.011408832	4.84E-47	1.53E-45
ENSG00000197361	FBXL22	239.227314	9.340434773	4.663649252	5.88E-47	1.86E-45
ENSG00000196975	ANXA4	1865.719223	3326.513333	-0.833973342	6.80E-47	2.14E-45
ENSG00000068438	FTSJ1	1286.816364	459.572397	1.481592029	7.02E-47	2.21E-45
ENSG00000198743	SLC5A3	5821.384366	3419.719501	0.767803905	7.54E-47	2.37E-45
ENSG00000064932	SBNO2	1949.163998	829.4639421	1.23301259	1.07E-46	3.35E-45
ENSG00000104361	NIPAL2	926.9505832	1917.486057	-1.048992298	1.13E-46	3.55E-45
ENSG00000146374	RSPO3	40.391566	355.8326645	-3.136436644	1.19E-46	3.73E-45
ENSG00000166016	ABTB2	728.6909155	216.1823142	1.749219804	1.22E-46	3.80E-45
ENSG00000233901	LINC01503	396.9035581	54.5612554	2.858186626	2.73E-46	8.51E-45
ENSG00000170681	CAVIN4	355.974024	47.47226251	2.895943547	3.48E-46	1.08E-44
ENSG00000145349	CAMK2D	1137.326661	2222.737188	-0.966256378	4.58E-46	1.42E-44
ENSG00000093167	LRRFIP2	2070.830663	3650.912129	-0.818093788	4.83E-46	1.50E-44
ENSG00000154277	UCHL1	1430.551163	631.505012	1.178684259	5.24E-46	1.62E-44
ENSG00000123146	ADGRE5	953.0240368	1985.101344	-1.058700573	9.55E-46	2.95E-44
ENSG00000118689	FOXO3	2249.521692	1037.609448	1.115632901	9.56E-46	2.95E-44
ENSG00000072163	LIMS2	1458.174171	615.3244323	1.242656115	1.21E-45	3.73E-44
ENSG00000072952	MRVI1	1325.568571	482.5781864	1.458621477	1.51E-45	4.63E-44
ENSG00000099377	HSD3B7	340.9212616	930.8832257	-1.449278069	2.31E-45	7.10E-44
ENSG00000204941	PSG5	3808.424244	6741.049599	-0.82431133	2.51E-45	7.69E-44
ENSG00000106819	ASPN	271.1175943	23.51409847	3.525024933	3.00E-45	9.18E-44
ENSG00000254851	AP005018.2	350.1032354	40.13940011	3.112798665	3.63E-45	1.11E-43
ENSG00000230838	LINC01614	234.5415606	5.586447596	5.369798234	3.91E-45	1.19E-43
ENSG00000124813	RUNX2	641.3432351	164.5913465	1.958447088	4.22E-45	1.29E-43
ENSG00000172260	NEGR1	2067.393071	1041.7623	0.989756682	4.56E-45	1.39E-43
ENSG00000231991	ANXA2P2	3047.039558	1750.379986	0.799212694	4.93E-45	1.50E-43

ENSG00000134882	UBAC2	2230.268278	1152.231275	0.952193187	6.12E-45	1.86E-43
ENSG00000128283	CDC42EP1	2134.779522	1039.904791	1.03716832	7.10E-45	2.15E-43
ENSG00000072840	EVC	2322.308198	4033.816644	-0.797146046	8.84E-45	2.67E-43
ENSG00000135424	ITGA7	114.3781677	523.2310777	-2.193411874	1.14E-44	3.44E-43
ENSG00000171067	C11orf24	1699.935363	768.0300914	1.146156962	1.40E-44	4.21E-43
ENSG00000131981	LGALS3	2398.743625	4281.002239	-0.835799434	1.66E-44	5.01E-43
ENSG00000136044	APPL2	1225.91357	2348.861418	-0.9388223	1.76E-44	5.29E-43
ENSG00000184840	TMED9	3313.514924	1954.289604	0.761860588	2.90E-44	8.71E-43
ENSG00000161249	DMKN	117.166186	547.2824034	-2.223455866	3.21E-44	9.63E-43
ENSG00000069188	SDK2	294.5841137	30.8357143	3.268499956	5.01E-44	1.50E-42
ENSG00000184347	SLIT3	9526.342964	16103.18319	-0.757320202	6.93E-44	2.07E-42
ENSG00000081923	ATP8B1	2399.957145	4052.252784	-0.756349879	9.07E-44	2.70E-42
ENSG00000149090	PAMR1	533.7980505	1321.531992	-1.309318688	1.20E-43	3.57E-42
ENSG00000150764	DIXDC1	2126.851173	1129.315174	0.912308127	1.46E-43	4.35E-42
ENSG00000101194	SLC17A9	1091.842937	418.68625	1.382153579	2.07E-43	6.14E-42
ENSG00000182541	LIMK2	1024.14096	364.0554004	1.488860306	2.16E-43	6.39E-42
ENSG00000082512	TRAF5	1557.248856	691.0197115	1.17030115	2.17E-43	6.42E-42
ENSG00000011426	ANLN	284.2647104	891.4194045	-1.648017287	2.31E-43	6.80E-42
ENSG00000187189	TSPYL4	1336.557278	599.279184	1.157958955	3.30E-43	9.70E-42
ENSG00000132294	EFR3A	4288.640139	2421.39179	0.824942712	3.84E-43	1.13E-41
ENSG00000043143	JADE2	463.3268439	1171.312763	-1.338210996	4.02E-43	1.18E-41
ENSG00000112078	KCTD20	3105.248322	1797.841002	0.78849829	5.68E-43	1.66E-41
ENSG00000119673	ACOT2	813.2480745	244.7151403	1.727103446	6.26E-43	1.83E-41
ENSG00000127124	HIVEP3	327.1192409	43.32220531	2.92471856	7.18E-43	2.09E-41
ENSG00000058091	CDK14	1715.116393	816.2724076	1.071583947	7.34E-43	2.14E-41
ENSG00000135111	TBX3	1949.824132	1033.306534	0.91629	1.06E-42	3.08E-41
ENSG00000117533	VAMP4	448.5582145	1163.993919	-1.375598272	1.10E-42	3.19E-41
ENSG00000144645	OSBPL10	396.2900846	73.1279745	2.433843491	1.12E-42	3.23E-41
ENSG00000112186	CAP2	953.1839835	344.4085565	1.466564528	1.39E-42	4.04E-41
ENSG00000175602	CCDC85B	811.6113736	1764.895683	-1.120248795	1.40E-42	4.06E-41
ENSG00000107263	RAPGEF1	1583.968023	2781.546184	-0.812117124	1.67E-42	4.81E-41
ENSG00000168734	PKIG	675.2466824	1548.525505	-1.19839466	2.17E-42	6.25E-41
ENSG00000176915	ANKLE2	3211.75277	1781.947976	0.848602582	2.78E-42	8.01E-41
ENSG00000076356	PLXNA2	288.3434703	846.3391663	-1.55454272	4.16E-42	1.19E-40
ENSG00000101955	SRPX	1228.822406	2372.355842	-0.948319069	4.68E-42	1.34E-40
ENSG00000103647	CORO2B	868.3796782	299.9292899	1.532385752	5.47E-42	1.56E-40
ENSG00000159128	IFNGR2	1377.010976	593.9020066	1.215121003	6.17E-42	1.76E-40
ENSG00000128284	APOL3	31.09681791	315.6733967	-3.340287405	8.23E-42	2.34E-40
ENSG00000134769	DTNA	23.51591227	290.4301716	-3.621676337	1.50E-41	4.26E-40
ENSG00000166482	MFAP4	5998.224904	3433.284885	0.804634106	1.86E-41	5.29E-40
ENSG00000164112	TMEM155	322.5585171	47.50231348	2.760979399	1.88E-41	5.31E-40
ENSG00000185950	IRS2	3058.778176	1750.663851	0.803974803	2.25E-41	6.35E-40
ENSG00000101367	MAPRE1	2419.20083	1309.811234	0.885863234	3.58E-41	1.01E-39
ENSG00000196730	DAPK1	414.5718344	1060.086451	-1.354981674	3.59E-41	1.01E-39
ENSG00000144802	NFKBIZ	817.6745808	249.9803173	1.712712825	3.83E-41	1.08E-39
ENSG00000142149	HUNK	824.4786935	283.1961176	1.539261978	3.87E-41	1.09E-39
ENSG00000155511	GRIA1	6.092410506	299.8830257	-5.638874232	4.11E-41	1.15E-39
ENSG00000185551	NR2F2	549.2632946	1259.836305	-1.198401379	5.07E-41	1.42E-39
ENSG00000115318	LOXL3	1173.956533	462.5422554	1.343136469	5.66E-41	1.58E-39
ENSG00000188906	LRRK2	255.5227017	759.7124624	-1.571144434	6.72E-41	1.87E-39
ENSG00000176046	NUPR1	5434.681655	3220.578029	0.754192199	9.84E-41	2.74E-39
ENSG00000150347	ARID5B	1537.4273	2820.898459	-0.875106759	1.18E-40	3.28E-39
ENSG00000251493	FOXD1	1416.705033	662.7279375	1.094858901	1.22E-40	3.37E-39
ENSG00000151090	THRB	822.9797307	1969.235327	-1.257807641	1.50E-40	4.15E-39
ENSG00000136802	LRRC8A	2960.976879	1655.263823	0.839252112	1.78E-40	4.90E-39
ENSG00000117724	CENPF	523.1948971	1218.866499	-1.220332672	2.77E-40	7.64E-39
ENSG00000144746	ARL6IP5	2303.474207	3927.831407	-0.769377362	2.90E-40	7.97E-39
ENSG00000007866	TEAD3	1407.659064	630.5183341	1.15687443	3.01E-40	8.27E-39
ENSG00000162337	LRP5	2598.794556	1451.235267	0.840768838	3.04E-40	8.35E-39
ENSG00000164576	SAP30L	610.2698525	1373.625158	-1.171091512	3.34E-40	9.14E-39
ENSG00000181019	NQO1	1275.972033	2593.804418	-1.024083542	3.82E-40	1.04E-38
ENSG00000138413	IDH1	1364.512892	2502.429049	-0.875328627	4.76E-40	1.30E-38
ENSG00000117020	AKT3	2617.870046	1444.633321	0.857778914	6.23E-40	1.70E-38
ENSG00000166473	PKD1L2	16.38498576	270.0474088	-4.03956166	9.32E-40	2.53E-38
ENSG00000240184	PCDHGC3	1175.508537	2210.237086	-0.910624183	1.18E-39	3.21E-38
ENSG00000074410	CA12	42.18170677	344.1214438	-3.022105898	1.75E-39	4.73E-38

ENSG00000162493	PDPN	804.1491145	218.4080161	1.887038805	2.04E-39	5.51E-38
ENSG00000127990	SGCE	893.9288192	1756.923275	-0.975183397	2.07E-39	5.60E-38
ENSG00000123080	CDKN2C	198.8006492	656.3860985	-1.722521313	2.55E-39	6.87E-38
ENSG00000135636	DYSF	214.4217318	7.569592403	4.822883343	2.68E-39	7.23E-38
ENSG00000121039	RDH10	729.8516761	245.2510784	1.573242409	2.73E-39	7.33E-38
ENSG00000117318	ID3	2368.974761	48.62845271	5.61194331	3.14E-39	8.42E-38
ENSG00000283154	IQCJ-SCHIP1	506.4420757	122.8333045	2.038381766	3.39E-39	9.09E-38
ENSG00000025423	HSD17B6	201.8505682	7.33934539	4.747143372	3.71E-39	9.93E-38
ENSG00000125968	ID1	213.5281797	14.91088393	3.857001438	4.77E-39	1.27E-37
ENSG00000124006	OBSL1	2936.867609	1698.391432	0.790180254	7.85E-39	2.09E-37
ENSG00000104213	PDGFRL	458.6235546	1185.684439	-1.369318209	8.61E-39	2.29E-37
ENSG00000143878	RHOB	2170.516167	1178.613856	0.880754098	1.10E-38	2.92E-37
ENSG00000165655	ZNF503	996.0577631	397.8099329	1.321728239	1.21E-38	3.21E-37
ENSG00000176720	BOK	1300.53698	605.5667474	1.102046046	1.31E-38	3.47E-37
ENSG00000147872	PLIN2	784.0356918	1688.619624	-1.106520928	1.51E-38	3.99E-37
ENSG00000102468	HTR2A	221.1146753	4.973753064	5.562283867	1.59E-38	4.18E-37
ENSG00000196776	CD47	1569.56475	3001.298888	-0.93464793	2.21E-38	5.82E-37
ENSG00000122870	BICC1	2476.15257	1420.6181	0.802142322	4.06E-38	1.07E-36
ENSG00000119812	FAM98A	2079.69192	1101.486473	0.918600422	4.26E-38	1.12E-36
ENSG00000087053	MTMR2	1274.949804	560.285192	1.185812319	7.78E-38	2.03E-36
ENSG00000111728	ST8SIA1	5.550995733	269.0689333	-5.586146355	9.08E-38	2.37E-36
ENSG00000276600	RAB7B	70.99128268	394.2379944	-2.474161631	1.06E-37	2.77E-36
ENSG00000143772	ITPKB	174.3779122	651.5937371	-1.900203674	1.48E-37	3.86E-36
ENSG00000106123	EPHB6	37.03714244	320.3849061	-3.116406073	1.56E-37	4.06E-36
ENSG00000206560	ANKRD28	1443.078369	2571.446068	-0.832802544	1.65E-37	4.27E-36
ENSG00000151748	SAV1	647.0839183	1451.305518	-1.165650099	2.03E-37	5.25E-36
ENSG00000148634	HERC4	1490.99795	2623.251748	-0.815378633	2.55E-37	6.60E-36
ENSG00000050820	BCAR1	2873.723523	1569.733253	0.871288075	2.57E-37	6.64E-36
ENSG00000164056	SPRY1	25.69180746	270.8564189	-3.400399401	2.77E-37	7.16E-36
ENSG00000175745	NR2F1	592.1443743	171.7799958	1.782417862	3.17E-37	8.18E-36
ENSG00000101665	SMAD7	1614.383185	769.5914492	1.069313224	3.30E-37	8.50E-36
ENSG00000166986	MARS	1757.743665	939.8358048	0.904002905	3.77E-37	9.70E-36
ENSG00000126218	F10	335.97736	912.5303601	-1.442138562	3.88E-37	9.97E-36
ENSG00000164050	PLXNB1	678.2800095	1448.01182	-1.095154718	4.55E-37	1.16E-35
ENSG00000174695	TMEM167A	2898.477405	1530.590896	0.922174957	4.78E-37	1.22E-35
ENSG00000187634	SAMD11	238.1621481	16.39483984	3.880284408	4.95E-37	1.27E-35
ENSG00000112837	TBX18	736.9736933	1533.297638	-1.056718773	5.32E-37	1.36E-35
ENSG00000158122	PRXL2C	631.3642785	1379.193616	-1.126598822	6.27E-37	1.60E-35
ENSG00000168994	PXDC1	2034.010597	1124.978985	0.854142417	6.54E-37	1.66E-35
ENSG00000165633	VSTM4	881.1849198	1743.727025	-0.983674611	7.06E-37	1.80E-35
ENSG00000124702	KLHDC3	1667.810953	828.5139777	1.008673445	8.28E-37	2.10E-35
ENSG00000054392	HHAT	367.6501808	69.45873183	2.413340644	8.35E-37	2.12E-35
ENSG00000125730	C3	227.6638308	24.65192581	3.216146376	8.53E-37	2.16E-35
ENSG00000205978	NYNRIN	716.9453772	1496.989818	-1.062006474	1.02E-36	2.57E-35
ENSG00000064309	CDON	71.56723525	385.765132	-2.432573997	1.11E-36	2.81E-35
ENSG00000100596	SPTLC2	2543.324008	1498.660812	0.762986588	1.24E-36	3.12E-35
ENSG00000116711	PLA2G4A	20.14818893	250.6144067	-3.64275217	2.00E-36	5.02E-35
ENSG00000181458	TMEM45A	908.1214185	358.8379931	1.338659766	2.07E-36	5.19E-35
ENSG00000246763	RGMB-AS1	49.68485946	341.1630472	-2.779993103	3.39E-36	8.47E-35
ENSG00000100342	APOL1	101.5900554	483.6284877	-2.248563135	3.60E-36	8.99E-35
ENSG00000177508	IRX3	582.7696529	153.8019854	1.921647198	5.82E-36	1.45E-34
ENSG00000108691	CCL2	243.0703899	28.30711618	3.092014346	6.61E-36	1.64E-34
ENSG00000072422	RHOBTB1	1674.780653	828.0552038	1.017661231	8.18E-36	2.03E-34
ENSG00000065320	NTN1	426.1578321	1091.012557	-1.356452139	8.56E-36	2.12E-34
ENSG00000148158	SNX30	1724.776195	860.2434114	1.003356464	9.89E-36	2.45E-34
ENSG00000114861	FOXP1	414.0980642	102.5969158	2.011486394	1.17E-35	2.89E-34
ENSG00000127954	STEAP4	9.962314407	221.3430708	-4.467449455	1.19E-35	2.95E-34
ENSG00000246859	STARD4-AS1	507.5142359	132.0591591	1.939392909	1.28E-35	3.16E-34
ENSG00000143669	LYST	962.4545069	1901.505866	-0.982935934	2.70E-35	6.65E-34
ENSG00000170558	CDH2	828.0196633	330.7866263	1.325672013	3.08E-35	7.59E-34
ENSG00000109089	CDR2L	1248.716226	602.1381268	1.051825074	3.20E-35	7.87E-34
ENSG00000169826	CSGALNACT2	1950.405598	1054.069549	0.888639413	3.34E-35	8.21E-34
ENSG00000114450	GNB4	2546.009766	1427.965433	0.835613417	3.69E-35	9.05E-34
ENSG00000107745	MICU1	2110.782563	1190.91289	0.825817467	4.47E-35	1.10E-33
ENSG00000133083	DCLK1	17.78566996	244.6556721	-3.789463179	4.96E-35	1.21E-33
ENSG00000246090	AP002026.1	61.114431	348.6442413	-2.511549295	6.55E-35	1.60E-33

ENSG00000170370	EMX2	417.3154113	1092.851094	-1.389204934	7.41E-35	1.81E-33
ENSG00000144152	FBLN7	140.6075871	552.1519513	-1.974272114	1.06E-34	2.57E-33
ENSG00000206527	HACD2	1652.356631	792.2913326	1.062333287	1.17E-34	2.83E-33
ENSG00000152784	PRDM8	126.9168358	489.6957604	-1.949184989	1.20E-34	2.92E-33
ENSG00000078804	TP53INP2	1077.443521	491.7856901	1.132705876	1.45E-34	3.53E-33
ENSG00000058272	PPP1R12A	2464.122744	1420.618303	0.794950923	1.74E-34	4.21E-33
ENSG00000221869	CEBPD	1562.815651	700.7413281	1.156226643	2.06E-34	4.99E-33
ENSG00000203805	PLPP4	312.7873447	1.72068591	7.584011792	2.56E-34	6.19E-33
ENSG00000197594	ENPP1	1120.605748	472.1931097	1.24895359	3.74E-34	9.00E-33
ENSG00000198873	GRK5	262.6521232	779.2812596	-1.568390766	4.12E-34	9.92E-33
ENSG00000164048	ZNF589	87.74811172	383.9065127	-2.129706429	4.30E-34	1.03E-32
ENSG00000186918	ZNF395	187.0075555	599.3072888	-1.680843577	4.34E-34	1.04E-32
ENSG00000189043	NDUFA4	2005.209497	1070.556296	0.905411337	5.01E-34	1.20E-32
ENSG00000137267	TUBB2A	1509.448099	769.9780038	0.971666901	5.74E-34	1.38E-32
ENSG00000189339	SLC35E2B	895.800798	1666.098124	-0.894787199	6.08E-34	1.45E-32
ENSG00000196141	SPATS2L	2459.811236	1452.912925	0.760324376	6.47E-34	1.54E-32
ENSG00000162520	SYNC	1785.544106	947.9515301	0.91263032	6.87E-34	1.64E-32
ENSG00000163815	CLEC3B	6.351580127	250.4192123	-5.292334893	7.69E-34	1.83E-32
ENSG00000183250	LINC01547	356.4654074	57.04683635	2.627800066	7.88E-34	1.87E-32
ENSG00000197467	COL13A1	372.3502046	1113.372195	-1.582286123	1.00E-33	2.37E-32
ENSG00000137675	MMP27	14.75430817	222.1890331	-3.907636407	1.24E-33	2.94E-32
ENSG00000143382	ADAMTSL4	469.9493347	1121.646819	-1.255751268	1.56E-33	3.68E-32
ENSG00000160255	ITGB2	309.3374005	53.35705816	2.525988126	1.95E-33	4.60E-32
ENSG00000110756	HPS5	1274.887545	581.2797541	1.130609505	2.75E-33	6.49E-32
ENSG00000143537	ADAM15	1064.144985	1945.268721	-0.870097992	3.06E-33	7.22E-32
ENSG00000140859	KIFC3	1740.661987	953.2547448	0.867606619	3.18E-33	7.48E-32
ENSG00000138162	TACC2	105.8904876	432.8047786	-2.029721484	3.32E-33	7.82E-32
ENSG00000233098	CCDC144NL-AS1	174.3747207	11.49156374	3.911402868	4.74E-33	1.11E-31
ENSG00000174791	RIN1	116.929148	436.6740143	-1.901413456	5.07E-33	1.19E-31
ENSG00000129048	ACKR4	968.6991579	1813.345335	-0.90475522	5.50E-33	1.29E-31
ENSG00000148841	ITPRIP	886.861889	379.0255032	1.225997422	1.02E-32	2.40E-31
ENSG00000144730	IL17RD	138.9123019	494.8115312	-1.830854058	1.04E-32	2.43E-31
ENSG00000159363	ATP13A2	1000.578259	449.5647896	1.153300772	1.82E-32	4.25E-31
ENSG00000104043	ATP8B4	40.11942916	267.6210698	-2.738543539	1.96E-32	4.58E-31
ENSG00000006534	ALDH3B1	746.3785384	1474.847529	-0.982717395	2.14E-32	4.99E-31
ENSG00000134668	SPOCD1	718.9919061	213.2286579	1.750814961	2.16E-32	5.03E-31
ENSG00000168701	TMEM208	688.8530663	257.6203865	1.418974363	3.09E-32	7.17E-31
ENSG00000086991	NOX4	174.9451336	3.126810327	5.758145111	3.48E-32	8.07E-31
ENSG00000222009	BTBD19	1101.353869	532.1834418	1.04882935	3.69E-32	8.53E-31
ENSG00000196781	TLE1	171.8921509	565.2377411	-1.717046709	5.93E-32	1.37E-30
ENSG00000150051	MKX	2644.029698	1499.455555	0.818951016	6.16E-32	1.42E-30
ENSG00000174851	YIF1A	1495.011212	764.8025268	0.967766715	6.48E-32	1.49E-30
ENSG00000145246	ATP10D	2200.537173	1247.754087	0.818561978	6.59E-32	1.51E-30
ENSG00000126016	AMOT	141.530238	498.1153996	-1.814345388	7.24E-32	1.66E-30
ENSG00000178860	MSC	703.7697267	213.8102388	1.717216105	7.31E-32	1.68E-30
ENSG00000196616	ADH1B	0.412532103	6400.608457	-13.85932606	7.33E-32	1.68E-30
ENSG00000176971	FIBIN	1361.127304	681.1753127	0.998184636	7.47E-32	1.71E-30
ENSG00000166002	SMCO4	377.2417503	84.30454696	2.158156648	7.49E-32	1.71E-30
ENSG00000151929	BAG3	1693.023734	899.8910008	0.912346223	7.79E-32	1.78E-30
ENSG00000169271	HSPB3	131.4122129	475.0170351	-1.852712039	7.95E-32	1.81E-30
ENSG00000018408	WWTR1	2634.77056	1460.703656	0.852476493	8.27E-32	1.88E-30
ENSG00000198108	CHSY3	426.4437256	102.0681057	2.05633155	9.69E-32	2.20E-30
ENSG00000091409	ITGA6	322.9438886	789.5030925	-1.289260678	1.10E-31	2.50E-30
ENSG00000242265	PEG10	79.85601314	395.8931587	-2.306286187	1.66E-31	3.75E-30
ENSG00000139083	ETV6	814.9778708	340.9051488	1.257620384	1.78E-31	4.02E-30
ENSG00000185742	C11orf87	60.47782809	311.5486266	-2.366055631	1.80E-31	4.06E-30
ENSG00000160161	CILP2	183.6463429	3.293063361	5.817681583	2.06E-31	4.65E-30
ENSG00000187479	C11orf96	674.5362575	206.5954192	1.707430279	3.71E-31	8.34E-30
ENSG00000117139	KDM5B	1911.825693	1081.034139	0.821689748	3.78E-31	8.49E-30
ENSG00000124343	XG	1018.347588	2027.065206	-0.993306202	3.83E-31	8.60E-30
ENSG00000184227	ACOT1	844.6525884	357.1324343	1.242108172	3.91E-31	8.76E-30
ENSG00000154930	ACSS1	56.52062394	314.2707347	-2.475892045	4.70E-31	1.05E-29
ENSG00000146143	PRIM2	159.6162771	512.5872078	-1.681895326	4.96E-31	1.11E-29
ENSG00000095383	TBC1D2	1229.49998	599.2545414	1.037421149	5.94E-31	1.32E-29
ENSG00000091436	MAP3K20	1404.118346	2552.374061	-0.861833961	6.96E-31	1.55E-29
ENSG00000196460	RFX8	185.0011815	597.5256405	-1.693090202	8.56E-31	1.90E-29

ENSG00000130956	HABP4	842.1710128	1546.090045	-0.876964791	8.76E-31	1.94E-29
ENSG00000225968	ELFN1	69.45975051	407.375649	-2.551663629	9.00E-31	1.99E-29
ENSG00000007237	GAS7	1381.477328	732.8155773	0.914248092	1.13E-30	2.50E-29
ENSG00000122644	ARL4A	582.8021716	204.8652061	1.509228949	2.10E-30	4.62E-29
ENSG00000106636	YKT6	3436.546923	2017.172168	0.769810683	2.23E-30	4.89E-29
ENSG00000166224	SGPL1	908.8650547	421.0944412	1.109276211	2.27E-30	4.97E-29
ENSG00000148773	MKI67	394.1554392	1008.94172	-1.357081071	2.34E-30	5.12E-29
ENSG00000116741	RGS2	64.00271674	367.3652986	-2.520753569	2.65E-30	5.79E-29
ENSG00000133812	SBF2	1110.915674	1993.757986	-0.84366613	2.76E-30	6.03E-29
ENSG00000197555	SIPA1L1	1919.52116	1127.041895	0.767768887	3.04E-30	6.64E-29
ENSG00000164603	BMT2	788.200143	313.763535	1.330270925	3.32E-30	7.22E-29
ENSG00000082397	EPB41L3	432.2380145	962.9596716	-1.155121272	3.88E-30	8.43E-29
ENSG00000056972	TRAF3IP2	333.2893807	838.7354043	-1.331484638	3.96E-30	8.60E-29
ENSG00000163820	FYCO1	1058.572341	1868.344352	-0.819541521	4.66E-30	1.01E-28
ENSG00000164330	EBF1	113.7336769	435.1087766	-1.937628754	4.72E-30	1.02E-28
ENSG00000108001	EBF3	152.2407573	519.6294945	-1.772769041	5.43E-30	1.18E-28
ENSG00000060982	BCAT1	1524.854809	773.1473665	0.982261677	5.57E-30	1.20E-28
ENSG00000214595	EML6	26.05303173	221.8448623	-3.090069136	7.85E-30	1.69E-28
ENSG00000109066	TMEM104	1659.993954	925.9991842	0.84340152	7.90E-30	1.70E-28
ENSG00000108984	MAP2K6	99.78247947	448.8504811	-2.169166429	8.37E-30	1.80E-28
ENSG00000138061	CYP1B1	1012.664112	2006.063958	-0.985472084	1.18E-29	2.53E-28
ENSG00000246430	LINC00968	47.40785355	279.7767974	-2.564620125	1.18E-29	2.54E-28
ENSG00000118985	ELL2	2511.170445	1434.43551	0.807351382	1.46E-29	3.13E-28
ENSG00000197380	DACT3	709.909921	271.6768788	1.38268099	1.56E-29	3.34E-28
ENSG00000019991	HGF	12.36406759	183.9636019	-3.8910335	1.68E-29	3.59E-28
ENSG00000189337	KAZN	272.6596951	700.546075	-1.363075114	1.90E-29	4.07E-28
ENSG00000007944	MYLIP	57.2394981	307.839161	-2.42679763	2.19E-29	4.66E-28
ENSG00000178573	MAF	630.6765459	1289.58583	-1.031230108	2.33E-29	4.95E-28
ENSG00000127955	GNAI1	334.0651527	808.6133195	-1.273984186	2.86E-29	6.07E-28
ENSG00000189067	LITAF	479.1812623	1006.344784	-1.069750107	3.06E-29	6.49E-28
ENSG00000171408	PDE7B	59.54925462	311.2288835	-2.385235201	3.09E-29	6.56E-28
ENSG00000166483	WEE1	423.8228299	923.1432852	-1.122804009	3.43E-29	7.26E-28
ENSG00000177374	HIC1	1424.103299	714.2082291	0.995933708	4.21E-29	8.89E-28
ENSG00000240849	TMEM189	1019.076346	443.3161244	1.202824843	4.58E-29	9.65E-28
ENSG00000170571	EMB	463.30443	148.0315435	1.64889134	5.51E-29	1.16E-27
ENSG00000196405	EVL	1192.380643	595.351589	1.00119893	7.45E-29	1.56E-27
ENSG00000129562	DAD1	2117.310373	1208.642053	0.809937224	8.45E-29	1.77E-27
ENSG00000260910	LINC00565	471.2616469	131.6499078	1.83362109	8.60E-29	1.80E-27
ENSG00000248334	WHAMMP2	342.648582	87.1371163	1.970186094	8.95E-29	1.87E-27
ENSG00000151012	SLC7A11	249.3847781	680.7217437	-1.449621581	9.36E-29	1.96E-27
ENSG00000054654	SYNE2	110.6145816	495.8129407	-2.161408399	1.02E-28	2.12E-27
ENSG00000110852	CLEC2B	780.81431	1459.727758	-0.901951364	1.08E-28	2.24E-27
ENSG00000105426	PTPRS	1193.369689	2156.145683	-0.854110675	1.10E-28	2.28E-27
ENSG00000168016	TRANK1	942.2198107	1647.914723	-0.806689926	1.11E-28	2.31E-27
ENSG00000259498	TPM1-AS	297.4477918	59.96112945	2.303405784	1.29E-28	2.69E-27
ENSG00000165244	ZNF367	39.96103769	257.3873335	-2.686493211	1.76E-28	3.65E-27
ENSG00000203706	SERTAD4-AS1	197.7205508	27.12950765	2.873597322	2.42E-28	4.99E-27
ENSG00000108381	ASPA	70.69193492	358.9886547	-2.34014707	2.48E-28	5.12E-27
ENSG00000099875	MKNK2	446.8881754	1038.51876	-1.214985829	2.64E-28	5.44E-27
ENSG00000144724	PTPRG	1149.675387	1965.96213	-0.773656407	2.76E-28	5.68E-27
ENSG00000253368	TRNP1	302.4615082	737.0478	-1.285707155	3.09E-28	6.34E-27
ENSG00000136859	ANGPTL2	2076.740693	3659.325941	-0.817283128	3.71E-28	7.61E-27
ENSG00000168268	NT5DC2	1881.429884	976.2402606	0.947156682	3.78E-28	7.75E-27
ENSG00000163064	EN1	834.6786839	373.721632	1.159633926	3.86E-28	7.90E-27
ENSG00000150594	ADRA2A	656.6930242	1393.082851	-1.084563621	3.93E-28	8.04E-27
ENSG00000070495	JMJD6	757.064756	331.3407235	1.193647423	3.97E-28	8.11E-27
ENSG00000134684	YARS	1829.487647	1015.349907	0.849542999	4.90E-28	1.00E-26
ENSG00000188641	DPYD	907.1406436	1671.256766	-0.881440275	5.75E-28	1.17E-26
ENSG00000119986	AVPI1	270.5360013	671.5944824	-1.312594669	6.09E-28	1.24E-26
ENSG00000113269	RNF130	457.8774424	982.0930472	-1.101747831	6.98E-28	1.42E-26
ENSG00000196588	MRTFA	1187.231406	591.1316883	1.008643122	7.10E-28	1.44E-26
ENSG00000163602	RYBP	2430.08	1437.838604	0.756405046	8.17E-28	1.66E-26
ENSG00000122877	EGR2	376.6783317	106.2198943	1.822441018	8.63E-28	1.75E-26
ENSG00000025708	TYMP	834.7793111	334.2424919	1.320539014	9.99E-28	2.02E-26
ENSG00000168528	SERINC2	1179.300711	585.7458666	1.011548662	1.07E-27	2.17E-26
ENSG00000186594	MIR22HG	1756.085602	922.0419798	0.929445047	1.08E-27	2.18E-26

ENSG00000103043	VAC14	1513.465467	809.4824306	0.90177429	1.18E-27	2.37E-26
ENSG00000184349	EFNA5	37.00528543	244.732632	-2.727327011	1.32E-27	2.65E-26
ENSG00000117069	ST6GALNAC5	666.7987975	252.7631595	1.395500328	1.35E-27	2.72E-26
ENSG00000145861	C1QTNF2	11.9833925	173.6585058	-3.853457692	1.40E-27	2.83E-26
ENSG00000189223	PAX8-AS1	931.1381365	1734.423815	-0.898382326	1.45E-27	2.91E-26
ENSG00000169583	CLIC3	422.5349975	130.0974094	1.69630702	1.47E-27	2.95E-26
ENSG00000154153	RETREG1	48.55038951	290.2025038	-2.574454124	1.52E-27	3.05E-26
ENSG00000106803	SEC61B	1354.594051	713.3019499	0.924444059	1.90E-27	3.82E-26
ENSG00000138835	RGS3	2363.100505	1351.998179	0.805289624	2.26E-27	4.52E-26
ENSG00000119314	PTBP3	1452.610359	800.7652808	0.858793459	2.66E-27	5.31E-26
ENSG00000157240	FZD1	1978.882131	1167.843491	0.761853749	2.92E-27	5.83E-26
ENSG00000177000	MTHFR	556.2620966	1103.370402	-0.989076088	2.99E-27	5.95E-26
ENSG00000166398	KIAA0355	1243.899083	589.3803182	1.078166693	3.42E-27	6.80E-26
ENSG00000168675	LDLRAD4	183.8422821	20.5416498	3.179318274	3.82E-27	7.59E-26
ENSG00000184156	KCNQ3	28.46361673	226.2638499	-2.990326018	3.88E-27	7.70E-26
ENSG00000185803	SLC52A2	792.8742831	351.1276498	1.174113097	3.89E-27	7.72E-26
ENSG00000188921	HACD4	254.6688945	674.9621303	-1.407035212	4.54E-27	8.99E-26
ENSG00000105855	ITGB8	67.50059925	322.2104533	-2.258160874	4.73E-27	9.37E-26
ENSG00000171608	PIK3CD	902.691495	409.3834474	1.141844018	5.04E-27	9.97E-26
ENSG00000125266	EFNB2	1677.968546	903.2154167	0.894745425	6.20E-27	1.22E-25
ENSG00000118263	KLF7	1032.803724	517.4377363	0.996411729	6.47E-27	1.27E-25
ENSG00000144791	LIMD1	274.603709	671.2280338	-1.288352877	8.87E-27	1.74E-25
ENSG00000028277	POU2F2	702.2234367	290.7336898	1.274716301	9.94E-27	1.95E-25
ENSG00000164300	SERINC5	1221.006145	602.6816109	1.020780645	1.11E-26	2.18E-25
ENSG00000169857	AVEN	801.4830083	364.0916963	1.139356843	1.12E-26	2.20E-25
ENSG00000165194	PCDH19	135.23696	4.446889133	4.889491063	1.13E-26	2.21E-25
ENSG00000170525	PFKFB3	835.7989876	378.9020641	1.141900861	1.72E-26	3.36E-25
ENSG00000155966	AFF2	22.09493399	200.2157449	-3.181569455	1.74E-26	3.40E-25
ENSG00000089060	SLC8B1	428.0718265	930.2902184	-1.12102918	1.77E-26	3.44E-25
ENSG00000116337	AMPD2	1146.554531	595.6810746	0.945219804	1.90E-26	3.70E-25
ENSG00000123643	SLC36A1	1738.399376	952.1731512	0.869071276	2.01E-26	3.91E-25
ENSG00000131791	PRKAB2	1464.733737	755.3890122	0.955014346	2.31E-26	4.49E-25
ENSG00000004776	HSPB6	218.1805711	671.8887232	-1.62155624	2.93E-26	5.66E-25
ENSG00000138639	ARHGAP24	561.8702724	1108.121251	-0.980270903	3.49E-26	6.73E-25
ENSG00000143079	CTTNBP2NL	1677.772116	938.4705594	0.837474269	3.69E-26	7.10E-25
ENSG00000227051	C14orf132	666.5221424	1280.612071	-0.941424819	3.71E-26	7.14E-25
ENSG00000133121	STARD13	1340.763849	717.8437438	0.901637917	4.04E-26	7.76E-25
ENSG00000099337	KCNK6	915.5365822	423.6057397	1.113536211	4.29E-26	8.23E-25
ENSG00000029534	ANK1	15.90549783	178.2093851	-3.478512658	4.57E-26	8.77E-25
ENSG00000100647	SUSD6	1039.830326	493.8873306	1.073828863	5.93E-26	1.14E-24
ENSG00000132329	RAMP1	134.0999637	8.034193484	4.081550632	6.47E-26	1.24E-24
ENSG00000119471	HSDL2	314.9779169	863.7757846	-1.455952528	7.46E-26	1.42E-24
ENSG00000120875	DUSP4	106.2776463	384.5533535	-1.853852686	7.96E-26	1.52E-24
ENSG00000124749	COL21A1	6.47397919	158.59369	-4.626520236	1.35E-25	2.57E-24
ENSG00000006016	CRLF1	962.9589083	459.9394916	1.066707278	1.55E-25	2.95E-24
ENSG00000125257	ABCC4	1240.755357	641.2748042	0.954274311	1.94E-25	3.67E-24
ENSG00000153714	LURAP1L	75.9360779	314.4125482	-2.052734457	2.06E-25	3.89E-24
ENSG00000066279	ASPM	222.5192665	565.799193	-1.347064968	2.37E-25	4.46E-24
ENSG00000128567	PODXL	272.7142447	659.2108838	-1.273612524	2.43E-25	4.57E-24
ENSG00000168952	STXBP6	12.78397689	172.5325581	-3.751293291	2.43E-25	4.57E-24
ENSG00000230630	DNM3OS	1250.021822	662.9769776	0.916442613	2.43E-25	4.57E-24
ENSG00000075651	PLD1	98.23021998	364.2317907	-1.887923385	2.55E-25	4.78E-24
ENSG00000081059	TCF7	109.9442068	388.6599522	-1.822997985	2.66E-25	4.98E-24
ENSG00000148344	PTGES	142.6269284	465.5350016	-1.706991353	3.16E-25	5.90E-24
ENSG00000131747	TOP2A	476.8838617	1005.751991	-1.076970482	3.33E-25	6.23E-24
ENSG00000156113	KCNMA1	813.999978	1484.396554	-0.867353676	3.55E-25	6.63E-24
ENSG00000150787	PTS	827.5601369	397.9777025	1.057096751	3.71E-25	6.91E-24
ENSG00000164985	PSIP1	584.1394743	1096.300214	-0.908221647	4.15E-25	7.73E-24
ENSG00000170271	FAXDC2	169.3304238	483.1971726	-1.513207306	4.67E-25	8.67E-24
ENSG00000135749	PCNX2	266.8241563	646.0403847	-1.277236911	5.02E-25	9.31E-24
ENSG00000139998	RAB15	769.9870084	356.3143503	1.113274162	5.97E-25	1.11E-23
ENSG00000112367	FIG4	561.1834334	1084.029941	-0.949719976	6.27E-25	1.16E-23
ENSG00000131871	SELENOS	1387.438252	771.9365061	0.845870503	6.33E-25	1.17E-23
ENSG00000102466	FGF14	469.8695123	160.6447211	1.550324735	6.86E-25	1.26E-23
ENSG00000164929	BAALC	194.4935553	562.645771	-1.532122477	6.87E-25	1.26E-23
ENSG00000189129	PLAC9	299.9594372	762.3197397	-1.34523476	7.30E-25	1.34E-23

ENSG00000106049	HIBADH	717.4167991	1386.245238	-0.950433813	8.18E-25	1.50E-23
ENSG00000166444	ST5	1939.224862	1046.175902	0.889429063	9.77E-25	1.79E-23
ENSG00000163659	TIPARP	935.1055767	400.4427369	1.226891484	1.25E-24	2.30E-23
ENSG00000143179	UCK2	1089.30301	585.9886613	0.895691601	1.28E-24	2.35E-23
ENSG00000196083	IL1RAP	503.5819955	177.1061459	1.512569274	1.49E-24	2.72E-23
ENSG00000105088	OLFM2	231.3190064	42.46456644	2.437876139	1.55E-24	2.83E-23
ENSG00000224259	LINC01133	35.46190808	235.9143091	-2.737905104	1.87E-24	3.40E-23
ENSG00000206052	DOK6	646.3200655	244.4009858	1.406543221	1.98E-24	3.59E-23
ENSG00000171345	KRT19	86.64588168	323.6686688	-1.902158303	1.98E-24	3.59E-23
ENSG00000155850	SLC26A2	1487.614397	746.6364036	0.996650233	2.05E-24	3.72E-23
ENSG00000117479	SLC19A2	534.6194396	205.9669295	1.380101583	2.22E-24	4.03E-23
ENSG00000117410	ATP6V0B	1666.455655	866.4661675	0.940981791	2.36E-24	4.27E-23
ENSG00000144218	AFF3	371.4338325	92.26004914	2.001250625	2.60E-24	4.70E-23
ENSG00000185924	RTN4RL1	24.04257264	186.4366467	-2.956889575	2.80E-24	5.05E-23
ENSG00000185339	TCN2	236.9783623	590.4487431	-1.316140802	2.84E-24	5.12E-23
ENSG00000115504	EHBP1	1494.221882	2546.186281	-0.768792159	3.09E-24	5.56E-23
ENSG00000135069	PSAT1	477.9191862	173.8524339	1.462729874	3.84E-24	6.90E-23
ENSG00000166130	IKBIP	1661.51946	969.0124395	0.778847466	4.12E-24	7.41E-23
ENSG00000111452	ADGRD1	225.5918781	584.9946171	-1.375741864	4.84E-24	8.69E-23
ENSG00000163071	SPATA18	1037.272885	1830.78355	-0.82005866	5.99E-24	1.07E-22
ENSG00000116991	SIPA1L2	84.58518647	351.2508859	-2.052208752	6.14E-24	1.10E-22
ENSG00000279118	AC093535.2	507.4843175	187.2335273	1.439694966	7.40E-24	1.32E-22
ENSG00000145431	PDGFC	2116.66172	1246.935382	0.763939526	9.02E-24	1.61E-22
ENSG00000182957	SPATA13	396.4172304	834.5728103	-1.07547565	1.19E-23	2.13E-22
ENSG00000183386	FHL3	855.5910819	432.3527257	0.985512954	1.27E-23	2.25E-22
ENSG00000144867	SRPRB	994.1360322	500.621086	0.991456533	1.54E-23	2.74E-22
ENSG00000177707	NECTIN3	544.2594314	1058.13966	-0.959061554	1.56E-23	2.78E-22
ENSG00000143801	PSEN2	292.2203182	689.5154353	-1.238150933	1.64E-23	2.91E-22
ENSG00000134575	ACP2	823.4937832	399.1853618	1.043728263	1.87E-23	3.31E-22
ENSG00000170775	GPR37	205.483817	546.1450293	-1.412725455	2.00E-23	3.55E-22
ENSG00000143367	TUFT1	611.5679446	237.3472605	1.364743711	2.29E-23	4.05E-22
ENSG00000147202	DIAPH2	629.9468479	1194.264719	-0.921723583	2.78E-23	4.90E-22
ENSG00000113369	ARRDC3	485.4853374	972.713918	-1.001350035	3.14E-23	5.54E-22
ENSG00000178033	CALHM5	929.7354367	435.2319777	1.097052255	3.29E-23	5.77E-22
ENSG00000138166	DUSP5	58.45445212	343.424005	-2.557111726	3.35E-23	5.87E-22
ENSG00000132031	MATN3	248.8738397	46.37291527	2.421560447	3.54E-23	6.21E-22
ENSG00000147044	CASK	1229.121932	666.9936384	0.883108659	3.63E-23	6.36E-22
ENSG00000089041	P2RX7	17.6706481	160.1876893	-3.182872942	3.99E-23	6.99E-22
ENSG00000134375	TIMM17A	1219.470246	627.6234669	0.958142333	4.10E-23	7.18E-22
ENSG00000021762	OSBPL5	1523.59184	834.3135984	0.866254679	4.13E-23	7.22E-22
ENSG00000171246	NPTX1	1.954122261	219.1919533	-6.777304891	4.95E-23	8.66E-22
ENSG00000175592	FOSL1	391.7135582	811.255016	-1.050369824	5.04E-23	8.79E-22
ENSG00000223485	LINC01615	607.9455196	268.0778865	1.182885413	5.14E-23	8.96E-22
ENSG00000164530	PI16	395.2347447	112.1905205	1.810054583	5.26E-23	9.16E-22
ENSG00000115363	EVA1A	619.9725876	243.7115693	1.346818531	5.35E-23	9.32E-22
ENSG00000174600	CMKLR1	339.9909784	836.7381725	-1.296935354	5.73E-23	9.97E-22
ENSG00000136888	ATP6V1G1	1404.822876	807.3146473	0.800411694	6.63E-23	1.15E-21
ENSG00000107554	DNMBP	349.8448819	763.4232292	-1.126596866	6.81E-23	1.18E-21
ENSG00000126950	TMEM35A	23.49612888	589.2361046	-4.646031277	6.91E-23	1.20E-21
ENSG00000176842	IRX5	779.3880506	358.2417153	1.122329573	7.57E-23	1.31E-21
ENSG00000153132	CLGN	24.79095561	175.3007154	-2.820081346	8.93E-23	1.54E-21
ENSG00000238042	LINC02257	120.8706538	4.899598834	4.68718099	9.12E-23	1.57E-21
ENSG00000159167	STC1	26.94739998	187.2798032	-2.801362313	1.10E-22	1.89E-21
ENSG00000124785	NRN1	907.6999036	1580.349618	-0.798875604	1.12E-22	1.93E-21
ENSG00000168291	PDHB	1328.392532	781.4905211	0.765963169	1.16E-22	1.99E-21
ENSG00000261786	AC006058.1	247.2785432	611.0472702	-1.305849454	1.43E-22	2.46E-21
ENSG00000173376	NDNF	30.2652701	195.1306589	-2.684212859	1.57E-22	2.69E-21
ENSG00000104856	RELB	398.4594961	141.4205592	1.49287767	1.80E-22	3.07E-21
ENSG00000110987	BCL7A	543.3568694	207.2863635	1.392605942	1.89E-22	3.23E-21
ENSG00000127526	SLC35E1	1416.532193	835.0337113	0.763376897	1.99E-22	3.39E-21
ENSG00000154027	AK5	663.1835449	1184.890353	-0.836886397	2.01E-22	3.43E-21
ENSG00000183779	ZNF703	591.3275809	1080.348354	-0.869192541	2.19E-22	3.73E-21
ENSG00000244879	GABPB1-AS1	383.7700507	890.1224989	-1.214729119	2.47E-22	4.19E-21
ENSG00000106080	FKBP14	1267.716363	671.6846243	0.91893883	2.61E-22	4.43E-21
ENSG00000162783	IER5	711.4949815	1253.690793	-0.817908079	2.86E-22	4.83E-21
ENSG00000198585	NUDT16	582.2450081	1112.371204	-0.932727355	3.20E-22	5.40E-21

ENSG00000164850	GPER1	116.0256154	390.4753633	-1.749667478	3.30E-22	5.56E-21
ENSG00000130758	MAP3K10	607.1143547	273.8524238	1.148518269	3.41E-22	5.74E-21
ENSG00000134909	ARHGAP32	925.5027769	478.7842148	0.951776656	3.59E-22	6.03E-21
ENSG00000169251	NMD3	1229.864802	633.7497568	0.959085211	3.78E-22	6.34E-21
ENSG00000102452	NALCN	268.8212653	67.57071806	1.984842607	4.13E-22	6.92E-21
ENSG00000172057	ORMDL3	1030.642916	555.4387704	0.891080305	4.15E-22	6.95E-21
ENSG00000197785	ATAD3A	891.3042214	458.1037839	0.961692563	4.17E-22	6.99E-21
ENSG00000197301	AC090673.1	83.52990043	337.246269	-2.013481152	4.53E-22	7.56E-21
ENSG00000103316	CRYM	26.86423513	182.5775715	-2.764231307	5.20E-22	8.66E-21
ENSG00000213523	SRA1	787.5514161	381.7209994	1.047626078	5.52E-22	9.17E-21
ENSG00000111711	GOLT1B	1282.766777	691.841915	0.890588418	5.53E-22	9.18E-21
ENSG00000164463	CREBRF	536.3370849	989.414878	-0.883125941	5.60E-22	9.29E-21
ENSG00000130762	ARHGEF16	113.5557843	3.367217591	5.114869586	6.01E-22	9.96E-21
ENSG00000186951	PPARA	659.929567	1158.334415	-0.81156596	6.31E-22	1.04E-20
ENSG00000056736	IL17RB	59.41444936	269.8431057	-2.179038214	6.59E-22	1.09E-20
ENSG00000196365	LONP1	1987.683531	1128.871584	0.814392008	7.87E-22	1.30E-20
ENSG00000140511	HAPLN3	806.9386378	400.9088534	1.007429966	1.14E-21	1.88E-20
ENSG00000137145	DENND4C	670.4136908	1227.126606	-0.871895776	1.35E-21	2.21E-20
ENSG00000167992	VWCE	48.78367503	236.51165	-2.275561746	1.42E-21	2.32E-20
ENSG00000163814	CDCP1	15.54840896	160.9383168	-3.370224374	1.54E-21	2.52E-20
ENSG00000119943	PYROXD2	130.701277	380.6690144	-1.542072333	1.64E-21	2.69E-20
ENSG00000119508	NR4A3	130.4822352	407.2379066	-1.641928293	1.88E-21	3.07E-20
ENSG00000157214	STEAP2	1216.809682	597.2570828	1.028938783	2.07E-21	3.36E-20
ENSG00000142694	EVA1B	939.1526613	1590.97147	-0.761609328	2.60E-21	4.22E-20
ENSG00000157045	NTAN1	689.4757581	1237.991254	-0.844914909	3.19E-21	5.17E-20
ENSG00000170456	DENND5B	850.8725009	432.2059333	0.977351176	3.19E-21	5.17E-20
ENSG00000104884	ERCC2	1157.533717	624.0418095	0.8911982	3.23E-21	5.23E-20
ENSG00000205336	ADGRG1	15.24855051	148.6863951	-3.287675535	3.29E-21	5.31E-20
ENSG00000182463	TSHZ2	168.0471097	456.9268202	-1.442506056	3.32E-21	5.37E-20
ENSG00000237125	HAND2-AS1	151.6836945	428.9394589	-1.499416699	3.61E-21	5.82E-20
ENSG00000067177	PHKA1	277.820801	81.02423479	1.779740533	3.64E-21	5.87E-20
ENSG00000084731	KIF3C	1326.637006	764.2804028	0.795831505	4.33E-21	6.96E-20
ENSG00000181072	CHRM2	41.60054709	218.7333941	-2.391705319	5.05E-21	8.12E-20
ENSG00000050628	PTGER3	364.7656979	122.8678926	1.571977203	5.57E-21	8.94E-20
ENSG00000149218	ENDOD1	356.8883034	747.7013893	-1.065633234	6.48E-21	1.04E-19
ENSG00000250722	SELENOP	28.58690939	186.6787733	-2.712191712	6.87E-21	1.10E-19
ENSG00000134202	GSTM3	760.5552346	1299.523664	-0.772521695	7.35E-21	1.18E-19
ENSG00000131386	GALNT15	44.48084428	291.9281916	-2.710199219	8.23E-21	1.32E-19
ENSG00000136928	GABBR2	333.1234339	786.2184822	-1.238669233	8.44E-21	1.35E-19
ENSG00000197780	TAF13	1050.180615	577.1686075	0.865049191	8.71E-21	1.39E-19
ENSG00000144857	BOC	583.003832	1119.095275	-0.941470808	8.74E-21	1.39E-19
ENSG00000197956	S100A6	10075.0775	22002.38503	-1.126878828	9.17E-21	1.46E-19
ENSG00000128536	CDHR3	96.96617856	316.165446	-1.706426921	9.69E-21	1.54E-19
ENSG00000125965	GDF5	4.320776625	131.6347692	-4.906203528	1.04E-20	1.65E-19
ENSG00000167703	SLC43A2	276.4916413	77.89331741	1.831346596	1.13E-20	1.80E-19
ENSG00000137752	CASP1	53.74322466	245.9270988	-2.193648161	1.22E-20	1.93E-19
ENSG00000175048	ZDHHC14	66.9860125	261.0628331	-1.961897531	1.56E-20	2.46E-19
ENSG00000116678	LEPR	152.2603862	435.881636	-1.516413231	2.18E-20	3.43E-19
ENSG00000071051	NCK2	836.3471144	442.6054926	0.919147457	2.54E-20	3.99E-19
ENSG00000138696	BMPR1B	170.9801693	26.51118972	2.689217543	2.65E-20	4.16E-19
ENSG00000278962	AC092645.1	24.95583069	168.5954714	-2.76049506	2.72E-20	4.26E-19
ENSG00000120278	PLEKHG1	29.0165441	187.9149679	-2.698017474	2.72E-20	4.26E-19
ENSG00000105894	PTN	97.95216056	332.2526374	-1.761218087	3.08E-20	4.81E-19
ENSG00000013588	GPRC5A	19.85208297	163.2675789	-3.031675989	3.66E-20	5.72E-19
ENSG00000198736	MSRB1	638.9019423	307.2669044	1.057372027	3.78E-20	5.90E-19
ENSG00000134285	FKBP11	613.3952265	287.6970439	1.093784354	3.97E-20	6.19E-19
ENSG00000035681	NSMAF	826.4553031	1397.485589	-0.758520421	4.84E-20	7.54E-19
ENSG00000261888	AC144831.1	167.2418289	427.055982	-1.353119811	5.20E-20	8.09E-19
ENSG00000158987	RAPGEF6	123.9659581	392.8543279	-1.662951879	5.66E-20	8.78E-19
ENSG00000187210	GCNT1	622.1125946	1169.338012	-0.909861516	6.95E-20	1.08E-18
ENSG00000112297	CRYBG1	363.016656	750.9838624	-1.047495099	7.46E-20	1.15E-18
ENSG00000140479	PCSK6	8.756575111	128.4227614	-3.866292413	8.15E-20	1.26E-18
ENSG00000163710	PCOLCE2	58.49509062	242.5516671	-2.050345872	8.53E-20	1.32E-18
ENSG00000137266	SLC22A23	946.7007156	498.9680826	0.922880318	8.67E-20	1.34E-18
ENSG00000107036	RIC1	1545.911966	877.0317648	0.815442554	9.47E-20	1.46E-18
ENSG00000162433	AK4	228.8931563	535.4741179	-1.224280981	1.07E-19	1.65E-18

ENSG00000162599	NFIA	32.35603514	208.4736907	-2.693114308	1.09E-19	1.67E-18
ENSG00000196935	SRGAP1	576.3652565	1050.949471	-0.865955914	1.18E-19	1.81E-18
ENSG00000155792	DEPTOR	184.7018841	40.25549668	2.19240965	1.52E-19	2.33E-18
ENSG00000134802	SLC43A3	130.2790195	363.7940046	-1.482612477	1.58E-19	2.43E-18
ENSG00000116704	SLC35D1	1078.01424	614.7919456	0.810906336	1.93E-19	2.95E-18
ENSG00000153944	MSI2	552.6700473	245.8475594	1.167426027	1.95E-19	2.97E-18
ENSG00000213281	NRAS	1505.674196	893.0477884	0.754088841	2.34E-19	3.57E-18
ENSG00000139209	SLC38A4	245.6763027	70.82443002	1.79132113	2.47E-19	3.76E-18
ENSG00000153048	CARHSP1	355.7156473	724.2076111	-1.024862772	2.77E-19	4.21E-18
ENSG00000119514	GALNT12	12.8010795	126.5602617	-3.303385124	3.08E-19	4.67E-18
ENSG00000146966	DENND2A	31.62889019	547.118765	-4.116490009	3.31E-19	5.02E-18
ENSG00000120549	KIAA1217	409.5933949	780.1421223	-0.930129778	3.34E-19	5.07E-18
ENSG00000241553	ARPC4	1412.359162	818.275682	0.789212024	3.52E-19	5.34E-18
ENSG00000173548	SNX33	1118.269499	1892.043997	-0.758319671	3.72E-19	5.63E-18
ENSG00000103184	SEC14L5	10.28106266	130.8231682	-3.656153334	4.57E-19	6.91E-18
ENSG00000167797	CDK2AP2	605.5092553	280.5771403	1.108474031	4.91E-19	7.40E-18
ENSG00000090006	LTBP4	630.1032237	1137.415545	-0.853201953	4.98E-19	7.51E-18
ENSG00000117525	F3	1293.048632	674.6037157	0.939281329	4.99E-19	7.51E-18
ENSG00000183856	IQGAP3	125.5728713	379.6503039	-1.59793855	5.20E-19	7.83E-18
ENSG00000116791	CRYZ	410.4625176	800.5648585	-0.962241629	6.32E-19	9.50E-18
ENSG00000172346	CSDC2	303.9510247	100.4423246	1.598963672	6.59E-19	9.88E-18
ENSG00000203721	LINC00862	99.50939871	6.636283182	3.889481926	7.00E-19	1.05E-17
ENSG00000173530	TNFRSF10D	888.6086838	467.1962421	0.929498778	7.36E-19	1.10E-17
ENSG00000273038	AL365203.2	1238.15363	720.2691878	0.781841203	7.66E-19	1.15E-17
ENSG00000157557	ETS2	94.49606526	323.6861831	-1.779929631	8.29E-19	1.24E-17
ENSG00000167566	NCKAP5L	1238.876462	723.0380132	0.77647958	8.75E-19	1.31E-17
ENSG00000259370	AC103740.1	132.2843992	15.19907173	3.134690059	9.60E-19	1.43E-17
ENSG00000248290	TNXA	2.404994985	148.4736399	-5.945606754	9.72E-19	1.45E-17
ENSG00000072832	CRMP1	523.0983318	1019.124226	-0.961959949	1.38E-18	2.06E-17
ENSG00000037965	HOXC8	1076.319391	627.5296605	0.778061434	1.48E-18	2.19E-17
ENSG00000101096	NFATC2	22.3337592	149.825094	-2.741822603	1.73E-18	2.58E-17
ENSG00000138669	PRKG2	142.483241	23.68445856	2.585152655	1.78E-18	2.64E-17
ENSG00000132170	PPARG	17.20915637	135.9796026	-2.980591301	1.99E-18	2.94E-17
ENSG00000131069	ACSS2	682.5119774	1192.267963	-0.804748268	2.18E-18	3.22E-17
ENSG00000114738	MAPKAPK3	479.5196662	909.5896823	-0.923825507	2.23E-18	3.30E-17
ENSG00000171132	PRKCE	150.8942901	396.6969749	-1.396312087	2.81E-18	4.14E-17
ENSG00000081913	PHLPP1	118.5996208	337.4871184	-1.507551509	3.04E-18	4.48E-17
ENSG00000164976	MYORG	203.5210411	51.34299107	1.985857963	3.06E-18	4.50E-17
ENSG00000167371	PRRT2	44.17544615	202.4721652	-2.199129348	3.23E-18	4.73E-17
ENSG00000168256	NKIRAS2	865.8943803	410.9685871	1.072275945	3.42E-18	5.01E-17
ENSG00000146376	ARHGAP18	551.0824068	1022.791523	-0.892229407	3.55E-18	5.19E-17
ENSG00000168938	PPIC	1189.17713	608.7603685	0.96331497	3.64E-18	5.32E-17
ENSG00000204219	TCEA3	112.1445314	328.1023537	-1.547254605	3.83E-18	5.59E-17
ENSG00000162814	SPATA17	84.57349576	3.200964556	4.707356539	4.40E-18	6.42E-17
ENSG00000132481	TRIM47	79.20447772	274.2175835	-1.793025965	4.55E-18	6.62E-17
ENSG00000265107	GJA5	94.16212937	3.874190809	4.626872485	4.84E-18	7.05E-17
ENSG00000155380	SLC16A1	1161.087651	642.9152825	0.854140995	4.95E-18	7.18E-17
ENSG00000138778	CENPE	211.4921691	485.894626	-1.201538089	5.30E-18	7.69E-17
ENSG00000162298	SYVN1	957.7516277	553.2495796	0.791902626	6.70E-18	9.70E-17
ENSG00000163827	LRRC2	381.8882695	734.8733178	-0.945541439	7.07E-18	1.02E-16
ENSG00000221963	APOL6	391.1592266	758.2261423	-0.954004112	8.07E-18	1.16E-16
ENSG00000132432	SEC61G	997.5000026	459.7630555	1.120376925	8.51E-18	1.23E-16
ENSG00000124216	SNAI1	210.6080369	55.33306344	1.924418276	9.58E-18	1.38E-16
ENSG00000135218	CD36	0.814445202	373.5119205	-8.864064709	1.03E-17	1.48E-16
ENSG00000060656	PTPRU	599.4843114	1045.646436	-0.802517516	1.29E-17	1.85E-16
ENSG00000114923	SLC4A3	479.3150733	188.5957632	1.344952088	1.38E-17	1.97E-16
ENSG00000221852	KRTAP1-5	21.87175678	170.7414187	-2.972515715	1.47E-17	2.10E-16
ENSG00000131080	EDA2R	273.1865839	653.3840291	-1.25795088	1.49E-17	2.12E-16
ENSG00000109472	CPE	221.5986312	60.28628099	1.870493991	1.49E-17	2.12E-16
ENSG00000072609	CHFR	930.7469973	507.9384026	0.871240958	1.58E-17	2.24E-16
ENSG00000189410	SH2D5	215.4723984	491.902658	-1.191459536	1.60E-17	2.27E-16
ENSG00000107643	MAPK8	640.9873458	309.1404358	1.054624211	1.75E-17	2.48E-16
ENSG00000136869	TLR4	699.979998	356.7651139	0.9742408	1.77E-17	2.51E-16
ENSG00000275993	SIK1B	82.95522436	4.258584466	4.266670518	1.88E-17	2.66E-16
ENSG00000066027	PPP2R5A	457.4746861	887.6455503	-0.956545921	1.90E-17	2.69E-16
ENSG00000151366	NDUFC2	421.4567869	174.8099557	1.270684142	1.98E-17	2.80E-16

ENSG00000092010	PSME1	650.483472	1100.314285	-0.757812177	2.16E-17	3.05E-16
ENSG00000148572	NRBF2	815.6653982	422.50207	0.947401459	2.27E-17	3.20E-16
ENSG00000100100	PIK3IP1	984.9257255	561.9650102	0.808834864	2.53E-17	3.57E-16
ENSG00000105989	WNT2	840.5545286	448.2808152	0.907987895	2.91E-17	4.09E-16
ENSG00000104131	EIF3J	937.6198762	540.0747235	0.795707681	2.92E-17	4.11E-16
ENSG00000185420	SMYD3	630.6855825	306.4924939	1.043413932	3.06E-17	4.30E-16
ENSG00000132199	ENOSF1	430.5167445	786.8672685	-0.870607339	3.29E-17	4.60E-16
ENSG00000074047	GLI2	259.8180545	81.88512091	1.666662114	3.58E-17	5.00E-16
ENSG00000170345	FOS	246.2802053	636.2098815	-1.370856765	3.61E-17	5.04E-16
ENSG00000172201	ID4	104.2112333	8.901777598	3.593696583	3.72E-17	5.19E-16
ENSG00000073008	PVR	935.3142818	537.1753542	0.798726516	3.92E-17	5.46E-16
ENSG00000169330	KIAA1024	233.4643211	71.6896382	1.702723605	5.68E-17	7.89E-16
ENSG00000164070	HSPA4L	137.4963526	358.3167293	-1.380898939	6.41E-17	8.87E-16
ENSG00000111261	MANSC1	50.08587897	197.2506506	-1.977923543	6.67E-17	9.23E-16
ENSG00000171502	COL24A1	132.8457755	17.18286133	2.975804577	7.35E-17	1.01E-15
ENSG00000138604	GLCE	326.6063136	636.9723543	-0.964151662	7.63E-17	1.05E-15
ENSG00000196396	PTPN1	986.4584838	559.9310528	0.818634243	1.11E-16	1.53E-15
ENSG00000214717	ZBED1	1243.517673	736.6338084	0.754071413	1.17E-16	1.61E-15
ENSG00000141682	PMAIP1	58.77046925	224.9035599	-1.934032986	1.21E-16	1.66E-15
ENSG00000157873	TNFRSF14	414.7734677	883.4987393	-1.092736331	1.22E-16	1.67E-15
ENSG00000164776	PHKG1	17.73671034	131.0858419	-2.89110124	1.23E-16	1.68E-15
ENSG00000163947	ARHGEF3	104.0139402	312.2921038	-1.583397793	1.23E-16	1.69E-15
ENSG00000184792	OSBP2	61.41842484	230.0597794	-1.901127327	1.39E-16	1.90E-15
ENSG00000184988	TMEM106A	354.4374628	134.5352246	1.392265831	1.51E-16	2.06E-15
ENSG00000099992	TBC1D10A	574.290472	263.2472598	1.122217962	1.53E-16	2.09E-15
ENSG00000138073	PREB	924.1753037	534.9239125	0.789252387	1.53E-16	2.09E-15
ENSG00000182179	UBA7	334.1204682	673.8364208	-1.013194327	1.61E-16	2.19E-15
ENSG00000182704	TSKU	759.3110773	391.3779529	0.953727448	1.63E-16	2.23E-15
ENSG00000028137	TNFRSF1B	46.7236953	195.3810114	-2.06270075	1.66E-16	2.25E-15
ENSG00000121989	ACVR2A	1126.70393	637.4155373	0.823957902	1.89E-16	2.57E-15
ENSG00000259091	LINC00517	5.578717349	102.6691124	-4.194046275	2.10E-16	2.85E-15
ENSG00000113448	PDE4D	185.8311202	42.49353107	2.135280148	2.19E-16	2.97E-15
ENSG00000227039	ITGB2-AS1	78.94168338	4.921650467	4.01303486	2.22E-16	3.01E-15
ENSG00000261573	AL157402.2	96.65575143	5.486564468	4.10919377	3.15E-16	4.26E-15
ENSG00000171310	CHST11	515.5725871	245.398957	1.071864778	3.29E-16	4.44E-15
ENSG00000198018	ENTPD7	771.8864532	423.1469766	0.866651466	3.64E-16	4.90E-15
ENSG00000169122	FAM110B	165.0668776	411.3179404	-1.317233181	3.65E-16	4.92E-15
ENSG00000153823	PID1	400.4617348	171.762708	1.222813577	3.66E-16	4.93E-15
ENSG00000161091	MFSD12	1127.703544	661.3654863	0.768119587	3.73E-16	5.02E-15
ENSG00000166689	PLEKHA7	318.8421034	115.8482903	1.463856353	4.06E-16	5.45E-15
ENSG00000154654	NCAM2	137.45689	18.40176741	2.899577964	4.11E-16	5.51E-15
ENSG00000151572	ANO4	14.08439364	115.6489656	-3.042039449	4.64E-16	6.21E-15
ENSG00000147324	MFHAS1	838.6765502	476.2698957	0.816382619	4.80E-16	6.41E-15
ENSG00000053747	LAMA3	46.2829309	194.0870915	-2.070453306	5.08E-16	6.77E-15
ENSG00000186310	NAP1L3	231.6638939	72.62553836	1.678738231	5.28E-16	7.04E-15
ENSG00000047648	ARHGAP6	43.74313066	186.2327736	-2.090861791	5.52E-16	7.34E-15
ENSG00000102554	KLF5	359.2672362	146.5932072	1.296814067	5.56E-16	7.40E-15
ENSG00000177674	AGTRAP	918.7598523	527.921622	0.80068341	6.23E-16	8.26E-15
ENSG00000074370	ATP2A3	477.254838	938.6527874	-0.976364454	6.73E-16	8.92E-15
ENSG00000145244	CORIN	129.3759975	20.73989971	2.630924915	6.83E-16	9.04E-15
ENSG00000135124	P2RX4	295.3208563	609.7297476	-1.046092593	7.59E-16	1.00E-14
ENSG00000164093	PITX2	639.9183267	328.2768206	0.963339533	8.15E-16	1.07E-14
ENSG00000171840	NINJ2	155.5832853	34.58668074	2.17233666	8.61E-16	1.13E-14
ENSG00000270069	MIR222HG	106.1090188	313.2265221	-1.563421129	8.86E-16	1.17E-14
ENSG00000154511	FAM69A	961.1529681	547.7377393	0.809395701	9.97E-16	1.31E-14
ENSG00000126878	AIF1L	1.607652394	124.949619	-6.286134993	1.01E-15	1.32E-14
ENSG00000154767	XPC	511.2893079	929.1537034	-0.863536269	1.06E-15	1.40E-14
ENSG00000164949	GEM	174.8029008	406.8425159	-1.219324045	1.16E-15	1.52E-14
ENSG00000110660	SLC35F2	321.4761207	129.4784469	1.313543652	1.28E-15	1.68E-14
ENSG00000156026	MCU	930.1921313	517.9606962	0.843970903	1.29E-15	1.68E-14
ENSG00000110318	CEP126	125.7292708	346.3272775	-1.460661693	1.53E-15	2.00E-14
ENSG00000186260	MRTFB	339.1428116	647.0430848	-0.931635969	1.54E-15	2.01E-14
ENSG00000188015	S100A3	62.66937127	215.0096725	-1.78064797	1.62E-15	2.10E-14
ENSG00000167645	YIF1B	1011.939396	536.1160262	0.913782136	1.71E-15	2.22E-14
ENSG00000241839	PLEKHO2	796.4802435	1341.00265	-0.752905707	1.78E-15	2.31E-14
ENSG00000172115	CYCS	1162.423012	659.6296624	0.818571627	1.79E-15	2.32E-14

ENSG00000072506	HSD17B10	606.2615186	288.8802876	1.070529546	1.81E-15	2.35E-14
ENSG00000153976	HS3ST3A1	454.9342241	203.2859156	1.160417501	1.83E-15	2.38E-14
ENSG00000148053	NTRK2	9.129872999	110.3040116	-3.587053498	1.90E-15	2.47E-14
ENSG00000173432	SAA1	217.3955573	0.234354069	9.534603636	1.94E-15	2.52E-14
ENSG00000138180	CEP55	112.0234089	315.941897	-1.497895247	2.02E-15	2.62E-14
ENSG00000082438	COBLL1	656.8783714	358.5800595	0.874571607	2.26E-15	2.92E-14
ENSG00000105722	ERF	940.1057649	556.9082205	0.754611893	2.27E-15	2.93E-14
ENSG00000179431	FJX1	609.5021968	304.6066526	0.99768187	2.38E-15	3.07E-14
ENSG00000128951	DUT	506.1831616	868.3757801	-0.778727421	2.93E-15	3.77E-14
ENSG00000157693	TMEM268	294.171249	109.3605421	1.425213345	3.18E-15	4.09E-14
ENSG00000177096	PHETA2	1037.133027	611.6089252	0.760188566	3.32E-15	4.26E-14
ENSG00000175567	UCP2	386.2928494	166.6588058	1.210888643	3.44E-15	4.40E-14
ENSG00000197019	SERTAD1	327.5164576	130.4178093	1.331684646	3.73E-15	4.78E-14
ENSG00000137672	TRPC6	24.05408524	133.3584759	-2.471484374	3.78E-15	4.83E-14
ENSG00000214688	C10orf105	8.382383629	101.9744793	-3.598537241	4.72E-15	6.01E-14
ENSG00000152422	XRCC4	271.9367363	64.52086791	2.080637077	6.24E-15	7.92E-14
ENSG00000133216	EPHB2	175.0407046	46.76400692	1.90571267	6.95E-15	8.80E-14
ENSG00000138675	FGF5	482.4534649	838.0989155	-0.79567744	7.18E-15	9.08E-14
ENSG00000176532	PRR15	121.4919916	18.53429221	2.691075328	7.60E-15	9.61E-14
ENSG00000169255	B3GALNT1	492.7860402	245.9614834	1.003103471	7.61E-15	9.62E-14
ENSG00000188393	CLEC2A	582.6516947	297.4186364	0.967367073	8.21E-15	1.04E-13
ENSG00000106511	MEOX2	151.1059547	370.8884061	-1.295165356	1.08E-14	1.36E-13
ENSG00000168079	SCARA5	0	144.6418444	-9.351962513	1.10E-14	1.38E-13
ENSG00000122547	EEPD1	264.1070245	526.1548748	-0.993687499	1.12E-14	1.41E-13
ENSG00000119950	MXI1	356.9242183	691.6455757	-0.955255516	1.21E-14	1.52E-13
ENSG00000213190	MLLT11	240.868661	81.10552854	1.570531781	1.22E-14	1.53E-13
ENSG00000141510	TP53	798.9977577	462.1965451	0.790723941	1.23E-14	1.53E-13
ENSG00000155962	CLIC2	22.38182523	129.9216411	-2.533908454	1.25E-14	1.57E-13
ENSG00000198042	MAK16	470.8590183	230.0805415	1.035132686	1.53E-14	1.91E-13
ENSG00000260285	AL133367.1	41.63475232	177.3180183	-2.089423902	1.56E-14	1.95E-13
ENSG00000139910	NOVA1	19.53009292	119.3869543	-2.607569902	1.59E-14	1.98E-13
ENSG00000187840	EIF4EBP1	386.7822676	164.3496379	1.230956198	1.62E-14	2.01E-13
ENSG00000158850	B4GALT3	581.7571216	312.5418112	0.897447745	1.62E-14	2.01E-13
ENSG00000076770	MBNL3	37.10192818	168.1118512	-2.177664463	1.65E-14	2.05E-13
ENSG00000138386	NAB1	996.8789707	591.2088748	0.754809848	1.71E-14	2.13E-13
ENSG00000112039	FANCE	74.37286683	234.9036097	-1.660315285	2.19E-14	2.70E-13
ENSG00000197442	MAP3K5	69.20902979	224.5018665	-1.695575634	2.37E-14	2.92E-13
ENSG00000172331	BPGM	581.7854546	281.9778678	1.041375213	2.44E-14	3.01E-13
ENSG00000108551	RASD1	10.17614911	107.2498392	-3.412972694	2.51E-14	3.10E-13
ENSG00000227456	LINC00310	72.54438544	6.043264354	3.597411811	2.62E-14	3.23E-13
ENSG00000162928	PEX13	612.8142721	320.4962388	0.935395648	2.66E-14	3.27E-13
ENSG00000153815	CMIP	859.9350619	496.287296	0.79294189	2.66E-14	3.27E-13
ENSG00000134058	CDK7	595.1500301	319.8467953	0.894875109	2.75E-14	3.38E-13
ENSG00000184232	OAF	197.3245603	444.1845683	-1.168754842	2.76E-14	3.38E-13
ENSG00000162267	ITIH3	69.72596475	4.452942328	3.994600476	2.84E-14	3.48E-13
ENSG00000079337	RAPGEF3	86.14045932	256.8126773	-1.577227084	2.87E-14	3.52E-13
ENSG00000102996	MMP15	371.8401095	167.5726543	1.149850111	3.04E-14	3.72E-13
ENSG00000058085	LAMC2	42.40940228	165.0985347	-1.960551643	3.08E-14	3.77E-13
ENSG00000105327	BBC3	276.6881686	535.4873219	-0.952768686	3.23E-14	3.95E-13
ENSG00000065882	TBC1D1	451.2778998	805.8642044	-0.835764954	3.24E-14	3.95E-13
ENSG00000219438	FAM19A5	382.8716749	167.3322471	1.19372382	3.30E-14	4.03E-13
ENSG00000227036	LINC00511	135.4525819	20.17584552	2.772392201	3.37E-14	4.10E-13
ENSG00000173540	GMPPB	695.1327924	364.6819092	0.929634327	3.54E-14	4.31E-13
ENSG00000130733	YIPF2	856.5159532	502.5605689	0.768836351	4.31E-14	5.23E-13
ENSG00000157680	DGKI	602.6107068	322.501435	0.899179855	4.40E-14	5.33E-13
ENSG00000105825	TFPI2	372.882939	168.0158486	1.152219469	4.55E-14	5.52E-13
ENSG00000267095	AC025048.1	69.15123835	5.013749272	3.811603348	5.53E-14	6.67E-13
ENSG00000178882	RFLNA	226.6288892	75.60555635	1.580847249	5.72E-14	6.89E-13
ENSG00000177694	NAALADL2	327.0368922	624.2720447	-0.933426279	5.78E-14	6.96E-13
ENSG00000088543	C3orf18	301.6069352	569.2967747	-0.916439604	5.81E-14	6.99E-13
ENSG00000177042	TMEM80	547.8445135	926.6017296	-0.758443742	6.08E-14	7.30E-13
ENSG00000101849	TBL1X	456.0586598	813.7468424	-0.83419042	6.51E-14	7.81E-13
ENSG00000103064	SLC7A6	766.7664698	440.6470019	0.79866434	7.19E-14	8.62E-13
ENSG00000167378	IRGQ	747.2564929	401.7723312	0.898697107	8.36E-14	9.98E-13
ENSG00000117586	TNFSF4	291.3773081	117.7060496	1.304630803	9.42E-14	1.12E-12
ENSG00000137767	SQOR	292.6935777	588.6699129	-1.009417798	9.84E-14	1.17E-12

ENSG00000133424	LARGE1	754.690496	431.4133632	0.807164864	1.01E-13	1.21E-12
ENSG00000149289	ZC3H12C	184.4303083	422.993033	-1.198193729	1.02E-13	1.21E-12
ENSG00000104738	MCM4	351.5278887	641.4125339	-0.867081169	1.12E-13	1.33E-12
ENSG00000168743	NPNT	67.66795032	2.693991338	4.644754866	1.17E-13	1.39E-12
ENSG00000131759	RARA	670.5926586	360.3772869	0.893414515	1.17E-13	1.39E-12
ENSG00000184465	WDR27	257.292498	509.7053529	-0.987176156	1.19E-13	1.41E-12
ENSG00000156486	KCNS2	4.730066925	85.89010539	-4.166124763	1.21E-13	1.43E-12
ENSG00000233016	SNHG7	341.6312536	638.4219259	-0.900669862	1.24E-13	1.46E-12
ENSG00000145819	ARHGAP26	130.9764775	335.4427971	-1.357568731	1.35E-13	1.60E-12
ENSG00000185432	METTL7A	349.511507	630.3959348	-0.849780504	1.39E-13	1.64E-12
ENSG00000117643	MAN1C1	245.4666537	506.0263802	-1.043618252	1.42E-13	1.67E-12
ENSG00000272825	AL844908.1	66.37713121	3.789876327	4.129568515	1.57E-13	1.85E-12
ENSG00000069667	RORA	314.8520209	583.5951791	-0.889082448	1.69E-13	1.98E-12
ENSG00000138759	FRAS1	1101.422558	654.9812524	0.75161883	1.71E-13	2.01E-12
ENSG00000184602	SNN	355.4061641	646.0020964	-0.862477552	1.78E-13	2.08E-12
ENSG00000139372	TDG	592.2372143	305.6586263	0.957885165	1.82E-13	2.13E-12
ENSG00000105514	RAB3D	124.2888417	312.0683512	-1.326467749	1.97E-13	2.31E-12
ENSG00000233695	GAS6-AS1	456.828079	190.1391878	1.260886187	2.05E-13	2.39E-12
ENSG00000138347	MYPN	4.464413697	83.32215587	-4.236862095	2.44E-13	2.84E-12
ENSG00000128165	ADM2	100.8758777	12.37125425	3.064438961	2.67E-13	3.10E-12
ENSG00000187720	THSD4	272.4431796	523.9406234	-0.943101391	2.78E-13	3.22E-12
ENSG00000183044	ABAT	344.0570263	155.9753693	1.142962534	3.07E-13	3.56E-12
ENSG00000131015	ULBP2	180.7756433	54.988881	1.719464396	3.07E-13	3.56E-12
ENSG00000183668	PSG9	53.25635953	178.5959397	-1.745895511	3.33E-13	3.86E-12
ENSG00000143622	RIT1	674.4995996	384.925426	0.809428658	3.59E-13	4.15E-12
ENSG00000175213	ZNF408	323.0423185	130.7799246	1.30666332	3.64E-13	4.20E-12
ENSG00000089639	GMIP	380.1893596	177.3266739	1.098781304	3.95E-13	4.56E-12
ENSG00000139508	SLC46A3	352.9397529	162.1356015	1.121744463	4.07E-13	4.69E-12
ENSG00000106003	LFNG	285.4644768	106.1254196	1.424742749	4.62E-13	5.32E-12
ENSG00000123496	IL13RA2	6.673570181	95.52001779	-3.818496829	4.65E-13	5.34E-12
ENSG00000138772	ANXA3	66.30923145	210.9430702	-1.666302955	4.73E-13	5.43E-12
ENSG00000176697	BDNF	332.9634837	144.7791328	1.20300104	4.81E-13	5.53E-12
ENSG00000121904	CSMD2	221.4234697	76.01264644	1.539130567	5.20E-13	5.96E-12
ENSG00000228526	MIR34AHG	254.7363107	499.1250237	-0.969577901	5.25E-13	6.02E-12
ENSG00000228221	LINC00578	111.5579599	14.9355265	2.934153118	5.26E-13	6.02E-12
ENSG00000111912	NCOA7	253.1629641	546.9215665	-1.111804198	5.44E-13	6.22E-12
ENSG00000204291	COL15A1	394.17489	188.9890278	1.062120983	6.36E-13	7.24E-12
ENSG00000101115	SALL4	79.30666014	8.428747393	3.237058237	6.38E-13	7.27E-12
ENSG00000054967	RELT	152.7213169	42.12211512	1.856120467	6.43E-13	7.32E-12
ENSG00000168646	AXIN2	168.640343	373.9010658	-1.149172965	6.83E-13	7.75E-12
ENSG00000104332	SFRP1	276.2256052	523.4617665	-0.923188238	6.96E-13	7.90E-12
ENSG00000185100	ADSSL1	22.97309322	122.7489901	-2.423835187	7.07E-13	8.02E-12
ENSG00000167642	SPINT2	134.9135154	31.00759051	2.110421564	8.06E-13	9.13E-12
ENSG00000134901	KDELC1	490.5289455	251.8132075	0.96433601	9.31E-13	1.05E-11
ENSG00000066468	FGFR2	65.80385942	4.040443844	4.082962615	1.09E-12	1.24E-11
ENSG00000125848	FLRT3	13.11245055	96.12688564	-2.867417695	1.13E-12	1.28E-11
ENSG00000139364	TMEM132B	36.65194905	151.8454173	-2.048036312	1.37E-12	1.54E-11
ENSG00000042832	TG	111.88771	24.18321767	2.21804985	1.60E-12	1.80E-11
ENSG00000087008	ACOX3	604.8962391	306.6269879	0.978505863	1.76E-12	1.97E-11
ENSG00000154721	JAM2	207.4135098	53.64481616	1.948017398	1.83E-12	2.05E-11
ENSG00000147862	NFIB	127.7834824	303.895365	-1.248401195	1.99E-12	2.22E-11
ENSG00000067221	STOML1	489.0935957	256.399331	0.930090788	2.01E-12	2.25E-11
ENSG00000167641	PPP1R14A	133.2905475	20.51808182	2.69329812	2.23E-12	2.49E-11
ENSG00000181195	PENK	22.87103857	117.9174808	-2.366403154	2.25E-12	2.51E-11
ENSG00000159479	MED8	572.3556728	311.9423212	0.876487955	2.30E-12	2.56E-11
ENSG00000184304	PRKD1	695.3215325	392.3763428	0.825504436	2.31E-12	2.57E-11
ENSG00000187837	HIST1H1C	307.6931946	131.6598416	1.223561349	2.33E-12	2.59E-11
ENSG00000154065	ANKRD29	50.00506233	167.4169913	-1.742993006	2.39E-12	2.65E-11
ENSG00000184500	PROS1	444.7321643	767.5030014	-0.785574113	2.49E-12	2.77E-11
ENSG00000130702	LAMA5	58.93947973	190.3160191	-1.693627016	2.60E-12	2.89E-11
ENSG00000161642	ZNF385A	107.0651357	285.5831054	-1.416840835	2.68E-12	2.97E-11
ENSG00000157570	TSPAN18	177.3722602	57.9226348	1.61578371	2.97E-12	3.28E-11
ENSG00000100003	SEC14L2	229.0289055	83.02251823	1.45750871	2.98E-12	3.29E-11
ENSG00000166603	MC4R	16.76890266	99.7288871	-2.570443648	3.14E-12	3.46E-11
ENSG00000184254	ALDH1A3	37.42754294	148.5629558	-1.993881444	3.38E-12	3.71E-11
ENSG00000148737	TCF7L2	230.0973132	478.1401919	-1.054704	3.57E-12	3.92E-11

ENSG00000049246	PER3	363.3691491	632.9193743	-0.801018342	3.58E-12	3.93E-11
ENSG00000198483	ANKRD35	40.51720686	153.796147	-1.927288992	3.83E-12	4.20E-11
ENSG00000178498	DTX3	495.2143663	265.4954547	0.899885221	3.84E-12	4.21E-11
ENSG00000120738	EGR1	443.3792669	1523.396921	-1.780833354	3.96E-12	4.34E-11
ENSG00000233682	AL356417.2	64.27194434	5.574556215	3.551305763	4.23E-12	4.62E-11
ENSG00000004864	SLC25A13	406.1617795	197.8644199	1.039228908	4.25E-12	4.64E-11
ENSG00000152749	GPR180	696.8561288	406.9802231	0.778169769	4.51E-12	4.92E-11
ENSG00000088325	TPX2	307.756321	565.0064197	-0.875833198	4.54E-12	4.94E-11
ENSG00000173846	PLK3	926.8275728	549.8836744	0.75039761	4.55E-12	4.95E-11
ENSG00000125445	MRPS7	611.6781694	339.3260616	0.850541349	4.55E-12	4.95E-11
ENSG00000210196	MT-TP	170.237326	366.7335851	-1.105679989	4.63E-12	5.04E-11
ENSG00000273270	AC090114.2	275.3913262	118.7902583	1.213823607	4.86E-12	5.28E-11
ENSG00000198721	ECI2	312.3408358	553.3736753	-0.82531115	4.96E-12	5.39E-11
ENSG00000148019	CEP78	719.668583	402.7637842	0.840149073	4.98E-12	5.40E-11
ENSG00000148335	NTMT1	345.5314825	161.2388262	1.099181952	5.38E-12	5.83E-11
ENSG00000123610	TNFAIP6	184.6284446	60.65380489	1.612084585	5.59E-12	6.05E-11
ENSG00000140832	MARVELD3	59.10810731	3.677457018	3.977665866	5.62E-12	6.08E-11
ENSG00000115159	GPD2	259.9153353	504.9938327	-0.958773202	5.68E-12	6.14E-11
ENSG00000120658	ENOX1	70.57137337	212.7725096	-1.593092083	5.87E-12	6.34E-11
ENSG00000143028	SYPL2	38.67575825	149.6878054	-1.95671017	6.21E-12	6.69E-11
ENSG00000011638	TMEM159	344.4389779	614.2319985	-0.834731736	6.22E-12	6.70E-11
ENSG00000153443	UBALD1	457.5473094	245.7982628	0.896889149	6.24E-12	6.72E-11
ENSG00000213366	GSTM2	266.1623078	495.2143105	-0.895800352	6.48E-12	6.97E-11
ENSG00000006118	TMEM132A	260.427752	105.7187477	1.304600049	6.83E-12	7.34E-11
ENSG00000166922	SCG5	169.8349526	49.34363281	1.778377722	6.85E-12	7.35E-11
ENSG00000169239	CA5B	293.1700019	535.9981755	-0.871640732	7.49E-12	8.03E-11
ENSG00000259345	AC013652.1	190.6117559	65.6571789	1.538646225	7.90E-12	8.45E-11
ENSG00000138134	STAMBPL1	89.52388099	234.3588014	-1.39068093	8.04E-12	8.59E-11
ENSG00000100307	CBX7	213.0906571	431.3872045	-1.017800338	8.10E-12	8.66E-11
ENSG00000115902	SLC1A4	666.8194478	368.823991	0.854847255	8.13E-12	8.68E-11
ENSG00000163874	ZC3H12A	371.1184539	158.8801465	1.219963322	9.20E-12	9.81E-11
ENSG00000196227	FAM217B	161.3371139	356.1429039	-1.139694337	9.37E-12	9.99E-11
ENSG00000139174	PRICKLE1	172.4556972	56.59109441	1.607914236	9.81E-12	1.04E-10
ENSG00000121653	MAPK8IP1	56.20741537	185.1154815	-1.717951597	9.95E-12	1.06E-10
ENSG00000090013	BLVRB	271.1249211	518.9292613	-0.938374238	1.07E-11	1.13E-10
ENSG00000156521	TYSND1	82.35282667	229.8464137	-1.484257605	1.08E-11	1.14E-10
ENSG00000137094	DNAJB5	386.4777867	172.1663475	1.168062629	1.13E-11	1.19E-10
ENSG00000231453	LINC01305	246.9018254	462.13039	-0.904646403	1.20E-11	1.26E-10
ENSG00000114520	SNX4	509.6668507	284.7423246	0.838792701	1.22E-11	1.28E-10
ENSG00000266094	RASSF5	14.83512481	93.736424	-2.661064188	1.23E-11	1.30E-10
ENSG00000164309	CMYA5	218.8596229	443.2516886	-1.018758116	1.27E-11	1.34E-10
ENSG00000167772	ANGPTL4	4.757788541	68.25021252	-3.831442016	1.41E-11	1.48E-10
ENSG00000170044	ZPLD1	54.8150523	0	8.5092189	1.42E-11	1.49E-10
ENSG00000245812	LINC02202	5.575475546	70.0690502	-3.648843747	1.46E-11	1.53E-10
ENSG00000198624	CCDC69	27.61820811	121.0451624	-2.132522014	1.47E-11	1.54E-10
ENSG00000157111	TMEM171	78.64093134	211.6720761	-1.428831324	1.47E-11	1.54E-10
ENSG00000143554	SLC27A3	161.4942288	367.6824745	-1.185793024	1.53E-11	1.60E-10
ENSG00000138411	HECW2	168.3832931	356.8081308	-1.082847473	1.61E-11	1.68E-10
ENSG00000172731	LRRC20	25.62250342	116.7249601	-2.185268914	1.69E-11	1.77E-10
ENSG00000155099	PIP4P2	222.9892071	440.7637437	-0.981019212	1.71E-11	1.78E-10
ENSG00000064763	FAR2	52.96298468	170.2975564	-1.684041068	1.91E-11	1.99E-10
ENSG00000115041	KCNIP3	78.3687945	214.0932333	-1.451936344	2.03E-11	2.11E-10
ENSG00000137522	RNF121	508.1339646	272.7472381	0.896183711	2.20E-11	2.28E-10
ENSG00000088756	ARHGAP28	252.3257257	101.3297994	1.320452549	2.33E-11	2.41E-10
ENSG00000183049	CAMK1D	40.2146173	153.7267446	-1.932801095	2.35E-11	2.43E-10
ENSG00000118242	MREG	89.27964389	238.9014551	-1.418667169	2.39E-11	2.47E-10
ENSG00000072041	SLC6A15	24.37752991	115.8255939	-2.245688399	2.62E-11	2.70E-10
ENSG00000213047	DENND1B	469.6117469	244.5073636	0.94452842	2.69E-11	2.77E-10
ENSG00000122678	POLM	640.2990556	338.3065033	0.918337041	2.72E-11	2.80E-10
ENSG00000197885	NKIRAS1	307.5558364	546.0981091	-0.827307691	2.72E-11	2.80E-10
ENSG00000279821	AC145098.2	108.1914627	25.26462034	2.093916293	3.07E-11	3.15E-10
ENSG00000110076	NRXN2	42.46484552	145.6499525	-1.778233395	3.18E-11	3.26E-10
ENSG00000281207	SLFNL1-AS1	65.11321757	7.924150104	3.045756676	3.22E-11	3.29E-10
ENSG00000159714	ZDHHC1	249.073713	485.959073	-0.965146797	3.31E-11	3.39E-10
ENSG00000128590	DNAJB9	616.8503005	346.428044	0.8311828	3.54E-11	3.62E-10
ENSG00000141449	GREB1L	503.1217803	283.3431132	0.829345571	3.64E-11	3.72E-10

ENSG00000160886	LY6K	0	66.18814989	-8.224724872	4.09E-11	4.17E-10
ENSG00000152413	HOMER1	185.5348865	65.21246853	1.511681677	4.30E-11	4.38E-10
ENSG00000169994	MYO7B	95.4537143	16.91954263	2.482466899	4.75E-11	4.81E-10
ENSG00000144655	CSRNP1	411.2942198	204.8012236	1.00261872	4.77E-11	4.83E-10
ENSG00000169515	CCDC8	573.8377885	315.440762	0.861506112	5.36E-11	5.43E-10
ENSG00000006453	BAIAP2L1	438.450093	228.1564123	0.946220879	5.39E-11	5.45E-10
ENSG00000136379	ABHD17C	116.9674886	264.5794453	-1.177865269	5.48E-11	5.53E-10
ENSG00000147852	VLDLR	334.8931262	159.6597391	1.068527685	5.60E-11	5.66E-10
ENSG00000152217	SETBP1	478.4281055	257.4415855	0.893748903	5.84E-11	5.89E-10
ENSG00000187098	MITF	42.71339613	146.3549608	-1.774014882	5.97E-11	6.02E-10
ENSG00000163050	COQ8A	178.2348992	364.1589493	-1.030081495	5.99E-11	6.04E-10
ENSG00000149948	HMGA2	142.7570372	326.7425049	-1.196847438	6.30E-11	6.33E-10
ENSG00000183715	OPCML	751.4865226	440.3799948	0.773963149	6.42E-11	6.45E-10
ENSG00000168334	XIRP1	59.77332542	0.925524897	6.177013201	6.63E-11	6.66E-10
ENSG00000146918	NCAPG2	262.1147664	482.9809779	-0.882667609	7.01E-11	7.02E-10
ENSG00000184428	TOP1MT	132.8645375	307.4993241	-1.211667093	7.67E-11	7.68E-10
ENSG00000163393	SLC22A15	505.965907	280.2167326	0.852953749	7.70E-11	7.70E-10
ENSG00000196139	AKR1C3	441.0339245	785.775288	-0.833672995	7.77E-11	7.77E-10
ENSG00000171492	LRRC8D	243.6770236	458.9367683	-0.913122912	8.03E-11	8.02E-10
ENSG00000172059	KLF11	162.1551838	333.6042715	-1.040867202	8.05E-11	8.04E-10
ENSG00000066382	MPPED2	81.98741667	13.84310377	2.579454565	8.09E-11	8.07E-10
ENSG00000100065	CARD10	14.19617369	95.41712544	-2.758610466	8.26E-11	8.24E-10
ENSG00000188917	TRMT2B	280.4363115	548.5880006	-0.968949068	8.31E-11	8.28E-10
ENSG00000165029	ABCA1	650.9719966	380.5319291	0.775372255	8.85E-11	8.80E-10
ENSG00000122121	XPNPEP2	6.77383764	72.60910991	-3.417718244	9.31E-11	9.25E-10
ENSG00000151136	BTBD11	57.59334517	5.63076587	3.38610526	9.63E-11	9.56E-10
ENSG00000117155	SSX2IP	243.0357243	461.7931088	-0.92551797	9.72E-11	9.65E-10
ENSG00000169594	BNC1	203.3704097	410.9486621	-1.013499439	1.00E-10	9.93E-10
ENSG00000162618	ADGRL4	403.873822	196.2475094	1.044241168	1.04E-10	1.03E-09
ENSG00000138650	PCDH10	198.9018103	76.04896561	1.392970405	1.14E-10	1.12E-09
ENSG00000177791	MYOZ1	52.14402118	1.149718715	5.572848568	1.19E-10	1.18E-09
ENSG00000154358	OBSCN	121.797262	279.0399954	-1.196411203	1.27E-10	1.25E-09
ENSG00000138182	KIF20B	160.2320249	326.5135593	-1.027668551	1.28E-10	1.26E-09
ENSG00000117266	CDK18	0	59.28228002	-8.065655851	1.34E-10	1.32E-09
ENSG00000137812	KNL1	116.3540387	288.4904738	-1.306843429	1.41E-10	1.39E-09
ENSG00000183337	BCOR	527.8070566	295.7604286	0.837063499	1.43E-10	1.40E-09
ENSG00000197093	GAL3ST4	134.8096735	314.9757081	-1.222010981	1.47E-10	1.44E-09
ENSG00000149571	KIRREL3	348.1385948	171.996417	1.013778308	1.58E-10	1.55E-09
ENSG00000175866	BAIAP2	271.3218313	480.1624609	-0.824020114	1.76E-10	1.73E-09
ENSG00000255874	LINC00346	70.39028928	197.6644389	-1.486580176	2.02E-10	1.98E-09
ENSG00000164104	HMGB2	311.5155935	546.2100864	-0.811404475	2.03E-10	1.98E-09
ENSG00000156876	SASS6	187.9217072	67.82279019	1.473837756	2.05E-10	2.00E-09
ENSG00000155330	C16orf87	198.0375118	78.15620609	1.342637667	2.07E-10	2.02E-09
ENSG00000144843	ADPRH	288.469239	136.2433628	1.084919511	2.13E-10	2.08E-09
ENSG00000083123	BCKDHB	367.4974835	630.4156337	-0.778959914	2.15E-10	2.09E-09
ENSG00000082146	STRADB	269.157882	494.5211935	-0.879445834	2.18E-10	2.13E-09
ENSG00000116729	WLS	203.0937545	389.5653834	-0.939813833	2.20E-10	2.14E-09
ENSG00000064652	SNX24	308.2806835	146.4150627	1.078071464	2.22E-10	2.16E-09
ENSG00000110042	DTX4	133.1757038	32.98100508	2.027976518	2.29E-10	2.23E-09
ENSG00000138449	SLC40A1	43.50698623	149.7427139	-1.784935942	2.49E-10	2.42E-09
ENSG00000136490	LIMD2	161.1111281	52.87126525	1.60943714	2.56E-10	2.48E-09
ENSG00000155085	AK9	45.72732274	164.1358194	-1.845054651	2.84E-10	2.74E-09
ENSG00000047936	ROS1	48.70699379	2.245603704	4.476542493	2.84E-10	2.75E-09
ENSG00000138311	ZNF365	78.93844158	11.55620252	2.782629254	2.89E-10	2.78E-09
ENSG00000102100	SLC35A2	488.8806043	278.9805503	0.809962068	3.02E-10	2.91E-09
ENSG00000113594	LIFR	285.6798164	495.0480577	-0.792876787	3.16E-10	3.04E-09
ENSG00000116285	ERRFI1	130.0846356	288.1158219	-1.148349481	3.25E-10	3.12E-09
ENSG00000282057	AC092807.3	265.0036132	108.2311324	1.296954151	3.37E-10	3.23E-09
ENSG00000215790	SLC35E2A	140.8284665	310.4233244	-1.138551368	3.42E-10	3.28E-09
ENSG00000100599	RIN3	339.8554069	572.8419215	-0.754370274	3.46E-10	3.31E-09
ENSG00000164066	INTU	435.8037701	229.8252104	0.924848108	3.82E-10	3.65E-09
ENSG00000197646	PDCD1LG2	398.0845892	213.1800295	0.899543164	3.91E-10	3.73E-09
ENSG00000159217	IGF2BP1	356.1041328	180.4335938	0.98194442	4.17E-10	3.97E-09
ENSG00000186812	ZNF397	301.5008729	521.12059	-0.788890703	4.52E-10	4.29E-09
ENSG00000055813	CCDC85A	347.6600004	181.5724957	0.938993844	5.22E-10	4.94E-09
ENSG00000232324	AC008440.3	2.342174551	57.94425664	-4.598398393	5.33E-10	5.04E-09

ENSG00000099998	GGT5	51.24641113	3.04854904	4.091118835	5.38E-10	5.08E-09
ENSG00000153721	CNKSR3	55.09780814	162.2404515	-1.55525038	5.82E-10	5.48E-09
ENSG00000211445	GPX3	22.91675639	105.4463436	-2.201481708	5.90E-10	5.55E-09
ENSG00000214530	STARD10	365.170087	182.3951165	0.999205601	6.23E-10	5.86E-09
ENSG00000050767	COL23A1	0	49.97037987	-7.81980286	6.30E-10	5.92E-09
ENSG00000177570	SAMD12	53.55767259	172.3137845	-1.684443073	6.55E-10	6.15E-09
ENSG00000123096	SSPN	524.6539337	309.2794436	0.764029402	6.89E-10	6.45E-09
ENSG00000138135	CH25H	120.5823583	35.14575656	1.784928241	6.97E-10	6.53E-09
ENSG00000196639	HRH1	147.221068	321.868647	-1.12894508	7.01E-10	6.56E-09
ENSG00000169026	SLC49A3	524.7537443	277.9525511	0.914143683	7.22E-10	6.74E-09
ENSG00000130787	HIP1R	265.7083981	482.7922547	-0.863927856	7.27E-10	6.79E-09
ENSG00000262454	MIR193BHG	382.9827898	206.9834783	0.887962922	7.92E-10	7.37E-09
ENSG00000148219	ASTN2	157.5594114	51.19771507	1.618358723	8.01E-10	7.44E-09
ENSG00000106483	SFRP4	123.7468659	32.80178585	1.905287311	8.09E-10	7.52E-09
ENSG00000151014	NOCT	151.4837709	49.14343676	1.62209979	9.75E-10	9.01E-09
ENSG00000154127	UBASH3B	200.0123614	380.0286566	-0.925831067	1.01E-09	9.31E-09
ENSG00000254510	AP001107.5	59.31545841	7.088992883	3.096694635	1.23E-09	1.13E-08
ENSG00000171385	KCND3	234.6095882	102.8390542	1.188543667	1.25E-09	1.15E-08
ENSG00000166938	DIS3L	331.6583706	557.9740782	-0.750491289	1.27E-09	1.16E-08
ENSG00000172296	SPTLC3	12.37468659	79.20173135	-2.677069413	1.27E-09	1.17E-08
ENSG00000112183	RBM24	189.2615362	74.69472878	1.345112184	1.29E-09	1.18E-08
ENSG00000189077	TMEM120A	341.4533339	578.6735404	-0.760791061	1.32E-09	1.21E-08
ENSG00000141401	IMPA2	150.8632764	312.8375686	-1.050416669	1.33E-09	1.22E-08
ENSG00000227925	LINC01655	59.75806033	6.75043362	3.116788928	1.33E-09	1.22E-08
ENSG00000145040	UCN2	62.02679545	9.730881624	2.670934283	1.40E-09	1.28E-08
ENSG00000129071	MBD4	547.0081651	316.6188003	0.786112512	1.41E-09	1.29E-08
ENSG00000103044	HAS3	108.9653972	27.75495337	1.985717737	1.42E-09	1.30E-08
ENSG00000204388	HSPA1B	253.3297039	443.8243987	-0.80832805	1.43E-09	1.30E-08
ENSG00000090376	IRAK3	239.7829725	426.6480206	-0.830953193	1.44E-09	1.32E-08
ENSG00000090776	EFNB1	169.9509455	348.8658211	-1.035355176	1.54E-09	1.40E-08
ENSG00000134201	GSTM5	40.7852083	142.8660235	-1.806158376	1.59E-09	1.44E-08
ENSG00000152953	STK32B	287.1848531	488.4312352	-0.766169447	1.59E-09	1.44E-08
ENSG00000242611	AC093627.6	8.438720455	64.08242552	-2.927168685	1.67E-09	1.52E-08
ENSG00000104524	PYCR3	263.4748896	126.03686	1.062468092	1.72E-09	1.56E-08
ENSG00000223764	LINC02593	180.9590253	67.85651819	1.411606274	1.74E-09	1.58E-08
ENSG00000132334	PTPRE	322.9748016	158.163677	1.03345676	1.82E-09	1.64E-08
ENSG00000188112	C6orf132	211.6617406	402.1292761	-0.926761119	1.87E-09	1.69E-08
ENSG00000127329	PTPRB	236.3930672	103.3514241	1.19341281	1.88E-09	1.69E-08
ENSG00000185760	KCNQ5	110.1918638	244.0838451	-1.146384779	1.91E-09	1.72E-08
ENSG00000198523	PLN	167.1888616	4.150487224	5.368989494	1.92E-09	1.73E-08
ENSG00000167977	KCTD5	560.6176158	320.9805036	0.802331916	1.95E-09	1.76E-08
ENSG00000171522	PTGER4	87.98425614	19.89631337	2.133564885	1.99E-09	1.80E-08
ENSG00000110799	VWF	8.766300521	70.51202967	-3.002292086	2.05E-09	1.84E-08
ENSG00000163637	PRICKLE2	439.7767767	246.5274722	0.837768263	2.20E-09	1.97E-08
ENSG00000030110	BAK1	394.3929105	220.2608083	0.839775496	2.42E-09	2.17E-08
ENSG00000146592	CREB5	11.60595921	73.55949238	-2.660601077	2.51E-09	2.24E-08
ENSG00000099957	P2RX6	10.68621756	73.22871744	-2.765872835	2.54E-09	2.27E-08
ENSG00000143256	PFDN2	448.8863053	262.2642244	0.776310143	2.61E-09	2.33E-08
ENSG00000242193	CRYZL2P	145.074732	312.6265675	-1.107940757	2.79E-09	2.49E-08
ENSG00000158089	GALNT14	16.32361994	89.82116239	-2.456104301	2.88E-09	2.56E-08
ENSG00000165030	NFIL3	181.2113788	355.2186684	-0.969969389	2.94E-09	2.61E-08
ENSG00000104998	IL27RA	96.41108109	21.29486847	2.183961959	2.98E-09	2.64E-08
ENSG00000102575	ACP5	49.88120865	164.4207597	-1.72021009	3.05E-09	2.71E-08
ENSG00000110203	FOLR3	25.59154	99.62619825	-1.959830855	3.32E-09	2.93E-08
ENSG00000117600	PLPPR4	28.68768753	118.6981597	-2.043086183	3.53E-09	3.11E-08
ENSG00000172985	SH3RF3	382.8081389	212.7392114	0.84813517	3.83E-09	3.36E-08
ENSG00000134470	IL15RA	33.77846804	117.5421728	-1.799810301	3.86E-09	3.39E-08
ENSG00000179715	PCED1B	228.1131212	97.00419999	1.228078331	3.87E-09	3.39E-08
ENSG00000233117	LINC00702	261.8904908	450.6466106	-0.782787012	4.05E-09	3.54E-08
ENSG00000168096	ANKS3	527.0155591	303.6087046	0.792323222	4.11E-09	3.60E-08
ENSG00000270562	AC097634.1	44.56674025	3.441371821	3.743748685	4.13E-09	3.61E-08
ENSG00000105671	DDX49	486.2913841	287.7915184	0.755824891	4.16E-09	3.64E-08
ENSG00000259207	ITGB3	64.14293389	186.5213912	-1.543462729	4.23E-09	3.69E-08
ENSG00000253304	TMEM200B	287.5433463	503.7531128	-0.809552993	4.27E-09	3.73E-08
ENSG00000145779	TNFAIP8	297.6045742	507.4433097	-0.769631283	4.42E-09	3.85E-08
ENSG00000166881	NEMP1	285.0870938	483.4092603	-0.760375522	4.52E-09	3.93E-08

ENSG00000221818	EBF2	7.263944579	59.40939643	-3.038819311	4.61E-09	4.01E-08
ENSG00000233521	LINC01638	40.46824724	1.514436668	4.86008083	4.66E-09	4.05E-08
ENSG00000162746	FCRLB	1.650128413	52.10788633	-5.014572943	5.01E-09	4.34E-08
ENSG00000142178	SIK1	50.52720439	3.304954742	3.884004538	5.25E-09	4.54E-08
ENSG00000114670	NEK11	155.2902934	303.6210145	-0.9678298	5.32E-09	4.60E-08
ENSG00000285517	LINC00941	56.44953271	158.7925965	-1.493304108	5.34E-09	4.61E-08
ENSG00000183508	TENT5C	2.39437598	47.80541338	-4.315507876	5.66E-09	4.89E-08
ENSG00000139318	DUSP6	87.45491498	219.2492261	-1.324322777	5.71E-09	4.93E-08
ENSG00000117620	SLC35A3	695.4350499	401.6912407	0.795691823	5.88E-09	5.07E-08
ENSG00000172548	NIPAL4	42.22183458	1.937095405	4.469628226	6.12E-09	5.26E-08
ENSG00000042781	USH2A	34.40680015	116.5766514	-1.761236635	6.75E-09	5.79E-08
ENSG00000168078	PBK	59.0942465	159.8283798	-1.433985831	6.76E-09	5.79E-08
ENSG00000179532	DNHD1	261.5240091	448.9049479	-0.778281384	6.82E-09	5.84E-08
ENSG00000177822	AC098864.1	181.0902562	62.01430992	1.557945483	6.84E-09	5.86E-08
ENSG00000244586	WNT5A-AS1	214.5784131	89.09021037	1.270320178	6.84E-09	5.86E-08
ENSG00000164237	CMBL	255.8583744	454.7292003	-0.828449698	7.04E-09	6.02E-08
ENSG00000130518	IQCN	37.38920232	125.3374749	-1.750295075	7.31E-09	6.25E-08
ENSG00000179403	VWA1	27.48932544	104.7659893	-1.92733661	7.33E-09	6.27E-08
ENSG00000187608	ISG15	733.5296218	405.3696153	0.85478724	7.43E-09	6.35E-08
ENSG00000261625	AP003071.4	18.58676505	95.7115697	-2.35504827	7.57E-09	6.46E-08
ENSG00000150779	TIMM8B	427.3440668	220.0013819	0.956905453	7.64E-09	6.51E-08
ENSG00000158560	DYNC1I1	85.44300128	19.82410527	2.10932758	7.92E-09	6.74E-08
ENSG00000139517	LNX2	319.9798655	169.7802077	0.912001598	7.93E-09	6.74E-08
ENSG00000002919	SNX11	403.5384315	225.2680631	0.844686729	8.00E-09	6.80E-08
ENSG00000174718	KIAA1551	274.345994	466.2543003	-0.765578484	8.38E-09	7.12E-08
ENSG00000113319	RASGRF2	39.21444191	2.70415159	3.856863949	8.42E-09	7.15E-08
ENSG00000088970	KIZ	218.1523891	388.1713769	-0.83212539	8.70E-09	7.37E-08
ENSG00000152582	SPEF2	82.31213784	196.5199133	-1.257347961	9.19E-09	7.77E-08
ENSG00000177303	CASKIN2	440.4445211	260.9435008	0.756129571	9.25E-09	7.81E-08
ENSG00000106526	ACTR3C	39.68979445	1.234033196	5.152980884	9.62E-09	8.12E-08
ENSG00000067836	ROGDI	247.9957846	116.1948604	1.092564599	9.80E-09	8.26E-08
ENSG00000166035	LIPC	27.72998817	108.6077535	-1.969771018	1.02E-08	8.60E-08
ENSG00000134057	CCNB1	210.4333087	394.1011123	-0.905533049	1.02E-08	8.62E-08
ENSG00000006704	GTF2IRD1	478.3412153	276.1667848	0.790260284	1.14E-08	9.53E-08
ENSG00000135437	RDH5	11.69325946	70.03662354	-2.587307807	1.15E-08	9.61E-08
ENSG00000183624	HMCES	176.5380316	333.775718	-0.920406471	1.25E-08	1.04E-07
ENSG00000179546	HTR1D	29.10524863	0	7.596591942	1.27E-08	1.06E-07
ENSG00000119922	IFIT2	89.22001493	234.1054286	-1.387739145	1.35E-08	1.12E-07
ENSG00000163795	ZNF513	393.0363114	215.9952991	0.861603227	1.37E-08	1.14E-07
ENSG00000136630	HLX	178.8459506	71.17098861	1.334916324	1.46E-08	1.21E-07
ENSG00000243244	STON1	278.1439131	480.2928133	-0.786862507	1.46E-08	1.21E-07
ENSG00000042062	RIPOR3	39.08270035	131.774207	-1.756185372	1.51E-08	1.25E-07
ENSG00000143067	ZNF697	207.8387807	88.93217145	1.224047782	1.67E-08	1.38E-07
ENSG00000131620	ANO1	48.90602377	2.702420462	4.263040034	1.73E-08	1.43E-07
ENSG00000137975	CLCA2	21.65143847	90.70581977	-2.064275033	1.75E-08	1.44E-07
ENSG00000141664	ZCCHC2	196.9685431	361.2407641	-0.874206066	1.77E-08	1.46E-07
ENSG00000104888	SLC17A7	11.48031835	73.06960393	-2.661307844	1.82E-08	1.50E-07
ENSG00000164107	HAND2	100.2831551	219.6803257	-1.130006691	1.84E-08	1.51E-07
ENSG00000244649	LINC02086	57.85759412	8.609267737	2.743892305	1.84E-08	1.52E-07
ENSG00000153094	BCL2L11	47.57972293	136.2816279	-1.515815649	1.92E-08	1.58E-07
ENSG00000241399	CD302	81.0325762	191.857701	-1.242663578	2.24E-08	1.83E-07
ENSG00000085117	CD82	168.7659838	323.7227058	-0.939208012	2.24E-08	1.84E-07
ENSG00000222032	AC112721.2	27.92074734	0	7.536521888	2.32E-08	1.90E-07
ENSG00000213397	HAUS7	60.39790504	11.22737372	2.435050858	2.62E-08	2.14E-07
ENSG00000119711	ALDH6A1	209.6346896	370.6804257	-0.821724508	2.96E-08	2.41E-07
ENSG00000170989	S1PR1	69.85198853	195.1449262	-1.485809614	2.97E-08	2.42E-07
ENSG00000259426	AC027237.3	225.8587575	105.7734528	1.093355316	3.00E-08	2.44E-07
ENSG00000077420	APBB1IP	13.68572234	70.7450824	-2.372678761	3.17E-08	2.57E-07
ENSG00000143333	RGS16	174.5514409	12.99605517	3.762627075	3.17E-08	2.57E-07
ENSG00000168917	SLC35G2	79.63372952	184.225416	-1.210431892	3.25E-08	2.63E-07
ENSG00000123600	METTL8	233.3964717	409.6713967	-0.810781614	3.33E-08	2.69E-07
ENSG00000164136	IL15	80.44169112	186.4128524	-1.210641546	3.34E-08	2.70E-07
ENSG00000167363	FN3K	205.7939116	364.0030713	-0.823855449	3.45E-08	2.79E-07
ENSG00000115539	PDCL3	401.8519277	225.4788604	0.830052122	3.54E-08	2.86E-07
ENSG00000140650	PMM2	404.0206002	226.9394754	0.830576739	3.59E-08	2.90E-07
ENSG00000169715	MT1E	231.6491898	99.63440085	1.222783489	3.88E-08	3.12E-07

ENSG00000136367	ZFHX2	134.5227319	45.67612126	1.567625707	4.02E-08	3.23E-07
ENSG00000007372	PAX6	90.59519794	21.44685419	2.090017691	4.03E-08	3.24E-07
ENSG00000099219	ERMP1	180.2919697	332.9195839	-0.883165969	4.13E-08	3.31E-07
ENSG00000125384	PTGER2	9.60611913	64.89249887	-2.750324277	4.21E-08	3.38E-07
ENSG00000130540	SULT4A1	1.213116497	45.6000209	-5.242901609	4.25E-08	3.40E-07
ENSG00000171488	LRRC8C	108.8151487	236.7944292	-1.123720032	4.48E-08	3.58E-07
ENSG00000214357	NEURL1B	30.67690861	111.4753522	-1.858732409	4.50E-08	3.60E-07
ENSG00000099204	ABLIM1	293.1737544	153.8728806	0.933828972	4.56E-08	3.65E-07
ENSG00000173406	DAB1	45.20283247	153.7101018	-1.7671425	4.61E-08	3.69E-07
ENSG00000075643	MOCOS	148.9133399	285.218817	-0.939390292	4.66E-08	3.72E-07
ENSG00000176890	TYMS	73.42394895	181.5686034	-1.304442925	4.79E-08	3.82E-07
ENSG00000131737	KRT34	17.88968991	88.23105515	-2.29948755	4.83E-08	3.85E-07
ENSG00000175746	C15orf54	36.59688872	2.441692694	3.90559411	4.87E-08	3.88E-07
ENSG00000197461	PDGFA	165.2932463	67.91813624	1.279890643	5.13E-08	4.09E-07
ENSG00000144712	CAND2	28.09121244	97.4841432	-1.795120889	5.31E-08	4.22E-07
ENSG00000218891	ZNF579	375.4170989	213.2956963	0.815500668	5.37E-08	4.27E-07
ENSG00000268894	PLCE1-AS1	33.25829128	1.338023382	4.621926172	5.41E-08	4.30E-07
ENSG00000144230	GPR17	138.7726221	49.08658231	1.494772808	5.44E-08	4.32E-07
ENSG00000178038	ALS2CL	84.27828339	197.9013954	-1.22953562	5.87E-08	4.65E-07
ENSG00000110455	ACCS	218.1670158	394.1646878	-0.855717668	6.15E-08	4.86E-07
ENSG00000243444	PALM2	65.89618866	14.24781793	2.215061811	6.34E-08	5.01E-07
ENSG00000172183	ISG20	44.02588649	137.5268959	-1.641832018	6.39E-08	5.05E-07
ENSG00000168961	LGALS9	99.52867142	213.0663091	-1.098440747	6.51E-08	5.13E-07
ENSG00000203930	LINC00632	341.8598931	195.2811283	0.80617591	6.54E-08	5.16E-07
ENSG00000259721	AC090877.2	280.7901082	140.2688945	1.002852168	6.60E-08	5.21E-07
ENSG00000181744	C3orf58	216.5613287	386.5196288	-0.834301442	6.82E-08	5.38E-07
ENSG00000124356	STAMBP	208.7532885	366.3344943	-0.81058103	7.02E-08	5.52E-07
ENSG00000131979	GCH1	52.20270621	7.55943215	2.786070326	7.46E-08	5.86E-07
ENSG00000268812	AC004264.1	24.41441592	0	7.342286648	7.58E-08	5.95E-07
ENSG00000127399	LRRC61	47.9294346	5.594876719	3.122884101	7.71E-08	6.05E-07
ENSG00000139117	CPNE8	284.1124699	482.4973346	-0.761736313	7.72E-08	6.06E-07
ENSG00000198353	HOXC4	271.1405691	466.3736324	-0.7836115	7.96E-08	6.24E-07
ENSG00000242600	MBL1P	14.93304406	72.52652655	-2.286699375	8.15E-08	6.38E-07
ENSG00000166839	ANKDD1A	122.9886298	255.5207151	-1.057906992	8.58E-08	6.71E-07
ENSG00000241684	ADAMTS9-AS2	10.90653587	61.89908467	-2.51002872	8.89E-08	6.95E-07
ENSG00000168303	MPLKIP	361.1631337	196.7694065	0.87939713	9.41E-08	7.35E-07
ENSG00000178726	THBD	2.380515172	41.41623546	-4.107479446	9.63E-08	7.52E-07
ENSG00000090971	NAT14	339.2004249	177.5067647	0.936945476	9.86E-08	7.69E-07
ENSG00000106804	C5	43.60776438	122.3274061	-1.48619861	9.96E-08	7.76E-07
ENSG00000282164	PEG13	38.10299715	125.048201	-1.709127801	1.00E-07	7.81E-07
ENSG00000125618	PAX8	203.552337	355.8888739	-0.805794387	1.01E-07	7.87E-07
ENSG00000121858	TNFSF10	0.776104581	49.67008569	-5.959364425	1.03E-07	8.05E-07
ENSG00000167749	KLK4	23.52977308	0	7.289739537	1.04E-07	8.10E-07
ENSG00000169429	CXCL8	37.62299853	0.617016598	6.095436793	1.09E-07	8.42E-07
ENSG00000178734	LMO7DN	44.99223956	6.175359366	2.857532331	1.10E-07	8.48E-07
ENSG00000151790	TDO2	37.78011351	2.612052786	3.825694748	1.10E-07	8.54E-07
ENSG00000163975	MELTF	96.50111245	216.7292836	-1.168774728	1.13E-07	8.77E-07
ENSG00000137168	PPIL1	287.6764147	155.7937741	0.882219694	1.14E-07	8.83E-07
ENSG00000097096	SYDE2	409.2649716	242.4511507	0.754138171	1.16E-07	8.92E-07
ENSG00000229373	LINC00452	64.01422934	13.33072216	2.278328636	1.24E-07	9.58E-07
ENSG00000132854	KANK4	377.8917808	3.365486463	6.842517677	1.30E-07	9.98E-07
ENSG00000091129	NRCAM	58.03214427	10.19375158	2.534873687	1.31E-07	1.01E-06
ENSG00000128596	CCDC136	212.7020438	369.9613764	-0.800800871	1.35E-07	1.04E-06
ENSG00000273033	LINC02035	140.7046128	268.8421368	-0.934967193	1.39E-07	1.07E-06
ENSG00000103121	CMC2	298.6972569	159.8843744	0.899628566	1.43E-07	1.09E-06
ENSG00000115841	RMDN2	305.147828	152.7787383	0.996294609	1.55E-07	1.19E-06
ENSG00000164220	F2RL2	177.4957309	79.6265395	1.160412925	1.59E-07	1.21E-06
ENSG00000105519	CAPS	70.14766125	165.3229433	-1.239346335	1.79E-07	1.36E-06
ENSG00000140450	ARRDC4	129.3774521	267.7127503	-1.048449129	1.90E-07	1.44E-06
ENSG00000171848	RRM2	70.34311684	165.023939	-1.22849638	1.98E-07	1.50E-06
ENSG00000171051	FPR1	44.74419963	6.241729273	2.844518579	2.03E-07	1.54E-06
ENSG00000188185	LINC00265	46.39108624	124.5226384	-1.423268766	2.06E-07	1.56E-06
ENSG00000005379	TSPOAP1	155.7670502	287.9227536	-0.887533347	2.11E-07	1.59E-06
ENSG00000219607	PPP1R3G	57.4718397	147.4748553	-1.357269936	2.20E-07	1.65E-06
ENSG00000101680	LAMA1	290.7587014	160.9450151	0.851720172	2.22E-07	1.67E-06
ENSG00000239672	NME1	313.3443034	171.0442921	0.87648325	2.23E-07	1.68E-06

ENSG00000108602	ALDH3A1	2.796289078	39.87651142	-3.8320133	2.25E-07	1.69E-06
ENSG00000171877	FRMD5	164.706164	67.1802597	1.30126799	2.28E-07	1.71E-06
ENSG00000162777	DENND2D	18.38717405	74.02496478	-2.007763974	2.39E-07	1.79E-06
ENSG00000100036	SLC35E4	166.050844	305.9239022	-0.883191258	2.40E-07	1.79E-06
ENSG00000186417	GLDN	0	26.6605845	-6.916019876	2.42E-07	1.81E-06
ENSG00000267365	KCNJ2-AS1	10.69035296	65.25678681	-2.600108622	2.45E-07	1.83E-06
ENSG00000061918	GUCY1B1	76.61298673	181.5923979	-1.244258636	2.48E-07	1.85E-06
ENSG00000249035	CLMAT3	97.56393015	31.3682014	1.631897365	2.66E-07	1.98E-06
ENSG00000100368	CSF2RB	41.88184832	3.713990969	3.536639049	2.72E-07	2.02E-06
ENSG00000162496	DHRS3	204.3693086	96.63428865	1.078866152	2.74E-07	2.03E-06
ENSG00000100739	BDKRB1	30.27913091	98.44232151	-1.698536581	2.77E-07	2.06E-06
ENSG00000176641	RNF152	110.505583	224.3929094	-1.02075716	2.79E-07	2.07E-06
ENSG00000149633	KIAA1755	41.71467536	4.791286583	3.109025684	2.85E-07	2.11E-06
ENSG00000143891	GALM	135.5099904	262.397394	-0.951948057	2.85E-07	2.11E-06
ENSG00000145687	SSBP2	225.8191907	385.1623945	-0.770677015	2.90E-07	2.14E-06
ENSG00000106031	HOXA13	11.59858201	58.59391492	-2.336145157	2.96E-07	2.19E-06
ENSG00000139668	WDFY2	209.2784943	360.9769922	-0.787116648	3.08E-07	2.27E-06
ENSG00000267123	LINC02081	32.43881708	2.321489062	3.760011069	3.17E-07	2.34E-06
ENSG00000154589	LY96	63.99623313	161.4515702	-1.335004985	3.21E-07	2.36E-06
ENSG00000169660	HEXDC	98.14782095	215.4102911	-1.138029061	3.22E-07	2.37E-06
ENSG00000105404	RABAC1	360.7721451	205.2947893	0.811417964	3.29E-07	2.42E-06
ENSG00000165185	KIAA1958	77.64953744	180.2878877	-1.2159857	3.59E-07	2.63E-06
ENSG00000235649	MXRA5Y	154.8861599	62.35871888	1.311790354	3.62E-07	2.65E-06
ENSG00000123892	RAB38	7.969851526	52.04497868	-2.703046825	3.67E-07	2.68E-06
ENSG00000175899	A2M	72.90198499	18.48845782	1.982749109	3.71E-07	2.71E-06
ENSG00000166924	NYAP1	1.23759631	39.8756401	-5.041378616	3.77E-07	2.75E-06
ENSG00000120075	HOXB5	116.9102582	230.0978295	-0.975403744	3.83E-07	2.79E-06
ENSG00000102547	CAB39L	215.2775039	373.7890647	-0.796621889	3.85E-07	2.81E-06
ENSG00000167775	CD320	166.1440668	310.6812575	-0.904736584	3.89E-07	2.83E-06
ENSG00000103876	FAH	162.5286361	337.8879284	-1.056617984	3.89E-07	2.83E-06
ENSG00000230733	AC092171.2	321.6877854	188.572637	0.770033803	3.89E-07	2.83E-06
ENSG00000257365	FNTB	220.8803684	98.40491602	1.161425222	3.91E-07	2.84E-06
ENSG00000279415	AC099494.2	49.34397961	6.985002698	2.86400713	3.99E-07	2.90E-06
ENSG00000197077	KIAA1671	110.8806681	220.668973	-0.993046424	4.07E-07	2.95E-06
ENSG00000103145	HCFC1R1	240.528574	428.5498836	-0.83113471	4.19E-07	3.04E-06
ENSG00000095587	TLL2	48.7156475	7.889992082	2.606321246	4.21E-07	3.05E-06
ENSG00000128228	SDF2L1	263.4862744	131.2160028	1.006960305	4.52E-07	3.27E-06
ENSG00000144597	EAF1	289.7081118	163.8478466	0.822192476	4.65E-07	3.36E-06
ENSG00000169126	ARMC4	12.90961776	62.33298998	-2.277415779	4.74E-07	3.42E-06
ENSG00000139173	TMEM117	300.1924405	168.5664953	0.833776508	4.81E-07	3.46E-06
ENSG00000125459	MSTO1	287.9630508	147.6789319	0.967152578	4.84E-07	3.49E-06
ENSG00000267248	AC025048.2	96.71400323	31.4717618	1.620481124	5.13E-07	3.69E-06
ENSG00000156869	FRRS1	79.15278697	22.37454001	1.821363852	5.16E-07	3.71E-06
ENSG00000064655	EYA2	85.02112668	26.51249105	1.684887587	5.19E-07	3.73E-06
ENSG00000188747	NOXA1	33.68881959	111.63554	-1.731666272	5.58E-07	4.00E-06
ENSG00000183570	PCBP3	106.1736264	220.9878798	-1.059816128	5.65E-07	4.05E-06
ENSG00000148908	RGS10	171.5442264	312.5321042	-0.865023747	5.70E-07	4.09E-06
ENSG00000129667	RHBDF2	192.0665565	90.09810361	1.094565709	5.90E-07	4.23E-06
ENSG00000279198	AC008894.3	131.7942923	51.35877472	1.365643559	5.97E-07	4.27E-06
ENSG00000280109	PLAC4	60.97962574	12.83564032	2.253474596	6.11E-07	4.36E-06
ENSG00000187398	LUZP2	33.72861482	3.114918946	3.473253416	6.43E-07	4.59E-06
ENSG00000247317	LY6E-DT	19.30837032	82.69022717	-2.102072388	6.62E-07	4.72E-06
ENSG00000130720	FIBCD1	53.43006642	10.7664499	2.321036497	6.71E-07	4.78E-06
ENSG00000135525	MAP7	92.44956343	198.4606745	-1.099589958	6.94E-07	4.94E-06
ENSG00000073282	TP63	2.86324491	40.36423873	-3.839064638	7.04E-07	5.00E-06
ENSG00000182621	PLCB1	65.67066324	166.1561431	-1.339917468	7.04E-07	5.00E-06
ENSG00000135750	KCNK1	178.8783686	79.87603242	1.168812631	7.20E-07	5.10E-06
ENSG00000130751	NPAS1	72.84100208	20.26535339	1.851850676	7.31E-07	5.18E-06
ENSG00000126787	DLGAP5	72.53141822	162.1094428	-1.160116047	7.74E-07	5.47E-06
ENSG00000235086	FNDC1-IT1	28.04963001	0.234354069	6.580515549	7.86E-07	5.55E-06
ENSG00000116883	AL591845.1	50.80743391	135.319139	-1.414109861	7.87E-07	5.56E-06
ENSG00000153291	SLC25A27	121.8350919	242.1860894	-0.991989983	7.88E-07	5.56E-06
ENSG00000136828	RALGPS1	43.34300475	115.4803366	-1.415725629	8.35E-07	5.87E-06
ENSG00000164306	PRIMPOL	80.05039702	176.3001913	-1.139477691	8.56E-07	6.01E-06
ENSG00000237234	Z99289.1	21.37516624	80.33522488	-1.915491654	8.98E-07	6.28E-06
ENSG00000107672	NSMCE4A	191.7481911	328.9035792	-0.779346021	9.14E-07	6.39E-06

ENSG00000128655	PDE11A	33.7373963	2.61810598	3.747206473	9.16E-07	6.40E-06
ENSG00000102312	PORCN	277.4245283	153.5176779	0.851647008	9.28E-07	6.48E-06
ENSG00000109805	NCAPG	88.93401728	195.5909262	-1.135181065	9.65E-07	6.73E-06
ENSG00000130748	TMEM160	267.3912771	141.8371649	0.916753357	9.72E-07	6.77E-06
ENSG00000239828	AC063944.1	38.03228883	4.228748511	3.199185061	9.73E-07	6.78E-06
ENSG00000176907	TCIM	18.56177455	0	6.94534166	9.98E-07	6.94E-06
ENSG00000114805	PLCH1	45.08564051	6.836694239	2.753540897	1.08E-06	7.47E-06
ENSG00000137642	SORL1	2.328313744	39.55156316	-4.051909772	1.15E-06	7.98E-06
ENSG00000135740	SLC9A5	67.97449717	160.7586565	-1.243915979	1.20E-06	8.28E-06
ENSG00000254554	AC080023.1	18.59687336	73.80746896	-1.992976768	1.21E-06	8.32E-06
ENSG00000225783	MIAT	123.7799994	46.39171965	1.423215405	1.22E-06	8.38E-06
ENSG00000105509	HAS1	40.92649716	6.093420814	2.733113859	1.22E-06	8.39E-06
ENSG00000140961	OSGIN1	95.8140953	192.520982	-1.007189504	1.25E-06	8.61E-06
ENSG00000175356	SCUBE2	147.4669378	266.0594974	-0.850496706	1.26E-06	8.63E-06
ENSG00000119636	BBOF1	210.7747495	106.5115328	0.987767643	1.28E-06	8.82E-06
ENSG00000231721	LINC-PINT	138.5428069	259.731734	-0.90732729	1.29E-06	8.85E-06
ENSG00000168310	IRF2	191.0478742	322.0463381	-0.752121746	1.30E-06	8.92E-06
ENSG00000180113	TDRD6	5.17680425	41.92624114	-3.009803551	1.31E-06	8.98E-06
ENSG00000132359	RAP1GAP2	10.04313105	51.8638018	-2.370196563	1.32E-06	9.06E-06
ENSG00000153162	BMP6	42.10037944	117.3480414	-1.480667489	1.35E-06	9.23E-06
ENSG00000157388	CACNA1D	8.805534737	50.61571608	-2.525961534	1.36E-06	9.34E-06
ENSG00000049192	ADAMTS6	407.6749325	217.665207	0.901241909	1.37E-06	9.36E-06
ENSG00000250038	AC109588.1	18.2712586	0	6.925546832	1.39E-06	9.51E-06
ENSG00000203760	CENPW	41.65777752	112.1686724	-1.42607414	1.40E-06	9.54E-06
ENSG00000232977	LINC00327	83.4181707	177.5990665	-1.089579144	1.42E-06	9.71E-06
ENSG00000113368	LMNB1	116.9743551	226.3648192	-0.954869164	1.47E-06	1.01E-05
ENSG00000138160	KIF11	183.6572674	319.6303741	-0.80107845	1.49E-06	1.02E-05
ENSG00000101000	PROCR	99.80639827	198.2027642	-0.988914032	1.53E-06	1.04E-05
ENSG00000197905	TEAD4	225.5687523	118.1803812	0.932300314	1.55E-06	1.05E-05
ENSG00000170312	CDK1	106.9378355	214.1887826	-1.000567453	1.58E-06	1.08E-05
ENSG00000106785	TRIM14	352.8904104	200.18333	0.82081536	1.68E-06	1.14E-05
ENSG00000183486	MX2	69.72139611	191.6592479	-1.455823217	1.68E-06	1.14E-05
ENSG00000222022	AC112721.1	17.20502098	0	6.837479192	1.71E-06	1.16E-05
ENSG00000267669	AC098847.1	2.440093802	36.58301827	-3.920605688	1.71E-06	1.16E-05
ENSG00000177432	NAP1L5	221.8041952	115.5882191	0.941046678	1.89E-06	1.28E-05
ENSG00000158716	DUSP23	300.7544819	169.2140041	0.826719236	1.93E-06	1.30E-05
ENSG00000275226	LINC00547	27.80483189	0.925524897	5.051851796	1.93E-06	1.30E-05
ENSG00000152284	TCF7L1	105.2875289	214.4441022	-1.024215622	1.95E-06	1.31E-05
ENSG00000164440	TXLNB	5.110742014	42.69978032	-3.045439174	2.00E-06	1.35E-05
ENSG00000237649	KIFC1	70.40683087	155.8512734	-1.146222727	2.00E-06	1.35E-05
ENSG00000138495	COX17	215.7227866	98.56857856	1.135073353	2.01E-06	1.35E-05
ENSG00000169679	BUB1	84.83355897	179.0596812	-1.076431431	2.03E-06	1.36E-05
ENSG00000182511	FES	106.8314673	217.7229215	-1.027136292	2.03E-06	1.36E-05
ENSG00000119661	DNAL1	349.7104596	204.0847656	0.77481721	2.04E-06	1.37E-05
ENSG00000151778	SERP2	41.96914857	112.2694266	-1.4213543	2.06E-06	1.38E-05
ENSG00000129465	RIPK3	128.9553994	241.832188	-0.90940928	2.08E-06	1.40E-05
ENSG00000271614	ATP2B1-AS1	31.24369679	92.27992834	-1.559707527	2.11E-06	1.41E-05
ENSG00000082196	C1QTNF3	181.7675479	89.40023479	1.022607192	2.15E-06	1.44E-05
ENSG00000075826	SEC31B	145.1681329	263.3843335	-0.861997896	2.20E-06	1.47E-05
ENSG00000276365	MIR145	49.67983047	10.59414367	2.216037491	2.21E-06	1.48E-05
ENSG00000262097	LINC02185	0	20.77946613	-6.554920849	2.22E-06	1.48E-05
ENSG00000112984	KIF20A	151.7285962	307.1205416	-1.016630946	2.23E-06	1.49E-05
ENSG00000198839	ZNF277	174.2467316	300.4591863	-0.785592804	2.33E-06	1.55E-05
ENSG00000153930	ANKFN1	5.610574363	41.41428932	-2.883089856	2.35E-06	1.56E-05
ENSG00000198932	GPRASP1	86.87732969	175.5493368	-1.014479627	2.35E-06	1.57E-05
ENSG00000076382	SPAG5	109.4305136	210.1719065	-0.943803363	2.37E-06	1.58E-05
ENSG00000154898	CCDC144CP	64.90065936	18.43224817	1.816298195	2.40E-06	1.59E-05
ENSG00000189423	USP32P3	48.75036341	10.50809806	2.211440792	2.42E-06	1.61E-05
ENSG00000260855	AL591848.4	14.45858512	62.24132097	-2.110030294	2.42E-06	1.61E-05
ENSG00000169418	NPR1	1.23759631	33.12434667	-4.775363635	2.60E-06	1.72E-05
ENSG00000101938	CHRDL1	16.6247549	75.38417999	-2.189820797	2.67E-06	1.77E-05
ENSG00000120162	MOB3B	31.22894239	3.114918946	3.361471139	2.68E-06	1.77E-05
ENSG00000241749	RPSAP52	8.5186435	49.69408346	-2.554093154	2.71E-06	1.79E-05
ENSG00000174576	NPAS4	39.67414645	5.017856329	2.968065186	2.74E-06	1.81E-05
ENSG00000115267	IFIH1	102.6840147	203.0597874	-0.984255869	2.75E-06	1.82E-05
ENSG00000197183	NOL4L	261.6893949	147.5258714	0.825116067	2.80E-06	1.84E-05

ENSG00000097046	CDC7	65.66525134	143.8079883	-1.130792583	2.85E-06	1.87E-05
ENSG00000154822	PLCL2	66.04955114	151.8590401	-1.202856556	2.89E-06	1.90E-05
ENSG00000057657	PRDM1	245.4896789	135.3184827	0.861467013	2.92E-06	1.92E-05
ENSG00000179841	AKAP5	66.193316	12.31829184	2.402088485	2.94E-06	1.93E-05
ENSG00000230487	PSMG3-AS1	180.0927113	322.8334996	-0.842385818	2.96E-06	1.94E-05
ENSG00000175130	MARCKSL1	191.0574215	94.61914697	1.014524485	3.07E-06	2.01E-05
ENSG00000140525	FANCI	182.6442526	310.2028194	-0.762693074	3.08E-06	2.02E-05
ENSG00000162383	SLC1A7	99.65717118	193.8994317	-0.958344995	3.11E-06	2.03E-05
ENSG00000141391	PRELID3A	255.8418328	145.0567186	0.816790287	3.14E-06	2.05E-05
ENSG00000123353	ORMDL2	278.7092467	164.4655197	0.761081317	3.24E-06	2.11E-05
ENSG00000137628	DDX60	156.2951149	279.667829	-0.837411818	3.28E-06	2.14E-05
ENSG00000139116	KIF21A	252.6854415	131.2440959	0.947551465	3.43E-06	2.23E-05
ENSG00000248323	LUCAT1	7.949507111	47.54100835	-2.576513171	3.47E-06	2.25E-05
ENSG00000119917	IFIT3	163.3892328	640.8117662	-1.970749835	3.49E-06	2.26E-05
ENSG00000168679	SLC16A4	191.2360804	87.60128759	1.126716084	3.49E-06	2.26E-05
ENSG00000068489	PRR11	123.9783643	231.6823131	-0.901600532	3.52E-06	2.28E-05
ENSG00000218052	ADAMTS7P4	0.388052291	29.73982931	-6.10723952	3.62E-06	2.34E-05
ENSG00000277954	AC092376.2	2.855867708	33.62158943	-3.576129907	3.63E-06	2.35E-05
ENSG00000163017	ACTG2	30.33960313	2.319757933	3.766606862	3.68E-06	2.38E-05
ENSG00000228784	LINC00954	20.85174767	73.39972233	-1.814096915	3.69E-06	2.38E-05
ENSG00000284968	AC093827.4	16.70932403	65.4141807	-1.966977634	3.77E-06	2.43E-05
ENSG00000102384	CENPI	62.39685153	138.956385	-1.153960253	3.79E-06	2.45E-05
ENSG00000011347	SYT7	14.27660743	62.18186407	-2.115164838	3.83E-06	2.47E-05
ENSG00000137193	PIM1	283.4540177	157.4171681	0.852474207	3.85E-06	2.48E-05
ENSG00000249835	VCAN-AS1	44.12235113	8.643425759	2.369168551	3.89E-06	2.50E-05
ENSG00000004660	CAMKK1	143.5199698	255.0437928	-0.829856551	3.90E-06	2.51E-05
ENSG00000230267	HERC2P4	51.17011279	129.8565721	-1.346191728	3.99E-06	2.57E-05
ENSG00000155926	SLA	29.36066576	2.61810598	3.543871005	3.99E-06	2.57E-05
ENSG00000272168	CASC15	92.31603467	29.25382139	1.669025226	4.08E-06	2.62E-05
ENSG00000162572	SCNN1D	76.69793877	165.0896756	-1.10738281	4.12E-06	2.64E-05
ENSG00000144057	ST6GAL2	16.37582137	0	6.766522172	4.28E-06	2.74E-05
ENSG00000161682	FAM171A2	195.1935396	100.6131262	0.953225429	4.39E-06	2.81E-05
ENSG00000169760	NLGN1	45.19042627	123.6297554	-1.453543535	4.51E-06	2.88E-05
ENSG00000170425	ADORA2B	8.777813121	47.13067101	-2.418758008	4.70E-06	2.99E-05
ENSG00000102683	SGCG	120.5251279	49.93838277	1.263586638	4.81E-06	3.06E-05
ENSG00000189057	FAM111B	20.49555239	72.72628107	-1.829296953	4.87E-06	3.09E-05
ENSG00000112742	TTK	40.4136976	105.0066116	-1.375687827	4.94E-06	3.13E-05
ENSG00000127328	RAB3IP	46.5278568	9.084029071	2.367620283	4.95E-06	3.14E-05
ENSG00000085276	MECOM	68.41023259	18.75934584	1.876026512	4.99E-06	3.16E-05
ENSG00000075218	GTSE1	87.78372122	182.4768285	-1.053634746	5.12E-06	3.24E-05
ENSG00000132321	IQCA1	38.18978671	6.107258332	2.658500626	5.14E-06	3.25E-05
ENSG00000154134	ROBO3	89.07155366	186.3616211	-1.066302275	5.20E-06	3.29E-05
ENSG00000143363	PRUNE1	244.4748769	136.9412431	0.834099001	5.22E-06	3.30E-05
ENSG00000274370	AC130371.2	53.66799804	123.7858479	-1.206512684	5.26E-06	3.32E-05
ENSG00000200033	RNU6-403P	30.61536468	2.744147798	3.414795417	5.27E-06	3.33E-05
ENSG00000162437	RAVER2	103.241971	200.270235	-0.956753393	5.31E-06	3.35E-05
ENSG00000139044	B4GALNT3	21.18435673	75.04603901	-1.823293451	5.39E-06	3.40E-05
ENSG00000171658	NMRAL2P	16.90426893	64.17863138	-1.927711489	5.43E-06	3.42E-05
ENSG00000203722	RAET1G	17.29556303	67.55254697	-1.966364375	5.44E-06	3.42E-05
ENSG00000117115	PADI2	25.58326921	1.674636508	3.962059634	5.48E-06	3.45E-05
ENSG00000158014	SLC30A2	24.89211666	0.617016598	5.489241602	5.48E-06	3.45E-05
ENSG00000130021	PUDP	164.4646077	291.6229193	-0.824765753	5.52E-06	3.47E-05
ENSG00000231445	TIMM8AP1	63.58045922	144.0425572	-1.180771187	5.67E-06	3.55E-05
ENSG00000124249	KCNK15	159.549832	289.26726	-0.857987548	5.73E-06	3.58E-05
ENSG00000158163	DZIP1L	371.8866705	217.4838034	0.768847978	5.81E-06	3.64E-05
ENSG00000237352	LINC01358	6.019864663	42.40705567	-2.813944627	5.85E-06	3.66E-05
ENSG00000197766	CFD	94.87161069	212.4291985	-1.161952055	5.86E-06	3.66E-05
ENSG00000230479	AP000695.1	26.89844035	2.321489062	3.491949191	6.10E-06	3.81E-05
ENSG00000188385	JAKMIP3	5.998626654	41.94721796	-2.809560927	6.10E-06	3.81E-05
ENSG00000160563	MED27	263.934416	150.4983203	0.8114384	6.16E-06	3.84E-05
ENSG00000161647	MPP3	307.3745742	178.3663495	0.787567793	6.36E-06	3.96E-05
ENSG00000124406	ATP8A1	24.86115324	79.07243055	-1.666652294	6.42E-06	3.99E-05
ENSG00000163739	CXCL1	37.03065883	5.688706653	2.701359998	6.44E-06	4.00E-05
ENSG00000243279	PRAF2	230.1339441	121.8139499	0.92074752	6.47E-06	4.02E-05
ENSG00000171790	SLFNL1	46.23218408	8.582894038	2.405258002	6.51E-06	4.04E-05
ENSG00000203709	MIR29B2CHG	56.55107667	144.9103563	-1.361201016	6.87E-06	4.25E-05

ENSG00000180769	WDFY3-AS2	43.35362375	113.8147738	-1.396521599	7.10E-06	4.39E-05
ENSG00000184635	ZNF93	137.8188534	62.4192506	1.145559868	7.13E-06	4.41E-05
ENSG00000283073	SMUG1-AS1	4.432556683	36.7172742	-3.058276434	7.24E-06	4.47E-05
ENSG00000143195	ILDR2	29.55471707	2.047138785	3.909517146	7.25E-06	4.48E-05
ENSG00000099282	TSPAN15	27.17884798	82.349937	-1.595648546	7.34E-06	4.53E-05
ENSG00000204991	SPIRE2	68.26748911	149.0904645	-1.126505905	7.48E-06	4.61E-05
ENSG00000197632	SERPINB2	115.6956871	50.72965173	1.190166856	7.57E-06	4.66E-05
ENSG00000054356	PTPRN	43.23122469	9.101973646	2.258507655	7.93E-06	4.88E-05
ENSG00000107020	PLGRKT	225.6432634	124.5148541	0.859094689	8.05E-06	4.95E-05
ENSG00000103490	PYCARD	36.27489867	101.2839765	-1.485653622	8.23E-06	5.06E-05
ENSG00000215769	ARHGAP27P1-BPTFP1-KPNA2P3	45.46486098	117.1121593	-1.364947971	8.31E-06	5.11E-05
ENSG00000184185	KCNJ12	138.5883466	60.77034299	1.190333881	8.32E-06	5.11E-05
ENSG00000184588	PDE4B	220.6243903	122.129156	0.851156598	8.49E-06	5.21E-05
ENSG00000096060	FKBP5	162.9756019	77.65419974	1.073631141	8.67E-06	5.31E-05
ENSG00000261189	AL031058.1	104.2108504	40.65242652	1.354110163	8.73E-06	5.34E-05
ENSG00000080854	IGSF9B	3.265158008	34.45306939	-3.418834507	8.78E-06	5.38E-05
ENSG00000133460	SLC2A11	285.8990127	151.8601144	0.910044623	8.81E-06	5.39E-05
ENSG00000162894	FCMR	44.55612124	8.767736448	2.352318081	8.82E-06	5.39E-05
ENSG00000104490	NCALD	92.32341188	178.8886646	-0.954173608	8.98E-06	5.49E-05
ENSG00000143355	LHX9	122.0084159	50.51951044	1.280153963	9.08E-06	5.55E-05
ENSG00000215386	MIR99AHG	58.13330533	133.5076439	-1.200946081	9.08E-06	5.55E-05
ENSG00000138615	CILP	0.789965389	29.63735524	-5.213959434	9.13E-06	5.57E-05
ENSG00000131669	NINJ1	261.2486808	149.3745454	0.808934002	9.36E-06	5.70E-05
ENSG00000167700	MFSD3	253.0703561	144.8398794	0.804516566	9.39E-06	5.72E-05
ENSG00000165983	PTER	97.96456675	192.0299955	-0.970301037	9.48E-06	5.77E-05
ENSG00000144354	CDCA7	10.02189304	46.93004495	-2.229656864	9.56E-06	5.82E-05
ENSG00000237187	NR2F1-AS1	273.7113061	151.7303958	0.856868668	9.62E-06	5.85E-05
ENSG00000170365	SMAD1	240.6552092	118.5007926	1.019938379	9.68E-06	5.89E-05
ENSG00000041353	RAB27B	3.118279133	34.68720867	-3.449301038	9.69E-06	5.89E-05
ENSG00000176485	PLA2G16	191.6198695	98.9867	0.948026133	9.70E-06	5.90E-05
ENSG00000215218	UBE2QL1	21.37695343	0.308508299	6.187379455	9.75E-06	5.92E-05
ENSG00000119946	CNNM1	25.30375517	80.15102727	-1.666516089	9.94E-06	6.04E-05
ENSG00000061455	PRDM6	36.97342841	6.373824286	2.523380753	1.03E-05	6.22E-05
ENSG00000223901	AP001469.1	67.45901683	146.9613989	-1.126578678	1.05E-05	6.37E-05
ENSG00000159314	ARHGAP27	144.9728554	251.4582196	-0.796227643	1.10E-05	6.66E-05
ENSG00000240065	PSMB9	49.30278009	125.7285667	-1.35421142	1.13E-05	6.83E-05
ENSG00000278709	NKILA	43.30052873	9.580842037	2.182485992	1.14E-05	6.89E-05
ENSG00000240204	SMKR1	5.208661264	35.94091757	-2.784837452	1.17E-05	7.04E-05
ENSG00000166851	PLK1	85.24284927	179.5355056	-1.071172244	1.20E-05	7.21E-05
ENSG00000168453	HR	66.09629034	19.69611732	1.746279506	1.23E-05	7.38E-05
ENSG00000283175	AC007920.2	33.44261718	93.31117433	-1.48568173	1.23E-05	7.40E-05
ENSG00000198133	TMEM229B	18.73364392	65.03346431	-1.793926583	1.27E-05	7.61E-05
ENSG00000116815	CD58	105.0289203	194.2464198	-0.887354595	1.27E-05	7.63E-05
ENSG00000092871	RFFL	107.6741951	201.1631182	-0.901807371	1.29E-05	7.74E-05
ENSG00000134690	CDCA8	54.90324614	122.7180678	-1.161534022	1.30E-05	7.76E-05
ENSG00000185513	L3MBTL1	84.89420931	175.1878661	-1.044386995	1.30E-05	7.78E-05
ENSG00000253210	AC040970.1	18.70178691	64.81705481	-1.790358576	1.30E-05	7.79E-05
ENSG00000205037	AC134312.1	0.412532103	25.67322677	-5.893776919	1.32E-05	7.88E-05
ENSG00000163322	ABRAXAS1	153.075164	258.902415	-0.759564587	1.32E-05	7.90E-05
ENSG00000129467	ADCY4	74.7279904	158.9132299	-1.091804907	1.34E-05	8.02E-05
ENSG00000183935	HTR7P1	109.2858048	211.9412212	-0.955473212	1.35E-05	8.06E-05
ENSG00000226526	AL049569.1	47.541893	9.085760199	2.38231582	1.37E-05	8.19E-05
ENSG00000135324	MRAP2	158.2355544	72.03014338	1.131813762	1.37E-05	8.19E-05
ENSG00000196449	YRDC	170.964471	85.92837047	0.992665553	1.38E-05	8.20E-05
ENSG00000137486	ARRB1	66.23293312	142.9855821	-1.112707283	1.38E-05	8.21E-05
ENSG00000042317	SPATA7	110.6906244	215.3119011	-0.960916465	1.43E-05	8.54E-05
ENSG00000140022	STON2	23.17592601	71.00516537	-1.612809195	1.44E-05	8.57E-05
ENSG00000141576	RNF157	82.76492554	180.4173686	-1.120921957	1.45E-05	8.60E-05
ENSG00000283196	AC006453.2	212.7676224	366.5468083	-0.783944471	1.48E-05	8.78E-05
ENSG00000184363	PKP3	3.219440186	32.32572318	-3.337049955	1.52E-05	9.02E-05
ENSG00000259863	SH3RF3-AS1	72.68115599	25.07631567	1.533908677	1.61E-05	9.55E-05
ENSG00000103522	IL21R	41.18477319	7.783625968	2.397538948	1.64E-05	9.72E-05
ENSG00000117407	ARTN	12.89899875	53.81494973	-2.060889651	1.67E-05	9.88E-05
ENSG00000236383	CCDC200	97.70215533	38.67101284	1.328144782	1.69E-05	9.98E-05
ENSG00000160352	ZNF714	169.8174167	88.31169259	0.946353711	1.72E-05	0.000101458
ENSG00000279207	AC015813.6	59.97010785	133.8196147	-1.159078392	1.85E-05	0.000108885

ENSG00000130653	PNPLA7	130.9174096	232.7233015	-0.830264133	1.91E-05	0.000112442
ENSG00000215883	CYB5RL	157.9084076	268.0590822	-0.763730177	1.98E-05	0.000116121
ENSG00000012048	BRCA1	109.7958733	200.8502763	-0.869888095	1.98E-05	0.000116285
ENSG00000115457	IGFBP2	7.925027299	43.43462462	-2.444395583	2.01E-05	0.000117739
ENSG00000123179	EBPL	73.01884438	149.070574	-1.029540656	2.05E-05	0.000119665
ENSG00000105792	CFAP69	90.70514048	172.9148024	-0.933313522	2.07E-05	0.00012095
ENSG00000196196	HRCT1	69.99810159	20.32070323	1.76822313	2.15E-05	0.000125399
ENSG00000173320	STOX2	0	15.48358225	-6.129513639	2.17E-05	0.00012625
ENSG00000177380	PPFIA3	81.25485982	28.33954308	1.523513252	2.23E-05	0.00013018
ENSG00000260439	LMF1-AS1	50.00830413	12.89790317	1.972798798	2.27E-05	0.000132118
ENSG00000231365	AL359915.2	39.73048328	97.92215558	-1.303186916	2.28E-05	0.000132717
ENSG00000169083	AR	33.80618966	89.73360044	-1.41297121	2.30E-05	0.0001337
ENSG00000254858	MPV17L2	180.0226143	92.4931021	0.960165838	2.32E-05	0.000134899
ENSG00000132122	SPATA6	64.95018001	147.7245395	-1.18553599	2.35E-05	0.000136532
ENSG00000171425	ZNF581	127.6842867	229.2762948	-0.841994584	2.42E-05	0.000140612
ENSG00000010219	DYRK4	197.7176919	107.1250869	0.88278506	2.44E-05	0.000141671
ENSG00000250602	AC093535.1	87.37772306	33.05256837	1.4055981	2.46E-05	0.000142766
ENSG00000144199	FAHD2B	19.88304639	66.61146516	-1.739427483	2.54E-05	0.000146752
ENSG00000253649	PRSS51	40.97724398	104.6408071	-1.355525252	2.55E-05	0.000147645
ENSG00000127083	OMD	118.0938155	214.2778607	-0.858295814	2.61E-05	0.000151058
ENSG00000204362	AL590644.1	59.54746743	17.10979343	1.800137124	2.73E-05	0.000157135
ENSG00000148082	SHC3	12.89251515	52.49227999	-2.02789688	2.82E-05	0.000162388
ENSG00000174132	FAM174A	258.9257019	153.3153209	0.757349913	2.86E-05	0.000164401
ENSG00000165171	METTL27	68.40050718	143.1341173	-1.065343866	2.90E-05	0.000166375
ENSG00000227953	LINC01341	9.972933412	44.44360419	-2.154094617	2.95E-05	0.000169324
ENSG00000154642	C21orf91	219.8020069	127.9568707	0.782004403	2.96E-05	0.000169649
ENSG00000119969	HELLS	60.54891932	129.6423237	-1.10115808	3.01E-05	0.000172527
ENSG00000224858	RPL29P11	73.6529713	19.71665283	1.913611471	3.01E-05	0.00017261
ENSG00000235823	OLMALINC	60.21955206	128.7704061	-1.097309736	3.03E-05	0.000173232
ENSG00000124491	F13A1	129.7405139	1.851049794	6.177060087	3.09E-05	0.00017651
ENSG00000279348	AC012513.3	62.52338599	132.0336335	-1.077945198	3.17E-05	0.000180922
ENSG00000124074	ENKD1	139.5543167	236.6800757	-0.762748006	3.17E-05	0.000181057
ENSG00000269973	AC010969.2	28.4261697	77.50524648	-1.445146848	3.20E-05	0.00018243
ENSG00000284730	AC068987.5	41.18242498	8.93745174	2.187248264	3.39E-05	0.000192918
ENSG00000137273	FOXF2	96.90838711	183.6812523	-0.923502056	3.42E-05	0.000194882
ENSG00000142494	SLC47A1	9.85791155	48.16429315	-2.276866042	3.45E-05	0.000196305
ENSG00000128340	RAC2	58.08669391	122.977721	-1.082261285	3.56E-05	0.000201929
ENSG00000157542	KCNJ6	54.0194969	15.69999175	1.782497348	3.61E-05	0.000204825
ENSG00000170577	SIX2	9.255513866	46.13077688	-2.320785331	3.84E-05	0.000217281
ENSG00000241697	TMEFF1	68.59182737	23.45378176	1.55209074	3.91E-05	0.000220859
ENSG00000109794	FAM149A	55.41566279	124.9688534	-1.169108205	3.93E-05	0.000221979
ENSG00000171388	APLN	2.74408765	28.98098724	-3.3809633	4.20E-05	0.000236492
ENSG00000285051	AC026316.4	0.412532103	20.93188164	-5.6044048	4.25E-05	0.000239068
ENSG00000255440	AP001085.1	11.60182382	0	6.269023439	4.26E-05	0.000239918
ENSG00000238279	BX470102.1	9.937834595	44.21594811	-2.146840918	4.35E-05	0.000244114
ENSG00000224690	UBE2D3P3	27.58076108	76.24267844	-1.46880044	4.35E-05	0.000244189
ENSG00000026297	RNASET2	150.1205338	253.9370913	-0.758689209	4.35E-05	0.000244475
ENSG00000280237	MIR4697HG	2.411478591	29.01623159	-3.592742351	4.42E-05	0.000247875
ENSG00000092969	TGFB2	54.39514299	15.84397814	1.767290942	4.63E-05	0.000259101
ENSG00000135363	LMO2	2.740845847	29.75820367	-3.420545759	4.70E-05	0.000262551
ENSG00000157554	ERG	35.03730236	5.807179156	2.631639613	4.83E-05	0.000268965
ENSG00000158050	DUSP2	44.63152598	11.0289088	2.026984255	4.90E-05	0.000272744
ENSG00000126860	EVI2A	41.48914994	100.1054964	-1.274715595	5.13E-05	0.000284572
ENSG00000167178	ISLR2	25.46914094	3.076653867	3.072590499	5.34E-05	0.000295931
ENSG00000250320	AC113383.1	16.38319857	0.280403472	5.805346623	5.50E-05	0.000303789
ENSG00000224397	SMIM25	38.43096013	90.9775791	-1.241671145	5.67E-05	0.00031291
ENSG00000226711	FAM66C	19.6908326	60.8250363	-1.631258529	5.67E-05	0.000313181
ENSG00000166278	C2	12.08779536	46.93804428	-1.959765219	5.70E-05	0.000314509
ENSG00000274070	CASTOR2	183.5430888	93.6884402	0.965654427	5.81E-05	0.00032005
ENSG00000279196	AC135048.4	13.33511708	53.82187425	-2.017739282	5.91E-05	0.00032571
ENSG00000243710	CFAP57	3.642591294	28.49542085	-2.979537466	5.98E-05	0.000329174
ENSG00000142065	ZFP14	95.39559028	176.0251845	-0.88254432	6.18E-05	0.000339995
ENSG00000164251	F2RL1	12.93460826	0	6.428856362	6.23E-05	0.000341879
ENSG00000123485	HJURP	71.72631552	149.8808619	-1.061657573	6.23E-05	0.000341957
ENSG00000100526	CDKN3	42.82193439	99.41345428	-1.213325447	6.37E-05	0.000349255
ENSG00000224963	U82695.1	18.92948242	0.617016598	5.083812001	6.40E-05	0.000350729

ENSG00000162650	ATXN7L2	61.1439398	129.8353805	-1.084369599	6.40E-05	0.000350729
ENSG00000134339	SAA2	15.59736858	0.234354069	5.734262089	6.45E-05	0.000353084
ENSG00000105750	ZNF85	119.5193625	57.79400205	1.04645537	6.63E-05	0.000362975
ENSG00000217236	SP9	45.88242208	115.1670765	-1.32682139	6.66E-05	0.000364188
ENSG00000182836	PLCXD3	0	13.79921529	-5.96854101	6.67E-05	0.000364973
ENSG00000092470	WDR76	60.47782809	125.1511048	-1.047576673	6.92E-05	0.000377834
ENSG00000105290	APLP1	172.4677205	93.89035587	0.879917045	7.06E-05	0.000385198
ENSG00000186854	TRABD2A	9.640324353	42.63514154	-2.146355635	7.10E-05	0.000386816
ENSG00000243649	CFB	14.27660743	54.4042913	-1.921709321	7.11E-05	0.000387518
ENSG00000140263	SORD	116.5499275	202.0023825	-0.794391834	7.17E-05	0.000390396
ENSG00000267013	LINC01929	10.81185843	0	6.167095921	7.21E-05	0.000392367
ENSG00000162946	DISC1	167.7283111	93.69967527	0.842230217	7.21E-05	0.000392623
ENSG00000263812	LINC00908	4.78875196	30.71724179	-2.677486519	7.26E-05	0.000394912
ENSG00000226445	BX322234.1	180.9590253	92.99812895	0.952580556	7.41E-05	0.000402744
ENSG00000146409	SLC18B1	118.0508288	57.20205781	1.044307438	7.49E-05	0.000406419
ENSG00000273356	LINC02019	8.819395545	39.5811841	-2.166225886	7.51E-05	0.000407513
ENSG00000226318	RPS3AP38	11.32230978	47.1899129	-2.069607932	7.53E-05	0.000408564
ENSG00000177990	DPY19L2	32.19853726	84.55770519	-1.398578861	7.62E-05	0.000412881
ENSG00000239713	APOBEC3G	23.72885339	69.99403639	-1.564148775	7.73E-05	0.000417979
ENSG00000135472	FAIM2	0	13.31861577	-5.914444955	7.76E-05	0.000419755
ENSG00000241104	CEACAMP10	5.95704423	35.47458536	-2.565576501	7.86E-05	0.000424534
ENSG00000158246	TENT5B	15.28689113	52.50827843	-1.783191245	7.90E-05	0.000426574
ENSG00000162241	SLC25A45	207.4955259	114.8635351	0.854246074	7.96E-05	0.000429255
ENSG00000165071	TMEM71	8.169825424	44.41246689	-2.455329762	8.17E-05	0.000440476
ENSG00000161544	CYGB	14.02716321	50.98195038	-1.861881567	8.47E-05	0.000455804
ENSG00000186868	MAPT	12.86389994	47.21217955	-1.881084435	8.62E-05	0.000463041
ENSG00000090447	TFAP4	40.19376219	96.62520322	-1.264599799	8.95E-05	0.000479778
ENSG00000136895	GARNL3	10.74255439	44.72443745	-2.056199577	8.98E-05	0.000481031
ENSG00000224020	MIR181A2HG	19.4949941	1.572377451	3.604523924	9.32E-05	0.000498658
ENSG00000175063	UBE2C	44.37000815	98.61462774	-1.151139577	9.74E-05	0.000519464
ENSG00000138185	ENTPD1	35.24427055	87.63176834	-1.314382274	9.78E-05	0.000521471
ENSG00000106462	EZH2	108.875799	194.1337855	-0.83488626	9.79E-05	0.000521823
ENSG00000245975	AC090515.2	29.17779447	4.578984146	2.633142672	9.85E-05	0.000524612
ENSG00000174343	CHRNA9	10.41318713	0	6.112866523	9.90E-05	0.000527043
ENSG00000145979	TBC1D7	218.0917388	129.3653828	0.753430488	0.000100526	0.000535015
ENSG00000185862	EVI2B	8.857736165	41.15809863	-2.214193511	0.000101759	0.000541023
ENSG00000175820	CCDC168	14.36066587	50.82996465	-1.822213392	0.000101782	0.000541023
ENSG00000183765	CHEK2	77.63802484	148.2138064	-0.935090544	0.00010368	0.000550012
ENSG00000213057	C1orf220	14.74782456	50.51561817	-1.772463249	0.000104346	0.000553268
ENSG00000143786	CNIH3	56.49200873	116.4207736	-1.043845237	0.000107372	0.000568744
ENSG00000279838	AL356273.3	26.387989	3.697777522	2.820206381	0.000107754	0.000570486
ENSG00000272088	AL512413.1	20.35102173	1.480278646	3.703973297	0.000108649	0.000574938
ENSG00000002745	WNT16	4.320776625	30.53477531	-2.807223548	0.000108695	0.000575035
ENSG00000111058	ACSS3	133.7770801	228.6210016	-0.770201696	0.000109461	0.000578802
ENSG00000160256	FAM207A	247.6095195	137.4283254	0.84309326	0.000111692	0.000589146
ENSG00000142632	ARHGEF19	155.110281	80.48267353	0.940667464	0.000111698	0.000589146
ENSG00000272316	AL021368.2	42.56041656	98.34545911	-1.209895055	0.000111722	0.000589146
ENSG00000161981	SNRNP25	214.5028036	123.6950507	0.798062587	0.000112263	0.000591705
ENSG00000173041	ZNF680	41.31868485	97.13738156	-1.233912768	0.000113004	0.000595315
ENSG00000228561	AC026355.1	25.70007826	3.950076167	2.682521649	0.000114749	0.000603758
ENSG00000204682	CASC10	59.24475008	128.0818262	-1.11380648	0.000114921	0.000604513
ENSG00000103254	FAM173A	269.361558	158.5960775	0.759470574	0.000116403	0.00061201
ENSG00000172575	RASGRP1	37.71119237	9.031926473	2.051381927	0.00011752	0.000617574
ENSG00000109674	NEIL3	13.97261358	51.23100155	-1.874665269	0.000120549	0.000632709
ENSG00000075035	WSCD2	4.002921969	29.2410702	-2.868345984	0.000121505	0.000637573
ENSG00000078487	ZCWPW1	27.64033971	74.27510251	-1.424186777	0.00012166	0.000638228
ENSG00000228649	SNHG26	151.2758085	81.16389912	0.89567777	0.000121785	0.000638566
ENSG00000283421	AF001550.2	4.817367171	30.68719083	-2.675282689	0.000125001	0.000654621
ENSG00000171700	RGS19	179.2655776	95.74465313	0.908888878	0.000127428	0.000666839
ENSG00000111247	RAD51AP1	26.82824272	71.2375733	-1.409276688	0.000129024	0.000674862
ENSG00000235997	LINC01936	42.50497333	10.82849774	1.959856423	0.000131052	0.000684797
ENSG00000272777	AC019131.2	5.96676964	32.66039039	-2.44488441	0.000133454	0.000696149
ENSG00000171509	RXFP1	1.213116497	21.112617	-4.127854752	0.000133557	0.000696516
ENSG00000223458	LMO7DN-IT1	33.77484333	6.477814471	2.359478183	0.000136399	0.000710114
ENSG00000197536	C5orf56	56.61205959	115.6589108	-1.033897882	0.000136621	0.0007111
ENSG00000144061	NPHP1	81.99897961	156.0717901	-0.928298138	0.000137735	0.00071637

ENSG00000128645	HOXD1	24.46158835	3.435318626	2.81558268	0.000138172	0.000718468
ENSG00000272398	CD24	25.75641509	2.702420462	3.320172436	0.000140571	0.000730051
ENSG00000170629	DPY19L2P2	18.17282866	61.8095768	-1.767506596	0.000141738	0.000735572
ENSG00000186765	FSCN2	63.23845733	21.15023727	1.58548927	0.000142992	0.000741355
ENSG00000227220	AL133346.1	29.09462962	5.213945319	2.458141844	0.000146238	0.000757076
ENSG00000093072	ADA2	36.21297183	93.49428584	-1.367675836	0.000146542	0.000758463
ENSG00000069399	BCL3	226.1106003	129.1152448	0.80194113	0.000146723	0.00075903
ENSG00000248869	LINC02511	3.583012664	26.13977377	-2.866067312	0.000151402	0.000781906
ENSG00000256616	AP002414.2	40.76989288	9.997662587	2.054442901	0.000153598	0.000792863
ENSG00000229051	LINC01788	26.67074484	3.677457018	2.83891747	0.000154094	0.000795103
ENSG00000198099	ADH4	2.408236788	23.72272364	-3.30242429	0.00015656	0.000807171
ENSG00000132821	VSTM2L	93.37762621	39.81251744	1.225834468	0.0001576	0.000812339
ENSG00000235652	AL356599.1	25.15866349	67.32101036	-1.418665005	0.000158928	0.000818784
ENSG00000168955	TM4SF20	14.0026834	0.308508299	5.578595558	0.000159219	0.000820087
ENSG00000112137	PHACTR1	43.53432494	95.51051385	-1.131538964	0.000160726	0.000827247
ENSG00000157456	CCNB2	87.53573162	160.9123734	-0.877331584	0.000160834	0.0008276
ENSG00000170011	MYRIP	0.803826197	20.52954341	-4.678783313	0.000161036	0.000828439
ENSG00000087903	RFX2	117.5519404	203.6473978	-0.795258823	0.000164193	0.000843864
ENSG00000105464	GRIN2D	152.1642542	79.20626843	0.944169186	0.000164337	0.0008444
ENSG00000204839	MROH6	17.13247513	56.95236161	-1.735226737	0.000167781	0.000860018
ENSG00000080031	PTPRH	6.012487462	31.77118444	-2.402926645	0.000172795	0.00088423
ENSG00000251364	AC107884.1	6.777973038	32.78643221	-2.270486304	0.000172913	0.000884621
ENSG00000248968	AC012640.1	0	11.62192763	-5.716043294	0.000173245	0.000886106
ENSG00000164308	ERAP2	36.87875097	85.72233624	-1.215468834	0.000174217	0.000890218
ENSG00000103056	SMPD3	11.94091648	45.64866124	-1.926016638	0.000175793	0.000897262
ENSG00000188662	HILS1	26.95242897	4.556932513	2.556356342	0.000176214	0.000899129
ENSG00000137142	IGFBPL1	23.00456733	2.131453266	3.515936225	0.000178751	0.000910544
ENSG00000125378	BMP4	10.08236526	45.67763738	-2.184048579	0.000178886	0.000911014
ENSG00000188707	ZBED6CL	34.35225051	6.840156495	2.312096077	0.00017959	0.000913939
ENSG00000187193	MT1X	30.94383834	5.755076559	2.434013503	0.000179795	0.000914767
ENSG00000223459	TCAF1P1	147.4844234	78.09848032	0.921365621	0.000180978	0.000920341
ENSG00000137727	ARHGAP20	10.05699185	40.22523094	-2.001156189	0.00018304	0.000930605
ENSG00000162643	WDR63	13.62290191	48.27087427	-1.820995084	0.000187353	0.000951172
ENSG00000077274	CAPN6	9.954937206	0	6.049006231	0.000188769	0.000957444
ENSG00000132819	RBM38	157.2755069	85.49296055	0.876970175	0.000193437	0.000979254
ENSG00000099849	RASSF7	124.1408911	63.46151688	0.974568009	0.000195719	0.000990097
ENSG00000103710	RASL12	2.782428271	23.51820553	-3.074420145	0.000201591	0.001017771
ENSG00000111110	PPM1H	72.94954034	29.47369315	1.310788042	0.000212798	0.001069878
ENSG00000037897	METTL1	149.3366691	78.14150877	0.929702966	0.000213245	0.001071873
ENSG00000272505	AC104964.3	36.61360843	88.29439282	-1.264963339	0.000213845	0.001074632
ENSG00000117877	CD3EAP	70.65716867	24.68781496	1.52209693	0.000214883	0.00107934
ENSG00000253161	LINC01605	60.41500766	122.2791841	-1.013663706	0.000218458	0.001095744
ENSG00000279713	AC080038.3	65.53167226	22.02344457	1.561226065	0.000219595	0.001100926
ENSG00000092295	TGM1	52.37585209	17.87922555	1.55085494	0.000222354	0.00111397
ENSG00000156970	BUB1B	64.04983884	134.6337949	-1.068190567	0.000223039	0.001117141
ENSG00000256508	MRGPRF-AS1	14.00771239	48.59841348	-1.791958292	0.000223953	0.001121335
ENSG00000139410	SDSL	55.76665096	19.73200647	1.497080994	0.000224226	0.001122289
ENSG00000258773	AC087636.1	10.84695724	41.30986935	-1.926783938	0.000224889	0.001125347
ENSG00000264268	MIR4767	13.77302259	49.45756847	-1.848756028	0.000226858	0.001134132
ENSG00000226992	AC145625.1	2.369896167	22.70467015	-3.245748954	0.00022711	0.001135122
ENSG00000272321	AP003355.2	0.401913099	16.83241069	-5.285636335	0.000230062	0.001148595
ENSG00000126822	PLEKHG3	95.82701218	169.7968512	-0.822857815	0.000232248	0.001158624
ENSG00000248144	ADH1C	0.776104581	19.4915992	-4.615686142	0.000240871	0.001198267
ENSG00000223653	AL078459.1	44.35436015	11.02869379	1.9839135	0.000241937	0.00120329
ENSG00000280073	AL157996.1	0.412532103	15.91640124	-5.20833854	0.000243373	0.001209298
ENSG00000267361	SEC24AP1	0.401913099	15.98450228	-5.21629501	0.000245264	0.001217843
ENSG00000180035	ZNF48	163.8349991	93.15659789	0.814160728	0.000246148	0.001221946
ENSG00000175287	PHYHD1	90.68910957	164.4620572	-0.85940269	0.000246417	0.001222714
ENSG00000122483	CCDC18	69.13413574	131.6209317	-0.928781831	0.000247668	0.001228061
ENSG00000068831	RASGRP2	43.61889407	10.32216932	2.08337361	0.000249249	0.001235322
ENSG00000166448	TMEM130	63.1452345	22.93945401	1.455661833	0.000250458	0.001240737
ENSG00000278970	HEIH	72.54203723	133.7709743	-0.88322502	0.000251598	0.001245802
ENSG00000130592	LSP1	3.932724334	27.66977908	-2.803192752	0.00025977	0.00128477
ENSG00000158125	XDH	16.21273348	54.6797279	-1.746917605	0.00026463	0.001307895
ENSG00000188766	SPRED3	126.3957654	66.62270001	0.924484397	0.000266954	0.00131877
ENSG00000165879	FRAT1	26.65453582	70.39139602	-1.400293437	0.000267097	0.001319169

ENSG00000102931	ARL2BP	117.4502183	51.69777529	1.174207838	0.00026718	0.001319273
ENSG00000258634	AL160006.1	107.0717471	190.7036722	-0.831692225	0.000267513	0.001320608
ENSG00000277152	AC110048.2	25.12915468	67.61589615	-1.424457212	0.000270029	0.001332101
ENSG00000165480	SKA3	26.56996669	67.97802316	-1.358880287	0.000272043	0.001341726
ENSG00000160460	SPTBN4	112.8015291	197.3924876	-0.810225316	0.000273199	0.001347112
ENSG00000130193	THEM6	83.87317882	149.9193537	-0.839039801	0.000275882	0.001359714
ENSG00000129521	EGLN3	27.15850357	3.599840532	2.975046908	0.000276508	0.001362168
ENSG00000065717	TLE2	108.28397	54.40775355	0.995466546	0.000278251	0.001369945
ENSG00000237523	LINC00857	22.54345851	61.79228854	-1.46076375	0.000281361	0.001384149
ENSG00000278864	AC055811.4	102.8922594	49.59982373	1.05685018	0.000285646	0.001403281
ENSG00000198093	ZNF649	105.8719808	52.59323771	1.011558525	0.000287529	0.001412206
ENSG00000161381	PLXDC1	131.153554	68.40586378	0.945786343	0.000290148	0.001424411
ENSG00000086848	ALG9	88.03773407	156.4354333	-0.831604736	0.000292021	0.001433275
ENSG00000024526	DEPDC1	57.15074324	112.6667865	-0.978589166	0.00029648	0.001452815
ENSG00000107821	KAZALD1	149.437652	79.77312834	0.899801685	0.000297166	0.00145584
ENSG00000106351	AGFG2	63.80128822	125.8885516	-0.979351145	0.000299762	0.001467883
ENSG00000232882	PHKA1P1	32.99912165	6.63822932	2.343822065	0.000302432	0.001479593
ENSG00000265298	AC132812.1	82.63463859	159.9459805	-0.954077717	0.000314778	0.001537517
ENSG00000174370	C11orf45	41.19952759	88.85780221	-1.110096827	0.000318902	0.001555875
ENSG00000120833	SOCS2	21.73963231	62.2503949	-1.521023802	0.000322354	0.001570912
ENSG00000214455	RCN1P2	67.56119926	141.0636253	-1.061894806	0.00033023	0.001606717
ENSG00000268941	LINC01711	22.50098249	3.143023774	2.875732669	0.000332586	0.001617069
ENSG00000149548	CCDC15	14.10149625	46.14893646	-1.71139788	0.000333543	0.001620981
ENSG00000125885	MCM8	98.21909029	169.17876	-0.783671125	0.000334625	0.00162587
ENSG00000210174	MT-TR	14.11535705	46.42566267	-1.720156202	0.000335649	0.001630096
ENSG00000186567	CEACAM19	34.77902633	86.29417476	-1.316538878	0.000340427	0.001650661
ENSG00000006747	SCIN	42.30410583	91.59286457	-1.110538977	0.000346312	0.001677286
ENSG00000100505	TRIM9	1.628890403	19.83491033	-3.619409582	0.000355201	0.0017168
ENSG00000167536	DHRS13	7.57118023	33.68320748	-2.150828431	0.00036009	0.001738079
ENSG00000272235	AL590438.1	17.24101339	1.234033196	3.914802923	0.000364399	0.001757281
ENSG00000081692	JMJD4	138.6199988	73.56943763	0.916765791	0.000366652	0.001767344
ENSG00000251301	LINC02384	14.42996991	0.468708139	4.783822578	0.000368454	0.001775223
ENSG00000054219	LY75	0.388052291	15.04881713	-5.130740046	0.000369498	0.001779853
ENSG00000279204	AC134043.2	6.768247628	35.28001249	-2.379918002	0.000370459	0.001784077
ENSG00000260641	AC114811.2	20.80927165	57.58969894	-1.466730789	0.000374643	0.00180341
ENSG00000140398	NEIL1	101.2167575	177.5078506	-0.813102168	0.000378455	0.001818464
ENSG00000237515	SHISA9	12.5843859	0.308508299	5.421733983	0.000381593	0.001831889
ENSG00000134443	GRP	0.825064206	18.54877453	-4.524794736	0.000382767	0.001836282
ENSG00000152076	CCDC74B	56.28968662	113.9213667	-1.018540867	0.000383161	0.001837757
ENSG00000198947	DMD	118.8306859	61.15170419	0.960846075	0.000384283	0.001842722
ENSG00000279490	AC109446.4	9.1487628	0	5.927255012	0.000387256	0.001854885
ENSG00000087237	CETP	10.81510023	39.26597781	-1.862828754	0.000390559	0.001869445
ENSG00000188487	INSC	0	10.7725031	-5.600913902	0.000392375	0.001877712
ENSG00000154263	ABCA10	52.45163974	105.8586269	-1.014958322	0.000393353	0.001881969
ENSG00000229720	AL109924.2	30.99514617	7.261084103	2.08839673	0.000394031	0.001884787
ENSG00000236698	EIF1AXP1	102.0187966	172.9842048	-0.762793599	0.000395402	0.001890496
ENSG00000142731	PLK4	51.99790812	108.2030391	-1.06135048	0.000398925	0.001906054
ENSG00000120457	KCNJ5	0.412532103	14.97660904	-5.119534834	0.000399466	0.001908211
ENSG00000117399	CDC20	91.22442365	160.7123923	-0.816950394	0.000401139	0.001915344
ENSG00000230615	AL139220.2	5.960286033	30.16075692	-2.329672533	0.000401842	0.001917838
ENSG00000138483	CCDC54	15.22228351	0.777216438	4.250050505	0.000404816	0.001931599
ENSG00000239474	KLHL41	0.776104581	18.07942159	-4.508384062	0.00040575	0.00193562
ENSG00000185361	TNFAIP8L1	61.90528998	119.5694208	-0.947155149	0.00040688	0.001940575
ENSG00000169981	ZNF35	166.7578226	99.07382043	0.752419868	0.000407753	0.001944304
ENSG00000164611	PTTG1	91.20783172	157.0485578	-0.782259888	0.000407918	0.001944653
ENSG00000091844	RGS17	8.350526615	36.6320884	-2.125888266	0.000408091	0.001945042
ENSG00000224513	AC109309.1	0	10.62635556	-5.59212217	0.00040896	0.001948744
ENSG00000102678	FGF9	1.56606997	19.64812178	-3.631536662	0.000420862	0.002002322
ENSG00000156875	MFSD14A	173.2332061	101.5291356	0.770756025	0.00042788	0.002031619
ENSG00000248996	AC145098.1	17.2654932	1.88088575	3.191537298	0.00043246	0.002052906
ENSG00000139146	SINHCAF	97.4721116	47.78682401	1.025667026	0.000440978	0.002089145
ENSG00000226950	DANCR	120.8263906	204.705448	-0.757278769	0.000441194	0.0020897
ENSG00000153237	CCDC148	7.589176436	32.21308909	-2.083879312	0.000441788	0.002091581
ENSG00000166432	ZMAT1	30.41628438	72.72131421	-1.25965279	0.000447061	0.002115606
ENSG00000156042	CFAP70	11.68912406	42.03325206	-1.846410873	0.000449586	0.002125311
ENSG00000187950	OVCH1	3.565910053	26.7470599	-2.904080697	0.000449612	0.002125311

ENSG00000171724	VAT1L	55.86367662	109.7267647	-0.973870367	0.000451225	0.00213246
ENSG00000154917	RAB6B	70.37032777	130.4904472	-0.892759471	0.000451493	0.002133255
ENSG00000163092	XIRP2	0.412532103	14.60475156	-5.080951392	0.000456401	0.002154527
ENSG00000169247	SH3TC2	13.53884346	45.79459354	-1.757310449	0.000458048	0.002160865
ENSG00000237945	LINC00649	10.5568242	40.72615096	-1.955685181	0.00046269	0.002180829
ENSG00000197978	GOLGA6L9	112.1652588	56.28647838	0.999866001	0.000468837	0.002207507
ENSG00000285748	AC090337.2	0	10.28758151	-5.536878855	0.000469485	0.002209427
ENSG00000108830	RND2	9.997413224	37.87736818	-1.919855685	0.000472039	0.002220955
ENSG00000111981	ULBP1	163.6312727	93.64021814	0.804644663	0.000472919	0.002224601
ENSG00000249609	AC080188.1	14.67527872	0.617016598	4.708869046	0.00047377	0.002227619
ENSG00000003137	CYP26B1	15.86715721	51.91482958	-1.709211039	0.000476566	0.002239282
ENSG00000186481	ANKRD20A5P	42.12899465	12.83801625	1.718632368	0.000479628	0.002252178
ENSG00000144134	RABL2A	89.00191704	152.1409597	-0.774486291	0.000484934	0.002276083
ENSG00000187699	C2orf88	29.33026336	74.2491586	-1.341463963	0.000486546	0.002282644
ENSG00000248429	FAM198B-AS1	63.08928058	118.6640014	-0.913405501	0.000492657	0.002309787
ENSG00000186496	ZNF396	7.103204895	32.95852343	-2.205699474	0.000494361	0.002317263
ENSG00000100558	PLEK2	47.5169025	14.84926588	1.686010454	0.000502444	0.002353075
ENSG00000179859	RNF227	122.1059522	66.97834404	0.863856374	0.000502791	0.002353663
ENSG00000106415	GLCCI1	76.17132873	138.9455683	-0.867093742	0.000503761	0.002357688
ENSG00000167034	NKX3-1	170.3809128	96.85869748	0.816327266	0.000507915	0.002375557
ENSG00000113555	PCDH12	2.397617783	21.97393291	-3.19514931	0.000509881	0.002384229
ENSG00000229689	AC009237.3	42.94974535	109.5845092	-1.35625036	0.00051114	0.002389064
ENSG00000196368	NUDT11	53.33628257	105.3527402	-0.981684814	0.000511798	0.002391615
ENSG00000151773	CCDC122	22.36561621	59.11277952	-1.402036879	0.000512445	0.002393777
ENSG00000266088	AC004585.1	14.35239507	0.617016598	4.676113759	0.000512486	0.002393777
ENSG00000143217	NECTIN4	18.8870064	2.303544486	2.994124152	0.000514862	0.002404346
ENSG00000139899	CBLN3	109.0618618	185.821791	-0.770460724	0.000519507	0.002424444
ENSG00000128683	GAD1	177.1994972	104.7151763	0.757852436	0.000520958	0.002429083
ENSG00000158352	SHROOM4	107.74618	54.620271	0.988271026	0.000524918	0.00244647
ENSG00000264472	AC096708.2	0	10.44951248	-5.564317182	0.000526128	0.0024509
ENSG00000185614	INKA1	52.11864777	112.795849	-1.111504824	0.000541197	0.002515174
ENSG00000259429	UBE2Q2P2	57.80769056	22.28006528	1.372986026	0.000548933	0.002548901
ENSG00000198774	RASSF9	87.92830222	41.86376329	1.066168608	0.000549102	0.002549129
ENSG00000095637	SORBS1	31.50935002	7.521811872	2.0830583	0.000559915	0.00259876
ENSG00000279419	AC004925.1	7.246841968	32.04942699	-2.144975042	0.000561094	0.002603093
ENSG00000167653	PSCA	0.401913099	14.21539104	-5.04896392	0.000563721	0.002613
ENSG00000256713	PGA5	0.412532103	13.95530807	-5.022177333	0.000567502	0.002628807
ENSG00000163491	NEK10	46.48721831	96.61288182	-1.055599015	0.000569085	0.002634991
ENSG00000273445	AC133644.2	18.18344767	1.898830325	3.257146854	0.000576641	0.002665914
ENSG00000274307	AC023449.2	45.40242346	13.52313388	1.746939042	0.000579921	0.002679913
ENSG00000132185	FCRLA	0.401913099	14.05929825	-5.034477248	0.000588377	0.002713681
ENSG00000136014	USP44	0.800584394	16.94245407	-4.398752272	0.000596145	0.002745341
ENSG00000183454	GRIN2A	0.789965389	17.52963471	-4.449965385	0.000596716	0.002747374
ENSG00000006606	CCL26	10.5429634	39.04783719	-1.897455625	0.00059719	0.002748964
ENSG00000183196	CHST6	16.27231211	1.600482278	3.336452339	0.000607106	0.002789174
ENSG00000172137	CALB2	26.52838427	4.611411039	2.5586595	0.000609531	0.002799707
ENSG00000163507	CIP2A	103.8564927	174.6158126	-0.751066801	0.000611013	0.00280591
ENSG00000187758	ADH1A	1.992462881	18.65471086	-3.219746267	0.000611988	0.002809173
ENSG00000105376	ICAM5	18.78584535	1.99092913	3.269775925	0.000629064	0.00288444
ENSG00000255624	AC073585.1	0	9.763093509	-5.467647337	0.000629719	0.002886825
ENSG00000285804	AC093218.1	2.401753181	20.68131413	-3.109052739	0.000630206	0.002887809
ENSG00000184350	MRGPRE	2.397617783	21.18655622	-3.142543898	0.000630597	0.00288898
ENSG00000179930	ZNF648	1.992462881	20.05564189	-3.320339251	0.000630947	0.002889958
ENSG00000132465	JCHAIN	62.22408856	24.83612342	1.33170931	0.000633558	0.002901296
ENSG00000254631	AP001372.1	8.022052954	0	5.736218316	0.000636607	0.002913967
ENSG00000237753	FLJ42351	52.55045258	106.169523	-1.01405848	0.000636737	0.002913967
ENSG00000163638	ADAMTS9	89.74940641	42.05508868	1.092324946	0.00063689	0.00291404
ENSG00000107736	CDH23	4.730066925	28.53368593	-2.583668816	0.000640341	0.002928572
ENSG00000073670	ADAM11	9.588122924	35.50852838	-1.886846898	0.000652254	0.002976652
ENSG00000244558	KCNK15-AS1	3.995544767	23.85828091	-2.581646934	0.000653054	0.002979024
ENSG00000133401	PDZD2	17.58658966	52.3956326	-1.568645	0.000657019	0.002995827
ENSG00000142661	MYOM3	60.92043002	23.74866755	1.370950424	0.000673259	0.003064617
ENSG00000143429	LSP1P4	62.34179121	117.3320312	-0.914159523	0.000674957	0.003071691
ENSG00000143126	CELSR2	60.74207703	23.28385146	1.377904403	0.000676399	0.003076937
ENSG00000004799	PDK4	5.550995733	27.33986395	-2.292748734	0.000678224	0.003083916
ENSG00000179862	CITED4	69.54291535	126.9589227	-0.869689003	0.000681301	0.00309592

ENSG00000223519	KIF28P	7.355380223	35.57836054	-2.287575576	0.000686833	0.003117731
ENSG00000133107	TRPC4	94.42581729	47.1040823	1.001284492	0.000688683	0.003125461
ENSG00000176595	KBTBD11	63.89920747	26.63507061	1.268968465	0.000691183	0.003135466
ENSG00000247416	AP000802.1	8.01143395	0	5.734571998	0.000691526	0.003136353
ENSG00000010319	SEMA3G	2.740845847	23.12775868	-3.060993152	0.000691857	0.003137185
ENSG00000163884	KLF15	25.5726502	72.02213276	-1.487657993	0.00069383	0.003144789
ENSG00000245869	AP004609.1	11.81241672	0.234354069	5.329961619	0.000694455	0.00314628
ENSG00000140557	ST8SIA2	138.0105565	79.19091456	0.79852224	0.000694963	0.003147914
ENSG00000168062	BATF2	4.33139563	25.90563471	-2.566726214	0.000696682	0.003155025
ENSG00000164627	KIF6	60.42562666	112.0696606	-0.890261702	0.000701492	0.003174779
ENSG00000254951	AC044810.2	13.85021451	0.560806944	4.660656604	0.000709349	0.00320829
ENSG00000188501	LCTL	22.70795068	3.978180994	2.502290914	0.000713015	0.003224188
ENSG00000224468	LAMC1-AS1	21.43849736	58.87778065	-1.454981937	0.000720149	0.00325368
ENSG00000007968	E2F2	5.327818524	31.32063588	-2.567233253	0.000721008	0.003256177
ENSG00000015520	NPC1L1	0	9.35427229	-5.400746796	0.000723634	0.003265819
ENSG00000170689	HOXB9	79.56442548	37.6894933	1.075924687	0.000724154	0.003266824
ENSG00000111665	CDCA3	67.20722441	124.5349595	-0.89295866	0.00072498	0.003268561
ENSG00000134242	PTPN22	4.02002458	24.00010639	-2.583481319	0.000726743	0.003275814
ENSG00000169607	CKAP2L	58.20350296	111.4794475	-0.938722739	0.000736335	0.003315537
ENSG00000227964	LINC01429	13.68807055	0.617016598	4.60339629	0.000740894	0.00333465
ENSG00000173267	SNCG	2.828146092	20.50143858	-2.865871944	0.000759015	0.003403963
ENSG00000132832	AL139352.1	0.814445202	15.86019159	-4.303310248	0.000763247	0.003420774
ENSG00000196247	ZNF107	62.52808241	118.140385	-0.918223445	0.000767013	0.003435479
ENSG00000100628	ASB2	19.86594378	2.411856738	3.113101742	0.000768168	0.003439929
ENSG00000175643	RMI2	37.26266786	80.4409462	-1.113114839	0.000792965	0.003545745
ENSG00000227744	LINC01940	14.05991382	43.48219014	-1.632458108	0.000796992	0.003562253
ENSG00000260075	NSFP1	28.68802011	3.7617715	2.927884405	0.000805836	0.003598002
ENSG00000235852	AC005540.1	48.50773538	16.13281074	1.585207063	0.000810105	0.003613268
ENSG00000239415	AP001469.3	12.94147477	43.68151487	-1.758086457	0.000817818	0.003645375
ENSG00000139618	BRCA2	87.27962569	151.526749	-0.79602126	0.000839509	0.003737363
ENSG00000235505	CASP17P	3.194960374	21.28708414	-2.732003217	0.000844351	0.00375813
ENSG00000174010	KLHL15	115.7817108	196.0505491	-0.755217836	0.000845395	0.003761991
ENSG00000133321	RARRES3	3.300256825	25.52297218	-2.972021659	0.000856925	0.003806923
ENSG00000140459	CYP11A1	0.803826197	16.37127186	-4.354909038	0.000871226	0.003866419
ENSG00000161888	SPC24	30.94708014	70.10883163	-1.182665334	0.000885227	0.003924455
ENSG00000055163	CYFIP2	18.02684338	49.55441913	-1.455524166	0.00088791	0.003933893
ENSG00000100784	RPS6KA5	20.15232433	53.62643006	-1.413468422	0.000891227	0.00394612
ENSG00000228914	OR1H1P	19.35822354	53.25133684	-1.464893989	0.000903886	0.003997175
ENSG00000128422	KRT17	17.60782767	2.377698716	2.87208605	0.000926732	0.004093099
ENSG00000178852	EFCAB13	45.24079018	90.73501069	-1.006316928	0.00092892	0.004101056
ENSG00000244257	PKD1P1	44.13437442	12.90784841	1.764454044	0.000937324	0.00413387
ENSG00000172901	LVRN	9.284129076	35.33859808	-1.934857467	0.000939596	0.004143028
ENSG00000184785	SMIM10	66.31609797	120.6382873	-0.864770212	0.000954044	0.004204993
ENSG00000177234	LINC01561	12.81169851	0.795161013	3.987318663	0.000981951	0.004318146
ENSG00000268575	AL031282.2	23.31721488	64.92018519	-1.482742791	0.000983771	0.004325255
ENSG00000248698	LINC01085	1.989221078	18.80539524	-3.231527226	0.000995496	0.00437319
ENSG00000232888	RPS11P5	47.19580604	17.05833563	1.468762159	0.001000103	0.004390705
ENSG00000105639	JAK3	75.82340425	35.72320674	1.088241743	0.001005384	0.004412981
ENSG00000224596	ZMIZ1-AS1	34.81374224	10.09149252	1.802721385	0.001005878	0.004414237
ENSG00000152760	TCTEX1D1	16.27593682	53.34667139	-1.71796808	0.00101073	0.0044337
ENSG00000133302	SLF1	122.1687727	70.14363445	0.801896571	0.00101399	0.004446166
ENSG00000153064	BANK1	72.09675451	33.35934554	1.118817629	0.001014294	0.004446582
ENSG00000091622	PITPNM3	88.2918747	148.4669649	-0.751909423	0.001017009	0.004456057
ENSG00000228242	AC093495.1	31.23631959	69.63233917	-1.157228844	0.00102531	0.004490244
ENSG00000118193	KIF14	90.72693951	155.0963236	-0.774207536	0.001028224	0.004502081
ENSG00000257322	AC138123.1	0	8.831730426	-5.317037268	0.00104148	0.004558246
ENSG00000132623	ANKEF1	3.586254468	22.36978815	-2.637914484	0.001049634	0.004590151
ENSG00000280269	AP000577.1	13.92276036	0.925524897	4.027795358	0.001078301	0.004708735
ENSG00000104848	KCNA7	34.83408666	8.080027877	2.076620089	0.0010956	0.004776429
ENSG00000256195	AP002518.1	26.13206118	5.620605618	2.235158153	0.00109654	0.004779549
ENSG00000233110	AC093797.1	7.53283961	0	5.647592762	0.00111995	0.00487559
ENSG00000285816	AP000944.2	3.992302964	23.46458681	-2.549456517	0.001125054	0.004895885
ENSG00000258311	BLOC1S1-RDH5	14.78527159	47.83589414	-1.69274957	0.001135828	0.004938647
ENSG00000233621	LINC01137	61.63550135	26.32483119	1.236858782	0.001162152	0.005043821
ENSG00000173269	MMRN2	10.45242135	37.27851094	-1.842003187	0.00116646	0.005058388
ENSG00000163673	DCLK3	14.94366306	1.646531681	3.19022127	0.001176379	0.005099202

ENSG00000148985	PGAP2	102.6106256	173.8697334	-0.761960783	0.001176591	0.005099202
ENSG00000264705	AC090337.1	0	8.80124967	-5.313214104	0.001185007	0.005132535
ENSG00000159261	CLDN14	7.187263338	0	5.57853572	0.001194131	0.005167842
ENSG00000056558	TRAF1	77.9886301	136.0926782	-0.804806322	0.001195862	0.005173823
ENSG00000269652	AC011510.1	9.249030259	33.56905704	-1.862502388	0.001196147	0.005173823
ENSG00000172955	ADH6	4.443175688	23.64813962	-2.415963943	0.001203112	0.005199668
ENSG00000140057	AK7	5.58609455	26.95330937	-2.262251037	0.001221249	0.005271256
ENSG00000134245	WNT2B	51.13322679	100.2654813	-0.97325344	0.001225722	0.005288415
ENSG00000101463	SYNDIG1	9.923973787	0.308508299	5.083481048	0.00123988	0.005342995
ENSG00000231889	TRAF3IP2-AS1	53.53497997	103.0993519	-0.947624374	0.001246943	0.005371253
ENSG00000267480	AP001542.3	3.233300994	20.47160263	-2.668306794	0.001250459	0.005385308
ENSG00000143228	NUF2	49.02339384	100.4168105	-1.034092818	0.001253172	0.005394806
ENSG00000213316	LTC4S	24.92073187	5.648710445	2.163200402	0.001256128	0.005404248
ENSG00000214174	AMZ2P1	74.79461366	136.3583728	-0.868314171	0.001262833	0.005427605
ENSG00000232320	AC009299.2	682.1562163	346.1934512	0.97831859	0.001267096	0.005444827
ENSG00000237004	ZNRF2P1	86.35015863	43.57493374	0.985672963	0.00127251	0.005466983
ENSG00000234997	AC016745.1	0	8.478903853	-5.264092201	0.00127665	0.005481446
ENSG00000262691	AC040160.1	19.15265963	49.92433047	-1.379659966	0.001278839	0.005488629
ENSG00000231123	SPATA20P1	17.63879109	2.555843131	2.7461933	0.001283804	0.005506607
ENSG00000251615	AC104825.1	100.8985703	51.80780716	0.967503166	0.001299224	0.005567129
ENSG00000162981	FAM84A	82.97105047	146.9317664	-0.820251314	0.001311664	0.005614478
ENSG00000181284	TMEM102	41.12519456	14.14555887	1.542176025	0.001314018	0.005621458
ENSG00000136237	RAPGEF5	24.23243823	5.003589019	2.290993528	0.001318752	0.005640577
ENSG00000169193	CCDC126	147.9586775	81.25751404	0.859562624	0.001331469	0.00569039
ENSG00000169507	SLC38A11	0	8.579431782	-5.274802158	0.001332497	0.005693638
ENSG00000130032	PRRG3	16.77862807	47.49128191	-1.494858856	0.001352224	0.005773289
ENSG00000238105	GOLGA2P5	9.239304849	33.37339784	-1.857092613	0.001356553	0.005790612
ENSG00000174899	PQLC2L	81.73378673	147.6622887	-0.849704527	0.001373573	0.005862085
ENSG00000264370	MIR3125	7.291666195	0	5.596680499	0.001375374	0.005868595
ENSG00000260230	FRRS1L	1.601168788	17.14091899	-3.41639638	0.001378488	0.0058807
ENSG00000182010	RTKN2	65.20141141	29.79777009	1.133843863	0.001385276	0.005908472
ENSG00000233251	AC007743.1	117.9264645	66.59805744	0.820906658	0.001396253	0.005950519
ENSG00000135476	ESPL1	82.62363667	151.4761625	-0.871663096	0.001409082	0.00600038
ENSG00000163935	SFMBT1	77.32017018	131.4148975	-0.764809305	0.001429845	0.006081489
ENSG00000124839	RAB17	4.725931527	25.4792987	-2.41857031	0.001431507	0.006086124
ENSG00000139890	REM2	0	8.40907169	-5.244322814	0.001435141	0.006099133
ENSG00000154493	C10orf90	28.03487561	7.717900862	1.870390657	0.001453335	0.006169056
ENSG00000160951	PTGER1	7.656132268	32.2470321	-2.080615372	0.001479447	0.006268632
ENSG00000104951	IL4I1	31.68395051	9.254389162	1.778296812	0.001489834	0.006310132
ENSG00000111907	TPD52L1	47.71833101	16.41126807	1.524816139	0.001495904	0.006332055
ENSG00000102886	GDPD3	16.05561851	44.46976288	-1.474471916	0.001498465	0.006341635
ENSG00000233471	KRT18P62	12.69343484	0.749111611	4.009125342	0.001528682	0.006452818
ENSG00000150995	ITPR1	120.138097	63.12058168	0.932489622	0.001529423	0.006454662
ENSG00000142677	IL22RA1	3.593631669	21.1329375	-2.548816486	0.00153222	0.006465185
ENSG00000164342	TLR3	28.48396114	70.60498806	-1.310811969	0.00153842	0.006486196
ENSG00000162641	AKNAD1	1.601168788	16.13043481	-3.33342574	0.001541962	0.006499841
ENSG00000205609	EIF3CL	40.89642734	293.0972995	-2.841637938	0.001554662	0.006545524
ENSG00000165272	AQP3	19.46727248	3.395322418	2.54876412	0.001557829	0.006555027
ENSG00000173227	SYT12	26.20404601	4.515205177	2.585389537	0.001579616	0.006644074
ENSG00000247095	MIR210HG	36.9498422	10.50463581	1.834298362	0.001590897	0.006683591
ENSG00000259583	AC015712.2	43.83921238	87.75024085	-0.998172618	0.00159621	0.006701938
ENSG00000115687	PASK	47.31965971	94.63731807	-1.003155814	0.001597065	0.006704203
ENSG00000228624	HDAC2-AS2	11.3574086	37.98848615	-1.748787084	0.001598238	0.006707804
ENSG00000196220	SRGAP3	3.957204147	22.18039716	-2.473528227	0.001601506	0.006718865
ENSG00000100167	3-Sep	4.342014634	27.22419717	-2.642717536	0.00160659	0.006738868
ENSG00000266714	MYO15B	70.6494589	126.8854248	-0.846396013	0.001616973	0.006777072
ENSG00000232192	KIF26B-AS1	14.33853426	1.121613887	3.700655175	0.00161753	0.00677807
ENSG00000264230	ANXA8L1	12.76363248	0.560806944	4.538827018	0.001618516	0.006780863
ENSG00000152253	SPC25	16.36374775	46.06505177	-1.490936586	0.001623258	0.006796165
ENSG00000188626	GOLGA8M	58.19250105	24.85817505	1.231878786	0.001623877	0.006796165
ENSG00000111305	GSG1	1.164156872	15.4016437	-3.694474584	0.001624086	0.006796165
ENSG00000153044	CENPH	33.96889464	75.56145308	-1.152393736	0.001626851	0.006806395
ENSG00000137959	IFI44L	84.75235943	28.19989049	1.593567169	0.001649583	0.006896073
ENSG00000171241	SHCBP1	116.9602392	198.9365219	-0.764244604	0.001674857	0.006990737
ENSG00000231185	SPRY4-AS1	24.55353469	5.276208168	2.234438183	0.001681758	0.007016785
ENSG00000274565	AC080038.1	138.339413	80.46710489	0.775216255	0.0016938	0.007064257

ENSG00000228536	AL513283.1	7.533733205	32.56137858	-2.110167549	0.001701051	0.007088933
ENSG00000181800	CELF2-AS1	0.388052291	11.37136011	-4.726224592	0.001706118	0.007104483
ENSG00000255346	NOX5	5.543618532	27.21792919	-2.287230495	0.001715585	0.007141104
ENSG00000163449	TMEM169	21.29524319	4.08217118	2.346922434	0.001728479	0.007191962
ENSG00000233237	LINC00472	63.46671386	114.1074987	-0.848846221	0.001740802	0.007240403
ENSG00000234290	AC116366.1	12.39503101	38.09074521	-1.617522253	0.001752776	0.007285928
ENSG00000021852	C8B	9.294748081	0.308508299	4.985281913	0.001759029	0.007309064
ENSG00000241288	AC092902.2	141.7483359	82.91485077	0.76964147	0.001769477	0.007349602
ENSG00000175305	CCNE2	13.56242968	40.64831947	-1.582200357	0.001771699	0.007357396
ENSG00000163701	IL17RE	80.96600328	138.126421	-0.774593373	0.001825256	0.007566504
ENSG00000196932	TMEM26	11.9833925	38.75640214	-1.686618324	0.001829705	0.007580584
ENSG00000244242	IFITM10	36.52255569	12.32498983	1.561874137	0.001848372	0.007651885
ENSG00000215399	HMGB3P7	23.28032887	5.213945319	2.136716182	0.001853407	0.007671238
ENSG00000262246	CORO7	119.9534652	63.77911061	0.907694943	0.001860899	0.007699249
ENSG00000228763	LIMS1-AS1	13.25519403	1.384072784	3.274716726	0.001874792	0.007750697
ENSG00000228918	LINC01344	0	8.178824678	-5.207742945	0.001894611	0.007827341
ENSG00000224743	TEX26-AS1	1.205739296	15.39321458	-3.680357173	0.00193055	0.007962629
ENSG00000119630	PGF	59.86715961	113.9738993	-0.929867648	0.001936876	0.007987173
ENSG00000260466	AC126696.2	7.145680914	0	5.571054181	0.001949384	0.008035633
ENSG00000103021	CCDC113	61.94597881	109.6422352	-0.82267474	0.00195305	0.008049185
ENSG00000231205	ZNF826P	46.05758359	90.42758872	-0.976977914	0.001970362	0.008117387
ENSG00000259062	ACTN1-AS1	22.52635589	5.059798674	2.173808115	0.001986656	0.008179758
ENSG00000115163	CENPA	21.97667033	54.68426498	-1.309184011	0.002003952	0.008241398
ENSG00000256525	POLG2	70.09277903	121.9283152	-0.799587337	0.002006591	0.008249059
ENSG00000158258	CLSTN2	15.6366028	1.937095405	3.029653509	0.002020394	0.008299383
ENSG00000163879	DNALI1	41.88547303	85.46593054	-1.029159816	0.002022203	0.008305213
ENSG00000107338	SHB	150.9581319	87.76711061	0.777777234	0.002026669	0.008317126
ENSG00000274654	AC022467.1	26.54599757	6.219677641	2.116949506	0.002033485	0.008341878
ENSG00000259673	IQCH-AS1	55.37859867	102.8425049	-0.890692388	0.002044383	0.008381736
ENSG00000273230	AC102953.2	44.41197348	90.79986448	-1.034909832	0.002048907	0.008398661
ENSG00000187796	CARD9	103.1973249	51.54384365	0.999725866	0.002049532	0.008399604
ENSG00000260077	AC104794.2	30.67366681	68.40414416	-1.157042835	0.002067913	0.008465145
ENSG00000160201	U2AF1	246.5441957	65.22003785	1.918148322	0.002071732	0.008479147
ENSG00000171931	FBXW10	0.803826197	13.8394265	-4.10869782	0.002072593	0.008479254
ENSG00000143847	PPFIA4	59.33077383	106.0283424	-0.836610017	0.002075939	0.008488197
ENSG00000174004	NRROS	6.456876579	27.41834025	-2.087463236	0.002087551	0.008530751
ENSG00000157150	TIMP4	0.388052291	11.46151278	-4.742187881	0.002100176	0.008572458
ENSG00000124126	PREX1	40.33326387	85.90806171	-1.094941278	0.002101833	0.008575929
ENSG00000272668	AL590560.1	49.68072407	93.70335254	-0.913780948	0.002109138	0.008602433
ENSG00000154478	GPR26	1.992462881	16.0707629	-3.003205675	0.002113996	0.008618942
ENSG00000176826	FKBP9P1	74.52712291	128.6995108	-0.791017012	0.002121329	0.00864221
ENSG00000059378	PARP12	108.3690498	196.4598349	-0.857308006	0.002130732	0.008677193
ENSG00000258433	AC087633.1	0.412532103	10.85660257	-4.659184577	0.002152206	0.008749563
ENSG00000285799	AL645929.2	3.181099566	19.02699812	-2.578918472	0.002153289	0.00875229
ENSG00000183773	AIFM3	1.576688975	15.62173046	-3.292284344	0.002173948	0.008829511
ENSG00000125851	PCSK2	0	7.854102932	-5.15486998	0.002194398	0.008909166
ENSG00000248587	GDNF-AS1	72.81545056	128.1406385	-0.815194373	0.002221998	0.009014334
ENSG00000017427	IGF1	6.435638569	0	5.41773198	0.002236978	0.009071644
ENSG00000174292	TNK1	5.232247482	26.26235333	-2.333074353	0.002253294	0.009132586
ENSG00000182916	TCEAL7	48.2907092	97.35658527	-1.009849039	0.002269355	0.009194174
ENSG00000003249	DBNDD1	35.29971378	73.40405591	-1.059981139	0.002270863	0.00919853
ENSG00000226380	LINC-PINT	38.09510927	76.79570129	-1.009330559	0.002272145	0.009201971
ENSG00000214243	AC004980.2	0	7.968253369	-5.177862551	0.002284527	0.009243315
ENSG00000258096	AC025031.2	20.02019986	3.276849914	2.568507072	0.002299224	0.009295703
ENSG00000260645	AL359715.2	13.28705105	38.6407471	-1.54384342	0.002303839	0.00931259
ENSG00000163617	CCDC191	97.09697619	169.6777341	-0.801250879	0.002329538	0.009410448
ENSG00000180777	ANKRD30B	101.9896707	55.65540926	0.87233085	0.002329818	0.009410448
ENSG00000128262	POM121L9P	13.59842209	1.234033196	3.562690069	0.002368586	0.009554331
ENSG00000064787	BCAS1	40.83271331	79.31781619	-0.957665712	0.002414575	0.009734299
ENSG00000164845	FAM86FP	17.33893264	46.25681869	-1.421645244	0.002423949	0.009770237
ENSG00000232814	COL4A2-AS1	6.379301743	0	5.406807977	0.002425065	0.009772885
ENSG00000261644	AC007728.2	10.47600756	34.25892653	-1.71336865	0.002452231	0.009874875
ENSG00000183569	SERHL2	25.82247732	59.6498037	-1.21127529	0.002453326	0.009877414
ENSG00000164035	EMCN	2.464573614	19.80334324	-3.019800938	0.002456332	0.009887644
ENSG00000260517	AC009093.2	49.98185902	96.49397953	-0.95164847	0.002488995	0.010002081
ENSG00000180044	C3orf80	26.3912308	7.335238333	1.849118615	0.002496676	0.010029158

ENSG00000253194	AL137009.1	5.998626654	25.07890661	-2.057380829	0.002524321	0.010131312
ENSG00000257671	KRT7-AS	13.61138931	1.674636508	3.046472958	0.002544638	0.010208318
ENSG00000064201	TSPAN32	12.81169851	37.08674401	-1.536188362	0.002555578	0.010246406
ENSG00000261087	AP003469.4	4.855707791	22.89124369	-2.24117315	0.002562741	0.010271254
ENSG00000260578	AC110597.1	39.53643197	79.80836095	-1.014775968	0.002586802	0.01036378
ENSG00000131242	RAB11FIP4	0	7.706009481	-5.116268289	0.002590165	0.010370605
ENSG00000280587	LINC01348	6.7045336	0	5.480342213	0.002626518	0.010503242
ENSG00000250091	DNAH10OS	54.06666933	98.26698282	-0.863696613	0.002626552	0.010503242
ENSG00000271880	AGAP11	0	7.533703252	-5.091069172	0.002641485	0.010557004
ENSG00000197933	ZNF823	38.07565845	77.04778492	-1.01694619	0.002643871	0.010562568
ENSG00000248257	PSG10P	0.388052291	10.4579416	-4.599662164	0.00265815	0.010613632
ENSG00000230918	AC008063.1	67.73853086	118.0897868	-0.803864738	0.002659075	0.010615333
ENSG00000158286	RNF207	133.2970815	77.94477498	0.767843638	0.002667718	0.010647837
ENSG00000244301	AOX3P	0	7.752058884	-5.124355672	0.002678653	0.010685465
ENSG00000010318	PHF7	57.32355654	102.7015508	-0.842034786	0.00269041	0.010728339
ENSG00000163694	RBM47	10.03988924	33.29772749	-1.728177302	0.002696724	0.010751498
ENSG00000277496	AL357033.4	5.509413309	25.57550457	-2.204024684	0.002711813	0.01080153
ENSG00000111816	FRK	65.99964759	30.69929722	1.105905228	0.002718952	0.010825908
ENSG00000234233	KCNH1-IT1	10.91715488	0.617016598	4.268751699	0.002719892	0.010827626
ENSG00000025039	RRAGD	24.07767146	55.18475498	-1.199749683	0.002737235	0.010892587
ENSG00000183793	NPIPA5	8.805534737	38.56787096	-2.12296321	0.002755524	0.010957161
ENSG00000174640	SLCO2A1	23.30246048	4.767288813	2.249661747	0.002759339	0.010970281
ENSG00000116771	AGMAT	42.03286259	14.78700303	1.505111642	0.00280443	0.011128739
ENSG00000151575	TEX9	113.1290588	65.10026423	0.803892635	0.002810227	0.011147584
ENSG00000255031	AP002807.1	15.35619517	42.47321057	-1.470460444	0.002810775	0.011147677
ENSG00000267528	AC008991.1	22.35499721	5.390358606	2.050961735	0.002815909	0.011165955
ENSG00000273108	AL121929.2	24.36545629	6.065315986	1.993293557	0.002819567	0.011178376
ENSG00000279117	AP001972.5	20.57274432	54.76166645	-1.406665509	0.002831698	0.011224377
ENSG00000224687	RASAL2-AS1	56.26883152	24.2967233	1.208791267	0.002842947	0.011264765
ENSG00000113196	HAND1	0	7.317293758	-5.048197421	0.002857539	0.01131415
ENSG00000166816	LDHD	89.63420644	44.08342331	1.029529437	0.002862505	0.011331706
ENSG00000226440	AL365214.2	2.828146092	17.79491106	-2.661527922	0.002889454	0.011431999
ENSG00000163462	TRIM46	131.4440699	76.80457194	0.776277844	0.002903684	0.011486162
ENSG00000260593	AC009097.2	10.83220284	0.617016598	4.258082528	0.002940474	0.011626496
ENSG00000251287	ALG1L2	0.825064206	13.20641147	-4.026402839	0.00295225	0.011667425
ENSG00000105516	DBP	30.71055281	68.18448741	-1.147546517	0.002955757	0.011679113
ENSG00000133116	KL	10.85109264	0.617016598	4.259931465	0.002967684	0.011721883
ENSG00000272563	AC016745.2	0.776104581	13.66560415	-4.098328005	0.00297426	0.011745674
ENSG00000258429	PDF	66.03893214	30.71551067	1.107614615	0.002989812	0.011802706
ENSG00000173258	ZNF483	18.82742777	46.19909292	-1.292989779	0.003011905	0.011881101
ENSG00000272476	AL024507.2	33.74750462	10.71845436	1.638485412	0.00302963	0.011948802
ENSG00000179300	RTL3	1.601168788	14.69079717	-3.196212927	0.003043698	0.012002061
ENSG00000276075	AC027682.6	10.3577439	32.61369619	-1.650891051	0.003062885	0.012075479
ENSG00000206384	COL6A6	13.68807055	40.37050693	-1.560526929	0.003064822	0.012080875
ENSG00000257219	LINC02407	24.88563306	6.876690446	1.863535225	0.003094099	0.012182728
ENSG00000084710	EFR3B	70.31863703	118.5617424	-0.753096004	0.003105441	0.012220596
ENSG00000139714	MORN3	13.29029285	37.94200695	-1.513816026	0.003160993	0.012430004
ENSG00000101187	SLCO4A1	0.401913099	10.25406829	-4.569794993	0.003198178	0.012566931
ENSG00000074660	SCARF1	14.84339561	41.93445526	-1.498214208	0.003232081	0.012688425
ENSG00000266043	MIR3649	15.50269114	2.059030165	2.878930201	0.003236194	0.012702226
ENSG00000198848	CES1	34.31804529	11.45156753	1.587255794	0.003248727	0.012744362
ENSG00000271811	Z97200.1	41.04978982	15.82019538	1.3770546	0.003252726	0.012754348
ENSG00000077616	NAALAD2	4.421937679	20.4631735	-2.214176824	0.003286165	0.012881717
ENSG00000162063	CCNF	140.024207	82.65996142	0.761398195	0.003312046	0.012968827
ENSG00000112981	NME5	9.553024107	32.05115812	-1.740578129	0.003323528	0.013006598
ENSG00000285336	AC108734.4	1.579930778	14.3379706	-3.172124786	0.00335249	0.01311075
ENSG00000228061	AC131571.1	14.37452668	1.542541495	3.30970135	0.003435924	0.013421756
ENSG00000164520	RAET1E	3.230059191	17.99748304	-2.488480108	0.003462102	0.013514084
ENSG00000165113	GKAP1	8.854494362	30.47747933	-1.784944238	0.003479918	0.013581132
ENSG00000136231	IGF2BP3	56.01213789	115.322716	-1.038284218	0.003483513	0.013592669
ENSG00000243660	ZNF487	43.0316337	81.63519819	-0.925472908	0.00348933	0.013610371
ENSG00000261490	AC005674.2	102.8913658	58.96447106	0.799771822	0.003505752	0.013669412
ENSG00000012124	CD22	0.412532103	10.11116822	-4.552413909	0.003507921	0.013670431
ENSG00000169282	KCNAB1	80.81123652	41.86873015	0.951435099	0.003521531	0.013720871
ENSG00000120899	PTK2B	57.27224871	100.1154419	-0.804054118	0.003564357	0.01387756
ENSG00000083782	EPYC	16.8340713	2.573787706	2.676553102	0.003574879	0.013913433

ENSG00000185745	IFIT1	223.9878509	539.3797195	-1.267392671	0.003578611	0.013925409
ENSG00000185883	ATP6V0C	171.8065875	94.77113224	0.852076536	0.003582601	0.013938383
ENSG00000091831	ESR1	49.14114681	20.24740881	1.282022215	0.003585509	0.013947143
ENSG00000175785	PRIMA1	1.213116497	13.16209319	-3.447049885	0.00359669	0.013988077
ENSG00000206417	H1FX-AS1	19.56519173	47.10429731	-1.263473427	0.003620076	0.014071309
ENSG00000258667	HIF1A-AS2	22.23170455	4.951486422	2.148496868	0.003670213	0.014250562
ENSG00000034239	EFCAB1	8.788432125	29.79517915	-1.763218668	0.003678624	0.014280612
ENSG00000256897	AC018410.2	6.389920747	24.18905585	-1.919901325	0.003727189	0.014453308
ENSG00000224074	LINC00691	0.388052291	9.752933257	-4.505154221	0.003729536	0.014459772
ENSG00000223695	FO393418.1	10.84047364	0.89742007	3.678568749	0.003754383	0.014548148
ENSG00000187951	AC091057.1	32.10151161	68.83393068	-1.103867152	0.003759019	0.014563459
ENSG00000239332	LINC01119	43.84786609	89.71046248	-1.034667959	0.003769192	0.014597551
ENSG00000277734	TRAC	9.612602737	0.542862368	4.150028447	0.003794726	0.014688416
ENSG00000250682	LINC00491	0	6.990840884	-4.983306673	0.003818363	0.014774528
ENSG00000102385	DRP2	38.88507466	15.01314299	1.38400504	0.003847816	0.014874956
ENSG00000102743	SLC25A15	115.1598623	67.48401614	0.778213787	0.003857214	0.014905868
ENSG00000147576	ADHFE1	10.90888408	36.95875606	-1.767103693	0.00387032	0.014952201
ENSG00000228307	OR2S1P	7.172508935	26.91720521	-1.903977116	0.003882707	0.014996208
ENSG00000200769	RF00019	0	7.269513227	-5.034523384	0.00388715	0.015010641
ENSG00000128965	CHAC1	69.77163224	33.21082208	1.06841107	0.00388979	0.015018106
ENSG00000279873	LINC01126	7.218226757	25.74954192	-1.835342677	0.003899459	0.015049971
ENSG00000159387	IRX6	2.027561699	14.91088393	-2.885996852	0.003959736	0.015256776
ENSG00000280721	LINC01943	40.6082596	14.59394651	1.476181117	0.003960217	0.015256776
ENSG00000245317	AC008393.1	3.586254468	19.46154824	-2.443033777	0.003970731	0.01529338
ENSG00000196656	AC004057.1	69.28749822	125.1746961	-0.854865419	0.003971158	0.01529338
ENSG00000245888	FLJ21408	0	6.992572012	-4.989072333	0.003990443	0.015356523
ENSG00000246203	AL353807.3	34.05384668	69.4126822	-1.030239422	0.004019046	0.015455408
ENSG00000231609	AC007098.1	15.69942323	41.2415533	-1.394438861	0.004046413	0.015555024
ENSG00000225031	EIF4BP7	46.93211811	89.71522607	-0.932685389	0.004052035	0.015571004
ENSG00000180638	SLC47A2	0.776104581	12.17084319	-3.934404936	0.004114015	0.015794904
ENSG00000272750	AL592148.3	24.52206058	54.37316574	-1.148984373	0.004117055	0.015803724
ENSG00000177337	DLGAP1-AS1	65.93542288	112.6923004	-0.776961764	0.004150044	0.015915986
ENSG00000151704	KCNJ1	0	6.944791481	-4.97457545	0.004164089	0.015964093
ENSG00000162598	C1orf87	1.213116497	13.07237032	-3.437643759	0.004165962	0.015968393
ENSG00000279369	AC046185.3	35.27199217	12.591126	1.489905109	0.004183423	0.01602377
ENSG00000189143	CLDN4	8.750091505	33.06576086	-1.92166392	0.004196419	0.016059082
ENSG00000232295	AL589935.1	14.33294425	40.39018263	-1.490118648	0.004200199	0.016070655
ENSG00000235257	ITGA9-AS1	34.28891939	70.36372121	-1.039160071	0.004210626	0.016107654
ENSG00000180611	MB21D2	112.2781105	65.23949877	0.786426649	0.004232501	0.016188422
ENSG00000121577	POPDC2	20.68273719	4.036121777	2.322013343	0.004259657	0.016289358
ENSG00000279236	AC064801.2	1.205739296	13.36834244	-3.47290344	0.004268818	0.016321455
ENSG00000185267	CDNF	12.11551697	35.6605141	-1.559898572	0.004273403	0.01633199
ENSG00000261553	AL137782.1	119.6986594	69.49635188	0.778527005	0.004277569	0.016343157
ENSG00000165202	OR1Q1	4.796129161	20.64974704	-2.10391137	0.004280756	0.016352396
ENSG00000254560	BBOX1-AS1	5.921945412	0	5.301187697	0.004384414	0.016715333
ENSG00000285849	AL021918.4	8.334317598	0.280403472	4.831386779	0.004436366	0.016898245
ENSG00000268655	AC008687.4	19.16886865	3.984234189	2.294100866	0.004448935	0.016940053
ENSG00000178977	LINC00324	10.63439904	37.88038891	-1.840877846	0.004551336	0.017314457
ENSG00000127507	ADGRE2	15.07305642	2.023141015	2.857080206	0.004561497	0.017350008
ENSG00000238103	RPL9P7	87.07367888	151.5797349	-0.797192333	0.004576795	0.017401969
ENSG00000101017	CD40	34.87712369	73.87903225	-1.083347159	0.004580845	0.017414253
ENSG00000164707	SLC13A4	51.46152234	100.576807	-0.971604661	0.004603647	0.01749468
ENSG00000226803	ZNF451-AS1	30.26565301	66.62119562	-1.139820288	0.004624469	0.017564532
ENSG00000258976	AC013451.2	5.58609455	0	5.215092342	0.004624506	0.017564532
ENSG00000106633	GCK	4.418695875	20.54381072	-2.22127631	0.004628228	0.017566115
ENSG00000088827	SIGLEC1	1.213116497	13.08858376	-3.436010232	0.004686642	0.017771962
ENSG00000204936	CD177	8.38976083	27.69679758	-1.720671221	0.004779799	0.01807364
ENSG00000267000	AC016229.1	4.345256438	20.24913994	-2.210958319	0.0048035	0.018153576
ENSG00000143476	DTL	55.98747996	98.7168868	-0.81728106	0.004825092	0.018228699
ENSG00000109771	LRP2BP	77.95263769	41.18059179	0.923187643	0.00483569	0.018264264
ENSG00000188338	SLC38A3	7.585934633	0.308508299	4.694676452	0.004854903	0.018331548
ENSG00000135604	STX11	14.43321171	37.63760571	-1.381901003	0.004856415	0.018334002
ENSG00000245667	AC006064.1	9.616738135	30.02412483	-1.64627155	0.004868216	0.018372027
ENSG00000188396	TCTEX1D4	40.34763536	16.12091936	1.328236456	0.004879269	0.018407208
ENSG00000233554	B4GALT1-AS1	13.84607912	39.80667926	-1.516186483	0.004921622	0.018553817
ENSG00000173535	TNFRSF10C	23.23550464	51.86401681	-1.154511639	0.004965732	0.018710152

ENSG00000138395	CDK15	45.11749752	85.06359231	-0.910605497	0.00498965	0.018790282
ENSG00000090530	P3H2	13.30415366	38.21721704	-1.519518147	0.005007042	0.018845766
ENSG00000100336	APOL4	10.03251204	30.19059287	-1.587741145	0.005032264	0.018933992
ENSG00000233818	AP000695.2	18.36269424	4.218588258	2.144562183	0.005061158	0.019035971
ENSG00000250608	AC010210.1	2.86324491	17.75123759	-2.648512145	0.005075121	0.019081737
ENSG00000256671	LIMS4	5.656292185	0	5.230692691	0.005127899	0.019263142
ENSG00000226012	AP001434.1	12.13027137	1.121613887	3.45459361	0.005132854	0.019274944
ENSG00000269845	AC092364.2	0	6.596286975	-4.896491293	0.005137882	0.01929042
ENSG00000182107	TMEM30B	16.2228418	44.64098278	-1.465281406	0.005153002	0.01934036
ENSG00000205572	SERF1B	35.86019647	91.66331852	-1.34738158	0.005158945	0.01935583
ENSG00000279957	AC110769.3	0	6.529917068	-4.88375181	0.005169087	0.019387039
ENSG00000245571	FAM111A-DT	49.05346366	90.57891791	-0.885438133	0.005210014	0.019530203
ENSG00000257038	AP002761.3	51.93794658	21.0739219	1.283907984	0.005213946	0.019541499
ENSG00000103569	AQP9	47.89941511	19.98862718	1.268333468	0.00522049	0.019562578
ENSG00000014138	POLA2	25.1831433	54.26896054	-1.107336252	0.005256996	0.019682097
ENSG00000213901	SLC23A3	7.612762654	29.11761932	-1.929271947	0.005259632	0.019688432
ENSG00000272970	AC107294.2	23.66927476	6.624391802	1.847020235	0.005275023	0.019741647
ENSG00000189149	CRYM-AS1	7.288424392	26.10777689	-1.84518915	0.005288758	0.019787009
ENSG00000064989	CALCRL	42.58959279	16.01519804	1.424239338	0.00532366	0.019903579
ENSG00000267136	AP005131.1	5.908084605	0	5.298036058	0.005338402	0.019955188
ENSG00000171208	NETO2	97.84668599	55.67810569	0.812751818	0.005396662	0.020162329
ENSG00000171320	ESCO2	23.3251531	53.63421438	-1.201331109	0.005409313	0.020202498
ENSG00000146700	SSC4D	33.67457587	11.03301586	1.610091237	0.005420991	0.020238121
ENSG00000285918	AC092376.3	2.003081886	13.87531565	-2.791522743	0.0054212	0.020238121
ENSG00000145536	ADAMTS16	9.563643112	0.617016598	4.075781404	0.005441596	0.020305231
ENSG00000257438	AC011595.1	17.13985234	2.711935913	2.658628705	0.00544342	0.020306821
ENSG00000281809	LINC01394	0	6.523863873	-4.888035415	0.005463757	0.02037005
ENSG00000257831	AL136418.1	0	6.523863873	-4.888035415	0.005463757	0.02037005
ENSG00000166333	ILK	179.1046869	102.9555803	0.795114845	0.005466979	0.020378492
ENSG00000005249	PRKAR2B	42.33093385	81.31674443	-0.943363112	0.005493326	0.02047311
ENSG00000023445	BIRC3	15.35384696	42.08449485	-1.458645242	0.005499845	0.020493815
ENSG00000186648	CARMIL3	0.803826197	11.50172399	-3.843763633	0.005506123	0.020513613
ENSG00000132517	SLC52A1	8.09962779	28.00941294	-1.797124009	0.005587506	0.020784035
ENSG00000231298	MANCR	3.628730486	18.08958185	-2.325642669	0.005607755	0.020848415
ENSG00000133739	LRRCC1	60.02192637	103.3778095	-0.784166566	0.005615648	0.020870463
ENSG00000258947	TUBB3	32.18842894	11.19926889	1.529057275	0.00565389	0.020994238
ENSG00000162490	DRAXIN	12.3926828	1.430122186	3.150842337	0.005655349	0.020995991
ENSG00000272631	AC067750.1	47.54138231	85.56300772	-0.848015122	0.0056583	0.021003278
ENSG00000110002	VWA5A	52.20308912	92.45807276	-0.823037592	0.005713314	0.021174218
ENSG00000151692	RNF144A	41.07388672	15.87619003	1.356949436	0.005724501	0.021208285
ENSG00000104177	MYEF2	45.75588762	89.14381735	-0.958792525	0.005754524	0.021312085
ENSG00000166920	C15orf48	29.82915178	9.128347345	1.731573275	0.005761907	0.021328285
ENSG00000237596	AL138828.1	0.814445202	11.33568597	-3.808876848	0.005800241	0.021444049
ENSG00000229563	LINC01204	25.13653188	54.16497036	-1.105543549	0.005828419	0.021529508
ENSG00000172927	MYEOV	22.21174304	51.48135428	-1.213713989	0.005838783	0.021560299
ENSG00000267426	AC087289.3	0	6.407982308	-4.852949665	0.005882951	0.021700782
ENSG00000146250	PRSS35	26.27061893	7.443550585	1.844688926	0.005944192	0.02190768
ENSG00000164855	TMEM184A	1.202497492	12.28931569	-3.345424788	0.005963906	0.021965108
ENSG00000174697	LEP	79.58208911	40.22652054	0.992053661	0.006000973	0.022090148
ENSG00000261455	LINC01003	31.32596804	66.76366567	-1.094599299	0.006004275	0.022098476
ENSG00000129173	E2F8	9.309502484	30.90360032	-1.739143459	0.006031621	0.0221876
ENSG00000156509	FBXO43	2.020184497	14.76084434	-2.87184589	0.006043034	0.022225737
ENSG00000181350	LRRC75A	8.431343254	27.89958457	-1.723887396	0.006076017	0.022323877
ENSG00000128918	ALDH1A2	4.068984205	20.07337145	-2.312354756	0.006103244	0.022416163
ENSG00000111405	ENDOU	0	6.317614631	-4.840467917	0.006118681	0.022461221
ENSG00000228816	AK3P5	23.79167382	6.940684424	1.787448111	0.006124572	0.022471209
ENSG00000133119	RFC3	59.46106078	101.5040515	-0.769846891	0.006177757	0.02265071
ENSG00000079335	CDC14A	73.98106205	39.5805393	0.899005128	0.006201496	0.022724353
ENSG00000158869	FCER1G	13.23160781	1.824676096	2.830679442	0.006274777	0.022970784
ENSG00000237410	AP001092.1	2.003081886	14.14620367	-2.815962386	0.00632243	0.023121806
ENSG00000238018	AC093110.1	10.03988924	29.99018182	-1.580667842	0.006322556	0.023121806
ENSG00000206129	AC006305.1	4.820608974	20.13845176	-2.068941908	0.006325543	0.023128752
ENSG00000214814	FER1L6	0.800584394	11.2595856	-3.814372614	0.006371429	0.023280515
ENSG00000124466	LYPD3	7.285182588	26.04248158	-1.84348099	0.006391832	0.023351054
ENSG00000130038	CRACR2A	47.71687639	86.54085022	-0.857756751	0.006439815	0.023518266
ENSG00000261051	AC107021.2	9.196828831	0.82326584	3.493521307	0.00648307	0.023659979

ENSG00000106809	OGN	28.08383523	58.84491223	-1.069285053	0.006493831	0.023695182
ENSG00000214425	LRRC37A4P	66.97444957	113.4016308	-0.757943509	0.006502204	0.023713526
ENSG00000164687	FABP5	64.27429255	31.5649352	1.025971976	0.006514291	0.023753532
ENSG00000234719	NPIPB2	5.515896916	23.69418903	-2.090510328	0.006545621	0.023855502
ENSG00000154102	C16orf74	4.684349103	22.54252417	-2.252217727	0.006585813	0.023977319
ENSG00000183072	NKX2-5	5.233141077	0	5.119166826	0.006602468	0.024033842
ENSG00000185666	SYN3	11.82913642	1.291973979	3.164550333	0.00660971	0.024056087
ENSG00000174628	IQCK	47.90367828	97.00852206	-1.015075824	0.006638896	0.024152122
ENSG00000235641	LINC00484	0.412532103	9.43058744	-4.459849145	0.006648679	0.024181358
ENSG00000186847	KRT14	25.64049963	54.80123287	-1.094817383	0.006650509	0.024183874
ENSG00000164684	ZNF704	30.94853476	65.0455707	-1.072962343	0.006652226	0.024185982
ENSG00000254602	AP000662.1	18.13862344	43.6514639	-1.26981545	0.006735777	0.024468835
ENSG00000225151	GOLGA2P7	18.03422058	4.128220582	2.099503439	0.006787773	0.02464088
ENSG00000196689	TRPV1	39.971146	80.80804031	-1.016365536	0.00682489	0.024754184
ENSG00000151883	PARP8	27.4097853	8.08867201	1.786186505	0.006831658	0.024770585
ENSG00000260966	AP001486.2	11.5699668	33.30961888	-1.521578391	0.006877279	0.024919004
ENSG00000196584	XRCC2	21.1280199	47.82876635	-1.176895384	0.006898589	0.024987701
ENSG00000054277	OPN3	72.82948948	38.82732064	0.908453277	0.006941652	0.025135116
ENSG00000165323	FAT3	0.803826197	11.34347029	-3.815841571	0.006963248	0.02520473
ENSG00000250033	SLC7A11-AS1	4.834469782	19.93630957	-2.053838697	0.006977604	0.025252394
ENSG00000159055	MIS18A	54.49865225	95.7887564	-0.811755123	0.007049332	0.025490289
ENSG00000285793	AC125232.2	17.98939635	45.06861989	-1.329646487	0.007058173	0.025517916
ENSG00000163357	DCST1	0	6.339666264	-4.84998093	0.007097766	0.025648401
ENSG00000131094	C1QL1	2.401753181	15.34305812	-2.676336813	0.007101522	0.025652829
ENSG00000137825	ITPKA	3.583012664	17.06438883	-2.24400815	0.007142485	0.025778903
ENSG00000249859	PVT1	48.78546222	86.63575475	-0.826933442	0.007149714	0.025800613
ENSG00000120337	TNFSF18	7.574422034	26.45455027	-1.798617742	0.007167807	0.025861517
ENSG00000184307	ZDHHC23	41.28090525	80.70124417	-0.967947826	0.007244606	0.026089906
ENSG00000124237	C20orf85	6.145505529	0	5.34878805	0.007302092	0.026270232
ENSG00000106560	GIMAP2	0.814445202	10.58830549	-3.712919711	0.00730868	0.026286712
ENSG00000119121	TRPM6	0	7.079477432	-4.991570527	0.007321137	0.026320937
ENSG00000101098	RIMS4	3.2512972	17.77761129	-2.45949553	0.007324947	0.026330182
ENSG00000108231	LGI1	0.789965389	10.55241634	-3.720869456	0.007381812	0.026521136
ENSG00000260236	AC099778.1	16.79662427	39.90936811	-1.248490499	0.007470532	0.026812704
ENSG00000104313	EYA1	7.871932275	29.06833418	-1.875065815	0.007488379	0.026863153
ENSG00000219736	AL356473.1	10.04313105	0.925524897	3.543215533	0.007494788	0.026881609
ENSG00000261379	AC010735.1	4.837711585	19.73027534	-2.030255167	0.007531604	0.027009101
ENSG00000159905	ZNF221	41.2207656	74.55398963	-0.855162757	0.007550297	0.027067005
ENSG00000179314	WSCD1	1.601168788	12.57318142	-2.971296397	0.007574735	0.027145461
ENSG00000231749	ABCA9-AS1	0.803826197	10.86438689	-3.763198427	0.007578924	0.027155895
ENSG00000168546	GFRA2	0	6.061208929	-4.774639784	0.007583287	0.027163989
ENSG00000162631	NTNG1	16.51711024	39.83716001	-1.271779363	0.007642098	0.027340785
ENSG00000186193	SAPCD2	26.52100707	55.61087597	-1.074348117	0.007643775	0.027342185
ENSG00000116701	NCF2	9.515577081	0.795161013	3.562697552	0.00764709	0.02734944
ENSG00000248487	ABHD14A	36.41491103	70.81730223	-0.960559629	0.007657324	0.027376833
ENSG00000253540	FAM86HP	43.02476718	79.97634511	-0.895076045	0.00766671	0.027405781
ENSG00000184786	TCTE3	17.15281955	43.38684386	-1.341514135	0.007673934	0.027417776
ENSG00000170542	SERPINB9	70.48591065	33.91409929	1.057037027	0.007704332	0.027512548
ENSG00000276012	AL160153.1	28.90565764	10.30963314	1.486879494	0.007704342	0.027512548
ENSG00000262001	DLGAP1-AS2	27.17471259	56.27804926	-1.046439698	0.007706392	0.02751525
ENSG00000141441	GAREM1	39.33594738	72.32524418	-0.878700289	0.007719553	0.027557612
ENSG00000213189	BTF3L4P2	38.70309696	15.97434203	1.270606543	0.007735459	0.027605124
ENSG00000085465	OVGP1	13.89917414	36.69413624	-1.395318581	0.007761308	0.027688077
ENSG00000243137	PSG4	27.96467797	56.55888252	-1.013824797	0.007777466	0.027736409
ENSG00000103269	RHBDL1	28.28342623	58.41382437	-1.045687274	0.007876015	0.028059624
ENSG00000260549	MT1L	26.5944465	8.861566382	1.579827042	0.007927234	0.028204294
ENSG00000285018	AC245140.3	21.63757766	6.397822055	1.77095628	0.007952362	0.028288959
ENSG00000246082	NUDT16P1	10.43442514	29.97655931	-1.520314631	0.007961843	0.028313215
ENSG00000109061	MYH1	14.4308635	1.851049794	3.037592974	0.007976014	0.028354124
ENSG00000279879	AC091152.4	8.515401696	0.542862368	3.972470808	0.007979884	0.02836314
ENSG00000198520	ARMH1	13.84697271	38.4046619	-1.464840267	0.008003133	0.028440638
ENSG00000224081	SLC44A3-AS1	32.75991354	12.17862751	1.436943216	0.008004364	0.028440638
ENSG00000279400	AC008957.3	67.04559113	35.77466454	0.908285525	0.008054744	0.028600528
ENSG00000128578	STRIP2	45.43897689	83.63541626	-0.878080227	0.008102843	0.028752109
ENSG00000238117	AP000844.2	6.897130298	0.234354069	4.554051162	0.00810841	0.028767065
ENSG00000175147	TMEM51-AS1	0	6.235676078	-4.822531823	0.008186182	0.029013935

ENSG00000183134	PTGDR2	1.978602073	13.48681494	-2.752946937	0.008187787	0.029014789
ENSG00000248508	SRP14-AS1	25.48986826	57.28724361	-1.176625343	0.008275643	0.029296823
ENSG00000118997	DNAH7	11.26686655	31.61293073	-1.48805023	0.008317627	0.029425855
ENSG00000101188	NTSR1	0	6.047371411	-4.777024281	0.008367821	0.029583739
ENSG00000157601	MX1	394.6109585	792.4448811	-1.005492903	0.008397947	0.029680377
ENSG00000220392	FCF1P5	0.401913099	8.550682154	-4.313788229	0.008431068	0.029782582
ENSG00000228696	ARL17B	6.432396766	23.26526208	-1.861388964	0.008538484	0.030124904
ENSG00000170477	KRT4	0.401913099	8.094510196	-4.232648039	0.008539309	0.030124904
ENSG00000254726	MEX3A	26.387989	8.699635414	1.617798182	0.008558356	0.030187088
ENSG00000072571	HMMR	40.8073399	78.9390342	-0.948434004	0.008561238	0.030190345
ENSG00000203446	AC004988.1	17.25163239	3.84781711	2.134336257	0.008620402	0.030365639
ENSG00000232830	AC110994.1	8.190680526	0.234354069	4.80845158	0.008676451	0.030527791
ENSG00000179029	TMEM107	34.98007194	68.32350694	-0.969850953	0.008677906	0.030527791
ENSG00000165325	DEUP1	10.00389683	0.98346568	3.270360772	0.008677921	0.030527791
ENSG00000270490	AC110048.1	0	5.911169341	-4.735309425	0.008719559	0.030643844
ENSG00000279220	GPR1-AS	0	5.985323571	-4.751781707	0.008722992	0.030650844
ENSG00000267731	AC005332.2	0.401913099	8.146612793	-4.236049493	0.008735324	0.030684035
ENSG00000234072	AC074117.1	93.585488	50.84747921	0.868852164	0.008736954	0.030684691
ENSG00000163808	KIF15	42.50229254	79.23394324	-0.901477832	0.008767607	0.030761858
ENSG00000235910	APOA1-AS	0	6.097098079	-4.782923706	0.008795772	0.030850496
ENSG00000250397	AP006623.1	36.23941694	68.94722153	-0.925647367	0.008802453	0.030868027
ENSG00000170835	CEL	7.679718485	25.61896303	-1.749517244	0.008823823	0.030923376
ENSG00000238184	CD81-AS1	5.592578157	22.5643608	-2.018918868	0.008895138	0.031142505
ENSG00000164406	LEAP2	0.412532103	8.719096108	-4.350355176	0.008904827	0.031161033
ENSG00000263345	AC006435.2	16.97122476	41.03530406	-1.276274769	0.008909512	0.031170074
ENSG00000279086	AC073130.3	54.69806513	25.87385261	1.077388747	0.008910343	0.031170074
ENSG00000172817	CYP7B1	12.36730939	2.115239819	2.532905649	0.008917809	0.031191062
ENSG00000232653	GOLGA8N	49.89149508	88.8699086	-0.831903903	0.00896208	0.031325334
ENSG00000148426	PROSER2	35.98114092	68.04137234	-0.919981917	0.009108018	0.031793578
ENSG00000228290	TBX18-AS1	0.825064206	11.08252751	-3.782633676	0.009123683	0.031837807
ENSG00000277382	AC005837.3	9.644459751	1.029515082	3.18501347	0.009127546	0.031846058
ENSG00000180938	ZNF572	12.88100255	34.44226433	-1.427091084	0.009186362	0.032040753
ENSG00000183763	TRAIP	22.05010976	48.31476253	-1.13251126	0.00920287	0.032093065
ENSG00000104321	TRPA1	45.12436404	19.3136698	1.234058672	0.009212416	0.032118332
ENSG00000105011	ASF1B	46.55144302	83.82459224	-0.845239007	0.009214647	0.032118332
ENSG00000248265	FLJ12825	15.12201605	38.18241422	-1.328985463	0.009266517	0.032272677
ENSG00000247774	PCED1B-AS1	33.74895923	12.61728469	1.406684506	0.009278908	0.032310537
ENSG00000139832	RAB20	1.590549783	11.85801282	-2.896725526	0.009288075	0.032331869
ENSG00000197233	OR1J2	0	6.04910254	-4.783112845	0.00930137	0.03236755
ENSG00000176771	NCKAP5	53.51323127	93.10211937	-0.802183123	0.00935977	0.032549466
ENSG00000207399	RNU6-1011P	6.400539752	0.308508299	4.449177169	0.009374308	0.032584034
ENSG00000244218	RN7SL81P	55.37949227	26.89342245	1.049656825	0.009441301	0.032790092
ENSG00000273381	AL158071.4	0.412532103	9.102618446	-4.395546008	0.009443246	0.032791489
ENSG00000251538	LINC02201	6.411158757	0.308508299	4.451187004	0.009458072	0.032832251
ENSG00000267277	AC024575.1	0	5.947058492	-4.743904751	0.009486272	0.032924767
ENSG00000266302	AC098850.3	93.41647752	52.49227976	0.82213187	0.009497874	0.032959655
ENSG00000238273	AC108058.1	60.99565664	29.22810423	1.065665642	0.009561146	0.033141359
ENSG00000228459	LINC01546	0	5.784912515	-4.713222659	0.009572324	0.033174696
ENSG00000227028	SLC8A1-AS1	2.391134177	13.74322064	-2.516127487	0.009574507	0.033176854
ENSG00000101447	FAM83D	54.58500857	94.78388366	-0.794883517	0.009590749	0.033216892
ENSG00000129195	PIMREG	40.85898031	78.23835966	-0.934946094	0.009595346	0.033224736
ENSG00000249743	AC116345.1	4.799370965	0	4.99564608	0.009596139	0.033224736
ENSG00000248610	HSPA8P4	6.389920747	0.280403472	4.447158835	0.009617035	0.033280825
ENSG00000162931	TRIM17	15.06008921	39.76905898	-1.393861003	0.009645468	0.033368359
ENSG00000278998	AC099552.4	14.84663741	2.846406855	2.357202841	0.009736001	0.033665127
ENSG00000233452	STXBP5-AS1	57.64733379	29.82155285	0.947857391	0.009744526	0.033689124
ENSG00000176909	MAMSTR	21.74287411	47.02171396	-1.1149086	0.009759118	0.033728604
ENSG00000270898	GPR75-ASB3	5.604090756	0	5.219015661	0.009815684	0.033896554
ENSG00000167798	C3P1	5.604090756	0	5.219015661	0.009815684	0.033896554
ENSG00000268621	IGFL2-AS1	6.463360185	0.234354069	4.46116962	0.009832438	0.033943384
ENSG00000134070	IRAK2	47.62600177	85.00241578	-0.834803109	0.009870575	0.034069506
ENSG00000255203	OR7E2P	10.08884887	1.177823542	3.157037706	0.009924592	0.034228171
ENSG00000091513	TF	1.164156872	11.38757356	-3.261194062	0.009937367	0.034266305
ENSG00000115353	TACR1	15.53868355	3.143023774	2.337146552	0.009943687	0.034277347
ENSG00000244675	AC108676.1	0.401913099	7.861887255	-4.195355397	0.009981136	0.034395286
ENSG00000261727	AC142381.4	4.837711585	0	5.005587799	0.009987216	0.034409284

ENSG00000085999	RAD54L	14.04191762	34.53090088	-1.301542605	0.009994625	0.034419457
ENSG00000198838	RYR3	109.9335374	64.71046218	0.770912814	0.01001607	0.034479712
ENSG00000130270	ATP8B3	49.98706612	88.11862433	-0.814842019	0.010016987	0.034479712
ENSG00000255443	CD44-AS1	6.466601989	23.09338587	-1.837385643	0.010042145	0.034560714
ENSG00000245904	AC025164.1	2.016942694	12.6114465	-2.648032971	0.010077433	0.034659719
ENSG00000138642	HERC6	69.6350398	118.9800793	-0.768954925	0.010117597	0.0347866
ENSG00000267582	AC020916.2	6.393162551	0.308508299	4.447784667	0.01013089	0.034826676
ENSG00000267659	LINC01482	0.412532103	7.894314148	-4.190015409	0.010143695	0.03485942
ENSG00000229848	AC139149.1	32.33620141	11.93648912	1.448417473	0.010244109	0.03515904
ENSG00000231104	AC022395.1	12.30124716	32.89474446	-1.411465918	0.010262268	0.035209995
ENSG00000186838	SELENOV	5.555131131	0	5.207673147	0.010355845	0.035502415
ENSG00000226937	CEP164P1	4.782268353	0	4.991145173	0.010373944	0.035547268
ENSG00000224251	AL391427.1	1.17801768	11.15689675	-3.221450943	0.010505936	0.035953198
ENSG00000257894	AC027288.3	9.25640746	1.103669312	3.080324278	0.010591465	0.036216748
ENSG00000189144	ZNF573	42.75084316	76.1402046	-0.831516397	0.010654366	0.036414262
ENSG00000039068	CDH1	22.19660573	6.111365389	1.854771737	0.010672794	0.036471384
ENSG00000121152	NCAPH	47.14919462	82.55963676	-0.804868248	0.010821722	0.036932818
ENSG00000240687	AC082651.1	0	5.63076587	-4.665098881	0.010841149	0.036992783
ENSG00000235902	AC108472.1	4.880187604	0	5.016605507	0.010887396	0.037114408
ENSG00000061337	LZTS1	27.63944612	10.34789822	1.419890225	0.010906951	0.037164038
ENSG00000230018	PPIAP39	7.28104719	23.98280661	-1.726441149	0.010925148	0.037214112
ENSG00000273240	AC013468.1	0.401913099	7.751843875	-4.176764606	0.010996256	0.037438333
ENSG00000146410	MTFR2	11.75607989	32.60655667	-1.475825299	0.011004124	0.037458853
ENSG00000185920	PTCH1	48.51421898	85.89399767	-0.82264233	0.011005806	0.037458853
ENSG00000183837	PNMA3	0	5.654548631	-4.683646429	0.011014268	0.037481653
ENSG00000230426	ERVMER61-1	12.30448896	34.67833802	-1.484961775	0.011058632	0.037620582
ENSG00000242861	AL591895.1	25.59154	53.66923199	-1.068085394	0.01112193	0.037795007
ENSG00000158528	PPP1R9A	35.70073329	65.52790135	-0.878931256	0.011134562	0.037824424
ENSG00000101695	RNF125	13.61463111	2.131453266	2.741263318	0.011138768	0.037832666
ENSG00000164742	ADCY1	14.67203692	39.45946435	-1.418700038	0.011195117	0.038005839
ENSG00000280077	AL353763.2	42.24815191	74.99459295	-0.830989885	0.011217537	0.038069797
ENSG00000278599	TBC1D3E	7.546700418	26.70813852	-1.813234399	0.011239825	0.038133264
ENSG00000207004	RNU6-301P	19.76751384	5.602661042	1.833569982	0.011293372	0.038296599
ENSG00000232415	ELN-AS1	24.28698786	6.498134975	1.887799557	0.01134494	0.038447821
ENSG00000205670	SMIM11A	6.009245658	25.7257709	-2.097654676	0.01135722	0.038476291
ENSG00000169908	TM4SF1	22.29217677	48.00171738	-1.100931058	0.011409949	0.038619615
ENSG00000138795	LEF1	13.64000452	2.760361245	2.317139896	0.011410423	0.038619615
ENSG00000260329	AC007541.1	7.172508935	23.9028142	-1.730882019	0.011463278	0.038786158
ENSG00000136856	SLC2A8	39.71103246	70.80064726	-0.834545855	0.01154313	0.039037701
ENSG00000140538	NTRK3	0.388052291	7.784270768	-4.171693556	0.011613036	0.039236672
ENSG00000165115	KIF27	53.37462319	90.44336087	-0.757800611	0.011620553	0.039249595
ENSG00000262898	AC139099.2	1.17801768	10.73466781	-3.170859103	0.011661397	0.03937504
ENSG00000198870	STKLD1	10.93839289	31.77745264	-1.539685889	0.011718949	0.039531697
ENSG00000159713	TPPP3	2.041422507	13.51254384	-2.736022857	0.011834376	0.039895752
ENSG00000147234	FRMPD3	17.94278494	4.348952143	2.079566867	0.011935675	0.040199007
ENSG00000188365	AC092171.1	1.650128413	13.15863094	-3.011273765	0.01195936	0.040272396
ENSG00000129682	FGF13	25.56795379	55.05547765	-1.103038763	0.011968365	0.040289958
ENSG00000018625	ATP1A2	0.388052291	7.539756447	-4.125016108	0.012002252	0.04039748
ENSG00000233581	AC069155.1	12.82231751	1.920881958	2.665410718	0.012033941	0.040472265
ENSG00000236829	Z97634.1	11.95891269	30.99937639	-1.372446019	0.012037166	0.040476706
ENSG00000285294	LINC00842	8.028536561	0.542862368	3.889789122	0.012074636	0.040586025
ENSG00000187764	SEMA4D	39.25837254	71.60855946	-0.86431581	0.012075402	0.040586025
ENSG00000184292	TACSTD2	13.24133322	32.53586469	-1.299508655	0.012154178	0.040837884
ENSG00000278784	AL136295.7	17.36989606	40.74798758	-1.238742633	0.012178106	0.040905355
ENSG00000280202	AC005831.1	13.13603677	34.17288092	-1.375517646	0.012369984	0.041505207
ENSG00000175946	KLHL38	27.9854053	8.805356727	1.659555687	0.012370355	0.041505207
ENSG00000133135	RNF128	26.01144931	9.054193115	1.542002236	0.012373363	0.041508746
ENSG00000117480	FAAH	10.46952396	28.35554152	-1.442931573	0.0124283	0.041671058
ENSG00000186409	CCDC30	12.51832367	34.31772713	-1.455120935	0.012429586	0.041671058
ENSG00000225166	AC012462.1	7.687095687	0.588911771	3.777787971	0.012456141	0.041753503
ENSG00000107165	TYRP1	5.988007649	0.308508299	4.353531952	0.012470353	0.041794553
ENSG00000248576	AC023794.1	0.803826197	9.610677992	-3.584997051	0.012496034	0.041867425
ENSG00000186088	GSAP	58.15650864	28.71874335	1.028860388	0.012503512	0.041885878
ENSG00000271204	AC016831.4	4.380355255	18.45083754	-2.067762726	0.012612533	0.042214944
ENSG00000181826	RELL1	68.8155656	121.5696504	-0.819226076	0.012613655	0.042214944
ENSG00000157654	PALM2-AKAP2	31.60816286	12.43914027	1.336426684	0.012664269	0.042364331

ENSG00000275178	AC018529.1	0	5.857335616	-4.723349007	0.012671015	0.042377787
ENSG00000185634	SHC4	38.17771309	15.60205476	1.300137458	0.012672278	0.042377787
ENSG00000280383	Z95331.1	25.96483789	52.04367735	-1.002707124	0.012731413	0.042547197
ENSG00000235703	LINC00894	52.07510004	89.42725306	-0.78406921	0.012759827	0.042623636
ENSG00000280537	AC068946.1	4.883429407	19.6221781	-2.01103625	0.012764859	0.042633748
ENSG00000258777	HIF1A-AS1	10.04637285	1.121613887	3.182532271	0.012777552	0.042662736
ENSG00000168389	MFSD2A	34.85767287	15.03519462	1.217118299	0.012874484	0.042932448
ENSG00000084628	NKAIN1	0	5.352093526	-4.599097236	0.012928808	0.043106839
ENSG00000154839	SKA1	17.73022673	39.95541751	-1.175916889	0.013004099	0.043323903
ENSG00000285700	AC104260.3	0.825064206	10.04176585	-3.632614914	0.013059263	0.043480435
ENSG00000099958	DERL3	8.063635378	0.468708139	3.973257128	0.013068051	0.043502881
ENSG00000105613	MAST1	0	5.594876719	-4.656644059	0.013078308	0.04353021
ENSG00000109819	PPARGC1A	3.614869678	16.67567311	-2.208631909	0.013091334	0.043566747
ENSG00000205002	AARD	21.80982994	6.690116908	1.696538272	0.01310497	0.043605302
ENSG00000132846	ZBED3	32.35565223	60.53900965	-0.899913504	0.013288923	0.044155194
ENSG00000264456	AC138207.4	3.593631669	15.47579793	-2.103666443	0.013340244	0.044298028
ENSG00000157064	NMNAT2	11.55699959	30.49910117	-1.39423129	0.013355313	0.044319157
ENSG00000272791	AC073389.3	6.075307895	0.280403472	4.371328077	0.013357029	0.044319157
ENSG00000180053	NKX2-6	69.40900369	39.64453328	0.80572801	0.013404456	0.044462642
ENSG00000177679	SRRM3	75.88025177	43.7747	0.795566078	0.013462371	0.044626901
ENSG00000084734	GCKR	16.79338247	4.556932513	1.876351366	0.01350802	0.044764265
ENSG00000279930	AL032819.2	0	5.288099549	-4.583121874	0.013533683	0.04482921
ENSG00000148680	HTR7	8.466442071	0.89742007	3.3145197	0.013533943	0.04482921
ENSG00000224691	AL133553.1	7.554077619	0.468708139	3.878327287	0.013557516	0.044893305
ENSG00000283199	C13orf46	43.33238574	20.91999026	1.051100107	0.013584362	0.044975196
ENSG00000255176	AP000941.1	42.33131676	18.8806358	1.161144757	0.013656316	0.045185278
ENSG00000258466	AL049779.1	6.096545904	0.308508299	4.375671151	0.013746214	0.045458164
ENSG00000197497	ZNF665	41.38474709	75.23066663	-0.858642857	0.01375059	0.045458164
ENSG00000147509	RGS20	4.708828916	20.56391644	-2.107927891	0.013770164	0.045505318
ENSG00000261742	LINC00922	4.408076871	0	4.872533996	0.0137843	0.045544954
ENSG00000281026	N4BP2L2-IT2	26.53900327	52.04799941	-0.97229489	0.013874394	0.04580356
ENSG00000184060	ADAP2	1.17801768	10.25169236	-3.099829036	0.013908523	0.045884097
ENSG00000225279	AL121987.1	0	5.276208168	-4.573007615	0.013918132	0.045908677
ENSG00000147697	GSDMC	0	5.259994721	-4.576299541	0.013928622	0.045933038
ENSG00000180881	CAPS2	33.91758681	63.3447755	-0.897795864	0.013929839	0.045933038
ENSG00000147408	CSGALNACT1	22.10409838	48.1478647	-1.126881023	0.013936453	0.045947723
ENSG00000237489	C10orf143	23.30067329	49.02323313	-1.075446993	0.013939649	0.045951134
ENSG00000067606	PRKCZ	3.219440186	14.64064071	-2.184660542	0.013982449	0.046077933
ENSG00000044524	EPHA3	27.03934631	9.042086726	1.575646962	0.014003451	0.046132843
ENSG00000260686	AC008669.1	5.613816166	19.8589081	-1.817210816	0.014103882	0.046427734
ENSG00000267174	AC011472.2	0	5.392089734	-4.615199516	0.014170168	0.046609857
ENSG00000213144	AC084880.1	0.412532103	7.128989091	-4.04703292	0.014193669	0.046672718
ENSG00000266420	RN7SL118P	0.412532103	7.158825047	-4.058541226	0.014272368	0.04686627
ENSG00000213412	HNRNPA1P33	4.457036496	0	4.886341808	0.014364389	0.04712477
ENSG00000282849	AL359834.1	14.07701643	3.321168188	2.095302249	0.01439756	0.047197181
ENSG00000168970	JMJD7-PLA2G4B	25.97724409	53.18518195	-1.031587332	0.014490173	0.047464189
ENSG00000101470	TNNC2	8.721476294	25.58934208	-1.545843672	0.01456652	0.047667524
ENSG00000255690	TRIL	31.52321083	59.04057142	-0.90336867	0.014566829	0.047667524
ENSG00000160808	MYL3	8.980645915	0.588911771	3.999175377	0.014567942	0.047667524
ENSG00000184492	FOXD4L1	0.388052291	7.641800495	-4.15702732	0.014604081	0.047763725
ENSG00000254781	GVINP2	0	5.191893686	-4.552347219	0.014620796	0.047811036
ENSG00000261193	AC134312.4	0	5.213945319	-4.564718731	0.014698858	0.048044137
ENSG00000123095	BHLHE41	100.8154055	58.92749581	0.784194812	0.014744548	0.048186073
ENSG00000204403	CASP12	2.369896167	13.2745125	-2.471555707	0.014762792	0.048238279
ENSG00000277639	AC007906.2	17.28818582	41.68885461	-1.268183595	0.014801963	0.04834707
ENSG00000272789	AC010976.2	7.679718485	0.560806944	3.799820179	0.014816326	0.048383463
ENSG00000182359	KBTBD3	35.23097076	66.18664528	-0.912386055	0.01486387	0.048508917
ENSG00000263327	TAPT1-AS1	19.98096564	42.17896958	-1.075459563	0.015096482	0.049177479
ENSG00000148200	NR6A1	4.802612768	18.68519161	-1.956571025	0.015099591	0.049180072
ENSG00000134317	GRHL1	30.71976754	10.80276884	1.496463424	0.015109903	0.049206121
ENSG00000249628	LINC00942	22.08520858	7.371127483	1.585237594	0.01513822	0.049275695
ENSG00000275367	AC092111.1	2.380515172	12.77769954	-2.412269561	0.015248509	0.049575472
ENSG00000269982	AC018809.2	27.72529175	56.37252399	-1.022561679	0.015296151	0.049690874
ENSG00000253366	AC139272.1	16.98273736	4.304633869	1.974761254	0.015320145	0.049750373
ENSG00000279289	AL136164.3	23.56397831	47.76973924	-1.014933227	0.015321486	0.049750373
ENSG00000232004	CAP1P2	47.20463785	23.25747776	1.012344853	0.015377141	0.049910469

ENSG00000170579	DLGAP1	3.167238758	14.72063313	-2.21017755	0.015413778	0.049989831

Supplementary Table S2: DEG list of the comparision of DHACM + TGFβ1 to TGFβ1 groups

gene_id	gene_name	DHACM_AVG	TGFB_AVG	log2FoldChange	pvalue	padj
ENSG00000196611	MMP1	229607.3967	10505.46929	4.449996198	0	0
ENSG00000149968	MMP3	139746.4451	2059.539358	6.084491533	0	0
ENSG00000011465	DCN	68475.52479	24088.96322	1.507164366	0	0
ENSG00000196924	FLNA	50819.27208	154889.3631	-1.60780714	0	0
ENSG00000112096	SOD2	57114.74611	6953.841833	3.038097368	0	0
ENSG00000196549	MME	30753.64208	12801.64692	1.264420493	0	0
ENSG00000122786	CALD1	23680.73423	66373.01127	-1.486988338	0	0
ENSG00000106624	AEBP1	19353.58447	47431.93404	-1.293325527	0	0
ENSG00000197635	DPP4	23903.12863	7879.117004	1.601219295	0	0
ENSG00000169429	CXCL8	32487.74482	32.92907552	9.945375564	0	0
ENSG00000146674	IGFBP3	1664.589777	65347.34247	-5.294834827	0	0
ENSG00000091986	CCDC80	6313.759363	21531.77915	-1.769901294	0	0
ENSG00000163739	CXCL1	20578.545	32.42715826	9.317079281	0	0
ENSG00000085662	AKR1B1	19413.55265	1006.162745	4.270751012	0	0
ENSG00000185567	AHNAK2	1744.80798	9331.758968	-2.418617765	0	0
ENSG00000115963	RND3	13119.02118	3069.03537	2.095989997	0	0
ENSG00000114251	WNT5A	26042.07285	7445.294498	1.806475533	0	0
ENSG00000166825	ANPEP	16151.75891	4341.064897	1.895791006	0	0
ENSG00000114270	COL7A1	27198.20094	7074.963611	1.942753541	0	0
ENSG00000138119	MYOF	5671.673989	15070.9341	-1.409912867	0	0
ENSG00000198467	TPM2	9231.268991	25118.72001	-1.444382977	0	0
ENSG00000176658	MYO1D	4926.00517	16194.72022	-1.717339513	0	0
ENSG00000129116	PALLD	8553.351173	23822.23306	-1.477928947	0	0
ENSG00000130402	ACTN4	5208.475773	19461.20127	-1.901753	0	0
ENSG00000082781	ITGB5	5790.57959	18439.03091	-1.671305814	0	0
ENSG00000099250	NRP1	9210.514487	2918.918707	1.658104535	0	0
ENSG00000148926	ADM	7292.330898	1458.667919	2.322015419	0	0
ENSG00000134853	PDGFRA	10117.37087	2683.948415	1.914627029	0	0
ENSG00000183255	PTTG1IP	4907.612225	16770.04823	-1.773200077	0	0
ENSG00000013297	CLDN11	4569.403923	66.10693892	6.114774315	0	0
ENSG00000104635	SLC39A14	12261.17958	2611.690238	2.230964183	0	0
ENSG00000125730	C3	12008.94134	199.2741797	5.911750675	0	0
ENSG00000107796	ACTA2	2127.24596	27318.76437	-3.683723603	0	0
ENSG00000102265	TIMP1	3456.643412	9802.802761	-1.503753741	0	0
ENSG00000154856	APCDD1	4018.719459	330.9854192	3.600288673	0	0
ENSG00000108691	CCL2	8022.209221	212.7596282	5.234444835	0	0
ENSG00000122707	RECK	1573.454945	6402.313241	-2.02493634	0	0
ENSG00000063660	GPC1	1923.060256	9192.718484	-2.256392923	0	0
ENSG00000105664	COMP	479.2874546	20492.07706	-5.419219269	0	0
ENSG00000103888	CEMIP	893.0845329	6065.217735	-2.765655407	0	0
ENSG00000079308	TNS1	3321.332486	10798.8974	-1.701056837	0	0
ENSG00000115594	IL1R1	5603.593742	988.8966249	2.502905271	0	0
ENSG00000136153	LMO7	2593.20875	11605.932	-2.161997704	0	0
ENSG00000150687	PRSS23	615.2163502	15475.67281	-4.654039264	0	0
ENSG00000154734	ADAMTS1	12429.05758	1251.373849	3.312313039	0	0
ENSG00000188783	PRELP	146.8134947	3443.042859	-4.557526188	0	0
ENSG00000104415	WISP1	9954.778985	2921.055364	1.768510406	0	0
ENSG00000096696	DSP	2224.945427	13052.34202	-2.552922124	0	0
ENSG00000146457	WTAP	5458.181148	1034.610809	2.399768816	0	0
ENSG00000119280	C1orf198	2562.796577	8862.507871	-1.790062108	0	0
ENSG00000137331	IER3	7934.496614	872.2707613	3.185312138	0	0
ENSG00000105835	NAMPT	4403.951846	481.0426322	3.194614155	0	0
ENSG00000049540	ELN	1398.004426	16631.94235	-3.573007828	0	0
ENSG00000156804	FBXO32	3275.647766	12112.24102	-1.886309134	0	0
ENSG00000106211	HSPB1	1737.85978	8184.625719	-2.236534186	0	0
ENSG00000136244	IL6	6619.566615	536.8552407	3.624083356	0	0
ENSG00000172403	SYNPO2	1248.584195	6451.002752	-2.36959853	0	0
ENSG00000154736	ADAMTS5	6952.436666	941.3434623	2.885014182	0	0
ENSG00000171812	COL8A2	1572.126958	10458.32762	-2.735060808	0	0
ENSG00000162493	PDPN	5728.9964	703.9382967	3.02470841	0	0
ENSG00000170525	PFKFB3	7696.808537	731.6185362	3.394740854	0	0
ENSG00000198542	ITGBL1	945.2667402	7992.498521	-3.080528237	0	0
ENSG00000065320	NTN1	7024.45108	373.0806926	4.236117667	0	0

ENSG00000197632	SERPINB2	2705.810069	101.2735567	4.739584319	0	0
ENSG00000153904	DDAH1	1149.701659	5072.735926	-2.141371515	0	0
ENSG00000144476	ACKR3	6897.538553	1130.159824	2.608909652	0	0
ENSG00000103257	SLC7A5	1338.27213	8035.947632	-2.586850764	0	0
ENSG00000213694	S1PR3	2570.039349	71.94905557	5.159705376	0	0
ENSG00000213626	LBH	568.6772405	5947.542965	-3.38910591	0	0
ENSG00000123610	TNFAIP6	2771.048318	161.6080154	4.099770748	0	0
ENSG00000240583	AQP1	484.5242694	5407.269936	-3.481517918	0	0
ENSG00000173918	C1QTNF1	2961.979465	294.3961154	3.332101533	0	0
ENSG00000115232	ITGA4	199.2651965	3450.634607	-4.117266466	0	0
ENSG00000134802	SLC43A3	2487.302572	114.0418493	4.447730996	0	0
ENSG00000138623	SEMA7A	518.296193	5425.4114	-3.386300557	0	0
ENSG00000110031	LPXN	2803.176291	347.2443023	3.014248108	0	0
ENSG00000137124	ALDH1B1	982.1057029	5362.720826	-2.449903626	0	0
ENSG00000134243	SORT1	531.1299577	3607.432149	-2.762966818	0	0
ENSG00000115129	TP53I3	662.0475282	5059.769574	-2.935150722	0	0
ENSG00000006118	TMEM132A	2766.839403	227.9736545	3.602923659	0	0
ENSG00000198959	TGM2	160.0829654	6431.863711	-5.323871937	0	0
ENSG00000206190	ATP10A	490.9027977	4015.204933	-3.03238998	0	0
ENSG00000145423	SFRP2	2762.050665	205.7097899	3.744824015	0	0
ENSG00000145681	HAPLN1	45.3232742	4032.11097	-6.478616471	0	0
ENSG00000099953	MMP11	51.554413	2475.595683	-5.588200526	0	0
ENSG00000180914	OXTR	36.71149891	2316.606757	-5.988020091	0	0
ENSG00000115461	IGFBP5	28.11031649	2530.299937	-6.508985959	4.91E-308	1.18E-305
ENSG00000163431	LMOD1	227.983197	2201.983442	-3.274490735	4.25E-303	1.01E-300
ENSG00000135111	TBX3	6194.540119	1706.761797	1.859920381	3.24E-301	7.60E-299
ENSG00000100345	MYH9	40209.03455	96008.46398	-1.255657864	6.66E-301	1.54E-298
ENSG00000177469	CAVIN1	12991.21681	28299.61573	-1.123241993	1.19E-298	2.73E-296
ENSG00000172061	LRRC15	27255.86068	12445.23191	1.130958055	1.77E-298	4.02E-296
ENSG00000111799	COL12A1	54376.80532	147605.0002	-1.440717375	1.22E-296	2.74E-294
ENSG00000070404	FSTL3	628.4028714	3186.265845	-2.342089714	6.13E-294	1.36E-291
ENSG00000154122	ANKH	375.9153976	2962.24765	-2.978412646	1.95E-292	4.27E-290
ENSG00000184347	SLIT3	1828.880271	8338.136614	-2.189490747	1.27E-290	2.75E-288
ENSG00000148180	GSN	3748.243559	10382.14117	-1.470102093	1.07E-289	2.30E-287
ENSG00000099194	SCD	3702.34659	15945.58857	-2.107082678	5.68E-288	1.21E-285
ENSG00000101335	MYL9	7992.251783	24236.89992	-1.600647649	2.21E-287	4.66E-285
ENSG00000134871	COL4A2	4933.418744	14068.57651	-1.511731729	2.89E-287	6.02E-285
ENSG00000158008	EXTL1	26.79677581	2269.003493	-6.4184667	1.47E-286	3.03E-284
ENSG00000120885	CLU	388.7117942	2985.227018	-2.943228006	1.76E-284	3.60E-282
ENSG00000138131	LOXL4	518.1407547	3063.109684	-2.564208919	6.29E-284	1.27E-281
ENSG00000173641	HSPB7	112.6196837	1760.248001	-3.969510822	1.66E-281	3.33E-279
ENSG00000118523	CTGF	18709.83024	51979.91641	-1.474248727	1.23E-279	2.44E-277
ENSG00000075624	ACTB	57665.40039	129188.313	-1.163699431	1.23E-275	2.42E-273
ENSG00000148344	PTGES	1863.870466	124.8501696	3.90270835	2.98E-267	5.80E-265
ENSG00000100906	NFKBIA	2432.645541	318.6534776	2.933291565	5.86E-267	1.13E-264
ENSG00000124875	CXCL6	9392.843078	9.09111805	10.00880894	2.47E-266	4.73E-264
ENSG00000142089	IFITM3	1516.200063	5118.023823	-1.754911502	8.98E-266	1.70E-263
ENSG00000090376	IRAK3	1928.234031	209.8977567	3.200295249	2.63E-259	4.93E-257
ENSG00000177606	JUN	4860.484287	1122.321377	2.11539264	1.81E-258	3.36E-256
ENSG00000197614	MFAP5	237.8428171	2417.470986	-3.349030166	1.12E-254	2.06E-252
ENSG00000162576	MXRA8	5646.714863	18639.07121	-1.723004192	2.02E-252	3.70E-250
ENSG00000159674	SPON2	2307.063258	7394.914308	-1.680924275	5.50E-252	9.97E-250
ENSG00000179630	LACC1	2093.061437	274.0379021	2.932965007	3.69E-251	6.63E-249
ENSG00000164574	GALNT10	1498.234407	5252.194159	-1.808941423	3.94E-251	7.03E-249
ENSG00000187498	COL4A1	3089.377821	10758.29843	-1.800361527	6.16E-250	1.09E-247
ENSG00000145934	TENM2	638.9065184	3577.246965	-2.486446762	2.03E-246	3.56E-244
ENSG00000151414	NEK7	2442.134945	8902.449008	-1.865683448	1.92E-245	3.33E-243
ENSG00000092820	EZR	1030.018965	3956.552009	-1.941518298	1.04E-236	1.80E-234
ENSG00000197879	MYO1C	4722.081349	11044.24723	-1.226025034	5.79E-232	9.89E-230
ENSG00000035403	VCL	4338.826301	10273.21229	-1.24355835	4.65E-231	7.89E-229
ENSG00000166741	NNMT	4069.487556	10500.26011	-1.367751874	1.46E-230	2.45E-228
ENSG00000104435	STMN2	61.54039727	1427.876488	-4.538372991	2.92E-230	4.86E-228
ENSG00000067798	NAV3	262.7403623	2319.369202	-3.138259338	6.90E-230	1.14E-227
ENSG00000173546	CSPG4	495.7914119	2897.492657	-2.547004713	9.13E-230	1.50E-227
ENSG00000038427	VCAN	10058.00209	23338.0557	-1.214428587	1.08E-229	1.75E-227
ENSG00000156466	GDF6	80.22519891	1393.895841	-4.112802709	5.30E-226	8.57E-224

ENSG00000156011	PSD3	3729.883771	764.4203128	2.287333376	7.87E-226	1.26E-223
ENSG00000137507	LRRC32	1836.655923	5613.809688	-1.61185317	5.34E-224	8.50E-222
ENSG00000133805	AMPD3	1415.6262	92.9162215	3.929039233	1.05E-222	1.66E-220
ENSG00000101856	PGRMC1	7593.421487	2477.791449	1.615322595	4.48E-222	7.03E-220
ENSG00000204131	NHSL2	126.998692	1610.938231	-3.666061949	9.05E-222	1.41E-219
ENSG00000275832	ARHGAP23	1022.033359	3854.397358	-1.915170153	1.32E-221	2.04E-219
ENSG00000169855	ROBO1	5162.192324	1598.021543	1.691906494	2.08E-220	3.19E-218
ENSG00000101825	MXRA5	8479.918826	25362.80561	-1.580784568	4.86E-219	7.40E-217
ENSG00000164761	TNFRSF11B	7540.820624	2112.333032	1.836162151	6.38E-218	9.63E-216
ENSG00000182718	ANXA2	14588.92942	32162.09101	-1.140517151	7.80E-217	1.17E-214
ENSG00000157214	STEAP2	4333.421893	1065.142441	2.025171081	4.15E-214	6.18E-212
ENSG00000064666	CNN2	4810.850698	11537.3076	-1.262063961	7.97E-214	1.18E-211
ENSG00000163531	NFASC	807.1785351	3077.247702	-1.931458036	1.53E-213	2.25E-211
ENSG00000133816	MICAL2	3841.330842	8806.110115	-1.196901777	5.10E-212	7.44E-210
ENSG00000019549	SNAI2	2984.651734	555.2727288	2.425755683	3.08E-211	4.46E-209
ENSG00000163110	PDLIM5	1134.008623	4282.388432	-1.918051734	9.21E-208	1.32E-205
ENSG00000138135	CH25H	1376.607693	105.5524851	3.705660328	2.01E-207	2.87E-205
ENSG00000125266	EFNB2	178.7077308	1468.810539	-3.039439358	1.05E-201	1.49E-199
ENSG00000169174	PCSK9	21.49021048	1277.491598	-5.892416355	1.39E-200	1.96E-198
ENSG00000180875	GREM2	39.95334019	1131.192337	-4.833857244	2.97E-200	4.16E-198
ENSG00000173210	ABLIM3	2247.242676	437.8256829	2.359189642	1.81E-199	2.52E-197
ENSG00000072952	MRVI1	55.36713205	1160.324032	-4.398913671	3.16E-199	4.36E-197
ENSG00000162849	KIF26B	938.3665269	3477.315866	-1.890469428	3.47E-199	4.76E-197
ENSG00000085117	CD82	1522.35486	147.7142183	3.365921378	1.86E-195	2.53E-193
ENSG00000214517	PPME1	677.8717478	2814.097017	-2.053044969	1.52E-192	2.06E-190
ENSG00000008513	ST3GAL1	3894.666523	1157.635294	1.750615175	2.18E-191	2.93E-189
ENSG00000147883	CDKN2B	241.0602345	1626.288668	-2.756330607	2.35E-190	3.14E-188
ENSG00000112769	LAMA4	1416.249509	4698.765276	-1.731126783	1.27E-189	1.69E-187
ENSG00000144802	NFKBIZ	3354.905879	715.7825617	2.229202813	5.36E-188	7.07E-186
ENSG00000139289	PHLDA1	1843.818492	200.1136039	3.204045081	3.16E-187	4.14E-185
ENSG00000129009	ISLR	1440.180741	4315.17178	-1.583972257	5.01E-187	6.52E-185
ENSG00000178209	PLEC	11244.60529	27869.91989	-1.309561067	6.12E-186	7.92E-184
ENSG00000168542	COL3A1	242810.9505	106694.368	1.186332502	2.65E-185	3.41E-183
ENSG00000072110	ACTN1	12311.65251	23737.88175	-0.947187218	4.81E-184	6.15E-182
ENSG00000196937	FAM3C	4735.154242	1580.372145	1.583463273	1.94E-183	2.46E-181
ENSG00000121440	PDZRN3	3301.593485	910.1655772	1.859026218	2.35E-183	2.97E-181
ENSG00000179820	MYADM	1324.991498	5127.840403	-1.951208456	3.04E-180	3.82E-178
ENSG00000149596	JPH2	806.1417734	2808.150267	-1.801392596	2.90E-176	3.62E-174
ENSG00000013619	MAMLD1	147.0892024	1291.183471	-3.137225557	6.27E-176	7.78E-174
ENSG00000026508	CD44	10752.11436	4547.423885	1.241343538	3.21E-175	3.96E-173
ENSG00000118503	TNFAIP3	1133.704292	80.56744004	3.814183157	5.90E-175	7.23E-173
ENSG00000125398	SOX9	82.22341049	1074.295436	-3.706804223	1.32E-173	1.61E-171
ENSG00000130176	CNN1	245.3530416	1942.878357	-2.986917678	2.69E-173	3.26E-171
ENSG00000177283	FZD8	149.8433702	1274.118782	-3.089157333	8.31E-172	1.00E-169
ENSG00000120708	TGFBI	19414.57562	45624.26199	-1.232653958	9.91E-172	1.19E-169
ENSG00000155324	GRAMD2B	799.748654	3206.020205	-2.00412842	2.83E-171	3.37E-169
ENSG00000074590	NUAK1	576.9508462	2413.436209	-2.066089256	3.63E-171	4.30E-169
ENSG00000075651	PLD1	1150.869046	85.97106941	3.739201139	9.01E-171	1.06E-168
ENSG00000233608	TWIST2	4287.677049	1405.59477	1.608786819	2.09E-170	2.45E-168
ENSG00000116191	RALGPS2	2423.572872	620.9609601	1.964085839	3.05E-169	3.55E-167
ENSG00000167996	FTH1	70409.94933	29801.05074	1.240467682	9.08E-167	1.05E-164
ENSG00000084636	COL16A1	17896.56367	8304.920136	1.107640128	9.16E-167	1.06E-164
ENSG00000140545	MFGE8	3090.941711	7381.772563	-1.256241356	6.67E-166	7.64E-164
ENSG00000138386	NAB1	3200.8006	872.5882384	1.875493514	7.40E-166	8.43E-164
ENSG00000028277	POU2F2	2660.878901	614.6910347	2.114750805	8.70E-166	9.85E-164
ENSG00000146648	EGFR	4041.254578	1377.860292	1.552490934	1.27E-165	1.43E-163
ENSG00000168077	SCARA3	236.4221158	1473.122083	-2.640989075	1.68E-165	1.88E-163
ENSG00000164442	CITED2	1049.458718	3463.639704	-1.721591571	8.45E-165	9.43E-163
ENSG00000163735	CXCL5	11973.91274	4.177841563	11.47483805	8.95E-165	9.93E-163
ENSG00000134013	LOXL2	12826.05775	5386.736391	1.251428194	5.15E-163	5.68E-161
ENSG00000149591	TAGLN	4030.106426	28584.32582	-2.826474083	1.97E-161	2.16E-159
ENSG00000077782	FGFR1	6836.804328	2843.07917	1.265708223	7.60E-161	8.29E-159
ENSG00000184216	IRAK1	1854.445274	5047.900751	-1.443833066	2.88E-160	3.13E-158
ENSG00000138685	FGF2	7280.324795	2659.894719	1.453017077	2.77E-158	2.99E-156
ENSG00000147224	PRPS1	349.8237562	1761.786905	-2.332752947	3.02E-157	3.25E-155
ENSG00000074410	CA12	999.9394829	36.91340537	4.752350151	1.35E-156	1.44E-154

ENSG00000157483	MYO1E	912.5024718	3200.315638	-1.811266419	3.56E-155	3.79E-153
ENSG00000164176	EDIL3	397.5519022	1816.664142	-2.192220238	8.41E-155	8.90E-153
ENSG00000073756	PTGS2	1607.329916	12.55250077	6.993158114	3.13E-151	3.29E-149
ENSG00000111885	MAN1A1	5207.167813	1903.207298	1.451989685	3.34E-151	3.50E-149
ENSG00000006016	CRLF1	34.47464062	842.8436487	-4.607771686	3.98E-150	4.15E-148
ENSG00000138771	SHROOM3	362.4685062	1663.958304	-2.198539481	5.05E-150	5.23E-148
ENSG00000163874	ZC3H12A	1709.432369	324.8682947	2.395565083	1.23E-149	1.27E-147
ENSG00000117519	CNN3	4507.335263	9618.814685	-1.093871258	2.31E-149	2.38E-147
ENSG00000147065	MSN	5982.488522	11591.00991	-0.954197093	4.62E-149	4.72E-147
ENSG00000143515	ATP8B2	2382.945783	5621.11961	-1.238097944	5.18E-149	5.26E-147
ENSG00000008256	CYTH3	712.8467072	2653.236432	-1.897776291	4.37E-148	4.42E-146
ENSG00000007237	GAS7	179.5957769	1209.215904	-2.748966401	2.65E-147	2.66E-145
ENSG00000172757	CFL1	7400.117991	15213.78573	-1.039743273	1.33E-146	1.34E-144
ENSG00000131236	CAP1	2612.439263	6322.656287	-1.275213397	3.01E-145	3.00E-143
ENSG00000203485	INF2	1366.283547	3781.192496	-1.468461071	4.80E-144	4.76E-142
ENSG00000159176	CSRP1	2717.564206	6580.932982	-1.276086788	8.55E-144	8.45E-142
ENSG00000162520	SYNC	312.1108574	1563.016814	-2.322925373	8.74E-144	8.60E-142
ENSG00000143320	CRABP2	2397.872961	607.9607194	1.979154745	1.90E-143	1.86E-141
ENSG00000153823	PID1	1627.037055	350.539008	2.215239717	3.14E-143	3.06E-141
ENSG00000161714	PLCD3	646.6063573	2451.060615	-1.922949862	6.07E-143	5.89E-141
ENSG00000218336	TENM3	1010.140585	3065.036459	-1.602398245	7.51E-143	7.25E-141
ENSG00000167123	CERCAM	3673.652285	7939.621369	-1.112113363	3.68E-142	3.54E-140
ENSG00000162909	CAPN2	5080.826177	9786.736054	-0.945756363	4.15E-142	3.97E-140
ENSG00000184584	TMEM173	273.852925	1420.789243	-2.376522643	1.68E-141	1.60E-139
ENSG00000100644	HIF1A	17547.06779	8064.937173	1.121392693	4.80E-141	4.55E-139
ENSG00000149256	TENM4	61.29565167	817.3316103	-3.740269656	4.76E-139	4.49E-137
ENSG00000174099	MSRB3	2003.588016	4973.834433	-1.311900249	1.19E-138	1.12E-136
ENSG00000154188	ANGPT1	2593.96023	866.3314975	1.581861789	4.24E-138	3.97E-136
ENSG00000095303	PTGS1	1312.968171	230.2709209	2.510841197	7.95E-138	7.40E-136
ENSG00000164111	ANXA5	14300.70372	25426.28829	-0.830149307	3.06E-137	2.84E-135
ENSG00000008710	PKD1	2454.532542	6511.251299	-1.408350432	8.40E-137	7.75E-135
ENSG00000113140	SPARC	68095.05514	150559.4822	-1.14473781	1.37E-136	1.26E-134
ENSG00000196352	CD55	1327.993166	3834.355504	-1.529816178	1.84E-136	1.68E-134
ENSG00000134107	BHLHE40	1020.813431	3144.62392	-1.623886674	6.07E-136	5.52E-134
ENSG00000169715	MT1E	1314.791337	202.7721206	2.698702337	6.53E-136	5.92E-134
ENSG00000163661	PTX3	999.208049	74.17657902	3.745563857	1.92E-135	1.73E-133
ENSG00000196739	COL27A1	2732.136972	749.1817186	1.86695351	3.03E-135	2.72E-133
ENSG00000124831	LRRFIP1	1105.061702	3076.405703	-1.476435029	9.00E-135	8.05E-133
ENSG00000103175	WFDC1	38.10922085	920.0998096	-4.603826549	1.42E-134	1.27E-132
ENSG00000134824	FADS2	3599.183177	8164.346911	-1.182207315	5.60E-134	4.97E-132
ENSG00000123384	LRP1	45788.27769	84827.63237	-0.889625147	1.01E-133	8.93E-132
ENSG00000136802	LRRC8A	823.7487259	2591.749929	-1.654298986	1.54E-133	1.36E-131
ENSG00000109846	CRYAB	568.0704633	2201.651452	-1.955296981	2.46E-133	2.15E-131
ENSG00000095383	TBC1D2	163.016532	1076.214128	-2.722993132	3.17E-133	2.77E-131
ENSG00000197208	SLC22A4	1124.327559	147.6499398	2.931156714	5.89E-133	5.12E-131
ENSG00000101265	RASSF2	116.2814543	1025.503761	-3.144750306	1.75E-132	1.51E-130
ENSG00000105989	WNT2	2477.659141	735.7776597	1.751332911	7.29E-132	6.28E-130
ENSG00000100504	PYGL	323.4781548	1571.531882	-2.281390466	7.86E-131	6.74E-129
ENSG00000223749	MIR503HG	192.5626326	1225.841327	-2.667846958	3.04E-130	2.59E-128
ENSG00000169439	SDC2	1048.363996	3001.542558	-1.518058145	2.84E-129	2.41E-127
ENSG00000136048	DRAM1	7522.624272	3194.977835	1.235259396	2.97E-129	2.50E-127
ENSG00000100234	TIMP3	4196.869694	28351.4139	-2.756134145	4.72E-129	3.97E-127
ENSG00000187764	SEMA4D	800.5833907	34.3586891	4.541662874	1.08E-128	9.03E-127
ENSG00000173442	EHBP1L1	897.6067399	2930.122971	-1.707349212	7.04E-128	5.87E-126
ENSG00000162104	ADCY9	866.8125075	2562.517946	-1.563020458	1.31E-127	1.08E-125
ENSG00000071967	CYBRD1	6486.906972	13488.65655	-1.056315573	1.05E-126	8.70E-125
ENSG00000198121	LPAR1	3420.007744	1197.087682	1.514695196	1.14E-126	9.36E-125
ENSG00000178031	ADAMTSL1	20.88459892	696.4851111	-5.059482307	1.60E-125	1.31E-123
ENSG00000255282	WTAPP1	727.4146559	34.64697473	4.389978343	2.87E-125	2.35E-123
ENSG00000113273	ARSB	1283.793295	3382.973227	-1.397633282	9.98E-125	8.13E-123
ENSG00000173114	LRRN3	2065.543698	636.4042899	1.698560782	1.43E-124	1.16E-122
ENSG00000109436	TBC1D9	2034.582908	559.8506587	1.860602819	7.00E-124	5.66E-122
ENSG00000000971	CFH	2057.485344	414.1549186	2.313921134	2.69E-123	2.16E-121
ENSG00000166341	DCHS1	2015.704877	538.9273724	1.902991209	3.75E-122	3.01E-120
ENSG00000149294	NCAM1	120.1196644	897.6997339	-2.904669182	2.93E-121	2.34E-119
ENSG00000135919	SERPINE2	25733.31813	46661.64525	-0.858670052	3.10E-121	2.47E-119

ENSG00000137809	ITGA11	14608.10897	65032.72942	-2.154455126	7.77E-119	6.16E-117
ENSG00000116132	PRRX1	10463.50858	5428.502261	0.946670274	1.05E-118	8.31E-117
ENSG00000186815	TPCN1	2192.758747	675.1429493	1.699923034	5.41E-118	4.25E-116
ENSG00000145246	ATP10D	569.5035469	1926.168728	-1.758420759	8.12E-118	6.37E-116
ENSG00000172817	CYP7B1	970.8122527	10.82263544	6.480758844	9.78E-118	7.64E-116
ENSG00000125675	GRIA3	3913.010499	1578.50625	1.310212147	1.00E-117	7.80E-116
ENSG00000164694	FNDC1	27223.76959	15662.97616	0.79747109	1.46E-117	1.13E-115
ENSG00000185201	IFITM2	350.2493169	1714.226502	-2.291791941	2.08E-117	1.61E-115
ENSG00000163359	COL6A3	60189.84773	103822.7449	-0.786562662	6.68E-117	5.14E-115
ENSG00000092841	MYL6	10467.42564	21309.91317	-1.025547317	8.22E-117	6.30E-115
ENSG00000198053	SIRPA	1764.84597	3970.195859	-1.169577853	6.95E-116	5.31E-114
ENSG00000213949	ITGA1	6882.992532	2808.935932	1.292940254	2.19E-115	1.67E-113
ENSG00000165029	ABCA1	2314.319927	569.8327954	2.022231746	5.37E-113	4.07E-111
ENSG00000175220	ARHGAP1	2373.796004	4981.802535	-1.069445043	1.23E-112	9.33E-111
ENSG00000123500	COL10A1	9246.372805	16106.94101	-0.800744918	5.89E-112	4.43E-110
ENSG00000182534	MXRA7	2622.423392	6846.818587	-1.384590513	1.85E-111	1.39E-109
ENSG00000173706	HEG1	2469.37484	5596.954451	-1.180616413	2.50E-111	1.87E-109
ENSG00000137312	FLOT1	677.948829	2029.456783	-1.58203814	1.66E-110	1.24E-108
ENSG00000076716	GPC4	17.69965421	622.5478466	-5.131776949	2.88E-110	2.14E-108
ENSG00000081923	ATP8B1	675.5786209	2100.74871	-1.635605074	5.62E-108	4.14E-106
ENSG00000077943	ITGA8	810.4031227	98.36576802	3.043727745	6.06E-108	4.45E-106
ENSG00000128591	FLNC	2987.752546	6700.763749	-1.164684179	8.29E-108	6.06E-106
ENSG00000071575	TRIB2	513.0264139	1804.439561	-1.815494168	4.74E-107	3.46E-105
ENSG00000187134	AKR1C1	1030.954206	175.2008637	2.557257858	1.25E-106	9.06E-105
ENSG00000162892	IL24	977.4852867	8.417567522	6.860036607	1.35E-106	9.75E-105
ENSG00000101608	MYL12A	3356.637621	7786.437523	-1.213904923	1.71E-106	1.24E-104
ENSG00000188452	CERKL	55.29417161	658.3792543	-3.575639822	2.21E-106	1.59E-104
ENSG00000140511	HAPLN3	74.47267957	706.3571344	-3.248699617	2.67E-106	1.91E-104
ENSG00000176014	TUBB6	2136.852781	5539.342036	-1.373686519	3.06E-106	2.19E-104
ENSG00000182957	SPATA13	1351.852736	346.9971665	1.962501444	4.12E-106	2.93E-104
ENSG00000143631	FLG	119.0023989	832.4024207	-2.808083394	8.04E-106	5.70E-104
ENSG00000121989	ACVR2A	158.8742582	986.1992755	-2.633901298	1.99E-105	1.41E-103
ENSG00000103647	CORO2B	76.9387532	760.0516248	-3.300688969	4.64E-105	3.27E-103
ENSG00000123975	CKS2	845.8461729	101.5214346	3.057801859	1.14E-104	8.00E-103
ENSG00000149948	HMGA2	987.2359767	124.9900121	2.984888199	2.06E-104	1.44E-102
ENSG00000172493	AFF1	3197.539571	1136.95752	1.492529368	1.06E-103	7.36E-102
ENSG00000110237	ARHGEF17	2249.720615	4910.679134	-1.126745286	1.95E-103	1.36E-101
ENSG00000183715	OPCML	47.87585629	657.737827	-3.772032078	2.62E-103	1.82E-101
ENSG00000183087	GAS6	9784.866601	44464.36854	-2.184092636	4.77E-103	3.29E-101
ENSG00000126603	GLIS2	436.2192452	1811.519442	-2.056532609	6.02E-103	4.14E-101
ENSG00000126803	HSPA2	352.5213765	1311.965349	-1.896434923	7.93E-103	5.44E-101
ENSG00000100439	ABHD4	593.7901783	1860.78898	-1.648777707	8.80E-103	6.02E-101
ENSG00000137501	SYTL2	745.6529417	2179.706319	-1.547456766	9.30E-103	6.34E-101
ENSG00000164733	CTSB	14511.27041	24896.14212	-0.778806269	1.23E-102	8.33E-101
ENSG00000166920	C15orf48	630.0217978	26.10809396	4.58498648	2.40E-102	1.63E-100
ENSG00000075275	CELSR1	10.48012988	665.7805502	-5.974989081	3.37E-102	2.27E-100
ENSG00000139641	ESYT1	1185.925896	2937.525567	-1.308030651	9.09E-102	6.11E-100
ENSG00000078401	EDN1	652.117016	50.62125401	3.683561894	2.97E-101	1.99E-99
ENSG00000023191	RNH1	1871.926314	4112.953909	-1.135634224	5.21E-101	3.48E-99
ENSG00000102359	SRPX2	3605.165511	1479.59667	1.284551435	6.00E-101	4.00E-99
ENSG00000152377	SPOCK1	3192.365194	6457.66261	-1.016366752	1.33E-100	8.86E-99
ENSG00000013364	MVP	3009.87346	6384.256986	-1.084618384	6.27E-100	4.15E-98
ENSG00000151790	TDO2	691.9607458	33.057694	4.380562359	6.33E-100	4.18E-98
ENSG00000052795	FNIP2	458.9060512	1644.288547	-1.839781178	7.33E-100	4.82E-98
ENSG00000163131	CTSS	600.5201412	34.22015829	4.139805013	1.66E-99	1.09E-97
ENSG00000088448	ANKRD10	3383.991907	1365.965837	1.308560335	2.52E-99	1.65E-97
ENSG00000160789	LMNA	8623.97846	16383.43608	-0.925819059	2.94E-99	1.92E-97
ENSG00000101160	CTSZ	4960.635444	8994.151588	-0.858488413	3.66E-99	2.38E-97
ENSG00000173402	DAG1	1530.332474	3778.802309	-1.304533214	5.49E-99	3.55E-97
ENSG00000112902	SEMA5A	3189.408241	6352.573806	-0.994100133	7.50E-99	4.84E-97
ENSG00000175274	TP53I11	616.3774697	1794.188974	-1.541746844	1.72E-98	1.11E-96
ENSG00000187955	COL14A1	9.328852799	657.8113372	-6.136801048	1.81E-98	1.16E-96
ENSG00000160752	FDPS	863.3061834	2242.052349	-1.377044407	7.46E-98	4.77E-96
ENSG00000042062	RIPOR3	1909.000042	34.2255162	5.805583155	2.35E-97	1.50E-95
ENSG00000072682	P4HA2	4350.647276	2050.542413	1.085530131	2.94E-97	1.87E-95
ENSG00000130164	LDLR	2175.369384	5374.095597	-1.30551631	3.44E-97	2.18E-95

ENSG00000249992	TMEM158	1087.452956	185.1343697	2.556916057	3.64E-97	2.30E-95
ENSG00000136068	FLNB	1146.316553	2810.730933	-1.294854805	5.06E-97	3.18E-95
ENSG00000183688	RFLNB	2856.712975	5464.355899	-0.935375567	2.64E-96	1.66E-94
ENSG00000115380	EFEMP1	41.23412521	642.6334431	-3.964469578	2.82E-96	1.77E-94
ENSG00000102007	PLP2	388.6187434	1382.346858	-1.830903292	3.25E-96	2.03E-94
ENSG00000171444	MCC	1211.234371	276.864535	2.128590704	1.13E-95	7.01E-94
ENSG00000073712	FERMT2	2126.124258	4418.118577	-1.055678094	1.77E-95	1.10E-93
ENSG00000082497	SERTAD4	42.21631751	578.346834	-3.771885044	1.83E-95	1.13E-93
ENSG00000189184	PCDH18	1442.94242	3333.035445	-1.207777637	1.93E-95	1.19E-93
ENSG00000107562	CXCL12	3856.319097	1452.41167	1.40825298	3.63E-95	2.23E-93
ENSG00000031081	ARHGAP31	682.6913136	1965.545925	-1.526724876	6.32E-95	3.88E-93
ENSG00000071127	WDR1	4969.048025	9329.685389	-0.908593816	1.21E-94	7.40E-93
ENSG00000110900	TSPAN11	653.3738666	36.55012538	4.161940616	1.78E-94	1.09E-92
ENSG00000162144	CYB561A3	1638.272239	531.6946768	1.622808852	4.98E-94	3.02E-92
ENSG00000122863	CHST3	900.4097031	2551.361023	-1.503930273	6.11E-94	3.69E-92
ENSG00000162630	B3GALT2	11.39967576	559.130707	-5.63609546	8.25E-94	4.97E-92
ENSG00000002586	CD99	4695.176502	8502.56094	-0.856848868	1.40E-93	8.41E-92
ENSG00000188393	CLEC2A	22.24884294	510.0497254	-4.508921727	2.05E-93	1.23E-91
ENSG00000163673	DCLK3	609.6492019	13.08381464	5.552727146	2.15E-93	1.28E-91
ENSG00000172216	CEBPB	5129.271512	2390.955994	1.100953491	4.60E-93	2.74E-91
ENSG00000164292	RHOBTB3	3291.872709	1463.015574	1.169827191	1.27E-92	7.55E-91
ENSG00000102024	PLS3	2701.049485	5386.898942	-0.996197371	7.84E-92	4.63E-90
ENSG00000170390	DCLK2	294.4720601	1272.798852	-2.112012709	1.38E-91	8.08E-90
ENSG00000115884	SDC1	4814.278735	10282.24356	-1.095206795	1.69E-91	9.89E-90
ENSG00000143369	ECM1	2249.733206	4550.097036	-1.016342532	2.88E-91	1.68E-89
ENSG00000135048	CEMIP2	4773.296994	2263.143353	1.076983636	3.02E-91	1.75E-89
ENSG00000175183	CSRP2	1159.238871	2774.944258	-1.260224294	3.31E-91	1.91E-89
ENSG00000143162	CREG1	2222.643625	846.7138882	1.392202121	7.63E-91	4.40E-89
ENSG00000128656	CHN1	897.2653081	2489.145028	-1.472675044	1.11E-89	6.37E-88
ENSG00000008441	NFIX	3867.750554	7335.877433	-0.923697357	1.75E-89	1.00E-87
ENSG00000115468	EFHD1	17.64822861	526.2803689	-4.910913372	3.33E-89	1.90E-87
ENSG00000148082	SHC3	623.0828943	11.2908146	5.793869448	3.50E-89	2.00E-87
ENSG00000111859	NEDD9	187.1487221	895.508404	-2.256807968	4.85E-89	2.76E-87
ENSG00000115738	ID2	1642.331152	466.0894905	1.817948291	5.36E-89	3.04E-87
ENSG00000170017	ALCAM	667.5344233	2040.920337	-1.612474715	5.59E-89	3.16E-87
ENSG00000197324	LRP10	6041.578313	10562.66567	-0.8060729	8.04E-89	4.53E-87
ENSG00000050405	LIMA1	4598.078152	8920.617077	-0.956362512	9.00E-89	5.06E-87
ENSG00000112378	PERP	278.4410868	1112.140075	-1.997384175	1.06E-88	5.92E-87
ENSG00000107957	SH3PXD2A	11801.59554	20859.47929	-0.821942067	1.34E-88	7.47E-87
ENSG00000168621	GDNF	929.1387276	197.7365758	2.232634295	2.43E-88	1.35E-86
ENSG00000154380	ENAH	2555.395582	4988.907638	-0.9651379	2.84E-88	1.58E-86
ENSG00000099860	GADD45B	1113.201871	2866.516377	-1.364337768	4.22E-88	2.34E-86
ENSG00000110427	KIAA1549L	655.6226219	1918.986632	-1.548641562	1.26E-87	6.98E-86
ENSG00000169018	FEM1B	1525.297694	3394.741987	-1.15443314	1.34E-87	7.37E-86
ENSG00000120437	ACAT2	212.8089345	1023.429448	-2.266980805	1.67E-87	9.17E-86
ENSG00000136848	DAB2IP	741.9095759	2205.858489	-1.572835486	3.36E-87	1.84E-85
ENSG00000150938	CRIM1	4037.596102	8207.208602	-1.023248494	1.45E-86	7.91E-85
ENSG00000177697	CD151	1813.872347	3924.754108	-1.113753794	2.68E-86	1.46E-84
ENSG00000166750	SLFN5	5522.023903	2796.165598	0.981697052	1.45E-85	7.90E-84
ENSG00000004399	PLXND1	3562.888684	7344.246425	-1.044187629	2.99E-85	1.62E-83
ENSG00000164603	BMT2	1898.557941	689.9170701	1.46033497	5.87E-85	3.18E-83
ENSG00000049130	KITLG	2021.496714	777.8685512	1.378239437	9.90E-85	5.35E-83
ENSG00000211455	STK38L	699.3892229	2014.150643	-1.527315918	3.58E-84	1.93E-82
ENSG00000111186	WNT5B	1877.299104	3824.750654	-1.026282752	6.33E-84	3.40E-82
ENSG00000128342	LIF	121.7395017	813.7584007	-2.743736847	1.02E-83	5.44E-82
ENSG00000065308	TRAM2	4414.890192	7921.098547	-0.843304482	1.36E-83	7.27E-82
ENSG00000138821	SLC39A8	1484.267881	364.0964661	2.027910987	1.64E-83	8.77E-82
ENSG00000131459	GFPT2	929.2179715	142.7353516	2.703129491	1.94E-83	1.03E-81
ENSG00000130066	SAT1	1744.489897	576.5509929	1.598298463	1.47E-82	7.82E-81
ENSG00000167779	IGFBP6	270.1865001	1057.546729	-1.96948509	1.67E-82	8.82E-81
ENSG00000108840	HDAC5	833.4564867	2036.935537	-1.289550232	2.04E-82	1.08E-80
ENSG00000197321	SVIL	2078.350843	732.7928439	1.503621301	3.94E-82	2.07E-80
ENSG00000127946	HIP1	928.8364574	2251.547253	-1.276832604	2.60E-81	1.36E-79
ENSG00000107731	UNC5B	2089.044322	4030.428366	-0.948417684	3.26E-81	1.70E-79
ENSG00000182871	COL18A1	1064.391099	2564.711253	-1.268674056	5.10E-81	2.66E-79
ENSG00000275342	PRAG1	20.36989548	447.3843896	-4.446086459	7.65E-81	3.98E-79

ENSG00000108821	COL1A1	514165.4762	891005.2514	-0.793205631	3.56E-80	1.85E-78
ENSG00000143774	GUK1	1227.033944	2789.487168	-1.185159098	3.75E-80	1.94E-78
ENSG00000150594	ADRA2A	77.66067374	574.8291749	-2.891488849	4.56E-80	2.35E-78
ENSG00000100243	CYB5R3	4862.191195	8788.354029	-0.853998849	5.29E-80	2.72E-78
ENSG00000140937	CDH11	6672.070493	11815.75139	-0.824692281	5.75E-80	2.95E-78
ENSG00000139329	LUM	29008.9402	49736.4294	-0.777869078	2.28E-79	1.17E-77
ENSG00000244486	SCARF2	974.4290739	5898.617589	-2.598271217	6.04E-79	3.08E-77
ENSG00000079931	MOXD1	7223.337018	4046.942536	0.835527794	6.55E-79	3.33E-77
ENSG00000144749	LRIG1	1856.894766	615.4890269	1.59199877	2.13E-78	1.08E-76
ENSG00000115641	FHL2	781.3909533	1956.772747	-1.324023989	3.26E-78	1.65E-76
ENSG00000101439	CST3	1493.398089	3406.1994	-1.190469916	3.79E-78	1.91E-76
ENSG00000068366	ACSL4	4757.618143	2016.17068	1.238598679	7.33E-78	3.69E-76
ENSG00000149451	ADAM33	5570.249318	2568.999022	1.116528725	8.73E-78	4.39E-76
ENSG00000137267	TUBB2A	424.2603518	1321.274903	-1.640171048	1.24E-77	6.21E-76
ENSG00000177425	PAWR	788.3205981	2156.977038	-1.45339327	1.29E-77	6.43E-76
ENSG00000159251	ACTC1	75.14480358	1565.371878	-4.380727668	1.45E-77	7.21E-76
ENSG00000082438	COBLL1	85.82146333	574.9717069	-2.745174489	1.57E-77	7.80E-76
ENSG00000087495	PHACTR3	17.86191782	427.6811524	-4.587547635	2.87E-77	1.43E-75
ENSG00000185070	FLRT2	5873.976012	2945.743034	0.995830365	1.72E-76	8.50E-75
ENSG00000172348	RCAN2	83.98730895	638.0966872	-2.92958278	1.18E-75	5.85E-74
ENSG00000155254	MARVELD1	2588.039956	4790.75062	-0.888856265	3.02E-74	1.49E-72
ENSG00000182580	EPHB3	197.2628641	905.9737693	-2.198350345	7.06E-74	3.46E-72
ENSG00000137309	HMGA1	2033.461053	794.2363106	1.356976408	7.17E-74	3.51E-72
ENSG00000177707	NECTIN3	1482.659792	476.3854488	1.638425122	9.03E-74	4.41E-72
ENSG00000120594	PLXDC2	711.014248	1925.780406	-1.438412524	1.77E-73	8.59E-72
ENSG00000109066	TMEM104	532.6805821	1453.073529	-1.448009124	2.55E-73	1.23E-71
ENSG00000166311	SMPD1	1199.559294	2591.970263	-1.112170775	3.06E-73	1.48E-71
ENSG00000172379	ARNT2	261.5168512	1005.04772	-1.942343624	1.14E-72	5.49E-71
ENSG00000172986	GXYLT2	309.6323029	1150.155926	-1.891757287	1.31E-72	6.33E-71
ENSG00000197594	ENPP1	228.6645637	980.8351517	-2.102788031	1.41E-72	6.75E-71
ENSG00000169047	IRS1	2448.110787	1018.620115	1.265773372	1.92E-72	9.18E-71
ENSG00000104936	DMPK	519.4472195	1599.259092	-1.622543201	2.59E-72	1.24E-70
ENSG00000114923	SLC4A3	16.84929705	419.5681944	-4.639574603	3.56E-72	1.70E-70
ENSG00000124126	PREX1	465.6704732	35.31732762	3.727918837	4.31E-72	2.05E-70
ENSG00000169184	MN1	342.7036604	1122.706171	-1.712163392	5.54E-72	2.63E-70
ENSG00000170485	NPAS2	793.5388331	167.4696163	2.244881996	6.33E-72	3.00E-70
ENSG00000124942	AHNAK	26557.1271	49081.12157	-0.886055104	6.51E-72	3.08E-70
ENSG00000178104	PDE4DIP	3254.734861	1676.080221	0.957389793	7.34E-72	3.46E-70
ENSG00000163171	CDC42EP3	941.2050564	2208.066123	-1.229482815	8.21E-72	3.86E-70
ENSG00000157240	FZD1	691.7485093	1732.224343	-1.324799238	1.00E-71	4.70E-70
ENSG00000148677	ANKRD1	9.047674137	414.8487328	-5.519944755	1.38E-71	6.48E-70
ENSG00000167772	ANGPTL4	902.7472576	4.16422339	7.746387404	1.48E-71	6.91E-70
ENSG00000106772	PRUNE2	269.0491242	2588.412331	-3.268023489	2.10E-71	9.80E-70
ENSG00000112972	HMGCS1	1561.006677	3578.641319	-1.197459079	9.74E-71	4.53E-69
ENSG00000231991	ANXA2P2	1184.605217	2667.221566	-1.170721077	1.41E-70	6.55E-69
ENSG00000159840	ZYX	4016.556504	6975.686009	-0.796342788	2.74E-70	1.27E-68
ENSG00000188042	ARL4C	2331.729256	1037.524858	1.168253141	3.50E-70	1.62E-68
ENSG00000182752	PAPPA	6454.161796	1033.742377	2.642652043	5.47E-70	2.52E-68
ENSG00000168398	BDKRB2	413.5247189	20.47766161	4.343682946	6.42E-70	2.95E-68
ENSG00000060718	COL11A1	616.426785	3834.182618	-2.637242752	7.54E-70	3.46E-68
ENSG00000130309	COLGALT1	2604.686045	4830.075004	-0.891101089	9.45E-70	4.32E-68
ENSG00000072163	LIMS2	330.4993074	1276.450274	-1.952270407	3.03E-69	1.38E-67
ENSG00000077942	FBLN1	3102.412674	5889.842976	-0.925501498	4.72E-69	2.15E-67
ENSG00000176720	BOK	367.4334793	1138.393157	-1.63240426	4.98E-69	2.26E-67
ENSG00000121769	FABP3	47.5593159	454.7334874	-3.259984988	2.00E-68	9.06E-67
ENSG00000131016	AKAP12	774.9187786	1952.098216	-1.333895694	3.40E-68	1.54E-66
ENSG00000164647	STEAP1	1731.718881	718.6977379	1.268508582	6.43E-68	2.90E-66
ENSG00000073921	PICALM	2938.035371	5305.107957	-0.852577053	6.97E-68	3.13E-66
ENSG00000104881	PPP1R13L	426.0465315	1459.575434	-1.77716417	7.65E-68	3.43E-66
ENSG00000118515	SGK1	1961.476094	3894.507699	-0.989052672	1.83E-67	8.17E-66
ENSG00000162772	ATF3	391.53559	16.72869028	4.541706179	1.95E-67	8.69E-66
ENSG00000018408	WWTR1	986.7484436	2306.257137	-1.224653993	2.98E-67	1.32E-65
ENSG00000106034	CPED1	1587.757723	526.8066521	1.592621471	3.74E-67	1.65E-65
ENSG00000150347	ARID5B	2822.269416	1345.737599	1.068198683	6.32E-67	2.78E-65
ENSG00000123643	SLC36A1	554.8133749	1521.621418	-1.456771779	6.49E-67	2.85E-65
ENSG00000115363	EVA1A	1574.547755	542.6268482	1.53616529	1.19E-66	5.21E-65

ENSG00000005884	ITGA3	464.4274714	1332.753613	-1.520709779	1.49E-66	6.53E-65
ENSG00000172893	DHCR7	829.2069137	2263.446369	-1.450160205	4.41E-66	1.92E-64
ENSG00000125753	VASP	877.0333708	2035.03908	-1.214415289	5.26E-66	2.29E-64
ENSG00000174348	PODN	337.3234206	3975.329325	-3.560079486	1.66E-65	7.21E-64
ENSG00000021762	OSBPL5	489.1935347	1333.634301	-1.446346374	2.29E-65	9.92E-64
ENSG00000147872	PLIN2	1727.694702	686.3141027	1.332343314	3.97E-65	1.72E-63
ENSG00000167508	MVD	535.3075732	1467.306083	-1.456303458	1.93E-64	8.31E-63
ENSG00000180139	ACTA2-AS1	38.25542059	448.8014014	-3.556820264	3.14E-64	1.35E-62
ENSG00000196923	PDLIM7	3480.749778	7168.417547	-1.042532744	3.39E-64	1.46E-62
ENSG00000057657	PRDM1	863.7514111	214.8877861	2.008404435	3.82E-64	1.64E-62
ENSG00000176046	NUPR1	2479.876246	4757.469373	-0.939854945	4.04E-64	1.73E-62
ENSG00000147027	TMEM47	1831.065999	3699.172743	-1.014235727	5.32E-64	2.27E-62
ENSG00000182985	CADM1	321.2406686	1101.055899	-1.777837255	5.38E-64	2.29E-62
ENSG00000011454	RABGAP1	995.3455052	2199.372535	-1.143377912	7.64E-64	3.25E-62
ENSG00000173801	JUP	57.52483665	436.7793869	-2.927123454	9.37E-64	3.98E-62
ENSG00000117724	CENPF	1320.407084	457.9887325	1.528209029	1.80E-63	7.62E-62
ENSG00000082512	TRAF5	506.5744157	1363.159128	-1.428439309	2.23E-63	9.42E-62
ENSG00000182606	TRAK1	1076.200116	2318.209542	-1.107773803	5.88E-63	2.48E-61
ENSG00000108932	SLC16A6	554.1778183	5.573208841	6.627915186	6.14E-63	2.59E-61
ENSG00000124006	OBSL1	1184.779121	2570.837998	-1.118105996	6.29E-63	2.64E-61
ENSG00000118680	MYL12B	2578.079256	4636.203914	-0.846812241	1.02E-62	4.29E-61
ENSG00000122966	CIT	716.4693362	170.7098493	2.069323267	1.32E-62	5.52E-61
ENSG00000104738	MCM4	1071.87818	307.7159856	1.80090601	2.10E-62	8.78E-61
ENSG00000128923	MINDY2	1210.749751	2613.249843	-1.110887143	2.58E-62	1.08E-60
ENSG00000172037	LAMB2	9284.411857	15933.8536	-0.779466662	2.98E-62	1.24E-60
ENSG00000188643	S100A16	1457.137243	2860.431926	-0.972733186	3.47E-62	1.44E-60
ENSG00000072310	SREBF1	632.026301	1645.821916	-1.382354628	4.05E-62	1.68E-60
ENSG00000197971	MBP	6.437378893	400.8284082	-6.002028555	4.59E-62	1.90E-60
ENSG00000229644	NAMPTP1	484.631492	52.02664008	3.22182156	5.18E-62	2.14E-60
ENSG00000090530	P3H2	370.131543	11.6471911	4.997016018	1.33E-61	5.49E-60
ENSG00000054793	ATP9A	912.182274	2026.593256	-1.151674782	1.75E-61	7.21E-60
ENSG00000060982	BCAT1	2691.231597	1334.747143	1.011992096	2.24E-61	9.19E-60
ENSG00000176490	DIRAS1	148.0880865	680.1366472	-2.200339603	3.14E-61	1.28E-59
ENSG00000280143	AP000892.3	35.18561921	383.3777948	-3.445174986	3.63E-61	1.48E-59
ENSG00000125148	MT2A	9389.50162	2410.769055	1.961519027	6.96E-61	2.83E-59
ENSG00000141522	ARHGDIA	1852.66548	3533.484531	-0.931322691	1.70E-60	6.90E-59
ENSG00000107104	KANK1	661.5165964	1587.592638	-1.262332327	1.71E-60	6.92E-59
ENSG00000162614	NEXN	774.032867	1776.046704	-1.198402511	1.76E-60	7.13E-59
ENSG00000196950	SLC39A10	207.8722231	840.0177578	-2.01565767	1.77E-60	7.15E-59
ENSG00000110911	SLC11A2	1575.890916	621.0575166	1.343727116	2.29E-60	9.22E-59
ENSG00000159403	C1R	7827.466732	4545.419778	0.783797809	2.39E-60	9.57E-59
ENSG00000006327	TNFRSF12A	2837.087454	4948.845444	-0.802940464	2.51E-60	1.01E-58
ENSG00000110906	KCTD10	1697.165346	3136.467327	-0.886304938	2.62E-60	1.05E-58
ENSG00000164530	PI16	8.783187274	345.9400482	-5.307204787	5.24E-60	2.09E-58
ENSG00000116679	IVNS1ABP	1792.119132	3703.865715	-1.047542162	6.83E-60	2.72E-58
ENSG00000119630	PGF	544.0329289	52.39663475	3.377634862	7.11E-60	2.82E-58
ENSG00000111145	ELK3	2650.894949	1274.316577	1.057382251	9.16E-60	3.63E-58
ENSG00000176170	SPHK1	2197.556481	4452.261824	-1.018440971	9.63E-60	3.81E-58
ENSG00000137831	UACA	1266.318817	2726.583297	-1.107628072	1.31E-59	5.17E-58
ENSG00000159335	PTMS	2262.082667	4002.510353	-0.823239535	1.74E-59	6.86E-58
ENSG00000132199	ENOSF1	1092.091518	376.8499726	1.535016182	5.44E-59	2.14E-57
ENSG00000049759	NEDD4L	593.5612659	117.9912359	2.33174124	8.51E-59	3.34E-57
ENSG00000176907	TCIM	336.6697077	16.25195557	4.380746991	1.14E-58	4.47E-57
ENSG00000140682	TGFB1I1	1454.977532	2958.301954	-1.023498096	1.29E-58	5.03E-57
ENSG00000145901	TNIP1	2457.832963	1198.827968	1.035837733	1.44E-58	5.63E-57
ENSG00000197442	MAP3K5	464.2230208	60.57282608	2.937630697	1.56E-58	6.10E-57
ENSG00000185483	ROR1	32.18707901	349.1494003	-3.432485459	5.62E-58	2.19E-56
ENSG00000122420	PTGFR	784.6173828	169.7106142	2.21048246	6.11E-58	2.37E-56
ENSG00000165124	SVEP1	1763.995197	3189.248529	-0.854239912	6.37E-58	2.47E-56
ENSG00000228495	LINC01013	45.85897478	422.9173806	-3.200283839	8.69E-58	3.36E-56
ENSG00000148700	ADD3	1875.808957	709.1429337	1.402815006	9.52E-58	3.68E-56
ENSG00000103966	EHD4	402.3863903	1107.092163	-1.460980677	1.11E-57	4.27E-56
ENSG00000131747	TOP2A	1364.748531	417.4255777	1.709306533	1.50E-57	5.77E-56
ENSG00000073910	FRY	105.5367432	540.5621072	-2.359777733	1.78E-57	6.81E-56
ENSG00000155011	DKK2	25.1011834	337.2724425	-3.754404212	1.78E-57	6.81E-56
ENSG00000187240	DYNC2H1	2616.845113	1151.995888	1.184074394	2.69E-57	1.03E-55

ENSG00000171617	ENC1	3887.558262	7397.749586	-0.928000986	2.72E-57	1.04E-55
ENSG00000128294	TPST2	478.2495934	1235.85457	-1.369466402	6.62E-57	2.51E-55
ENSG00000132000	PODNL1	588.3896554	1520.147648	-1.369782619	7.69E-57	2.92E-55
ENSG00000182809	CRIP2	218.2508519	828.0534332	-1.924421275	1.01E-56	3.81E-55
ENSG00000105825	TFPI2	1013.626885	326.3784121	1.634875906	1.12E-56	4.23E-55
ENSG00000112658	SRF	1057.450776	2232.707094	-1.078137349	1.26E-56	4.75E-55
ENSG00000114450	GNB4	1049.832574	2228.560633	-1.085673333	1.61E-56	6.06E-55
ENSG00000065413	ANKRD44	330.7158815	957.4014966	-1.534164587	2.30E-56	8.63E-55
ENSG00000103742	IGDCC4	797.952066	149.6897111	2.41291505	3.94E-56	1.48E-54
ENSG00000119714	GPR68	1217.847142	467.4822265	1.38067996	4.33E-56	1.62E-54
ENSG00000080573	COL5A3	131.9490458	623.6553059	-2.24310345	1.09E-55	4.09E-54
ENSG00000056972	TRAF3IP2	957.7996469	291.7660403	1.715089253	1.11E-55	4.13E-54
ENSG00000108639	SYNGR2	133.9928629	584.4899608	-2.12312497	1.22E-55	4.55E-54
ENSG00000164032	H2AFZ	1816.219285	849.1201613	1.096878542	1.60E-55	5.94E-54
ENSG00000146950	SHROOM2	15.3773588	307.7554834	-4.330221341	2.67E-55	9.92E-54
ENSG00000005059	MCUB	106.9409372	526.1368447	-2.298792027	6.71E-55	2.49E-53
ENSG00000072778	ACADVL	2945.173282	1539.789876	0.935213386	9.16E-55	3.39E-53
ENSG00000173530	TNFRSF10D	1776.511761	777.7641361	1.19127826	1.01E-54	3.73E-53
ENSG00000129353	SLC44A2	2011.428704	3776.850527	-0.90979811	1.81E-54	6.66E-53
ENSG00000198814	GK	522.9246015	85.40862453	2.616139445	3.85E-54	1.41E-52
ENSG00000075618	FSCN1	3022.491838	5249.809153	-0.796884328	3.99E-54	1.46E-52
ENSG00000204291	COL15A1	32.53269768	345.0229161	-3.401517846	4.92E-54	1.79E-52
ENSG00000185813	PCYT2	255.2542828	815.7058406	-1.677339809	8.86E-54	3.22E-52
ENSG00000158966	CACHD1	786.4584327	231.2064758	1.766262986	1.04E-53	3.77E-52
ENSG00000112078	KCTD20	1444.863139	2718.128467	-0.911959723	1.42E-53	5.14E-52
ENSG00000153560	UBP1	2565.484209	1262.726971	1.022422716	1.53E-53	5.52E-52
ENSG00000183876	ARSI	1028.882656	358.4876542	1.521561702	1.55E-53	5.59E-52
ENSG00000124766	SOX4	2415.157508	1092.439432	1.145203317	2.08E-53	7.48E-52
ENSG00000166833	NAV2	990.3595391	350.797897	1.496785604	2.42E-53	8.68E-52
ENSG00000027697	IFNGR1	1453.880012	574.9234857	1.337974993	4.81E-53	1.72E-51
ENSG00000119655	NPC2	1567.924447	2928.126416	-0.901332068	5.60E-53	2.01E-51
ENSG00000106976	DNM1	1722.131177	768.0905147	1.164744098	5.88E-53	2.10E-51
ENSG00000142197	DOP1B	185.5184524	700.0826305	-1.914150299	8.00E-53	2.85E-51
ENSG00000131669	NINJ1	787.3351636	228.6901134	1.7845832	9.85E-53	3.51E-51
ENSG00000185022	MAFF	1158.349815	406.4180925	1.510212828	1.02E-52	3.62E-51
ENSG00000118113	MMP8	299.3803212	11.8529642	4.652241179	1.19E-52	4.24E-51
ENSG00000105355	PLIN3	1765.61602	3215.669964	-0.865530705	1.75E-52	6.20E-51
ENSG00000138944	SHISAL1	1718.584056	768.7496436	1.161260612	1.99E-52	7.04E-51
ENSG00000099284	H2AFY2	573.4159734	135.4644248	2.082200061	2.81E-52	9.90E-51
ENSG00000134198	TSPAN2	154.8752991	726.6972333	-2.23538676	2.81E-52	9.91E-51
ENSG00000184254	ALDH1A3	349.6035006	32.76621073	3.419526612	3.77E-52	1.32E-50
ENSG00000133657	ATP13A3	5977.00445	3120.107679	0.938254494	5.43E-52	1.90E-50
ENSG00000113758	DBN1	2130.665484	3811.832195	-0.839046152	7.63E-52	2.67E-50
ENSG00000132613	MTSS1L	895.7203192	2180.81639	-1.28488539	1.22E-51	4.27E-50
ENSG00000160255	ITGB2	11.86043776	270.7985791	-4.514047464	1.30E-51	4.54E-50
ENSG00000181019	NQO1	2693.605665	1116.975889	1.270936482	1.39E-51	4.85E-50
ENSG00000117394	SLC2A1	567.738142	1431.038271	-1.335445953	1.46E-51	5.08E-50
ENSG00000151276	MAGI1	365.1882852	1061.397577	-1.537222025	1.93E-51	6.68E-50
ENSG00000100596	SPTLC2	1131.486489	2226.178071	-0.976855323	4.03E-51	1.40E-49
ENSG00000091409	ITGA6	20.1910344	282.6719585	-3.804014443	1.11E-50	3.83E-49
ENSG00000179134	SAMD4B	1594.514794	2814.795426	-0.820052739	1.13E-50	3.88E-49
ENSG00000162817	C1orf115	650.4610989	173.7492652	1.90358466	1.73E-50	5.96E-49
ENSG00000107281	NPDC1	207.3524218	813.296362	-1.969777382	2.62E-50	8.99E-49
ENSG00000133106	EPSTI1	489.7268138	96.22289323	2.346580095	4.56E-50	1.56E-48
ENSG00000175567	UCP2	32.04222946	338.11777	-3.389898839	7.17E-50	2.45E-48
ENSG00000099889	ARVCF	39.99929481	329.0878393	-3.036006536	8.14E-50	2.77E-48
ENSG00000259207	ITGB3	450.3291984	56.16909149	3.008801002	1.23E-49	4.19E-48
ENSG00000196878	LAMB3	449.9417459	75.05270501	2.580496507	1.28E-49	4.34E-48
ENSG00000177239	MAN1B1	1244.784273	2402.431235	-0.9485322	1.83E-49	6.19E-48
ENSG00000138759	FRAS1	284.079793	964.0576046	-1.765817827	2.75E-49	9.31E-48
ENSG00000158555	GDPD5	81.8224341	485.1302092	-2.568836353	2.98E-49	1.01E-47
ENSG00000198719	DLL1	294.907048	8.069451293	5.192560836	4.31E-49	1.45E-47
ENSG00000115419	GLS	7056.99471	11933.75298	-0.758194478	7.02E-49	2.36E-47
ENSG00000102317	RBM3	2632.44882	5057.931018	-0.941516951	2.17E-48	7.30E-47
ENSG00000176871	WSB2	1299.892319	2599.917328	-1.000324695	2.94E-48	9.85E-47
ENSG00000154864	PIEZO2	124.8930409	557.084963	-2.156740083	4.01E-48	1.34E-46

ENSG00000164741	DLC1	1892.831787	3371.732802	-0.832629988	5.57E-48	1.86E-46
ENSG00000198722	UNC13B	480.2990701	1157.382518	-1.269049918	7.18E-48	2.39E-46
ENSG00000138835	RGS3	3751.078571	2068.635749	0.858753749	9.38E-48	3.12E-46
ENSG00000141429	GALNT1	1019.458959	2112.183586	-1.050806211	1.34E-47	4.46E-46
ENSG00000155760	FZD7	957.2145691	1928.387831	-1.011097614	1.95E-47	6.45E-46
ENSG00000144583	4-Mar	303.7237374	894.8211981	-1.556829028	2.92E-47	9.67E-46
ENSG00000081041	CXCL2	1194.918184	1.73316944	9.413222426	3.50E-47	1.16E-45
ENSG00000131323	TRAF3	1231.680485	529.0106451	1.219539905	4.69E-47	1.54E-45
ENSG00000211448	DIO2	249.6512915	817.0018182	-1.710940531	6.22E-47	2.04E-45
ENSG00000123143	PKN1	1498.774557	2771.290207	-0.887094605	1.03E-46	3.38E-45
ENSG00000102804	TSC22D1	3464.157159	1935.67147	0.840089549	1.89E-46	6.17E-45
ENSG00000251493	FOXD1	535.1865114	1240.124095	-1.211473351	1.99E-46	6.51E-45
ENSG00000185215	TNFAIP2	1498.482083	677.1616084	1.146446231	2.23E-46	7.26E-45
ENSG00000135678	CPM	375.1723856	59.00923611	2.670150139	2.39E-46	7.78E-45
ENSG00000159363	ATP13A2	319.0123307	875.8350717	-1.457854309	2.60E-46	8.43E-45
ENSG00000164619	BMPER	515.5057808	1186.073589	-1.201713221	2.62E-46	8.48E-45
ENSG00000019144	PHLDB1	2000.663942	3392.997724	-0.762027491	3.95E-46	1.28E-44
ENSG00000109099	PMP22	1931.46916	3666.430954	-0.9249939	4.42E-46	1.43E-44
ENSG00000172081	MOB3A	562.3822194	1260.619716	-1.164588846	4.57E-46	1.47E-44
ENSG00000196562	SULF2	588.8440396	1367.034791	-1.216606656	4.87E-46	1.57E-44
ENSG00000162337	LRP5	1139.098498	2274.890115	-0.998206385	7.96E-46	2.55E-44
ENSG00000168056	LTBP3	1269.00037	2516.966633	-0.988868206	8.79E-46	2.81E-44
ENSG00000144645	OSBPL10	50.97536388	346.8975801	-2.769218759	1.75E-45	5.58E-44
ENSG00000150764	DIXDC1	969.0672989	1861.675842	-0.941663609	2.09E-45	6.67E-44
ENSG00000169710	FASN	3708.63223	11424.68284	-1.623379463	2.23E-45	7.10E-44
ENSG00000162894	FCMR	326.4412389	39.00841568	3.066308956	2.86E-45	9.08E-44
ENSG00000055070	SZRD1	1639.807521	2854.824553	-0.800183509	3.00E-45	9.52E-44
ENSG00000163347	CLDN1	275.5224927	13.28257779	4.370118364	3.21E-45	1.01E-43
ENSG00000204767	INSYN2B	509.2880957	114.5041624	2.152603983	7.74E-45	2.45E-43
ENSG00000171608	PIK3CD	1610.336474	790.1317049	1.027223156	1.25E-44	3.93E-43
ENSG00000128585	MKLN1	1811.962361	863.346046	1.068821194	1.46E-44	4.60E-43
ENSG00000100300	TSPO	1429.361866	2742.87366	-0.940733727	1.70E-44	5.35E-43
ENSG00000114423	CBLB	1192.780417	2226.67135	-0.901260998	1.97E-44	6.15E-43
ENSG00000165895	ARHGAP42	515.9723629	115.4005148	2.160694953	2.48E-44	7.74E-43
ENSG00000117525	F3	489.8627036	1131.821008	-1.207905361	2.73E-44	8.51E-43
ENSG00000111371	SLC38A1	519.8705432	1208.650594	-1.216510626	3.00E-44	9.32E-43
ENSG00000104332	SFRP1	15.07266716	241.8002093	-4.011064688	3.03E-44	9.40E-43
ENSG00000169583	CLIC3	53.32296584	369.84499	-2.802860156	3.17E-44	9.82E-43
ENSG00000173511	VEGFB	1209.857949	2277.02951	-0.912127625	3.51E-44	1.09E-42
ENSG00000102287	GABRE	768.515383	247.1584298	1.636357721	4.13E-44	1.27E-42
ENSG00000069702	TGFBR3	1005.111423	370.8067889	1.439257976	6.41E-44	1.97E-42
ENSG00000166689	PLEKHA7	28.31781246	279.0759396	-3.299879342	7.52E-44	2.31E-42
ENSG00000169330	KIAA1024	652.7105942	204.3463199	1.675098905	7.90E-44	2.42E-42
ENSG00000112186	CAP2	294.2983209	834.3202511	-1.50341425	1.34E-43	4.10E-42
ENSG00000196141	SPATS2L	1187.116938	2153.147542	-0.858563055	1.90E-43	5.80E-42
ENSG00000160213	CSTB	994.0564085	2111.770657	-1.085797844	2.02E-43	6.17E-42
ENSG00000102760	RGCC	30.84329851	297.0236207	-3.267195763	2.12E-43	6.44E-42
ENSG00000172331	BPGM	1236.994475	509.2263586	1.280366675	2.17E-43	6.61E-42
ENSG00000160209	PDXK	1391.736613	2454.253677	-0.818894541	2.49E-43	7.57E-42
ENSG00000058668	ATP2B4	1746.383632	3005.559597	-0.783443755	2.79E-43	8.44E-42
ENSG00000183762	KREMEN1	1270.340625	455.909402	1.479104661	3.47E-43	1.05E-41
ENSG00000156515	HK1	1586.690844	2835.603518	-0.837169558	5.46E-43	1.65E-41
ENSG00000102466	FGF14	81.80052606	411.271948	-2.330796719	6.74E-43	2.03E-41
ENSG00000151320	AKAP6	8.191846001	259.2288138	-4.95478219	7.90E-43	2.37E-41
ENSG00000148484	RSU1	1845.694034	3234.242394	-0.809327899	1.09E-42	3.28E-41
ENSG00000206538	VGLL3	3383.643505	5788.181711	-0.774647242	1.15E-42	3.45E-41
ENSG00000111912	NCOA7	791.3093538	221.6036797	1.837865297	1.18E-42	3.52E-41
ENSG00000008294	SPAG9	1904.4201	3238.967528	-0.765893811	1.22E-42	3.63E-41
ENSG00000028137	TNFRSF1B	319.3749902	40.89949375	2.961953371	1.27E-42	3.78E-41
ENSG00000162804	SNED1	171.9691531	625.1829152	-1.864927738	1.61E-42	4.78E-41
ENSG00000119927	GPAM	832.8304075	289.4785184	1.524641303	1.76E-42	5.21E-41
ENSG00000189120	SP6	255.6639683	9.353417644	4.758050766	2.80E-42	8.30E-41
ENSG00000118508	RAB32	1819.011655	957.9941043	0.925277649	2.99E-42	8.86E-41
ENSG00000134569	LRP4	725.5799397	250.2684372	1.53549487	3.14E-42	9.29E-41
ENSG00000100364	KIAA0930	2951.69344	1713.012119	0.785044228	3.80E-42	1.12E-40
ENSG00000130304	SLC27A1	481.651022	1163.383408	-1.271961124	4.10E-42	1.21E-40

ENSG00000176225	RTTN	599.8998862	165.2543543	1.861351433	5.00E-42	1.47E-40
ENSG00000174945	AMZ1	111.8762985	470.7111531	-2.076328002	5.33E-42	1.57E-40
ENSG00000165633	VSTM4	285.8789547	771.3386061	-1.431452101	5.90E-42	1.73E-40
ENSG00000168209	DDIT4	347.6971982	50.2615734	2.787452966	8.48E-42	2.49E-40
ENSG00000143153	ATP1B1	513.7636059	1238.339753	-1.269934127	1.72E-41	5.04E-40
ENSG00000083223	TUT7	1568.586672	798.8753214	0.973645797	3.63E-41	1.06E-39
ENSG00000116574	RHOU	365.6315429	72.62135579	2.332349815	4.04E-41	1.18E-39
ENSG00000183779	ZNF703	138.4275362	517.6119838	-1.900301924	5.60E-41	1.63E-39
ENSG00000123989	CHPF	2981.159449	10056.24252	-1.754302668	6.14E-41	1.79E-39
ENSG00000052802	MSMO1	2126.944303	4221.427398	-0.989379968	7.51E-41	2.18E-39
ENSG00000175592	FOSL1	914.3580165	342.8644759	1.415257799	1.24E-40	3.58E-39
ENSG00000072422	RHOBTB1	682.023453	1466.015516	-1.1039405	1.86E-40	5.37E-39
ENSG00000150457	LATS2	1309.699791	2361.589388	-0.850609027	2.15E-40	6.21E-39
ENSG00000125740	FOSB	381.3604926	41.43946963	3.20400408	2.30E-40	6.62E-39
ENSG00000168575	SLC20A2	505.507344	1234.600828	-1.287395973	2.45E-40	7.03E-39
ENSG00000100292	HMOX1	4035.779636	2068.046305	0.965161571	2.89E-40	8.28E-39
ENSG00000254851	AP005018.2	40.17690002	306.4565421	-2.936912145	3.62E-40	1.04E-38
ENSG00000099864	PALM	86.80374996	420.654648	-2.278228859	3.87E-40	1.11E-38
ENSG00000042493	CAPG	914.6055409	1740.318211	-0.928797738	6.40E-40	1.82E-38
ENSG00000135269	TES	846.1478894	1652.417177	-0.966283382	7.07E-40	2.01E-38
ENSG00000144560	VGLL4	679.8597156	1391.468623	-1.033335987	8.47E-40	2.40E-38
ENSG00000143382	ADAMTSL4	90.81274414	411.3508984	-2.182534764	8.62E-40	2.44E-38
ENSG00000197747	S100A10	744.2907743	1501.622606	-1.013580483	8.79E-40	2.49E-38
ENSG00000145040	UCN2	341.033144	54.29347246	2.650864039	9.55E-40	2.70E-38
ENSG00000161202	DVL3	1702.724064	2899.701724	-0.768099375	9.94E-40	2.80E-38
ENSG00000105971	CAV2	1294.153348	2370.843051	-0.873522766	1.10E-39	3.09E-38
ENSG00000099204	ABLIM1	25.00955304	256.6331607	-3.372427238	1.31E-39	3.68E-38
ENSG00000107819	SFXN3	1045.781524	2032.464536	-0.959531762	1.44E-39	4.03E-38
ENSG00000175115	PACS1	1428.127286	2433.978825	-0.769313286	1.44E-39	4.04E-38
ENSG00000183386	FHL3	282.2543395	748.9382272	-1.406836084	2.20E-39	6.15E-38
ENSG00000112414	ADGRG6	61.54093088	343.6322098	-2.475632167	2.23E-39	6.23E-38
ENSG00000183250	LINC01547	45.32390214	312.0413845	-2.779850144	2.37E-39	6.61E-38
ENSG00000103222	ABCC1	2377.703686	1371.478306	0.793767038	3.52E-39	9.81E-38
ENSG00000120306	CYSTM1	146.5406719	519.8008581	-1.824569039	3.57E-39	9.94E-38
ENSG00000249669	CARMN	487.6265717	1187.697898	-1.284795575	4.39E-39	1.22E-37
ENSG00000143344	RGL1	941.9947493	379.9465749	1.31073085	4.47E-39	1.24E-37
ENSG00000167972	ABCA3	11.36692009	208.8278238	-4.183725656	5.66E-39	1.57E-37
ENSG00000109743	BST1	56.65473824	335.4767289	-2.569316992	6.09E-39	1.69E-37
ENSG00000033170	FUT8	1538.756901	741.6474759	1.053302785	8.04E-39	2.22E-37
ENSG00000197361	FBXL22	15.0218695	209.3939745	-3.805326379	8.89E-39	2.45E-37
ENSG00000241553	ARPC4	586.747561	1236.277644	-1.075353881	9.09E-39	2.50E-37
ENSG00000159399	HK2	799.3502472	264.8672803	1.594480675	1.07E-38	2.94E-37
ENSG00000187688	TRPV2	457.7985	1057.796025	-1.208629591	1.10E-38	3.02E-37
ENSG00000109320	NFKB1	1826.978657	952.8248784	0.939130499	1.11E-38	3.04E-37
ENSG00000171604	CXXC5	607.2901244	1320.417495	-1.120627377	1.14E-38	3.11E-37
ENSG00000178996	SNX18	1094.565697	2055.100113	-0.90868169	1.14E-38	3.11E-37
ENSG00000233117	LINC00702	4.024708987	229.2265722	-5.822939647	1.91E-38	5.18E-37
ENSG00000081189	MEF2C	45.46193051	291.8846851	-2.682247289	1.97E-38	5.36E-37
ENSG00000125845	BMP2	231.9081001	20.64753635	3.488191839	2.51E-38	6.80E-37
ENSG00000135596	MICAL1	1060.624849	1952.035901	-0.880374928	3.58E-38	9.71E-37
ENSG00000135821	GLUL	3417.30068	1998.086583	0.774092547	4.00E-38	1.08E-36
ENSG00000173621	LRFN4	195.6133908	665.7217733	-1.769122568	4.49E-38	1.21E-36
ENSG00000184557	SOCS3	877.1491421	363.9746447	1.269528052	5.87E-38	1.58E-36
ENSG00000187699	C2orf88	264.0282274	25.66850792	3.364808694	6.61E-38	1.77E-36
ENSG00000244586	WNT5A-AS1	644.0340057	187.8619695	1.778886667	8.25E-38	2.21E-36
ENSG00000163453	IGFBP7	2224.615154	4102.012332	-0.883421017	9.36E-38	2.50E-36
ENSG00000171992	SYNPO	122.8680823	486.9558034	-1.989613252	9.52E-38	2.54E-36
ENSG00000187479	C11orf96	129.5534167	590.3890188	-2.191485594	1.53E-37	4.09E-36
ENSG00000179295	PTPN11	4370.055261	2595.984821	0.751339956	1.77E-37	4.70E-36
ENSG00000109686	SH3D19	2641.928898	1524.283066	0.794114734	2.18E-37	5.79E-36
ENSG00000197381	ADARB1	448.0867548	1112.39516	-1.312719702	2.20E-37	5.83E-36
ENSG00000171067	C11orf24	713.2022868	1487.972608	-1.061760282	2.37E-37	6.27E-36
ENSG00000133169	BEX1	331.9294559	2.763498208	6.8883408	2.47E-37	6.53E-36
ENSG00000117318	ID3	1084.175949	2073.577373	-0.93643357	2.65E-37	7.01E-36
ENSG00000198743	SLC5A3	8632.163341	5095.510718	0.760409094	2.68E-37	7.06E-36
ENSG00000052126	PLEKHA5	859.1524099	350.0539065	1.295042993	3.01E-37	7.95E-36

ENSG00000189223	PAX8-AS1	1783.194983	815.0933951	1.129605616	3.24E-37	8.54E-36
ENSG00000026103	FAS	1496.431638	744.4981312	1.007174117	4.12E-37	1.08E-35
ENSG00000173156	RHOD	97.40924286	441.8129765	-2.184207656	4.14E-37	1.09E-35
ENSG00000152990	ADGRA3	916.0602957	345.2037647	1.407570224	4.51E-37	1.18E-35
ENSG00000033627	ATP6V0A1	662.4761584	1368.047889	-1.045177426	5.47E-37	1.43E-35
ENSG00000135272	MDFIC	2789.467437	1554.525827	0.843686184	7.16E-37	1.87E-35
ENSG00000105810	CDK6	2294.652418	1246.222759	0.881273529	7.59E-37	1.98E-35
ENSG00000169902	TPST1	1366.785118	685.8549259	0.994487492	9.56E-37	2.50E-35
ENSG00000003402	CFLAR	2256.167742	1230.599618	0.873967653	1.27E-36	3.32E-35
ENSG00000093010	COMT	1158.377756	2006.734508	-0.793008196	1.28E-36	3.32E-35
ENSG00000185189	NRBP2	496.0768959	1160.806436	-1.225751002	1.71E-36	4.43E-35
ENSG00000148773	MKI67	940.5307856	345.0130038	1.447301967	1.93E-36	4.99E-35
ENSG00000265972	TXNIP	693.3020428	252.470294	1.458582206	2.27E-36	5.86E-35
ENSG00000173457	PPP1R14B	1417.779856	2718.02389	-0.938451586	3.04E-36	7.85E-35
ENSG00000068305	MEF2A	792.3734945	1514.817465	-0.934547855	3.62E-36	9.35E-35
ENSG00000182022	CHST15	57.02754727	303.3101358	-2.410832028	3.81E-36	9.82E-35
ENSG00000102452	NALCN	30.81529156	235.3096182	-2.931590421	4.25E-36	1.09E-34
ENSG00000137393	RNF144B	365.4481878	66.66813349	2.451556219	4.65E-36	1.19E-34
ENSG00000111696	NT5DC3	197.9393636	598.8216546	-1.597488492	5.05E-36	1.30E-34
ENSG00000279118	AC093535.2	102.2375619	444.166011	-2.116050188	5.91E-36	1.51E-34
ENSG00000164093	PITX2	180.1245661	560.1077069	-1.638710631	6.04E-36	1.55E-34
ENSG00000014216	CAPN1	1638.317315	2767.466156	-0.75685325	8.25E-36	2.11E-34
ENSG00000123159	GIPC1	1063.453446	2020.701499	-0.925782019	8.42E-36	2.15E-34
ENSG00000122547	EEPD1	19.57652922	231.1622107	-3.573172099	9.08E-36	2.31E-34
ENSG00000109756	RAPGEF2	1844.833334	969.9497251	0.928337177	1.07E-35	2.71E-34
ENSG00000079215	SLC1A3	1318.310217	624.3128761	1.079053705	1.49E-35	3.78E-34
ENSG00000179954	SSC5D	1137.879797	2156.922673	-0.923080933	1.68E-35	4.25E-34
ENSG00000152818	UTRN	2246.69699	3958.100747	-0.817003907	2.16E-35	5.44E-34
ENSG00000101955	SRPX	1892.401105	1075.605516	0.815094977	2.68E-35	6.74E-34
ENSG00000133142	TCEAL4	962.4599526	1847.540467	-0.940118321	2.95E-35	7.39E-34
ENSG00000159200	RCAN1	1227.695447	569.3742091	1.108113937	3.68E-35	9.21E-34
ENSG00000116133	DHCR24	2451.895923	6757.191282	-1.462854313	3.86E-35	9.65E-34
ENSG00000124212	PTGIS	56.48816931	349.1182452	-2.635448565	4.07E-35	1.02E-33
ENSG00000154678	PDE1C	143.7806882	552.9396255	-1.945529263	4.09E-35	1.02E-33
ENSG00000115828	QPCT	711.1563229	267.864698	1.409800612	4.33E-35	1.08E-33
ENSG00000243156	MICAL3	464.8865383	1051.465785	-1.17808919	4.98E-35	1.24E-33
ENSG00000054356	PTPRN	269.0560315	37.8444058	2.829920459	5.00E-35	1.24E-33
ENSG00000187634	SAMD11	18.50773414	208.4402921	-3.499771931	5.22E-35	1.30E-33
ENSG00000151929	BAG3	753.0005536	1481.954669	-0.976278449	6.03E-35	1.49E-33
ENSG00000140945	CDH13	2021.35599	1092.372379	0.888095534	7.12E-35	1.76E-33
ENSG00000123146	ADGRE5	346.3020865	834.1887675	-1.268201385	7.17E-35	1.77E-33
ENSG00000104951	IL4I1	229.7809183	27.7328713	3.051294246	7.53E-35	1.86E-33
ENSG00000172346	CSDC2	42.31414111	266.0537141	-2.651415272	1.08E-34	2.65E-33
ENSG00000143545	RAB13	682.3133084	238.2145496	1.518098786	1.17E-34	2.88E-33
ENSG00000168386	FILIP1L	519.8763873	1154.880364	-1.150785971	1.44E-34	3.53E-33
ENSG00000175416	CLTB	903.6288398	1680.309461	-0.894603506	1.57E-34	3.84E-33
ENSG00000070961	ATP2B1	3411.622421	1733.012414	0.977534388	1.86E-34	4.54E-33
ENSG00000114268	PFKFB4	438.5591351	121.9788698	1.846272276	1.91E-34	4.67E-33
ENSG00000181072	CHRM2	254.72496	36.40738052	2.805251291	1.96E-34	4.77E-33
ENSG00000170681	CAVIN4	60.31166578	311.585065	-2.367079211	2.41E-34	5.87E-33
ENSG00000177426	TGIF1	1115.810812	500.9446939	1.156505687	2.59E-34	6.30E-33
ENSG00000128606	LRRC17	273.8505979	42.21069644	2.69337	4.37E-34	1.06E-32
ENSG00000122359	ANXA11	1496.268865	2669.165763	-0.834916686	4.83E-34	1.17E-32
ENSG00000135837	CEP350	1888.273516	1001.306274	0.915551303	6.00E-34	1.45E-32
ENSG00000166396	SERPINB7	422.2208359	1005.212306	-1.25058658	9.83E-34	2.37E-32
ENSG00000125648	SLC25A23	561.4568293	1133.595056	-1.01351877	1.04E-33	2.50E-32
ENSG00000078804	TP53INP2	403.3129178	943.1057703	-1.226415176	1.08E-33	2.60E-32
ENSG00000203706	SERTAD4-AS1	8.289669605	173.0687047	-4.367279782	1.13E-33	2.71E-32
ENSG00000100605	ITPK1	402.9827592	915.6586599	-1.184713871	1.57E-33	3.76E-32
ENSG00000157766	ACAN	2.752095335	223.5134758	-6.318192349	1.63E-33	3.89E-32
ENSG00000107331	ABCA2	715.3362237	1469.93693	-1.03939599	1.76E-33	4.20E-32
ENSG00000198873	GRK5	672.5236977	229.9123391	1.547323682	1.82E-33	4.35E-32
ENSG00000256235	SMIM3	895.3030862	405.81524	1.14116926	2.69E-33	6.42E-32
ENSG00000113389	NPR3	3.049965797	208.1009444	-6.063820331	2.70E-33	6.43E-32
ENSG00000135932	CAB39	1632.311427	907.0617312	0.84802357	2.93E-33	6.97E-32
ENSG00000135373	EHF	197.8850731	4.855097942	5.335885719	3.27E-33	7.78E-32

ENSG00000189410	SH2D5	15.86163378	188.6298922	-3.569334543	4.09E-33	9.71E-32
ENSG00000120738	EGR1	2321.664617	388.1911314	2.580731139	4.23E-33	1.00E-31
ENSG00000145147	SLIT2	3563.420757	2101.531674	0.761602334	4.67E-33	1.11E-31
ENSG00000151376	ME3	58.66489291	327.6005313	-2.486089191	4.94E-33	1.17E-31
ENSG00000169926	KLF13	1608.575625	2711.131827	-0.75305961	5.10E-33	1.20E-31
ENSG00000099337	KCNK6	321.3775313	801.4394659	-1.317479883	6.51E-33	1.54E-31
ENSG00000146242	TPBG	2425.384314	1315.968149	0.882378808	6.91E-33	1.63E-31
ENSG00000173599	PC	155.958549	480.7882525	-1.624365363	9.25E-33	2.18E-31
ENSG00000143507	DUSP10	468.3887456	138.4144248	1.759607679	9.93E-33	2.33E-31
ENSG00000004799	PDK4	198.3692538	4.856749996	5.339802467	1.00E-32	2.36E-31
ENSG00000144959	NCEH1	627.6505926	227.1090929	1.466969245	1.25E-32	2.92E-31
ENSG00000158270	COLEC12	467.4173969	1028.693604	-1.137680221	1.26E-32	2.96E-31
ENSG00000114853	ZBTB47	913.4624352	1670.256293	-0.871121293	1.33E-32	3.12E-31
ENSG00000125534	PPDPF	883.6661422	1627.326954	-0.881311342	1.44E-32	3.37E-31
ENSG00000132589	FLOT2	335.3367748	804.6796296	-1.264535235	2.27E-32	5.29E-31
ENSG00000244879	GABPB1-AS1	919.0928055	335.9941084	1.452735244	3.15E-32	7.34E-31
ENSG00000198715	GLMP	744.3376157	1488.786813	-1.001657934	3.19E-32	7.41E-31
ENSG00000140285	FGF7	1947.345073	3428.099036	-0.816098442	3.21E-32	7.46E-31
ENSG00000101400	SNTA1	206.5320256	596.0453417	-1.528306245	3.36E-32	7.80E-31
ENSG00000170571	EMB	109.6933816	405.5365714	-1.889411314	3.57E-32	8.28E-31
ENSG00000189129	PLAC9	38.02566752	262.5932414	-2.787658346	3.96E-32	9.15E-31
ENSG00000149289	ZC3H12C	511.8735278	161.4290769	1.665742396	5.14E-32	1.19E-30
ENSG00000137193	PIM1	684.128169	248.1168553	1.461961411	5.81E-32	1.34E-30
ENSG00000155511	GRIA1	188.565463	5.333189432	5.161130918	6.39E-32	1.47E-30
ENSG00000163734	CXCL3	2919.943714	0	13.88132684	6.46E-32	1.49E-30
ENSG00000148154	UGCG	1309.233743	691.373662	0.921091432	8.10E-32	1.86E-30
ENSG00000067064	IDI1	1160.89109	2180.080564	-0.909777988	1.03E-31	2.36E-30
ENSG00000166503	HDGFL3	956.2977236	438.1722986	1.12575904	1.08E-31	2.46E-30
ENSG00000092621	PHGDH	553.4862119	185.9420854	1.573681718	1.38E-31	3.15E-30
ENSG00000122729	ACO1	2742.454858	1627.62288	0.752933495	1.47E-31	3.35E-30
ENSG00000013288	MAN2B2	1084.043129	2051.73999	-0.920595902	1.56E-31	3.53E-30
ENSG00000121691	CAT	1252.987878	651.7202889	0.942735818	1.73E-31	3.93E-30
ENSG00000163637	PRICKLE2	889.0258242	384.970699	1.208583309	1.85E-31	4.19E-30
ENSG00000158292	GPR153	182.0230662	555.1826772	-1.608771932	3.26E-31	7.38E-30
ENSG00000139263	LRIG3	205.9484825	566.3113691	-1.459062131	3.53E-31	7.97E-30
ENSG00000250657	AC097451.1	370.9472566	1.808672008	7.723642072	3.55E-31	8.03E-30
ENSG00000025423	HSD17B6	16.29448316	176.6786371	-3.443609968	3.86E-31	8.71E-30
ENSG00000042445	RETSAT	882.4467477	1596.706188	-0.855796025	4.45E-31	1.00E-29
ENSG00000146278	PNRC1	1119.633633	537.6589102	1.057754621	4.87E-31	1.10E-29
ENSG00000075213	SEMA3A	52.10913257	285.2459089	-2.449197614	5.32E-31	1.19E-29
ENSG00000185504	FAAP100	690.8420681	1301.378178	-0.913312554	6.48E-31	1.45E-29
ENSG00000117586	TNFSF4	44.63813578	255.0381203	-2.515628681	7.07E-31	1.58E-29
ENSG00000260910	LINC00565	111.9813165	412.5236768	-1.881437713	7.46E-31	1.66E-29
ENSG00000196517	SLC6A9	443.1358915	131.6209749	1.75030977	7.57E-31	1.69E-29
ENSG00000127329	PTPRB	2.521714333	206.9287807	-6.373396576	9.17E-31	2.04E-29
ENSG00000005243	COPZ2	903.60114	1679.592658	-0.894249845	9.82E-31	2.18E-29
ENSG00000108179	PPIF	970.5393274	420.5140712	1.207547136	1.20E-30	2.65E-29
ENSG00000184227	ACOT1	308.4776315	739.3654556	-1.262041161	1.38E-30	3.06E-29
ENSG00000196712	NF1	2455.041639	1426.523061	0.783483784	1.45E-30	3.21E-29
ENSG00000161091	MFSD12	441.5702504	987.1763237	-1.161248049	1.46E-30	3.22E-29
ENSG00000095752	IL11	948.8229187	368.0239501	1.36646155	1.67E-30	3.67E-29
ENSG00000128283	CDC42EP1	1033.996833	1868.747235	-0.853381652	2.03E-30	4.47E-29
ENSG00000104447	TRPS1	1693.095376	840.5305666	1.010323287	2.32E-30	5.10E-29
ENSG00000177508	IRX3	168.6276947	510.117747	-1.597310392	2.76E-30	6.07E-29
ENSG00000115649	CNPPD1	444.6206555	954.6159684	-1.102368682	2.82E-30	6.19E-29
ENSG00000163820	FYCO1	427.8536518	926.5637706	-1.113725273	2.90E-30	6.36E-29
ENSG00000232679	LINC01705	300.0701381	814.9713398	-1.438949504	2.93E-30	6.43E-29
ENSG00000129925	TMEM8A	1060.375358	1837.403058	-0.793939265	3.22E-30	7.05E-29
ENSG00000125347	IRF1	343.9674748	80.67678717	2.091792831	4.13E-30	9.01E-29
ENSG00000173281	PPP1R3B	600.621572	1225.535041	-1.027805858	4.15E-30	9.06E-29
ENSG00000151012	SLC7A11	653.9683015	218.3294506	1.584661727	4.45E-30	9.70E-29
ENSG00000025772	TOMM34	260.2054491	663.1092429	-1.348286918	4.90E-30	1.07E-28
ENSG00000082482	KCNK2	984.9004163	1778.384516	-0.851918937	4.90E-30	1.07E-28
ENSG00000166165	CKB	17.40178375	183.5269614	-3.395292854	5.49E-30	1.19E-28
ENSG00000141052	MYOCD	175.2347863	4.981769096	5.154392859	5.68E-30	1.23E-28
ENSG00000076344	RGS11	14.73386967	165.9079737	-3.498508833	5.85E-30	1.27E-28

ENSG00000101236	RNF24	1282.907367	625.5262719	1.035685428	6.48E-30	1.40E-28
ENSG00000186594	MIR22HG	689.6145505	1537.260973	-1.15560266	6.65E-30	1.44E-28
ENSG00000103066	PLA2G15	290.0431366	754.866448	-1.379996426	6.79E-30	1.47E-28
ENSG00000131378	RFTN1	795.645391	1451.721836	-0.867585535	7.15E-30	1.54E-28
ENSG00000167657	DAPK3	1451.310553	2442.330691	-0.751409231	7.29E-30	1.57E-28
ENSG00000131504	DIAPH1	1105.315477	1957.64559	-0.824516926	7.54E-30	1.62E-28
ENSG00000141736	ERBB2	938.2909845	1689.84283	-0.849177527	8.34E-30	1.79E-28
ENSG00000152127	MGAT5	759.0922938	1423.678337	-0.908096005	8.40E-30	1.80E-28
ENSG00000198951	NAGA	257.7712484	638.5877612	-1.309460028	8.57E-30	1.84E-28
ENSG00000063854	HAGH	201.7909614	544.4923661	-1.431514899	9.56E-30	2.04E-28
ENSG00000169026	SLC49A3	129.685625	459.3994314	-1.824578672	1.03E-29	2.20E-28
ENSG00000165802	NSMF	582.7647802	1210.046531	-1.053831707	1.06E-29	2.26E-28
ENSG00000123689	G0S2	333.4967721	1.808672008	7.57000893	1.11E-29	2.35E-28
ENSG00000136040	PLXNC1	726.3667436	272.4831178	1.413044203	1.24E-29	2.64E-28
ENSG00000061938	TNK2	526.7128347	1129.879142	-1.101512625	1.27E-29	2.68E-28
ENSG00000064042	LIMCH1	870.8337979	1584.210234	-0.863871495	1.45E-29	3.07E-28
ENSG00000067177	PHKA1	42.44472038	243.1892637	-2.524726688	1.66E-29	3.51E-28
ENSG00000155792	DEPTOR	13.6711735	161.674856	-3.560741345	1.75E-29	3.71E-28
ENSG00000055813	CCDC85A	73.51390591	304.3082721	-2.051815193	1.82E-29	3.84E-28
ENSG00000102554	KLF5	73.21370826	314.4900577	-2.10487809	2.04E-29	4.31E-28
ENSG00000181104	F2R	223.5687001	717.353986	-1.679224552	2.26E-29	4.75E-28
ENSG00000165434	PGM2L1	723.7864294	1350.02225	-0.898783556	2.55E-29	5.35E-28
ENSG00000119938	PPP1R3C	709.8955621	1318.325538	-0.893203846	3.32E-29	6.98E-28
ENSG00000157551	KCNJ15	453.4101263	142.0101856	1.675549671	3.68E-29	7.71E-28
ENSG00000117069	ST6GALNAC5	213.4916514	583.7084199	-1.451410687	4.23E-29	8.85E-28
ENSG00000223764	LINC02593	13.49841133	158.39902	-3.559648826	4.30E-29	9.01E-28
ENSG00000108551	RASD1	182.0849944	8.91463509	4.368609328	4.54E-29	9.50E-28
ENSG00000142733	MAP3K6	356.3626361	783.1275257	-1.136195046	4.66E-29	9.74E-28
ENSG00000257354	AC048341.1	696.0699933	273.545155	1.347663938	5.67E-29	1.18E-27
ENSG00000179981	TSHZ1	1440.082428	773.7852948	0.896856486	5.98E-29	1.25E-27
ENSG00000109610	SOD3	61.28578104	307.339678	-2.328333761	7.02E-29	1.46E-27
ENSG00000172059	KLF11	448.0428685	141.9359334	1.658367614	1.10E-28	2.28E-27
ENSG00000142327	RNPEPL1	566.5442345	1166.440337	-1.042287376	1.17E-28	2.42E-27
ENSG00000099814	CEP170B	1101.579707	1884.825397	-0.775006607	1.38E-28	2.86E-27
ENSG00000109790	KLHL5	1048.343156	537.5775657	0.963993377	1.64E-28	3.39E-27
ENSG00000134882	UBAC2	1131.52786	1952.213096	-0.787307549	1.70E-28	3.51E-27
ENSG00000156265	MAP3K7CL	187.4359053	530.7590847	-1.499319934	1.95E-28	4.01E-27
ENSG00000179604	CDC42EP4	370.3888596	104.3298949	1.826314679	2.76E-28	5.68E-27
ENSG00000106819	ASPN	42.30731994	237.3407968	-2.485859601	3.01E-28	6.16E-27
ENSG00000197858	GPAA1	980.8557573	1680.608565	-0.777515845	3.02E-28	6.19E-27
ENSG00000171502	COL24A1	415.9575735	116.2726769	1.836389057	3.30E-28	6.75E-27
ENSG00000206527	HACD2	783.3418842	1446.257827	-0.884601025	4.93E-28	1.00E-26
ENSG00000274180	NATD1	463.4960809	977.0413037	-1.07731795	6.19E-28	1.26E-26
ENSG00000143867	OSR1	287.3406692	36.1197919	2.987080487	6.31E-28	1.28E-26
ENSG00000135679	MDM2	2492.441649	1460.032586	0.771872268	6.38E-28	1.29E-26
ENSG00000131370	SH3BP5	116.7177924	403.5927792	-1.788696982	6.86E-28	1.39E-26
ENSG00000204525	HLA-C	1030.662803	1882.556416	-0.86894151	7.13E-28	1.44E-26
ENSG00000106780	MEGF9	254.6150329	661.495378	-1.377742872	8.32E-28	1.68E-26
ENSG00000101460	MAP1LC3A	149.7525281	455.838446	-1.606598423	1.10E-27	2.21E-26
ENSG00000141458	NPC1	1009.563005	1769.466397	-0.809835891	1.16E-27	2.33E-26
ENSG00000154127	UBASH3B	498.8400234	175.0774967	1.511771175	1.25E-27	2.52E-26
ENSG00000164484	TMEM200A	276.6121245	691.0194458	-1.322999218	1.30E-27	2.62E-26
ENSG00000110047	EHD1	838.4405931	1495.45207	-0.833891633	1.35E-27	2.70E-26
ENSG00000151208	DLG5	992.0517991	1719.40157	-0.793485517	1.41E-27	2.82E-26
ENSG00000121753	ADGRB2	491.3815074	166.8860351	1.559287758	1.63E-27	3.27E-26
ENSG00000146966	DENND2A	242.1756121	27.70038352	3.137157609	1.75E-27	3.50E-26
ENSG00000147324	MFHAS1	313.2130361	734.0719605	-1.22843927	2.13E-27	4.26E-26
ENSG00000135636	DYSF	14.21621109	187.6527617	-3.734264445	2.26E-27	4.51E-26
ENSG00000073060	SCARB1	122.4334394	394.0294116	-1.686223494	2.46E-27	4.91E-26
ENSG00000160233	LRRC3	44.95709769	245.9835976	-2.448805272	2.64E-27	5.25E-26
ENSG00000019991	HGF	164.0404397	10.82098338	3.918225275	2.70E-27	5.37E-26
ENSG00000137449	CPEB2	355.9931595	810.0247028	-1.187342664	3.37E-27	6.68E-26
ENSG00000170801	HTRA3	385.0062358	99.53433232	1.952525945	3.39E-27	6.71E-26
ENSG00000163827	LRRC2	86.75087984	334.3011788	-1.948599978	4.29E-27	8.47E-26
ENSG00000160161	CILP2	4.045172504	160.7349355	-5.310931292	5.15E-27	1.02E-25
ENSG00000149782	PLCB3	666.2449993	1269.361742	-0.930563809	5.95E-27	1.17E-25

ENSG00000170345	FOS	707.816437	215.564239	1.716470853	6.49E-27	1.27E-25
ENSG00000131634	TMEM204	505.1641468	1091.023232	-1.110443807	7.61E-27	1.49E-25
ENSG00000121039	RDH10	259.0305688	638.8111987	-1.304195099	9.03E-27	1.76E-25
ENSG00000198736	MSRB1	224.1577183	559.2283832	-1.319903666	1.03E-26	2.00E-25
ENSG00000132780	NASP	998.2653593	501.5709705	0.993551737	1.07E-26	2.09E-25
ENSG00000152217	SETBP1	135.2695973	418.8057439	-1.631121983	1.33E-26	2.58E-25
ENSG00000167778	SPRYD3	531.0477263	1061.876743	-0.998295926	1.34E-26	2.59E-25
ENSG00000082196	C1QTNF3	15.60019637	159.0944489	-3.3512802	1.37E-26	2.66E-25
ENSG00000175832	ETV4	418.3095751	126.2812837	1.727876436	1.43E-26	2.78E-25
ENSG00000121281	ADCY7	223.9450061	603.0185546	-1.430582996	1.45E-26	2.81E-25
ENSG00000158122	PRXL2C	219.619656	552.671271	-1.330892753	1.52E-26	2.94E-25
ENSG00000130513	GDF15	620.801566	201.167418	1.626718466	1.54E-26	2.97E-25
ENSG00000116117	PARD3B	340.5457954	749.6018041	-1.138908179	1.74E-26	3.36E-25
ENSG00000112419	PHACTR2	926.2598247	1697.456784	-0.872873443	1.79E-26	3.45E-25
ENSG00000147010	SH3KBP1	1767.950497	1009.475743	0.808591364	2.09E-26	4.03E-25
ENSG00000117643	MAN1C1	35.34106165	214.8564422	-2.606016879	2.12E-26	4.08E-25
ENSG00000168497	CAVIN2	8.875539901	139.7140631	-3.957440336	2.61E-26	5.02E-25
ENSG00000105419	MEIS3	668.3068585	1279.666894	-0.937104498	2.84E-26	5.45E-25
ENSG00000138378	STAT4	266.9928463	59.46214505	2.169758146	4.58E-26	8.75E-25
ENSG00000149541	B3GAT3	336.314752	736.792165	-1.131492164	5.55E-26	1.06E-24
ENSG00000116001	TIA1	1471.848627	820.3911252	0.842933615	5.63E-26	1.07E-24
ENSG00000010810	FYN	978.6505834	514.2372663	0.928090691	6.03E-26	1.15E-24
ENSG00000154822	PLCL2	272.2367652	57.82470457	2.238660567	7.43E-26	1.41E-24
ENSG00000186907	RTN4RL2	240.161431	1.767415745	7.097744024	8.05E-26	1.52E-24
ENSG00000183098	GPC6	667.3387278	275.2438652	1.276200138	8.18E-26	1.55E-24
ENSG00000143067	ZNF697	527.4077735	181.9645767	1.53732121	8.61E-26	1.63E-24
ENSG00000187210	GCNT1	195.6622063	544.5435719	-1.476175913	8.94E-26	1.69E-24
ENSG00000087076	HSD17B14	330.9807174	731.1945608	-1.14491124	9.53E-26	1.80E-24
ENSG00000124177	CHD6	1414.853917	804.6633671	0.814513154	1.09E-25	2.05E-24
ENSG00000100626	GALNT16	7.686479574	138.9739673	-4.204551171	1.10E-25	2.06E-24
ENSG00000182463	TSHZ2	445.9505107	147.0938902	1.601424121	1.83E-25	3.43E-24
ENSG00000165533	TTC8	1132.706951	593.6677199	0.93143023	1.94E-25	3.64E-24
ENSG00000068137	PLEKHH3	177.4753219	474.2942057	-1.418544231	2.27E-25	4.24E-24
ENSG00000122861	PLAU	108.5673409	407.9965961	-1.909315654	2.39E-25	4.47E-24
ENSG00000137573	SULF1	72231.4643	38121.31802	0.922019668	2.47E-25	4.62E-24
ENSG00000178980	SELENOW	473.09121	916.0390992	-0.953617285	2.53E-25	4.72E-24
ENSG00000066279	ASPM	537.4739085	194.7912367	1.465796456	2.76E-25	5.15E-24
ENSG00000154447	SH3RF1	396.6086547	811.089807	-1.0312834	3.49E-25	6.50E-24
ENSG00000157514	TSC22D3	244.9885644	583.9726054	-1.254462477	3.65E-25	6.79E-24
ENSG00000164099	PRSS12	943.178413	1611.328114	-0.773403742	4.26E-25	7.90E-24
ENSG00000066629	EML1	991.7046457	521.5540651	0.926784158	6.54E-25	1.21E-23
ENSG00000122691	TWIST1	666.2552432	1250.079987	-0.908039419	7.49E-25	1.39E-23
ENSG00000165105	RASEF	12.16207994	147.900675	-3.604040126	7.68E-25	1.42E-23
ENSG00000188211	NCR3LG1	142.2055215	427.5216159	-1.58761984	8.02E-25	1.48E-23
ENSG00000162066	AMDHD2	252.0654962	697.5144188	-1.466681247	9.34E-25	1.72E-23
ENSG00000127241	MASP1	1139.832506	530.5668847	1.10253741	9.46E-25	1.74E-23
ENSG00000074370	ATP2A3	966.1652563	417.8263871	1.210126197	9.60E-25	1.77E-23
ENSG00000118898	PPL	18.18697864	161.7151572	-3.143535468	1.01E-24	1.85E-23
ENSG00000182287	AP1S2	564.8088766	1058.807824	-0.906119257	1.03E-24	1.89E-23
ENSG00000124813	RUNX2	1121.660084	561.4079226	0.999754693	1.10E-24	2.02E-23
ENSG00000145016	RUBCN	286.1526843	669.9614829	-1.227817588	1.19E-24	2.17E-23
ENSG00000242732	RTL5	71.57196297	275.6082067	-1.946770789	1.30E-24	2.37E-23
ENSG00000139278	GLIPR1	825.0428703	1412.852964	-0.776131193	1.33E-24	2.41E-23
ENSG00000076706	MCAM	167.8308356	447.1911732	-1.412432736	1.58E-24	2.87E-23
ENSG00000211584	SLC48A1	142.4178605	407.2622841	-1.51677236	1.77E-24	3.21E-23
ENSG00000097021	ACOT7	124.1977771	391.5479926	-1.654749834	1.77E-24	3.22E-23
ENSG00000133687	TMTC1	706.7719879	302.7316545	1.221514096	2.17E-24	3.94E-23
ENSG00000197927	C2orf27A	280.7789084	61.76737641	2.185918618	3.81E-24	6.88E-23
ENSG00000126391	FRMD8	461.766194	923.7567898	-1.001017018	5.26E-24	9.49E-23
ENSG00000104321	TRPA1	226.7876604	39.50342945	2.525402117	5.29E-24	9.54E-23
ENSG00000167642	SPINT2	5.558037786	118.0893139	-4.398614587	5.62E-24	1.01E-22
ENSG00000114346	ECT2	866.3603843	439.1898127	0.980496998	6.26E-24	1.13E-22
ENSG00000185499	MUC1	59.51892746	244.7147127	-2.039100986	6.40E-24	1.15E-22
ENSG00000171848	RRM2	264.4700606	61.57096234	2.102472505	6.69E-24	1.20E-22
ENSG00000155465	SLC7A7	283.9873419	68.9883398	2.040580566	6.76E-24	1.21E-22
ENSG00000134070	IRAK2	230.4572533	41.6770335	2.467015036	6.82E-24	1.22E-22

ENSG00000064393	HIPK2	1119.5901	588.3775694	0.928399294	7.74E-24	1.38E-22
ENSG00000271383	NBPF19	1131.350246	609.3694469	0.892602564	8.79E-24	1.57E-22
ENSG00000105726	ATP13A1	627.6392114	1121.271306	-0.837554834	9.83E-24	1.75E-22
ENSG00000165072	MAMDC2	19.72040176	171.9355306	-3.12778097	1.03E-23	1.84E-22
ENSG00000147955	SIGMAR1	714.6684752	1288.957927	-0.851260915	1.09E-23	1.94E-22
ENSG00000109472	CPE	29.19275392	193.9749413	-2.744551131	1.17E-23	2.07E-22
ENSG00000116604	MEF2D	1036.077875	1794.950989	-0.792425008	1.39E-23	2.47E-22
ENSG00000113387	SUB1	1912.721298	1102.189064	0.795778112	1.51E-23	2.67E-22
ENSG00000155366	RHOC	1110.59456	1876.505833	-0.75662997	1.62E-23	2.86E-22
ENSG00000168477	TNXB	13.5566581	142.9602072	-3.387881777	1.85E-23	3.26E-22
ENSG00000182307	C8orf33	430.4767769	856.9181218	-0.993305758	2.02E-23	3.57E-22
ENSG00000130956	HABP4	360.8310662	737.1829393	-1.029655958	2.26E-23	3.97E-22
ENSG00000010404	IDS	704.554612	1252.247496	-0.83006032	2.42E-23	4.25E-22
ENSG00000002745	WNT16	133.21861	3.780208803	5.114104091	2.68E-23	4.71E-22
ENSG00000163083	INHBB	8.482267357	122.6215846	-3.861017004	2.81E-23	4.93E-22
ENSG00000112759	SLC29A1	42.40514355	208.1524082	-2.296052877	2.94E-23	5.14E-22
ENSG00000089327	FXYD5	703.3321681	1249.708242	-0.829329189	3.46E-23	6.05E-22
ENSG00000134324	LPIN1	608.217308	1074.609941	-0.821432793	3.63E-23	6.34E-22
ENSG00000104812	GYS1	449.4502063	861.4612358	-0.937979951	4.00E-23	6.97E-22
ENSG00000198171	DDRGK1	422.3027182	844.9921557	-1.000357433	4.75E-23	8.27E-22
ENSG00000149218	ENDOD1	95.68781029	312.3726608	-1.70903177	5.54E-23	9.62E-22
ENSG00000134874	DZIP1	1698.167308	991.3660536	0.776238535	5.61E-23	9.74E-22
ENSG00000177363	LRRN4CL	536.4703459	1041.884822	-0.956369562	6.93E-23	1.20E-21
ENSG00000276170	AC244153.1	25.9030701	184.3930686	-2.832229107	7.35E-23	1.27E-21
ENSG00000154451	GBP5	129.4998485	11.26152446	3.527409474	7.76E-23	1.34E-21
ENSG00000205364	MT1M	167.1254223	23.77607307	2.810967116	8.77E-23	1.51E-21
ENSG00000133639	BTG1	1518.500979	886.3673226	0.77703691	9.06E-23	1.56E-21
ENSG00000123358	NR4A1	270.5126081	726.0553089	-1.426343357	9.43E-23	1.62E-21
ENSG00000100490	CDKL1	24.95086288	174.7021441	-2.798775719	9.91E-23	1.70E-21
ENSG00000187239	FNBP1	591.0387109	1066.354246	-0.851919563	9.99E-23	1.71E-21
ENSG00000184292	TACSTD2	131.6956854	11.59066461	3.508072655	1.04E-22	1.78E-21
ENSG00000087510	TFAP2C	690.5049698	323.1642717	1.096319667	1.14E-22	1.95E-21
ENSG00000181458	TMEM45A	403.3759374	794.8933857	-0.978181283	1.18E-22	2.01E-21
ENSG00000141556	TBCD	670.0640921	1164.591138	-0.796763492	1.22E-22	2.09E-21
ENSG00000103569	AQP9	237.1541617	41.9291255	2.501799027	1.34E-22	2.29E-21
ENSG00000119673	ACOT2	345.5886864	711.8935963	-1.041457493	1.46E-22	2.49E-21
ENSG00000183856	IQGAP3	354.2874135	109.93826	1.689092038	1.73E-22	2.94E-21
ENSG00000073792	IGF2BP2	1476.446183	875.4160687	0.754066005	1.91E-22	3.25E-21
ENSG00000169136	ATF5	544.1262124	240.0170151	1.180500567	1.94E-22	3.29E-21
ENSG00000082397	EPB41L3	802.3239001	378.3630538	1.08515047	2.49E-22	4.21E-21
ENSG00000068438	FTSJ1	613.1832864	1126.422396	-0.878511237	2.68E-22	4.53E-21
ENSG00000196730	DAPK1	87.14164065	362.9195351	-2.065171439	2.94E-22	4.96E-21
ENSG00000133424	LARGE1	305.0233568	660.6580691	-1.114594784	3.82E-22	6.44E-21
ENSG00000115159	GPD2	556.6910624	227.5418819	1.292029553	4.10E-22	6.90E-21
ENSG00000183496	MEX3B	219.3071662	519.3385078	-1.242326076	4.18E-22	7.04E-21
ENSG00000153993	SEMA3D	196.4326916	486.1243443	-1.30868466	4.54E-22	7.63E-21
ENSG00000171385	KCND3	43.2064023	205.3403485	-2.251823443	4.64E-22	7.80E-21
ENSG00000111962	UST	790.7459334	380.8395167	1.053790675	4.70E-22	7.88E-21
ENSG00000147889	CDKN2A	119.5640339	348.4956744	-1.543943953	5.18E-22	8.69E-21
ENSG00000132329	RAMP1	7.615218397	117.3815171	-3.964275512	5.52E-22	9.25E-21
ENSG00000140931	CMTM3	704.5702325	1220.458937	-0.792814305	6.17E-22	1.03E-20
ENSG00000081760	AACS	471.5966415	901.5019517	-0.934977452	6.19E-22	1.03E-20
ENSG00000112679	DUSP22	194.8451666	479.0326964	-1.297784648	6.28E-22	1.05E-20
ENSG00000196547	MAN2A2	409.1970175	816.0751383	-0.995358227	6.38E-22	1.06E-20
ENSG00000161835	GRASP	104.3516391	325.5380329	-1.63975339	6.50E-22	1.08E-20
ENSG00000106236	NPTX2	138.4040934	11.49989181	3.582310435	6.67E-22	1.11E-20
ENSG00000170775	GPR37	34.4814618	179.8794787	-2.380144556	8.98E-22	1.49E-20
ENSG00000153029	MR1	325.8322949	669.1138677	-1.038355011	9.16E-22	1.52E-20
ENSG00000113645	WWC1	154.5554504	18.3724437	3.078854252	9.56E-22	1.59E-20
ENSG00000164171	ITGA2	732.3611856	329.6995055	1.150959562	9.81E-22	1.62E-20
ENSG00000248334	WHAMMP2	89.47543575	299.9216486	-1.74523137	1.03E-21	1.70E-20
ENSG00000136560	TANK	868.2328597	464.881109	0.90188031	1.04E-21	1.72E-20
ENSG00000169255	B3GALNT1	161.2039084	431.3655499	-1.417081453	1.04E-21	1.72E-20
ENSG00000183287	CCBE1	945.5399079	471.7889687	1.003692242	1.09E-21	1.81E-20
ENSG00000154265	ABCA5	1293.098897	677.294957	0.933458034	1.40E-21	2.29E-20
ENSG00000272841	AL139393.2	122.2421918	375.389462	-1.616406197	1.48E-21	2.43E-20

ENSG00000067836	ROGDI	52.13713952	217.0881797	-2.054910505	1.67E-21	2.74E-20
ENSG00000118596	SLC16A7	1188.540382	676.462859	0.81315783	1.73E-21	2.83E-20
ENSG00000136928	GABBR2	68.87003736	291.6378236	-2.086470853	1.75E-21	2.86E-20
ENSG00000125354	6-Sep	203.4596182	481.7094229	-1.244970976	1.88E-21	3.08E-20
ENSG00000127863	TNFRSF19	454.0915873	906.2202611	-0.995746312	1.92E-21	3.14E-20
ENSG00000130150	MOSPD2	627.638485	1133.619563	-0.85237698	2.33E-21	3.80E-20
ENSG00000128335	APOL2	372.0158913	746.8165711	-1.006150274	2.36E-21	3.85E-20
ENSG00000166002	SMCO4	100.5102852	330.248061	-1.713021464	2.43E-21	3.95E-20
ENSG00000101096	NFATC2	144.5254897	19.54706292	2.882616164	2.64E-21	4.29E-20
ENSG00000164023	SGMS2	277.7094561	610.4846451	-1.134825765	2.84E-21	4.61E-20
ENSG00000128309	MPST	427.7259375	810.2799683	-0.921726521	3.36E-21	5.43E-20
ENSG00000110921	MVK	255.1374161	618.2410299	-1.279458867	3.47E-21	5.61E-20
ENSG00000156381	ANKRD9	328.9725368	752.3684339	-1.191401559	3.47E-21	5.61E-20
ENSG00000145244	CORIN	8.157112206	113.2291533	-3.802173928	3.49E-21	5.63E-20
ENSG00000159167	STC1	156.3981253	23.58927605	2.732430657	3.72E-21	5.99E-20
ENSG00000151729	SLC25A4	71.58821138	281.2574264	-1.968353322	4.15E-21	6.68E-20
ENSG00000132031	MATN3	42.30049877	217.809238	-2.362931557	4.20E-21	6.77E-20
ENSG00000073008	PVR	430.4151316	818.7450227	-0.927353693	4.33E-21	6.97E-20
ENSG00000109861	CTSC	486.4159483	207.2046985	1.231004217	4.42E-21	7.11E-20
ENSG00000196588	MRTFA	575.831093	1039.23289	-0.852685846	6.07E-21	9.73E-20
ENSG00000103449	SALL1	286.7135028	85.73776468	1.743191701	6.80E-21	1.09E-19
ENSG00000136717	BIN1	905.7438279	500.1302393	0.856767678	6.89E-21	1.10E-19
ENSG00000149084	HSD17B12	540.7176136	963.121603	-0.832641564	6.94E-21	1.11E-19
ENSG00000183023	SLC8A1	82.09399688	277.377381	-1.759140318	7.23E-21	1.16E-19
ENSG00000187837	HIST1H1C	606.6046369	269.3693673	1.1722545	8.54E-21	1.36E-19
ENSG00000139874	SSTR1	24.8652371	178.4951676	-2.833456381	8.78E-21	1.40E-19
ENSG00000120129	DUSP1	1795.112702	1037.581756	0.791595762	1.12E-20	1.79E-19
ENSG00000105976	MET	580.5092479	1060.329083	-0.867648454	1.20E-20	1.92E-19
ENSG00000283154	IQCJ-SCHIP1	165.3068884	443.3440415	-1.422217781	1.22E-20	1.94E-19
ENSG00000117594	HSD11B1	541.2977099	0.702840672	9.591795807	1.39E-20	2.20E-19
ENSG00000204209	DAXX	779.7497699	410.9068644	0.92474126	1.43E-20	2.27E-19
ENSG00000151892	GFRA1	912.3687811	1620.159606	-0.829409037	1.56E-20	2.46E-19
ENSG00000162618	ADGRL4	771.3381977	353.5295672	1.126912587	1.78E-20	2.82E-19
ENSG00000183671	GPR1	263.7626733	65.34096355	2.013142998	1.82E-20	2.87E-19
ENSG00000262655	SPON1	560.9540407	229.2833116	1.292365106	1.95E-20	3.07E-19
ENSG00000139998	RAB15	331.1209126	673.9947629	-1.02654609	1.96E-20	3.09E-19
ENSG00000173846	PLK3	408.8439539	811.2959368	-0.990441263	2.31E-20	3.63E-19
ENSG00000164976	MYORG	29.3539462	178.1408449	-2.602448881	2.34E-20	3.67E-19
ENSG00000178814	OPLAH	150.9990228	436.0965387	-1.532836177	2.35E-20	3.69E-19
ENSG00000182218	HHIPL1	267.928456	570.2305719	-1.089380451	2.39E-20	3.75E-19
ENSG00000230838	LINC01614	35.77966088	205.3012525	-2.51391283	2.43E-20	3.82E-19
ENSG00000105245	NUMBL	361.025901	721.105179	-0.999055506	3.15E-20	4.93E-19
ENSG00000170955	CAVIN3	553.2599799	1070.81115	-0.952644941	3.28E-20	5.13E-19
ENSG00000151617	EDNRA	261.7896457	67.11333545	1.964290486	3.58E-20	5.59E-19
ENSG00000162595	DIRAS3	113.6295097	4.542880073	4.639506032	3.68E-20	5.74E-19
ENSG00000141232	TOB1	399.8035643	767.80507	-0.940994462	3.69E-20	5.75E-19
ENSG00000241749	RPSAP52	113.8475042	7.459035471	3.945066261	3.87E-20	6.03E-19
ENSG00000137103	TMEM8B	207.6931733	471.211078	-1.181390886	4.08E-20	6.35E-19
ENSG00000129048	ACKR4	1518.618861	847.9757049	0.840312075	4.43E-20	6.90E-19
ENSG00000188522	FAM83G	284.4053172	596.140243	-1.067002071	4.74E-20	7.36E-19
ENSG00000120896	SORBS3	607.262302	1080.007979	-0.829633631	4.80E-20	7.45E-19
ENSG00000060140	STYK1	105.5881688	6.293373536	4.064894903	5.11E-20	7.92E-19
ENSG00000104320	NBN	1131.921798	634.2233356	0.835940725	5.12E-20	7.93E-19
ENSG00000142224	IL19	104.768868	4.923590552	4.414098347	5.14E-20	7.96E-19
ENSG00000143367	TUFT1	246.611102	535.2905892	-1.11842086	5.46E-20	8.45E-19
ENSG00000154640	BTG3	635.1488782	303.4904311	1.06493171	5.62E-20	8.69E-19
ENSG00000133048	CHI3L1	126.1292215	10.05831211	3.638618607	5.99E-20	9.25E-19
ENSG00000145779	TNFAIP8	571.9315826	260.4993376	1.135190837	6.01E-20	9.28E-19
ENSG00000106483	SFRP4	2.947742542	108.3129745	-5.259243416	6.81E-20	1.05E-18
ENSG00000165996	HACD1	402.787112	757.4576237	-0.911159659	7.03E-20	1.08E-18
ENSG00000011426	ANLN	604.3484347	248.8383768	1.280960701	7.81E-20	1.20E-18
ENSG00000242802	AP5Z1	818.5822614	1454.187567	-0.829223268	8.88E-20	1.36E-18
ENSG00000196850	PPTC7	609.2389126	285.655803	1.093117256	9.21E-20	1.41E-18
ENSG00000241644	INMT	2.558869649	121.0038996	-5.59503733	1.02E-19	1.57E-18
ENSG00000158716	DUSP23	77.18994678	263.2920809	-1.77131978	1.09E-19	1.67E-18
ENSG00000176842	IRX5	326.6031252	682.2898282	-1.06252121	1.11E-19	1.70E-18

ENSG00000135324	MRAP2	16.43996064	138.4763915	-3.071594366	1.19E-19	1.81E-18
ENSG00000162976	PQLC3	109.4124818	318.9788669	-1.541916238	1.32E-19	2.02E-18
ENSG00000231924	PSG1	411.0550822	139.5771844	1.560636734	1.47E-19	2.24E-18
ENSG00000134516	DOCK2	522.4250132	932.731149	-0.835507635	1.64E-19	2.51E-18
ENSG00000119698	PPP4R4	123.2735527	2.137812159	5.869844963	1.87E-19	2.84E-18
ENSG00000048740	CELF2	130.8257756	363.8756567	-1.477593849	1.89E-19	2.87E-18
ENSG00000108960	MMD	277.5435151	75.30685081	1.881886118	2.04E-19	3.09E-18
ENSG00000125375	ATP5S	128.1052703	340.7613117	-1.411716041	2.22E-19	3.36E-18
ENSG00000174600	CMKLR1	0	297.573993	-10.75528458	2.25E-19	3.41E-18
ENSG00000204389	HSPA1A	383.6680165	740.559793	-0.949749255	2.33E-19	3.53E-18
ENSG00000164929	BAALC	31.64213575	170.2714684	-2.424306966	2.43E-19	3.67E-18
ENSG00000128045	RASL11B	121.3747697	8.61302656	3.800118514	2.58E-19	3.89E-18
ENSG00000142279	WTIP	565.3800373	981.1893019	-0.795434324	2.85E-19	4.30E-18
ENSG00000073711	PPP2R3A	1169.815933	678.788354	0.784893691	2.86E-19	4.31E-18
ENSG00000100344	PNPLA3	885.3489508	486.697505	0.862667996	2.92E-19	4.40E-18
ENSG00000213722	DDAH2	544.9761062	981.9485626	-0.850840568	3.41E-19	5.14E-18
ENSG00000121957	GPSM2	661.7412234	327.7931572	1.0142492	4.07E-19	6.11E-18
ENSG00000142149	HUNK	373.9493138	721.6810291	-0.948963162	4.27E-19	6.40E-18
ENSG00000106785	TRIM14	110.2359034	308.8790855	-1.487798306	4.99E-19	7.48E-18
ENSG00000088881	EBF4	126.5815333	13.39768456	3.243395108	5.11E-19	7.66E-18
ENSG00000117602	RCAN3	226.7911774	495.3085842	-1.125863585	5.14E-19	7.69E-18
ENSG00000204103	MAFB	562.4786026	242.2467439	1.213895745	5.21E-19	7.78E-18
ENSG00000168014	C2CD3	506.4486553	959.6071494	-0.923239794	6.64E-19	9.90E-18
ENSG00000189339	SLC35E2B	1333.039733	784.0980238	0.764737906	7.51E-19	1.12E-17
ENSG00000102802	MEDAG	185.6071276	42.74571617	2.118524759	7.94E-19	1.18E-17
ENSG00000105855	ITGB8	220.7269566	59.09174864	1.90353696	8.61E-19	1.28E-17
ENSG00000229152	ANKRD10-IT1	468.844825	213.4448496	1.135196827	1.04E-18	1.54E-17
ENSG00000103047	TANGO6	217.7255556	495.5483697	-1.186378619	1.06E-18	1.57E-17
ENSG00000099785	2-Mar	190.6778409	435.1943202	-1.189409252	1.14E-18	1.68E-17
ENSG00000049192	ADAMTS6	690.9823576	356.8439459	0.95371504	1.26E-18	1.86E-17
ENSG00000233695	GAS6-AS1	134.36181	399.950314	-1.576124647	1.38E-18	2.03E-17
ENSG00000099849	RASSF7	11.39725423	108.6657516	-3.242537571	1.48E-18	2.18E-17
ENSG00000130529	TRPM4	545.6667156	975.2201574	-0.839259284	1.55E-18	2.27E-17
ENSG00000175745	NR2F1	238.1061383	518.3214327	-1.12285742	1.70E-18	2.49E-17
ENSG00000221926	TRIM16	363.8060693	136.5458157	1.412570116	1.76E-18	2.59E-17
ENSG00000067113	PLPP1	508.6679591	895.1533843	-0.814594679	1.82E-18	2.67E-17
ENSG00000168234	TTC39C	637.1691782	316.6828267	1.007951594	1.88E-18	2.75E-17
ENSG00000132854	KANK4	123.766627	330.8029427	-1.416943964	2.10E-18	3.07E-17
ENSG00000198795	ZNF521	934.1866261	520.918975	0.843190538	2.19E-18	3.19E-17
ENSG00000010278	CD9	170.3481502	415.4275094	-1.285492948	2.29E-18	3.34E-17
ENSG00000180573	HIST1H2AC	502.5386704	236.8080197	1.085361136	2.83E-18	4.11E-17
ENSG00000136378	ADAMTS7	477.8033846	865.5398104	-0.857472994	2.89E-18	4.20E-17
ENSG00000185760	KCNQ5	8.468625012	96.45065124	-3.515522694	2.91E-18	4.23E-17
ENSG00000177732	SOX12	822.8744124	454.4981661	0.856845374	3.07E-18	4.46E-17
ENSG00000165949	IFI27	81.40043235	265.4670868	-1.707425668	3.29E-18	4.77E-17
ENSG00000167994	RAB3IL1	306.8126521	620.0124017	-1.014272016	3.37E-18	4.89E-17
ENSG00000183691	NOG	24.81148431	142.2431951	-2.515693303	3.77E-18	5.46E-17
ENSG00000169271	HSPB3	12.95786772	115.0346277	-3.141360622	3.92E-18	5.67E-17
ENSG00000182612	TSPAN10	39.71811615	173.9135933	-2.126936572	3.98E-18	5.76E-17
ENSG00000178307	TMEM11	271.1641041	565.6978621	-1.059929509	4.20E-18	6.07E-17
ENSG00000123096	SSPN	208.1655052	459.2456141	-1.141234587	4.26E-18	6.14E-17
ENSG00000152137	HSPB8	91.48351796	277.4896678	-1.604096276	4.47E-18	6.45E-17
ENSG00000115226	FNDC4	155.2879639	395.502737	-1.350948275	4.69E-18	6.75E-17
ENSG00000120725	SIL1	453.28565	825.958234	-0.86537579	4.83E-18	6.95E-17
ENSG00000185565	LSAMP	254.9909575	70.10243141	1.862907917	5.03E-18	7.23E-17
ENSG00000179431	FJX1	230.8565304	533.5298431	-1.206283877	5.25E-18	7.53E-17
ENSG00000095209	TMEM38B	521.7794516	251.5837475	1.052238098	6.40E-18	9.17E-17
ENSG00000086289	EPDR1	330.6707394	639.6289574	-0.950889076	6.74E-18	9.65E-17
ENSG00000198523	PLN	24.2682403	146.3231474	-2.600546331	7.25E-18	1.04E-16
ENSG00000167363	FN3K	39.6312345	180.1297057	-2.190113113	8.03E-18	1.14E-16
ENSG00000139567	ACVRL1	504.5647044	893.3503896	-0.824771639	8.97E-18	1.28E-16
ENSG00000106462	EZH2	290.7977185	95.28623962	1.608705988	9.05E-18	1.29E-16
ENSG00000161281	COX7A1	163.6995037	386.1772155	-1.238001322	9.65E-18	1.37E-16
ENSG00000144843	ADPRH	83.12833703	252.4907092	-1.602452849	9.91E-18	1.41E-16
ENSG00000124249	KCNK15	21.43196371	139.6300873	-2.712525025	1.01E-17	1.43E-16
ENSG00000261040	WFDC21P	270.235763	79.83170984	1.759408762	1.02E-17	1.44E-16

ENSG00000153179	RASSF3	331.9424462	644.3052605	-0.957808781	1.07E-17	1.52E-16
ENSG00000181982	CCDC149	197.5556127	445.0331797	-1.171639884	1.07E-17	1.52E-16
ENSG00000176532	PRR15	9.179603601	106.3430897	-3.547953361	1.08E-17	1.53E-16
ENSG00000168899	VAMP5	354.4209761	692.1746872	-0.966821993	1.09E-17	1.53E-16
ENSG00000164300	SERINC5	615.826937	1068.734365	-0.796029546	1.12E-17	1.58E-16
ENSG00000092345	DAZL	232.9078008	0	10.23327318	1.12E-17	1.58E-16
ENSG00000162433	AK4	52.48957936	200.345363	-1.93039418	1.18E-17	1.66E-16
ENSG00000155363	MOV10	679.9121464	356.8446429	0.929550712	1.29E-17	1.82E-16
ENSG00000117533	VAMP4	735.3791463	392.6715124	0.905681097	1.36E-17	1.90E-16
ENSG00000134057	CCNB1	440.7325545	184.2149336	1.260413756	1.45E-17	2.03E-16
ENSG00000138604	GLCE	569.9283152	285.8876686	0.995781328	1.52E-17	2.13E-16
ENSG00000132970	WASF3	700.3835146	336.7426742	1.055858928	1.67E-17	2.33E-16
ENSG00000167797	CDK2AP2	249.364991	530.0696283	-1.088511582	1.85E-17	2.58E-16
ENSG00000168938	PPIC	558.4240028	1040.861939	-0.900238521	1.95E-17	2.71E-16
ENSG00000155158	TTC39B	511.2554395	233.1369692	1.134156713	1.96E-17	2.73E-16
ENSG00000048392	RRM2B	1310.962157	745.6842534	0.81402607	1.96E-17	2.73E-16
ENSG00000270069	MIR222HG	272.7380108	92.87456349	1.554759708	2.28E-17	3.16E-16
ENSG00000187193	MT1X	147.0732328	27.09191503	2.442334374	2.38E-17	3.29E-16
ENSG00000171488	LRRC8C	274.6947618	95.25580564	1.529317526	2.43E-17	3.36E-16
ENSG00000088826	SMOX	353.5955482	139.9289	1.337211605	2.58E-17	3.56E-16
ENSG00000105792	CFAP69	278.1725211	79.40518868	1.809605046	2.76E-17	3.80E-16
ENSG00000119950	MXI1	643.2244152	312.4316055	1.042346348	2.84E-17	3.91E-16
ENSG00000196460	RFX8	401.0899105	161.9445673	1.309408346	2.90E-17	4.00E-16
ENSG00000145794	MEGF10	397.2883882	0.351420336	10.04204904	3.04E-17	4.18E-16
ENSG00000023909	GCLM	1035.196392	585.838546	0.82145309	3.10E-17	4.26E-16
ENSG00000134247	PTGFRN	399.8757082	172.381536	1.213805294	3.12E-17	4.29E-16
ENSG00000205356	TECPR1	331.3952701	667.7498234	-1.01144153	3.35E-17	4.60E-16
ENSG00000275131	AC241952.1	563.3087427	247.7150085	1.184347124	3.36E-17	4.61E-16
ENSG00000166946	CCNDBP1	507.1479938	876.7330389	-0.789432587	3.47E-17	4.74E-16
ENSG00000138669	PRKG2	17.48838653	124.7225434	-2.84927932	3.86E-17	5.27E-16
ENSG00000164112	TMEM155	96.67144711	282.348578	-1.546708834	4.09E-17	5.58E-16
ENSG00000141337	ARSG	432.6823902	181.4020012	1.252608411	4.09E-17	5.58E-16
ENSG00000128567	PODXL	75.63140575	238.7207874	-1.657038568	4.36E-17	5.94E-16
ENSG00000233098	CCDC144NL-AS1	33.35746941	152.6195298	-2.194354513	4.46E-17	6.07E-16
ENSG00000143355	LHX9	12.67228941	106.8072678	-3.088348704	4.54E-17	6.17E-16
ENSG00000222009	BTBD19	536.363075	964.041736	-0.847713178	4.64E-17	6.31E-16
ENSG00000163344	PMVK	229.8949903	489.2145885	-1.088111666	4.74E-17	6.43E-16
ENSG00000175130	MARCKSL1	430.0986614	167.2696029	1.362520201	4.80E-17	6.52E-16
ENSG00000149380	P4HA3	531.9823632	925.7860342	-0.799587885	4.82E-17	6.54E-16
ENSG00000276023	DUSP14	628.0977364	1059.298329	-0.753656282	5.04E-17	6.83E-16
ENSG00000109736	MFSD10	404.0823277	783.0138985	-0.953823446	5.22E-17	7.06E-16
ENSG00000233901	LINC01503	713.5495345	347.3661448	1.03813514	5.26E-17	7.11E-16
ENSG00000231721	LINC-PINT	342.9697522	121.2533472	1.499712152	5.37E-17	7.25E-16
ENSG00000119508	NR4A3	306.9101025	114.2287528	1.427066702	5.50E-17	7.42E-16
ENSG00000125538	IL1B	378.3065947	0.351420336	9.971721289	6.48E-17	8.72E-16
ENSG00000088756	ARHGAP28	58.92633031	220.8877663	-1.909251489	6.74E-17	9.07E-16
ENSG00000091129	NRCAM	195.6570183	50.80154929	1.944675416	6.85E-17	9.20E-16
ENSG00000109929	SC5D	642.1260985	1091.645311	-0.76490159	7.11E-17	9.54E-16
ENSG00000050628	PTGER3	124.5103612	319.2808157	-1.358944049	7.14E-17	9.58E-16
ENSG00000187800	PEAR1	35.14478651	168.2032927	-2.262957352	8.00E-17	1.07E-15
ENSG00000225697	SLC26A6	566.6860346	288.9634912	0.972183009	8.82E-17	1.18E-15
ENSG00000152049	KCNE4	469.6848481	839.2432883	-0.839024774	9.25E-17	1.24E-15
ENSG00000104312	RIPK2	487.2373215	237.4474304	1.037331992	9.87E-17	1.32E-15
ENSG00000178607	ERN1	551.1868015	266.6178803	1.04910269	1.00E-16	1.33E-15
ENSG00000139428	MMAB	220.1770801	469.8931606	-1.09537357	1.02E-16	1.36E-15
ENSG00000090975	PITPNM2	82.88431368	242.9542004	-1.551309816	1.04E-16	1.38E-15
ENSG00000138650	PCDH10	41.6253253	174.1006856	-2.067176038	1.05E-16	1.40E-15
ENSG00000088325	TPX2	579.3175113	269.3706999	1.104684867	1.07E-16	1.42E-15
ENSG00000064205	WISP2	89.02507798	266.1242605	-1.577556702	1.08E-16	1.44E-15
ENSG00000106868	SUSD1	165.4085539	411.1574932	-1.317422019	1.31E-16	1.73E-15
ENSG00000115758	ODC1	252.0497814	531.4687465	-1.074555234	1.31E-16	1.74E-15
ENSG00000159164	SV2A	554.1478173	276.8588367	1.000783157	1.40E-16	1.86E-15
ENSG00000139508	SLC46A3	118.0827587	308.9390226	-1.385899725	1.45E-16	1.92E-15
ENSG00000105339	DENND3	136.9182581	345.9948776	-1.339675164	1.49E-16	1.97E-15
ENSG00000197646	PDCD1LG2	681.3157986	348.4323268	0.967781879	1.68E-16	2.22E-15
ENSG00000137166	FOXP4	157.2020844	373.5349342	-1.247169833	1.70E-16	2.24E-15

ENSG00000147471	PLPBP	238.3932272	484.8809744	-1.023545939	1.91E-16	2.52E-15
ENSG00000228221	LINC00578	8.896003418	97.63518279	-3.442295639	2.12E-16	2.79E-15
ENSG00000138696	BMPR1B	31.90285089	149.6535626	-2.225652992	2.22E-16	2.92E-15
ENSG00000068079	IFI35	125.5358078	324.2230179	-1.369207275	2.34E-16	3.07E-15
ENSG00000067445	TRO	567.7303197	267.3271776	1.085627371	2.52E-16	3.29E-15
ENSG00000176697	BDNF	112.2807918	291.4435535	-1.376345925	2.74E-16	3.58E-15
ENSG00000184588	PDE4B	429.2735365	193.1170944	1.153441424	2.76E-16	3.60E-15
ENSG00000165943	MOAP1	234.7285015	487.8470932	-1.054767066	2.91E-16	3.80E-15
ENSG00000109062	SLC9A3R1	150.2543115	350.9101014	-1.224847191	3.19E-16	4.15E-15
ENSG00000132535	DLG4	519.8149066	259.6173005	1.001146782	3.26E-16	4.25E-15
ENSG00000185885	IFITM1	145.4877562	384.707822	-1.405945914	3.33E-16	4.33E-15
ENSG00000125877	ITPA	398.9075314	726.0407357	-0.863030667	3.48E-16	4.52E-15
ENSG00000100739	BDKRB1	137.6880171	26.49871676	2.373417308	3.64E-16	4.73E-15
ENSG00000232187	FTH1P7	228.4189974	63.93858631	1.839144227	3.70E-16	4.80E-15
ENSG00000182013	PNMA8A	150.0122663	349.9655892	-1.221288244	3.75E-16	4.86E-15
ENSG00000112137	PHACTR1	163.6486117	38.10134749	2.10102439	3.78E-16	4.90E-15
ENSG00000172197	MBOAT1	23.59881668	138.7700592	-2.5524589	3.83E-16	4.96E-15
ENSG00000169515	CCDC8	220.7541511	502.3367943	-1.188512008	4.01E-16	5.19E-15
ENSG00000096060	FKBP5	30.8122421	142.662411	-2.209407886	4.82E-16	6.21E-15
ENSG00000116863	ADPRHL2	250.3601033	508.8912249	-1.023551042	5.27E-16	6.79E-15
ENSG00000185697	MYBL1	191.8407822	411.0060863	-1.099810534	5.69E-16	7.32E-15
ENSG00000166508	MCM7	708.0749516	388.875714	0.864965488	5.92E-16	7.61E-15
ENSG00000169857	AVEN	394.7951242	701.5480359	-0.828963296	6.56E-16	8.43E-15
ENSG00000152495	CAMK4	130.7694127	26.00836388	2.332059859	7.58E-16	9.72E-15
ENSG00000171345	KRT19	3.428968063	75.84602321	-4.466824505	8.86E-16	1.13E-14
ENSG00000065833	ME1	771.7778524	420.9528087	0.873879942	9.09E-16	1.16E-14
ENSG00000100100	PIK3IP1	504.2603859	862.097281	-0.774114892	9.91E-16	1.27E-14
ENSG00000113578	FGF1	94.00551117	269.0236452	-1.514379751	1.06E-15	1.35E-14
ENSG00000077684	JADE1	211.2904096	445.5974896	-1.075761519	1.18E-15	1.50E-14
ENSG00000159423	ALDH4A1	77.06106677	231.8416273	-1.589496058	1.20E-15	1.53E-14
ENSG00000248905	FMN1	482.6980034	839.3016425	-0.797547321	1.22E-15	1.55E-14
ENSG00000154654	NCAM2	17.16080985	120.3483942	-2.81058871	1.51E-15	1.92E-14
ENSG00000180263	FGD6	105.8868558	289.5344916	-1.453992128	1.53E-15	1.94E-14
ENSG00000127838	PNKD	688.508925	372.0214964	0.88754517	1.65E-15	2.09E-14
ENSG00000136824	SMC2	579.1276841	276.4488812	1.067626757	1.71E-15	2.17E-14
ENSG00000003436	TFPI	537.1625602	252.2894082	1.092038986	1.72E-15	2.17E-14
ENSG00000169231	THBS3	331.5960392	634.9345881	-0.93780158	1.75E-15	2.22E-14
ENSG00000164823	OSGIN2	615.465094	317.778602	0.953486235	1.81E-15	2.28E-14
ENSG00000114698	PLSCR4	546.6591517	950.8068417	-0.799091406	1.84E-15	2.32E-14
ENSG00000064115	TM7SF3	485.2330151	852.2357847	-0.813353571	1.99E-15	2.50E-14
ENSG00000135622	SEMA4F	85.48841569	250.0301763	-1.544571182	2.10E-15	2.64E-14
ENSG00000102763	VWA8	335.1629413	633.7141713	-0.920319227	2.28E-15	2.86E-14
ENSG00000132429	POPDC3	436.8457796	193.1122447	1.179819611	2.73E-15	3.41E-14
ENSG00000112139	MDGA1	530.1549776	232.5644814	1.187410621	2.86E-15	3.57E-14
ENSG00000157445	CACNA2D3	94.30373069	11.60263073	3.024897275	2.90E-15	3.61E-14
ENSG00000187091	PLCD1	199.2554202	440.5078738	-1.146992927	2.96E-15	3.69E-14
ENSG00000111913	RIPOR2	44.14461811	175.3214107	-1.989048719	3.11E-15	3.87E-14
ENSG00000087008	ACOX3	251.3343129	529.4410981	-1.077006551	4.31E-15	5.35E-14
ENSG00000196323	ZBTB44	885.5069952	525.0571509	0.754340116	4.53E-15	5.62E-14
ENSG00000090674	MCOLN1	433.5690902	760.9833464	-0.812951202	4.90E-15	6.07E-14
ENSG00000043143	JADE2	726.226733	405.5867399	0.840236684	4.93E-15	6.10E-14
ENSG00000171840	NINJ2	30.42884019	136.1774731	-2.166687091	5.10E-15	6.31E-14
ENSG00000103064	SLC7A6	363.2309104	671.1258573	-0.885563491	5.66E-15	6.98E-14
ENSG00000130748	TMEM160	77.91896736	234.0930007	-1.58632614	6.10E-15	7.51E-14
ENSG00000253522	MIR3142HG	198.9735192	0.713154737	8.14690613	6.18E-15	7.61E-14
ENSG00000123496	IL13RA2	88.62875598	5.839214286	3.904330862	6.21E-15	7.64E-14
ENSG00000182916	TCEAL7	172.5045047	42.27023177	2.031928562	6.33E-15	7.78E-14
ENSG00000213222	AC093724.1	239.2161151	74.72172401	1.680819851	6.53E-15	8.01E-14
ENSG00000185745	IFIT1	36.045847	195.9913096	-2.442495426	7.16E-15	8.77E-14
ENSG00000166816	LDHD	5.314014439	78.48335847	-3.886567548	7.70E-15	9.43E-14
ENSG00000166398	KIAA0355	626.9232293	1088.748259	-0.796258812	8.06E-15	9.86E-14
ENSG00000164659	KIAA1324L	19.15562292	115.8314388	-2.590169415	8.19E-15	1.00E-13
ENSG00000090539	CHRD	204.6116175	421.198186	-1.042559045	8.62E-15	1.05E-13
ENSG00000168890	TMEM150A	202.1307359	429.7263882	-1.088958084	8.69E-15	1.06E-13
ENSG00000101000	PROCR	246.6249991	87.36118525	1.497205357	9.29E-15	1.13E-13
ENSG00000082126	MPP4	110.6114128	15.72765165	2.812842775	9.61E-15	1.17E-13

ENSG00000166949	SMAD3	179.6210835	391.7034456	-1.125460512	1.01E-14	1.23E-13
ENSG00000103034	NDRG4	347.3371248	153.5542361	1.177194435	1.06E-14	1.29E-13
ENSG00000196932	TMEM26	92.96947175	10.49019118	3.14587482	1.09E-14	1.32E-13
ENSG00000125968	ID1	401.9159423	186.8958128	1.105012807	1.26E-14	1.52E-13
ENSG00000088387	DOCK9	253.9685604	509.0244592	-1.002466576	1.29E-14	1.55E-13
ENSG00000196507	TCEAL3	458.25989	775.6533264	-0.759091967	1.29E-14	1.56E-13
ENSG00000097096	SYDE2	152.788318	358.2460278	-1.230991387	1.39E-14	1.68E-13
ENSG00000132334	PTPRE	558.2596627	282.7019457	0.980242014	1.43E-14	1.73E-13
ENSG00000181649	PHLDA2	98.77043743	266.065385	-1.43109101	1.50E-14	1.80E-13
ENSG00000198355	PIM3	504.9156956	252.6182903	0.9997135	1.54E-14	1.85E-13
ENSG00000176641	RNF152	260.6989667	96.717907	1.430680256	1.84E-14	2.21E-13
ENSG00000106605	BLVRA	255.9130433	507.8082643	-0.987514006	1.91E-14	2.28E-13
ENSG00000250303	AP002884.1	35.47289679	150.7826292	-2.086071799	2.03E-14	2.42E-13
ENSG00000159348	CYB5R1	446.9007922	789.5120737	-0.820297009	2.30E-14	2.74E-13
ENSG00000164379	FOXQ1	287.0234106	77.21029674	1.893084773	2.44E-14	2.91E-13
ENSG00000112297	CRYBG1	125.7825315	317.7482052	-1.334605496	2.45E-14	2.92E-13
ENSG00000132205	EMILIN2	474.5206163	242.9691754	0.966134006	2.62E-14	3.11E-13
ENSG00000139668	WDFY2	397.1623972	183.1861504	1.117783884	3.23E-14	3.83E-13
ENSG00000172156	CCL11	79.79018274	3.862319586	4.371299453	3.28E-14	3.88E-13
ENSG00000186918	ZNF395	369.3172657	163.695614	1.175166641	3.48E-14	4.12E-13
ENSG00000111110	PPM1H	198.5626681	63.85236793	1.63791896	3.67E-14	4.33E-13
ENSG00000109016	DHRS7B	154.2668227	350.291827	-1.183930151	3.98E-14	4.70E-13
ENSG00000113070	HBEGF	130.1968356	336.7714803	-1.368435693	4.01E-14	4.73E-13
ENSG00000198113	TOR4A	63.40246429	205.7464918	-1.694570917	4.25E-14	5.01E-13
ENSG00000151151	IPMK	395.0653609	187.4262781	1.077039161	4.49E-14	5.28E-13
ENSG00000137965	IFI44	451.1728046	222.2087748	1.020576418	4.62E-14	5.43E-13
ENSG00000120913	PDLIM2	494.7578883	857.7238902	-0.794205026	5.22E-14	6.13E-13
ENSG00000169607	CKAP2L	180.5900066	50.95751058	1.827745384	5.37E-14	6.30E-13
ENSG00000167114	SLC27A4	447.3713966	779.4796653	-0.799338755	5.42E-14	6.36E-13
ENSG00000150787	PTS	420.6570166	724.3609161	-0.785233407	5.79E-14	6.79E-13
ENSG00000108342	CSF3	108.6604819	0	9.134074386	6.41E-14	7.51E-13
ENSG00000152527	PLEKHH2	266.9964334	500.7760453	-0.908335849	6.43E-14	7.53E-13
ENSG00000149809	TM7SF2	35.46679788	143.7872184	-2.017478773	7.52E-14	8.80E-13
ENSG00000143537	ADAM15	542.686422	931.4399357	-0.77816816	7.76E-14	9.07E-13
ENSG00000162878	PKDCC	64.05626743	208.1228842	-1.70352483	7.80E-14	9.11E-13
ENSG00000104213	PDGFRL	737.768213	401.4694259	0.876906946	8.05E-14	9.38E-13
ENSG00000113494	PRLR	110.6464957	21.08642538	2.393992136	8.13E-14	9.47E-13
ENSG00000117399	CDC20	241.8202413	79.85564208	1.600963531	8.62E-14	1.00E-12
ENSG00000147155	EBP	255.2559119	505.8608727	-0.985243645	8.89E-14	1.03E-12
ENSG00000139946	PELI2	422.6816963	198.6495552	1.089874839	9.13E-14	1.06E-12
ENSG00000184678	HIST2H2BE	461.9984588	226.790775	1.027744058	9.53E-14	1.10E-12
ENSG00000162302	RPS6KA4	453.8278227	783.5049934	-0.787053061	9.74E-14	1.13E-12
ENSG00000143479	DYRK3	612.9074802	347.3707606	0.819010301	1.10E-13	1.27E-12
ENSG00000155816	FMN2	302.1993724	558.7919739	-0.886536942	1.15E-13	1.33E-12
ENSG00000144230	GPR17	24.2515485	121.4930263	-2.33118658	1.22E-13	1.41E-12
ENSG00000183044	ABAT	131.7769115	301.1728319	-1.193067443	1.43E-13	1.65E-12
ENSG00000105552	BCAT2	151.2345257	340.4389928	-1.172430572	1.50E-13	1.72E-12
ENSG00000087258	GNAO1	69.35349575	3.486967011	4.307281418	1.60E-13	1.84E-12
ENSG00000024526	DEPDC1	169.8961875	50.0248581	1.763407499	1.70E-13	1.95E-12
ENSG00000119699	TGFB3	106.3288536	273.0263606	-1.362929303	1.83E-13	2.09E-12
ENSG00000154721	JAM2	429.5440982	181.5620329	1.243372746	1.86E-13	2.13E-12
ENSG00000122644	ARL4A	267.6785224	510.1254618	-0.931199168	1.96E-13	2.23E-12
ENSG00000167641	PPP1R14A	19.99950797	116.6807302	-2.556549125	1.99E-13	2.27E-12
ENSG00000227039	ITGB2-AS1	5.832395276	69.10014247	-3.551567036	2.22E-13	2.53E-12
ENSG00000004776	HSPB6	53.10820532	190.9710592	-1.84824146	2.46E-13	2.80E-12
ENSG00000143333	RGS16	0.304691634	152.8076385	-8.831388771	2.62E-13	2.98E-12
ENSG00000117595	IRF6	4.078556103	65.29725174	-4.007376507	2.71E-13	3.08E-12
ENSG00000127955	GNAI1	530.1263007	292.4251692	0.857992798	2.87E-13	3.25E-12
ENSG00000073146	MOV10L1	154.2420538	38.38067583	2.008142948	3.22E-13	3.63E-12
ENSG00000175063	UBE2C	148.6673201	38.83513036	1.936742086	3.30E-13	3.72E-12
ENSG00000261553	AL137782.1	19.36114075	104.7750969	-2.436680766	3.31E-13	3.73E-12
ENSG00000119946	CNNM1	115.8879931	22.14564255	2.388918884	3.41E-13	3.85E-12
ENSG00000166483	WEE1	678.4776341	370.9954881	0.872050226	3.42E-13	3.85E-12
ENSG00000100911	PSME2	592.2201576	325.4427749	0.865126749	3.48E-13	3.92E-12
ENSG00000198947	DMD	18.00434584	103.999398	-2.528181442	3.53E-13	3.97E-12
ENSG00000162383	SLC1A7	13.48171953	87.23120999	-2.698584914	3.95E-13	4.43E-12

ENSG00000153976	HS3ST3A1	191.9974805	398.2561341	-1.05164533	4.38E-13	4.91E-12
ENSG00000162522	KIAA1522	428.6087053	733.4868951	-0.776058465	4.78E-13	5.35E-12
ENSG00000076382	SPAG5	241.3665552	95.78906684	1.334283138	4.94E-13	5.53E-12
ENSG00000182154	MRPL41	388.4248898	684.5102353	-0.818127064	5.35E-13	5.99E-12
ENSG00000272168	CASC15	220.6895466	80.81252208	1.451258793	5.41E-13	6.04E-12
ENSG00000175040	CHST2	487.5027812	238.3361131	1.032484005	5.58E-13	6.22E-12
ENSG00000175866	BAIAP2	92.22375937	237.5076627	-1.364319919	5.65E-13	6.30E-12
ENSG00000185269	NOTUM	77.00659172	1.38009705	5.780418339	6.19E-13	6.89E-12
ENSG00000174697	LEP	5.216190836	69.6412622	-3.724330249	6.21E-13	6.91E-12
ENSG00000138411	HECW2	330.5469813	147.4069115	1.16637506	6.57E-13	7.31E-12
ENSG00000006607	FARP2	493.7926626	841.5823192	-0.768129572	6.60E-13	7.33E-12
ENSG00000112367	FIG4	267.896866	491.2158995	-0.875653899	7.34E-13	8.14E-12
ENSG00000175899	A2M	2.417069557	63.80314667	-4.688023094	7.37E-13	8.17E-12
ENSG00000165175	MID1IP1	393.5073576	671.3452276	-0.770055989	8.04E-13	8.90E-12
ENSG00000126353	CCR7	100.3461378	18.58192266	2.432326481	8.47E-13	9.37E-12
ENSG00000119640	ACYP1	170.5458699	43.82255263	1.9563063	9.02E-13	9.96E-12
ENSG00000125657	TNFSF9	104.9608378	263.9783397	-1.33447249	9.11E-13	1.01E-11
ENSG00000197467	COL13A1	655.2319554	326.0063427	1.008874988	9.27E-13	1.02E-11
ENSG00000154027	AK5	335.6256815	580.5265543	-0.790250457	9.28E-13	1.02E-11
ENSG00000135245	HILPDA	166.6912227	53.33494041	1.644097785	1.02E-12	1.13E-11
ENSG00000183578	TNFAIP8L3	9.187052708	78.13843988	-3.073657587	1.11E-12	1.22E-11
ENSG00000224189	HAGLR	105.1795546	252.5204011	-1.264516763	1.11E-12	1.22E-11
ENSG00000184232	OAF	371.243801	172.7350102	1.103908559	1.15E-12	1.26E-11
ENSG00000140950	TLDC1	439.6456933	744.230995	-0.760059599	1.21E-12	1.33E-11
ENSG00000116729	WLS	373.7153456	177.7617865	1.072623024	1.24E-12	1.36E-11
ENSG00000166289	PLEKHF1	115.9099011	273.832826	-1.241746093	1.26E-12	1.38E-11
ENSG00000003137	CYP26B1	93.31974481	13.88638539	2.741244206	1.37E-12	1.49E-11
ENSG00000001561	ENPP4	20.90191866	110.6005865	-2.394850717	1.37E-12	1.50E-11
ENSG00000001167	NFYA	591.803003	329.6905031	0.843411098	1.43E-12	1.56E-11
ENSG00000138160	KIF11	366.4908839	160.7816189	1.191201667	1.49E-12	1.62E-11
ENSG00000085433	WDR47	330.902914	572.8003082	-0.79132536	1.52E-12	1.65E-11
ENSG00000065054	SLC9A3R2	292.7207368	528.3699841	-0.853418321	1.53E-12	1.66E-11
ENSG00000182261	NLRP10	2.521714333	60.1013428	-4.588089748	1.54E-12	1.68E-11
ENSG00000165644	COMTD1	40.0085375	143.7636879	-1.846286046	1.57E-12	1.70E-11
ENSG00000127418	FGFRL1	262.9065863	500.2451332	-0.927067056	1.58E-12	1.71E-11
ENSG00000183597	TANGO2	199.0031311	410.6529074	-1.047727821	1.58E-12	1.72E-11
ENSG00000152642	GPD1L	71.63118673	206.8063687	-1.531095073	1.63E-12	1.76E-11
ENSG00000206073	SERPINB4	81.64696743	0	8.722402235	1.66E-12	1.80E-11
ENSG00000124491	F13A1	0.602562096	113.5472824	-7.510770402	1.76E-12	1.91E-11
ENSG00000234975	FTH1P2	153.6714267	38.37531793	2.005051897	1.77E-12	1.91E-11
ENSG00000110218	PANX1	654.1206284	377.8041018	0.793014633	1.79E-12	1.94E-11
ENSG00000196196	HRCT1	3.689683209	61.25834273	-4.039412421	1.82E-12	1.96E-11
ENSG00000158560	DYNC1I1	8.198039239	74.77803756	-3.199498638	1.89E-12	2.04E-11
ENSG00000203805	PLPP4	96.20835791	273.8168137	-1.509743209	1.92E-12	2.07E-11
ENSG00000169994	MYO7B	12.17195057	83.55443712	-2.78070384	2.01E-12	2.16E-11
ENSG00000134376	CRB1	77.6401159	0	8.647860656	2.02E-12	2.18E-11
ENSG00000253368	TRNP1	107.3032477	264.7778192	-1.302973999	2.14E-12	2.30E-11
ENSG00000152422	XRCC4	64.11218701	237.9549767	-1.894268542	2.20E-12	2.36E-11
ENSG00000151632	AKR1C2	317.9052849	139.6097786	1.187922462	2.20E-12	2.36E-11
ENSG00000171130	ATP6V0E2	60.28070371	182.3328128	-1.597328172	2.37E-12	2.54E-11
ENSG00000091656	ZFHX4	347.0875361	615.1617759	-0.825135974	2.49E-12	2.67E-11
ENSG00000125170	DOK4	281.999284	520.8612191	-0.88638404	2.50E-12	2.68E-11
ENSG00000136720	HS6ST1	556.5159069	306.5976799	0.859000848	2.51E-12	2.68E-11
ENSG00000163251	FZD5	9.527549462	78.35577736	-3.055418153	2.52E-12	2.69E-11
ENSG00000167244	IGF2	75.43440835	0	8.606362103	2.58E-12	2.76E-11
ENSG00000127528	KLF2	282.6606305	547.9929062	-0.956899879	2.75E-12	2.93E-11
ENSG00000141576	RNF157	227.6697963	72.44050722	1.649138657	2.96E-12	3.14E-11
ENSG00000138772	ANXA3	1.844841605	58.03943496	-4.959523199	3.00E-12	3.19E-11
ENSG00000167969	ECI1	195.3948586	384.7050953	-0.977646493	3.25E-12	3.45E-11
ENSG00000158792	SPATA2L	55.11449394	172.1897829	-1.642889002	3.82E-12	4.04E-11
ENSG00000126903	SLC10A3	321.1372797	567.2664906	-0.820712229	3.91E-12	4.14E-11
ENSG00000173334	TRIB1	261.7445277	97.05845998	1.430305371	4.11E-12	4.34E-11
ENSG00000172201	ID4	17.74668015	91.21854867	-2.36072743	4.12E-12	4.34E-11
ENSG00000185761	ADAMTSL5	90.21835342	229.0663518	-1.345730472	4.15E-12	4.38E-11
ENSG00000248587	GDNF-AS1	196.9656918	63.75509339	1.628768348	4.91E-12	5.16E-11
ENSG00000164611	PTTG1	214.7352811	79.83200511	1.426826755	5.05E-12	5.30E-11

ENSG00000168743	NPNT	1.881996921	59.21530449	-4.98411696	5.19E-12	5.44E-11
ENSG00000196639	HRH1	31.85815214	128.8868733	-2.01136633	5.47E-12	5.73E-11
ENSG00000132646	PCNA	754.2444663	444.5270484	0.762629412	5.51E-12	5.77E-11
ENSG00000139618	BRCA2	211.0082539	76.41363718	1.468384937	6.32E-12	6.60E-11
ENSG00000104522	TSTA3	305.285398	554.2822172	-0.861437947	6.43E-12	6.71E-11
ENSG00000280241	AC079298.3	70.18716111	8.422523683	3.059473103	6.76E-12	7.05E-11
ENSG00000176890	TYMS	196.9965837	64.26967379	1.615008343	6.90E-12	7.19E-11
ENSG00000167992	VWCE	148.9271285	42.69209204	1.800957828	7.00E-12	7.29E-11
ENSG00000105509	HAS1	129.4491451	35.82460278	1.854301321	7.44E-12	7.73E-11
ENSG00000243335	KCTD7	301.7683166	532.5744263	-0.820074488	8.01E-12	8.32E-11
ENSG00000105321	CCDC9	300.5089962	526.6874299	-0.809716183	8.18E-12	8.49E-11
ENSG00000188153	COL4A5	30.43701155	124.0868386	-2.025901846	8.28E-12	8.59E-11
ENSG00000170312	CDK1	242.7351989	93.58966556	1.37391376	8.36E-12	8.67E-11
ENSG00000120217	CD274	114.9759906	28.09625776	2.034085446	8.53E-12	8.83E-11
ENSG00000196843	ARID5A	200.3585057	386.5568644	-0.948050824	9.22E-12	9.53E-11
ENSG00000148143	ZNF462	270.4362491	114.8969455	1.235239483	1.01E-11	1.04E-10
ENSG00000275993	SIK1B	9.007469366	72.61639963	-3.007899272	1.01E-11	1.04E-10
ENSG00000164236	ANKRD33B	20.03424177	95.67766591	-2.258851925	1.02E-11	1.05E-10
ENSG00000078177	N4BP2	401.7715079	198.5071056	1.017465579	1.12E-11	1.15E-10
ENSG00000197019	SERTAD1	130.9241328	286.6682545	-1.131176302	1.12E-11	1.15E-10
ENSG00000101846	STS	421.3234569	722.6958002	-0.779131333	1.19E-11	1.22E-10
ENSG00000160145	KALRN	339.6568384	158.1750743	1.102324787	1.20E-11	1.23E-10
ENSG00000188158	NHS	557.0922515	319.4859291	0.801567906	1.26E-11	1.29E-10
ENSG00000132465	JCHAIN	2.470916673	54.46265019	-4.451515302	1.31E-11	1.34E-10
ENSG00000166448	TMEM130	1.540149971	55.26892679	-5.151165771	1.31E-11	1.34E-10
ENSG00000165572	KBTBD6	327.4225203	159.9070385	1.033863637	1.36E-11	1.39E-10
ENSG00000154175	ABI3BP	708.5334848	402.2957479	0.8175627	1.36E-11	1.39E-10
ENSG00000131069	ACSS2	343.8105217	597.4138581	-0.797860462	1.46E-11	1.49E-10
ENSG00000136783	NIPSNAP3A	280.4365037	500.6831364	-0.836611852	1.51E-11	1.54E-10
ENSG00000101849	TBL1X	680.5984423	399.165535	0.770215329	1.52E-11	1.55E-10
ENSG00000205795	CYS1	68.85155197	190.5073521	-1.467786812	1.74E-11	1.77E-10
ENSG00000228914	OR1H1P	96.10333994	16.94613423	2.506992889	1.79E-11	1.81E-10
ENSG00000070808	CAMK2A	192.9159592	57.54783176	1.744476289	1.79E-11	1.82E-10
ENSG00000180921	FAM83H	109.1937651	252.304314	-1.210250613	1.81E-11	1.84E-10
ENSG00000224594	RPL29P19	112.3026999	25.48200616	2.136712929	1.86E-11	1.88E-10
ENSG00000167549	CORO6	67.83767534	204.8323071	-1.598602586	1.97E-11	1.99E-10
ENSG00000205221	VIT	234.0224603	93.5515246	1.323794131	2.10E-11	2.12E-10
ENSG00000119986	AVPI1	97.29123461	236.8237981	-1.280308063	2.16E-11	2.18E-10
ENSG00000284968	AC093827.4	86.3316728	14.62347237	2.555494907	2.18E-11	2.20E-10
ENSG00000180660	MAB21L1	93.98602465	226.0638957	-1.26506589	2.30E-11	2.32E-10
ENSG00000257337	AC068888.1	28.40029446	125.6003214	-2.139149393	2.39E-11	2.40E-10
ENSG00000284691	AC073111.5	227.5089772	90.83648141	1.323100775	2.56E-11	2.57E-10
ENSG00000076356	PLXNA2	110.3617339	252.4030517	-1.194590138	2.59E-11	2.61E-10
ENSG00000144730	IL17RD	34.23061728	121.588247	-1.827772937	2.65E-11	2.66E-10
ENSG00000184988	TMEM106A	141.8571322	310.24993	-1.125937962	2.71E-11	2.72E-10
ENSG00000137198	GMPR	47.34707122	160.129287	-1.760963526	2.73E-11	2.75E-10
ENSG00000176155	CCDC57	735.0185592	434.7496957	0.758800311	2.86E-11	2.87E-10
ENSG00000185742	C11orf87	1.60763943	52.93730765	-5.079029006	3.06E-11	3.06E-10
ENSG00000115840	SLC25A12	527.38857	306.9192196	0.780865784	3.07E-11	3.07E-10
ENSG00000145362	ANK2	197.5635052	377.3289127	-0.934760462	3.38E-11	3.37E-10
ENSG00000170537	TMC7	8.749803675	68.22031063	-2.965393791	3.47E-11	3.47E-10
ENSG00000145990	GFOD1	20.81648153	102.5147212	-2.307082803	3.47E-11	3.47E-10
ENSG00000124570	SERPINB6	335.0778491	573.688544	-0.775906118	3.52E-11	3.52E-10
ENSG00000203721	LINC00862	16.66289253	87.09217097	-2.392611997	3.56E-11	3.55E-10
ENSG00000198597	ZNF536	16.21335135	88.9667285	-2.457329485	3.76E-11	3.75E-10
ENSG00000148019	CEP78	345.0069327	629.8988754	-0.866838897	3.95E-11	3.92E-10
ENSG00000067221	STOML1	235.2571964	428.1252106	-0.864553781	3.99E-11	3.96E-10
ENSG00000196155	PLEKHG4	202.5346013	79.415101	1.351979042	4.36E-11	4.31E-10
ENSG00000198417	MT1F	93.50659271	0.678908432	7.05991151	4.61E-11	4.55E-10
ENSG00000144891	AGTR1	97.72417832	237.975887	-1.282998864	4.63E-11	4.57E-10
ENSG00000164104	HMGB2	487.9901098	272.6999713	0.840033049	5.06E-11	4.98E-10
ENSG00000122678	POLM	302.5718324	560.4968827	-0.88894066	5.07E-11	4.99E-10
ENSG00000235770	LINC00607	36.3263034	128.5764962	-1.822077708	5.17E-11	5.09E-10
ENSG00000131386	GALNT15	335.2287315	38.93526218	3.104059429	5.21E-11	5.12E-10
ENSG00000100077	GRK3	72.98225593	7.341026329	3.311705072	5.30E-11	5.21E-10
ENSG00000136158	SPRY2	634.8780295	364.2625658	0.801439702	5.45E-11	5.35E-10

ENSG00000105499	PLA2G4C	328.5197657	160.7460158	1.030688973	5.59E-11	5.48E-10
ENSG00000174792	ODAPH	56.54569381	0	8.189127684	5.63E-11	5.51E-10
ENSG00000166971	AKTIP	179.724029	357.3868241	-0.991034101	5.68E-11	5.56E-10
ENSG00000132821	VSTM2L	13.51205368	81.74477282	-2.601542633	5.79E-11	5.66E-10
ENSG00000189362	NEMP2	196.8939872	77.17495167	1.351624464	5.81E-11	5.68E-10
ENSG00000185652	NTF3	120.2429791	261.1195142	-1.118521094	5.89E-11	5.76E-10
ENSG00000136114	THSD1	48.12365135	154.73557	-1.679239978	6.07E-11	5.92E-10
ENSG00000102468	HTR2A	70.43190671	193.5228809	-1.458107084	6.46E-11	6.29E-10
ENSG00000265107	GJA5	0.609383268	82.41230568	-7.04879156	6.52E-11	6.34E-10
ENSG00000140832	MARVELD3	3.093942285	51.73574735	-4.056289647	6.55E-11	6.37E-10
ENSG00000166908	PIP4K2C	293.3227612	501.3852108	-0.7731803	6.67E-11	6.48E-10
ENSG00000144642	RBMS3	295.5875321	514.0920659	-0.798272326	7.05E-11	6.84E-10
ENSG00000143473	KCNH1	106.7189823	240.6224748	-1.173818581	7.86E-11	7.60E-10
ENSG00000006283	CACNA1G	59.06184284	4.162571336	3.816626944	8.15E-11	7.88E-10
ENSG00000198108	CHSY3	189.0894092	373.2700353	-0.981179139	8.29E-11	8.01E-10
ENSG00000137675	MMP27	80.07837123	12.90887727	2.629264347	8.66E-11	8.35E-10
ENSG00000182253	SYNM	97.77793111	232.3361329	-1.246768994	9.31E-11	8.97E-10
ENSG00000261573	AL157402.2	0.335025778	84.61266199	-7.977619607	1.02E-10	9.80E-10
ENSG00000133597	ADCK2	83.5328304	215.6862042	-1.36491128	1.05E-10	1.01E-09
ENSG00000169594	BNC1	359.5193893	178.0155305	1.013745717	1.06E-10	1.01E-09
ENSG00000259354	AC025580.2	59.38052588	2.398459699	4.603648865	1.07E-10	1.02E-09
ENSG00000108239	TBC1D12	305.2643307	523.2815363	-0.776945287	1.10E-10	1.06E-09
ENSG00000169122	FAM110B	312.9285291	144.5003732	1.115507722	1.15E-10	1.10E-09
ENSG00000166228	PCBD1	99.61192509	251.8505564	-1.335188723	1.17E-10	1.12E-09
ENSG00000177822	AC098864.1	55.16359234	158.5100191	-1.519509372	1.19E-10	1.13E-09
ENSG00000184497	TMEM255B	41.29102178	130.7684858	-1.665253316	1.20E-10	1.14E-09
ENSG00000013810	TACC3	366.3911765	187.2961964	0.968157867	1.23E-10	1.18E-09
ENSG00000197586	ENTPD6	378.6762964	642.9329769	-0.764936413	1.24E-10	1.18E-09
ENSG00000100003	SEC14L2	80.09578529	200.4787488	-1.323149694	1.30E-10	1.24E-09
ENSG00000153714	LURAP1L	182.9009869	66.47898739	1.463086774	1.35E-10	1.28E-09
ENSG00000171877	FRMD5	39.79692076	144.1694745	-1.855541688	1.59E-10	1.51E-09
ENSG00000172954	LCLAT1	110.163292	255.1254002	-1.212817088	1.61E-10	1.52E-09
ENSG00000164066	INTU	193.1501078	381.4354044	-0.98023532	1.62E-10	1.53E-09
ENSG00000122877	EGR2	170.0435529	329.7202401	-0.955637521	1.62E-10	1.53E-09
ENSG00000099992	TBC1D10A	291.20419	502.6641145	-0.787249259	1.64E-10	1.55E-09
ENSG00000173166	RAPH1	146.6812162	302.5760577	-1.044837167	1.66E-10	1.56E-09
ENSG00000062582	MRPS24	115.9265929	28.46705592	2.026827192	1.66E-10	1.56E-09
ENSG00000130751	NPAS1	7.765189857	63.75293312	-3.034521851	1.68E-10	1.58E-09
ENSG00000114166	KAT2B	268.4899307	474.130431	-0.820230045	1.69E-10	1.60E-09
ENSG00000108950	FAM20A	179.5308976	346.0887415	-0.947921234	1.71E-10	1.61E-09
ENSG00000154262	ABCA6	244.8487424	103.2086046	1.248677358	1.76E-10	1.66E-09
ENSG00000285967	NIPBL-DT	276.5782032	128.3583553	1.106206219	1.79E-10	1.68E-09
ENSG00000141391	PRELID3A	81.00518167	223.9553737	-1.473011167	1.86E-10	1.75E-09
ENSG00000175356	SCUBE2	41.44027098	129.0852347	-1.638838517	1.89E-10	1.77E-09
ENSG00000161381	PLXDC1	31.99250313	114.7998597	-1.847568521	1.93E-10	1.81E-09
ENSG00000163412	EIF4E3	109.7778418	260.5339165	-1.24677543	2.01E-10	1.88E-09
ENSG00000168334	XIRP1	1.249100681	52.31892683	-5.391359146	2.20E-10	2.06E-09
ENSG00000187608	ISG15	347.7182036	641.9834231	-0.886773846	2.32E-10	2.16E-09
ENSG00000108688	CCL7	52.82998178	1.719551266	4.918684922	2.35E-10	2.19E-09
ENSG00000126785	RHOJ	193.7967166	385.1030684	-0.992326373	2.44E-10	2.27E-09
ENSG00000124216	SNAI1	70.04787686	184.3471515	-1.399957362	2.60E-10	2.41E-09
ENSG00000157570	TSPAN18	55.02358584	155.27184	-1.497734157	2.65E-10	2.46E-09
ENSG00000184304	PRKD1	355.6014919	608.678051	-0.776312448	2.93E-10	2.72E-09
ENSG00000013293	SLC7A14	52.48895142	2.51151268	4.410312495	2.95E-10	2.74E-09
ENSG00000099377	HSD3B7	150.6110567	298.4162372	-0.985644505	3.00E-10	2.78E-09
ENSG00000244649	LINC02086	1.272613652	50.63953307	-5.33893476	3.09E-10	2.86E-09
ENSG00000100979	PLTP	534.764953	313.0923786	0.771746902	3.41E-10	3.15E-09
ENSG00000228716	DHFR	342.7613977	171.8615977	0.997550389	3.57E-10	3.30E-09
ENSG00000081177	EXD2	247.3057379	441.7778203	-0.838710558	3.64E-10	3.35E-09
ENSG00000132563	REEP2	87.60770911	211.9170515	-1.272967906	3.65E-10	3.36E-09
ENSG00000242265	PEG10	187.5103103	69.88333541	1.422705987	3.76E-10	3.46E-09
ENSG00000107738	VSIR	218.5305861	398.9884164	-0.868653754	3.78E-10	3.47E-09
ENSG00000112299	VNN1	49.91876662	1.731517386	4.834340227	3.78E-10	3.48E-09
ENSG00000170044	ZPLD1	2.917408399	47.97947079	-4.080529014	3.81E-10	3.50E-09
ENSG00000203666	EFCAB2	141.3067882	46.28219971	1.609541436	3.85E-10	3.53E-09
ENSG00000142661	MYOM3	5.351169755	53.31926844	-3.32558326	3.89E-10	3.57E-09

ENSG00000172766	NAA16	332.2202264	166.6953807	0.995392955	4.70E-10	4.30E-09
ENSG00000079841	RIMS1	5.290501468	52.00270784	-3.2951061	5.08E-10	4.63E-09
ENSG00000134508	CABLES1	45.99197557	142.8804455	-1.641181832	5.14E-10	4.69E-09
ENSG00000111615	KRR1	507.4169464	297.3513668	0.771868468	5.45E-10	4.97E-09
ENSG00000279207	AC015813.6	157.287337	52.49812335	1.585366256	5.47E-10	4.99E-09
ENSG00000128965	CHAC1	178.0952981	61.07429651	1.541636208	5.92E-10	5.38E-09
ENSG00000087301	TXNDC16	134.2784412	281.854224	-1.067102369	5.92E-10	5.38E-09
ENSG00000169692	AGPAT2	315.172327	537.5831365	-0.769807055	5.93E-10	5.39E-09
ENSG00000159640	ACE	64.33493025	174.0223871	-1.433989229	5.97E-10	5.42E-09
ENSG00000179163	FUCA1	143.8974365	290.2789903	-1.014414454	6.17E-10	5.60E-09
ENSG00000132819	RBM38	43.90741594	137.6502903	-1.648856985	6.18E-10	5.61E-09
ENSG00000154556	SORBS2	365.7629346	619.4790177	-0.759421443	6.66E-10	6.04E-09
ENSG00000186310	NAP1L3	85.68029118	202.7938474	-1.24073837	6.79E-10	6.15E-09
ENSG00000174325	DIRC1	53.23016983	3.439102533	3.93306079	6.84E-10	6.20E-09
ENSG00000153814	JAZF1	216.9093043	396.1041341	-0.869624118	7.34E-10	6.64E-09
ENSG00000131697	NPHP4	639.1965424	364.4947718	0.808470297	7.35E-10	6.64E-09
ENSG00000125885	MCM8	207.9731863	85.97077413	1.276256694	7.81E-10	7.05E-09
ENSG00000139364	TMEM132B	123.0592315	32.0772835	1.938114391	7.89E-10	7.12E-09
ENSG00000008517	IL32	517.5242755	284.5931753	0.860886283	8.11E-10	7.31E-09
ENSG00000188483	IER5L	272.3721173	469.812513	-0.785715557	8.22E-10	7.40E-09
ENSG00000140525	FANCI	310.7932902	159.8785518	0.959795126	8.27E-10	7.44E-09
ENSG00000170113	NIPA1	232.2679891	418.1734255	-0.84705672	8.31E-10	7.47E-09
ENSG00000164400	CSF2	42.16174813	0	7.768093036	9.75E-10	8.72E-09
ENSG00000095739	BAMBI	22.63906603	101.8733632	-2.158707603	1.06E-09	9.49E-09
ENSG00000102996	MMP15	170.2156872	325.4958908	-0.935711315	1.07E-09	9.52E-09
ENSG00000146918	NCAPG2	410.311328	229.4639022	0.839377814	1.08E-09	9.60E-09
ENSG00000115919	KYNU	41.4774263	0	7.743363753	1.08E-09	9.64E-09
ENSG00000164920	OSR2	97.53947307	219.921929	-1.173790265	1.09E-09	9.75E-09
ENSG00000120156	TEK	44.26057804	135.6455687	-1.615091015	1.13E-09	1.00E-08
ENSG00000023445	BIRC3	80.8189617	13.44925489	2.593491377	1.13E-09	1.01E-08
ENSG00000237649	KIFC1	175.9685797	61.63284673	1.514227441	1.17E-09	1.04E-08
ENSG00000144152	FBLN7	269.0770568	123.0925146	1.129130195	1.28E-09	1.13E-08
ENSG00000113368	LMNB1	233.2168921	102.404677	1.190049428	1.32E-09	1.17E-08
ENSG00000157193	LRP8	217.9100722	393.1810302	-0.849848147	1.35E-09	1.20E-08
ENSG00000188906	LRRK2	95.8199243	223.6603733	-1.219115264	1.41E-09	1.25E-08
ENSG00000136689	IL1RN	40.36025508	0	7.703363119	1.45E-09	1.28E-08
ENSG00000106100	NOD1	30.27484227	121.5526439	-2.00659969	1.49E-09	1.31E-08
ENSG00000170745	KCNS3	134.0293903	275.2958824	-1.038696142	1.59E-09	1.40E-08
ENSG00000083123	BCKDHB	164.3919969	321.7138784	-0.967156703	1.65E-09	1.45E-08
ENSG00000228526	MIR34AHG	396.8807552	222.9909304	0.83122111	1.65E-09	1.45E-08
ENSG00000082269	FAM135A	266.9647491	128.4760691	1.054929062	1.67E-09	1.46E-08
ENSG00000105088	OLFM2	77.83308685	202.4558323	-1.377439452	1.74E-09	1.52E-08
ENSG00000050730	TNIP3	218.9313077	6.528436784	5.059667641	1.79E-09	1.57E-08
ENSG00000160469	BRSK1	116.1896352	35.81594077	1.698167696	1.80E-09	1.57E-08
ENSG00000138778	CENPE	365.0354488	185.1355135	0.981665084	1.82E-09	1.59E-08
ENSG00000250479	CHCHD10	111.903862	266.5105046	-1.249806409	1.83E-09	1.60E-08
ENSG00000136160	EDNRB	39.49015667	0	7.671598171	1.84E-09	1.61E-08
ENSG00000136167	LCP1	54.67724491	6.348247973	3.111211798	1.94E-09	1.69E-08
ENSG00000160796	NBEAL2	80.54029889	202.024568	-1.330743844	1.94E-09	1.69E-08
ENSG00000133460	SLC2A11	113.9453278	250.2924309	-1.135624887	1.97E-09	1.71E-08
ENSG00000215114	UBXN2B	109.6734517	244.6678856	-1.15788554	2.00E-09	1.74E-08
ENSG00000143816	WNT9A	38.32586518	119.1369667	-1.636213687	2.01E-09	1.75E-08
ENSG00000164930	FZD6	280.3781908	506.29235	-0.853379733	2.10E-09	1.82E-08
ENSG00000165626	BEND7	218.6394419	392.5934027	-0.843360358	2.21E-09	1.91E-08
ENSG00000138134	STAMBPL1	186.2357185	78.37320786	1.249764465	2.21E-09	1.91E-08
ENSG00000104823	ECH1	391.705048	223.1348191	0.81149412	2.21E-09	1.91E-08
ENSG00000111247	RAD51AP1	93.65612078	23.47952717	1.996794832	2.28E-09	1.97E-08
ENSG00000161010	MRNIP	620.8357262	363.3407356	0.771781643	2.40E-09	2.07E-08
ENSG00000185947	ZNF267	187.4662395	65.81098357	1.507933508	2.40E-09	2.07E-08
ENSG00000162814	SPATA17	15.42438474	74.03239504	-2.26955665	2.53E-09	2.18E-08
ENSG00000163814	CDCP1	71.46040269	13.60676178	2.389810967	2.55E-09	2.19E-08
ENSG00000121152	NCAPH	126.5459127	41.26783636	1.616284997	2.57E-09	2.21E-08
ENSG00000180332	KCTD4	97.3444538	25.56286664	1.92878372	2.60E-09	2.23E-08
ENSG00000157557	ETS2	202.9701511	82.72022715	1.297463568	2.71E-09	2.33E-08
ENSG00000198855	FICD	121.0076162	255.0060342	-1.073264026	2.88E-09	2.47E-08
ENSG00000145819	ARHGAP26	29.47653864	114.6713477	-1.951259708	2.91E-09	2.49E-08

ENSG00000272825	AL844908.1	0.63289624	58.10392644	-6.539933117	3.02E-09	2.59E-08
ENSG00000238042	LINC02257	28.81877924	105.7965056	-1.873793276	3.04E-09	2.61E-08
ENSG00000072571	HMMR	114.8886656	35.71485391	1.685752393	3.08E-09	2.64E-08
ENSG00000254510	AP001107.5	3.940527728	51.92966081	-3.694888089	3.08E-09	2.64E-08
ENSG00000075218	GTSE1	189.1531269	76.83579273	1.297400871	3.10E-09	2.66E-08
ENSG00000171132	PRKCE	271.1364703	132.0830991	1.039372591	3.18E-09	2.72E-08
ENSG00000253161	LINC01605	5.896113019	52.88078116	-3.161558954	3.24E-09	2.77E-08
ENSG00000172164	SNTB1	233.0830788	403.0642161	-0.789848286	3.34E-09	2.85E-08
ENSG00000116711	PLA2G4A	82.11813778	17.63700878	2.222773166	3.45E-09	2.94E-08
ENSG00000110987	BCL7A	274.7157589	475.6587	-0.790593535	3.56E-09	3.04E-08
ENSG00000153064	BANK1	11.13276737	63.10496689	-2.496996348	3.61E-09	3.08E-08
ENSG00000109805	NCAPG	202.0981648	77.84218926	1.375878118	3.74E-09	3.18E-08
ENSG00000262246	CORO7	26.93615438	105.0270373	-1.965362457	3.92E-09	3.34E-08
ENSG00000164136	IL15	169.6576352	70.41174691	1.268599324	4.00E-09	3.40E-08
ENSG00000137877	SPTBN5	23.30471794	88.43482408	-1.921682366	4.01E-09	3.40E-08
ENSG00000132196	HSD17B7	100.4295025	232.1343158	-1.209365509	4.56E-09	3.87E-08
ENSG00000179859	RNF227	32.19999909	106.8810118	-1.727053911	4.57E-09	3.87E-08
ENSG00000172830	SSH3	148.3188407	299.5328908	-1.012579591	4.57E-09	3.87E-08
ENSG00000235531	MSC-AS1	462.4011586	254.5307628	0.863236924	4.72E-09	3.99E-08
ENSG00000136630	HLX	60.96367534	156.5548513	-1.362469146	4.83E-09	4.09E-08
ENSG00000160352	ZNF714	53.3538336	148.6305391	-1.479240235	5.31E-09	4.47E-08
ENSG00000131080	EDA2R	446.2726205	239.103195	0.900509087	5.59E-09	4.70E-08
ENSG00000137767	SQOR	456.7723271	256.247707	0.835580793	5.71E-09	4.80E-08
ENSG00000168078	PBK	141.276105	51.72378123	1.448209604	5.73E-09	4.82E-08
ENSG00000235649	MXRA5Y	47.36744041	135.5677543	-1.517779902	5.74E-09	4.82E-08
ENSG00000143416	SELENBP1	335.1536986	186.6665092	0.844370198	6.05E-09	5.08E-08
ENSG00000254554	AC080023.1	80.67024611	16.28454982	2.315774584	6.13E-09	5.14E-08
ENSG00000213047	DENND1B	236.3749012	411.0425379	-0.797779764	6.21E-09	5.20E-08
ENSG00000099260	PALMD	3.807970329	46.85301132	-3.637678549	6.35E-09	5.31E-08
ENSG00000163249	CCNYL1	259.3237808	438.3814372	-0.755830819	6.89E-09	5.75E-08
ENSG00000137959	IFI44L	192.687462	74.17162286	1.373850637	6.93E-09	5.78E-08
ENSG00000112561	TFEB	53.10066188	159.8230417	-1.589772355	7.13E-09	5.95E-08
ENSG00000167394	ZNF668	170.6935844	324.7656009	-0.930887631	7.77E-09	6.47E-08
ENSG00000134769	DTNA	87.4843944	20.58440165	2.083814332	7.81E-09	6.50E-08
ENSG00000138182	KIF20B	280.5794895	140.2522805	1.001753827	7.95E-09	6.60E-08
ENSG00000235865	GSN-AS1	131.1570297	264.8038053	-1.015009487	8.14E-09	6.75E-08
ENSG00000178734	LMO7DN	1.942665208	39.38707236	-4.382835514	8.25E-09	6.83E-08
ENSG00000213066	FGFR1OP	318.0456646	171.8153853	0.889553708	8.30E-09	6.87E-08
ENSG00000239697	TNFSF12	101.8194262	216.8261753	-1.090261889	8.58E-09	7.09E-08
ENSG00000064687	ABCA7	23.89058823	90.798931	-1.924349299	8.75E-09	7.23E-08
ENSG00000160460	SPTBN4	26.34651237	98.73370889	-1.909040943	8.78E-09	7.24E-08
ENSG00000197905	TEAD4	87.83673992	197.4489872	-1.168619728	9.40E-09	7.74E-08
ENSG00000108448	TRIM16L	194.0016306	77.27739531	1.33071845	9.71E-09	7.99E-08
ENSG00000275074	NUDT18	106.916702	230.7635856	-1.109458316	9.84E-09	8.09E-08
ENSG00000219507	FTH1P8	117.4398975	32.12195035	1.874923495	1.01E-08	8.29E-08
ENSG00000117245	KIF17	7.046762162	55.70586849	-3.002493893	1.04E-08	8.51E-08
ENSG00000179242	CDH4	27.86387163	100.3955898	-1.850550468	1.05E-08	8.60E-08
ENSG00000074047	GLI2	106.4361987	227.4173469	-1.096506573	1.10E-08	9.03E-08
ENSG00000023171	GRAMD1B	221.2901305	378.0515329	-0.772508075	1.13E-08	9.26E-08
ENSG00000143476	DTL	145.5930248	49.01185336	1.571673231	1.13E-08	9.27E-08
ENSG00000163017	ACTG2	98.05857617	26.5589491	1.882043774	1.13E-08	9.27E-08
ENSG00000108947	EFNB3	42.53330129	3.509247197	3.599461583	1.17E-08	9.52E-08
ENSG00000184083	FAM120C	259.6044919	121.0940818	1.099936828	1.17E-08	9.55E-08
ENSG00000112742	TTK	113.8850328	35.37745349	1.687175874	1.18E-08	9.62E-08
ENSG00000178773	CPNE7	67.38086959	188.1466558	-1.47719306	1.19E-08	9.70E-08
ENSG00000233251	AC007743.1	32.25016882	103.2365379	-1.680792386	1.24E-08	1.01E-07
ENSG00000087157	PGS1	482.4827794	283.0282527	0.769831652	1.28E-08	1.04E-07
ENSG00000092470	WDR76	138.178391	52.93730765	1.384524804	1.30E-08	1.06E-07
ENSG00000174028	FAM3C2	213.7857501	93.27344656	1.196361142	1.33E-08	1.08E-07
ENSG00000178038	ALS2CL	16.25050667	73.77409657	-2.184940159	1.38E-08	1.12E-07
ENSG00000162944	RFTN2	283.1151952	145.4942954	0.961148463	1.40E-08	1.13E-07
ENSG00000275180	AC048341.2	157.8565155	56.01222024	1.493823237	1.42E-08	1.15E-07
ENSG00000120833	SOCS2	78.99216209	19.03442985	2.057288977	1.43E-08	1.15E-07
ENSG00000136367	ZFHX2	33.43680763	117.7381888	-1.808802004	1.46E-08	1.17E-07
ENSG00000229271	AC091493.1	32.84223235	0	7.404496445	1.52E-08	1.22E-07
ENSG00000138190	EXOC6	8.933158734	57.4393332	-2.676011217	1.57E-08	1.26E-07

ENSG00000101255	TRIB3	170.6426924	314.6870624	-0.885031305	1.69E-08	1.36E-07
ENSG00000268894	PLCE1-AS1	0	29.1166742	-7.401204759	1.76E-08	1.41E-07
ENSG00000174891	RSRC1	399.9912949	236.9780009	0.754847955	1.81E-08	1.44E-07
ENSG00000198774	RASSF9	17.89287989	76.96877682	-2.097593191	1.81E-08	1.45E-07
ENSG00000276600	RAB7B	155.5096358	62.13612077	1.324701453	1.82E-08	1.46E-07
ENSG00000153162	BMP6	1.570484114	36.84792159	-4.560886442	1.88E-08	1.50E-07
ENSG00000179104	TMTC2	201.1665856	352.2240176	-0.807863982	1.94E-08	1.55E-07
ENSG00000116396	KCNC4	160.5096216	305.5620997	-0.926517755	1.97E-08	1.57E-07
ENSG00000092969	TGFB2	129.2436273	47.60907438	1.440101696	1.97E-08	1.57E-07
ENSG00000223458	LMO7DN-IT1	0	29.55545676	-7.42443943	1.97E-08	1.57E-07
ENSG00000149089	APIP	316.2089	175.2403615	0.852076533	2.07E-08	1.65E-07
ENSG00000269190	FBXO17	228.0709613	389.7384347	-0.772994458	2.08E-08	1.65E-07
ENSG00000187720	THSD4	118.3813514	238.4649896	-1.008804158	2.11E-08	1.68E-07
ENSG00000145365	TIFA	168.1124576	71.62857743	1.23089035	2.11E-08	1.68E-07
ENSG00000167565	SERTAD3	188.243546	335.6238394	-0.834809964	2.13E-08	1.69E-07
ENSG00000166851	PLK1	188.2656386	74.62258447	1.335780537	2.13E-08	1.69E-07
ENSG00000119969	HELLS	149.3871924	53.00785405	1.497024967	2.15E-08	1.71E-07
ENSG00000127311	HELB	166.4243143	68.90788107	1.270892799	2.46E-08	1.94E-07
ENSG00000111335	OAS2	157.0362377	297.9337801	-0.924423874	2.54E-08	2.00E-07
ENSG00000198324	PHETA1	222.0995606	376.3640677	-0.76078312	2.60E-08	2.05E-07
ENSG00000184922	FMNL1	31.7119524	105.1882952	-1.728520469	2.69E-08	2.12E-07
ENSG00000142178	SIK1	3.482815179	44.20576373	-3.681166016	3.09E-08	2.42E-07
ENSG00000183935	HTR7P1	209.043775	95.68147823	1.129558989	3.17E-08	2.48E-07
ENSG00000128709	HOXD9	22.40051366	82.35878802	-1.877361193	3.17E-08	2.48E-07
ENSG00000242258	LINC00996	39.05048611	1.415995409	4.796327384	3.23E-08	2.53E-07
ENSG00000126787	DLGAP5	162.310232	63.49268733	1.356180732	3.45E-08	2.70E-07
ENSG00000166592	RRAD	260.5305099	23.83164452	3.447424028	3.58E-08	2.80E-07
ENSG00000141682	PMAIP1	144.4070139	51.43178975	1.489535517	3.60E-08	2.81E-07
ENSG00000011258	MBTD1	311.0116379	172.4134333	0.851003222	3.63E-08	2.83E-07
ENSG00000177791	MYOZ1	0.602562096	45.6308229	-6.199119737	3.70E-08	2.88E-07
ENSG00000179362	HMGN2P46	45.43464583	5.208170332	3.116441145	3.84E-08	2.98E-07
ENSG00000166166	TRMT61A	175.313312	319.0451992	-0.86638353	4.00E-08	3.11E-07
ENSG00000171813	PWWP2B	163.2147853	309.6328609	-0.921658143	4.16E-08	3.23E-07
ENSG00000196782	MAML3	166.52861	301.8400695	-0.856578077	4.20E-08	3.26E-07
ENSG00000149548	CCDC15	62.58621299	12.34672766	2.347561228	4.25E-08	3.29E-07
ENSG00000105559	PLEKHA4	160.9400536	298.2605712	-0.892377929	4.32E-08	3.35E-07
ENSG00000135838	NPL	76.86865767	18.61616896	2.045548539	4.32E-08	3.35E-07
ENSG00000185008	ROBO2	18.76539983	73.80268969	-1.977118632	4.68E-08	3.62E-07
ENSG00000187123	LYPD6	256.4521424	132.566442	0.952489935	4.75E-08	3.66E-07
ENSG00000029534	ANK1	67.38364018	13.9206317	2.268759187	4.85E-08	3.75E-07
ENSG00000119471	HSDL2	484.6260534	275.7558254	0.814336144	5.01E-08	3.86E-07
ENSG00000101997	CCDC22	132.8643765	254.9258708	-0.939699737	5.01E-08	3.86E-07
ENSG00000147862	NFIB	221.458493	111.8561727	0.985236583	5.29E-08	4.08E-07
ENSG00000135976	ANKRD36	442.2341707	259.432959	0.770262195	5.33E-08	4.10E-07
ENSG00000235314	LINC00957	17.75350132	72.63857334	-2.033117786	5.50E-08	4.23E-07
ENSG00000131620	ANO1	0.304691634	42.80789583	-6.996787755	5.74E-08	4.41E-07
ENSG00000169427	KCNK9	4.068685476	40.41244497	-3.31513427	6.47E-08	4.96E-07
ENSG00000143772	ITPKB	303.6092019	152.6255847	0.989465199	6.56E-08	5.03E-07
ENSG00000164663	USP49	320.2438989	165.6088658	0.949914731	6.62E-08	5.06E-07
ENSG00000005102	MEOX1	70.30607616	15.28010062	2.19595018	6.68E-08	5.11E-07
ENSG00000118193	KIF14	190.2404315	79.41139515	1.261580362	6.72E-08	5.13E-07
ENSG00000132436	FIGNL1	168.9639763	74.45065593	1.184138626	6.98E-08	5.33E-07
ENSG00000125414	MYH2	0	24.9095425	-7.176041762	6.98E-08	5.33E-07
ENSG00000148335	NTMT1	166.3606909	302.4865966	-0.86251945	7.24E-08	5.52E-07
ENSG00000183828	NUDT14	36.35591528	113.5007296	-1.640401344	7.58E-08	5.76E-07
ENSG00000186187	ZNRF1	385.4513071	224.856871	0.776770359	7.69E-08	5.84E-07
ENSG00000267279	AC090409.1	49.79375266	7.68708876	2.691521733	7.91E-08	6.00E-07
ENSG00000267669	AC098847.1	37.04580242	2.137812159	4.131052362	8.21E-08	6.23E-07
ENSG00000140455	USP3	312.33209	169.3990483	0.883279879	9.05E-08	6.84E-07
ENSG00000156970	BUB1B	154.5436919	56.04716356	1.461292847	9.18E-08	6.94E-07
ENSG00000066468	FGFR2	8.38821547	57.59654481	-2.776759203	9.73E-08	7.34E-07
ENSG00000065615	CYB5R4	430.6793355	255.6646549	0.75248035	9.86E-08	7.43E-07
ENSG00000224596	ZMIZ1-AS1	97.62302639	30.47614312	1.677325176	1.00E-07	7.52E-07
ENSG00000171533	MAP6	58.03441819	147.0274513	-1.346434169	1.00E-07	7.52E-07
ENSG00000065060	UHRF1BP1	151.641276	313.659423	-1.046126598	1.04E-07	7.79E-07
ENSG00000115841	RMDN2	130.6750819	267.0543961	-1.033512568	1.04E-07	7.81E-07

ENSG00000279159	AC003681.1	185.9006927	87.57437005	1.085297669	1.05E-07	7.89E-07
ENSG00000137812	KNL1	222.6216189	101.8380553	1.126634458	1.09E-07	8.16E-07
ENSG00000273270	AC090114.2	126.1204222	241.0617656	-0.936611824	1.13E-07	8.46E-07
ENSG00000152503	TRIM36	40.45260771	3.505943089	3.528713276	1.13E-07	8.46E-07
ENSG00000138744	NAAA	121.5738395	240.6334036	-0.983382536	1.15E-07	8.58E-07
ENSG00000282057	AC092807.3	110.5229262	231.9671997	-1.068480363	1.16E-07	8.67E-07
ENSG00000169432	SCN9A	12.72676446	59.5701354	-2.224050541	1.31E-07	9.75E-07
ENSG00000241697	TMEFF1	12.39855985	60.03325194	-2.272907092	1.34E-07	9.96E-07
ENSG00000110723	EXPH5	27.48046971	90.72148111	-1.730716701	1.41E-07	1.05E-06
ENSG00000091844	RGS17	50.35915944	7.304726227	2.778324524	1.41E-07	1.05E-06
ENSG00000118322	ATP10B	24.87825151	0	7.006440861	1.42E-07	1.06E-06
ENSG00000078967	UBE2D4	99.3205026	204.6286943	-1.042988149	1.57E-07	1.16E-06
ENSG00000237493	AC034102.1	127.8004843	50.16845153	1.349974613	1.57E-07	1.16E-06
ENSG00000091651	ORC6	131.0566902	49.87550502	1.391310102	1.59E-07	1.18E-06
ENSG00000112562	SMOC2	0	23.1404747	-7.069880942	1.60E-07	1.18E-06
ENSG00000138347	MYPN	42.48313157	3.912237862	3.45451334	1.60E-07	1.18E-06
ENSG00000120658	ENOX1	152.7526974	61.76572436	1.308114315	1.61E-07	1.19E-06
ENSG00000255299	AP003557.1	38.82136098	2.398459699	3.995571297	1.62E-07	1.19E-06
ENSG00000254726	MEX3A	80.76879198	23.09386053	1.805789688	1.66E-07	1.22E-06
ENSG00000198513	ATL1	15.09375862	67.52829224	-2.153294338	1.66E-07	1.22E-06
ENSG00000157353	FUK	130.8937987	244.3443163	-0.900040096	1.66E-07	1.22E-06
ENSG00000154134	ROBO3	180.6899729	77.98629091	1.212464806	1.71E-07	1.25E-06
ENSG00000118407	FILIP1	62.97373569	12.074114	2.391352146	1.72E-07	1.26E-06
ENSG00000186847	KRT14	0	22.44424224	-7.025779942	1.78E-07	1.31E-06
ENSG00000154065	ANKRD29	6.458470344	43.76903498	-2.750052656	1.85E-07	1.36E-06
ENSG00000285410	GABPB1-IT1	275.7824154	152.8749259	0.850950972	1.88E-07	1.37E-06
ENSG00000104998	IL27RA	22.57641961	84.36446465	-1.906326743	1.91E-07	1.40E-06
ENSG00000125618	PAX8	321.8766845	178.1862088	0.851731806	1.97E-07	1.44E-06
ENSG00000150977	RILPL2	413.9553354	241.6967283	0.777171685	2.02E-07	1.47E-06
ENSG00000178425	NT5DC1	325.0718689	184.0294692	0.820164569	2.02E-07	1.47E-06
ENSG00000272398	CD24	0	22.55233906	-7.031761698	2.03E-07	1.48E-06
ENSG00000184985	SORCS2	49.62089616	127.5049112	-1.363138667	2.09E-07	1.52E-06
ENSG00000272695	GAS6-DT	193.9419393	336.4294562	-0.796162249	2.11E-07	1.53E-06
ENSG00000131196	NFATC1	186.2861671	331.6624416	-0.831301677	2.18E-07	1.58E-06
ENSG00000198556	ZNF789	157.4950216	279.5298036	-0.829096429	2.23E-07	1.62E-06
ENSG00000064655	EYA2	21.14531407	74.42672369	-1.813566286	2.29E-07	1.65E-06
ENSG00000108001	EBF3	258.3962522	133.2875167	0.956644879	2.33E-07	1.69E-06
ENSG00000099998	GGT5	5.99770834	44.84587144	-2.914027175	2.36E-07	1.70E-06
ENSG00000128052	KDR	36.25136484	3.156174807	3.521476794	2.36E-07	1.71E-06
ENSG00000167703	SLC43A2	125.1917279	242.0436393	-0.951225732	2.38E-07	1.72E-06
ENSG00000198771	RCSD1	23.31458856	0	6.912869967	2.47E-07	1.78E-06
ENSG00000115756	HPCAL1	313.350904	184.7328181	0.762445461	2.55E-07	1.84E-06
ENSG00000166670	MMP10	34.53225946	1.446937607	4.612909589	2.67E-07	1.92E-06
ENSG00000160325	CACFD1	134.6257591	256.1239832	-0.926277137	2.74E-07	1.97E-06
ENSG00000154319	FAM167A	89.77151263	26.14564437	1.77791357	2.77E-07	1.99E-06
ENSG00000118785	SPP1	6.813959639	44.6637353	-2.706707457	2.78E-07	1.99E-06
ENSG00000174576	NPAS4	3.087121113	34.72207555	-3.482236034	2.80E-07	2.01E-06
ENSG00000221890	NPTXR	84.71488501	21.57142036	1.968558333	2.85E-07	2.04E-06
ENSG00000226012	AP001434.1	55.95435254	10.62387229	2.403508602	2.90E-07	2.08E-06
ENSG00000081059	TCF7	32.98223886	96.24157403	-1.543415567	2.90E-07	2.08E-06
ENSG00000226287	TMEM191A	2.789250651	34.25469987	-3.61331124	2.92E-07	2.09E-06
ENSG00000184156	KCNQ3	89.15100286	24.90914075	1.839879169	2.93E-07	2.10E-06
ENSG00000084628	NKAIN1	22.95982153	0	6.891135107	2.98E-07	2.13E-06
ENSG00000097046	CDC7	138.8439485	57.47688361	1.272475685	3.04E-07	2.17E-06
ENSG00000162490	DRAXIN	58.36567225	10.85027353	2.42385929	3.09E-07	2.21E-06
ENSG00000267095	AC025048.1	10.65773509	60.52866745	-2.527777505	3.21E-07	2.29E-06
ENSG00000050344	NFE2L3	95.19259336	31.67069346	1.590317881	3.41E-07	2.43E-06
ENSG00000107872	FBXL15	87.33065123	185.184772	-1.086689655	3.43E-07	2.45E-06
ENSG00000233521	LINC01638	0.609383268	35.41996006	-5.83130278	3.50E-07	2.50E-06
ENSG00000124356	STAMBP	312.0404128	182.7179713	0.772483741	3.54E-07	2.52E-06
ENSG00000183111	ARHGEF37	6.502446832	42.96150806	-2.717584839	3.59E-07	2.55E-06
ENSG00000161277	THAP8	91.07867553	195.5224125	-1.103957316	3.61E-07	2.57E-06
ENSG00000184160	ADRA2C	0	21.54263843	-6.965308476	3.67E-07	2.61E-06
ENSG00000138028	CGREF1	77.02400578	165.2385758	-1.10317071	3.75E-07	2.66E-06
ENSG00000084710	EFR3B	14.16909082	61.54868215	-2.111918255	3.77E-07	2.67E-06
ENSG00000146409	SLC18B1	206.9236691	103.3288563	1.000210115	3.77E-07	2.67E-06

ENSG00000168405	CMAHP	50.39254304	124.3435914	-1.304666235	3.80E-07	2.69E-06
ENSG00000162891	IL20	22.46935332	0	6.861371321	4.17E-07	2.94E-06
ENSG00000216775	AL109918.1	56.47308244	142.2737355	-1.338548947	4.24E-07	2.99E-06
ENSG00000158163	DZIP1L	188.4249229	325.5485599	-0.790347106	4.28E-07	3.02E-06
ENSG00000151725	CENPU	117.3541113	42.31218505	1.471872645	4.38E-07	3.09E-06
ENSG00000142910	TINAGL1	6.332734118	43.83462522	-2.803054077	4.58E-07	3.22E-06
ENSG00000067191	CACNB1	142.5518624	253.3874634	-0.830312012	4.93E-07	3.45E-06
ENSG00000136490	LIMD2	264.3019569	141.0282746	0.906919349	5.02E-07	3.51E-06
ENSG00000163701	IL17RE	19.76123447	70.87456818	-1.836517933	5.18E-07	3.62E-06
ENSG00000177694	NAALADL2	160.0898809	286.2639763	-0.836842624	5.21E-07	3.63E-06
ENSG00000176435	CLEC14A	11.94901867	56.76868504	-2.236882737	5.23E-07	3.64E-06
ENSG00000235790	AC114488.2	90.41535082	28.47902204	1.668237785	5.24E-07	3.65E-06
ENSG00000227825	SLC9A7P1	12.49638346	61.1574688	-2.290792085	5.25E-07	3.66E-06
ENSG00000126522	ASL	192.2281445	339.1010829	-0.820307675	5.29E-07	3.68E-06
ENSG00000168763	CNNM3	198.9015358	337.5277935	-0.764691714	5.63E-07	3.91E-06
ENSG00000213888	LINC01521	79.45974527	24.27849854	1.71380631	5.66E-07	3.93E-06
ENSG00000101384	JAG1	83.70801409	178.9589812	-1.094615401	6.08E-07	4.22E-06
ENSG00000259426	AC027237.3	85.33907627	197.7247161	-1.216776662	6.19E-07	4.29E-06
ENSG00000188917	TRMT2B	125.0143775	245.5110397	-0.972660716	6.32E-07	4.37E-06
ENSG00000092853	CLSPN	143.8876561	58.99591321	1.28581378	6.35E-07	4.39E-06
ENSG00000116663	FBXO6	80.09264151	168.0202016	-1.066778148	6.64E-07	4.58E-06
ENSG00000147119	CHST7	57.80431606	142.7432715	-1.302119505	6.65E-07	4.59E-06
ENSG00000124466	LYPD3	44.05540927	6.380842224	2.79431751	6.71E-07	4.63E-06
ENSG00000179841	AKAP5	13.49159016	57.93133814	-2.107761069	6.76E-07	4.66E-06
ENSG00000157554	ERG	0.914074903	30.67831685	-5.040249982	6.79E-07	4.68E-06
ENSG00000046889	PREX2	2.856740111	31.87316245	-3.497252383	6.93E-07	4.77E-06
ENSG00000113722	CDX1	30.90387247	1.743483506	4.143404863	7.10E-07	4.88E-06
ENSG00000165030	NFIL3	284.3604298	158.6006855	0.84272024	7.25E-07	4.98E-06
ENSG00000205336	ADGRG1	60.77403272	13.35147214	2.188924122	7.58E-07	5.19E-06
ENSG00000139572	GPR84	32.05379935	0.339454216	6.410113858	7.88E-07	5.39E-06
ENSG00000211445	GPX3	74.18584127	20.05940072	1.890854931	7.99E-07	5.46E-06
ENSG00000038002	AGA	180.5020536	317.2818297	-0.812158664	8.01E-07	5.47E-06
ENSG00000259330	INAFM2	187.9053764	328.2653616	-0.803853996	8.01E-07	5.47E-06
ENSG00000164109	MAD2L1	176.6606054	83.09958085	1.089941978	8.08E-07	5.51E-06
ENSG00000149636	DSN1	263.9696316	147.2042296	0.841356093	8.22E-07	5.61E-06
ENSG00000120318	ARAP3	146.6756509	60.87763225	1.268288763	8.31E-07	5.67E-06
ENSG00000142731	PLK4	119.6683296	45.52789518	1.398200399	8.46E-07	5.76E-06
ENSG00000279821	AC145098.2	31.75422963	94.71292739	-1.570673893	9.14E-07	6.21E-06
ENSG00000151967	SCHIP1	56.93896636	130.6319023	-1.200211454	9.29E-07	6.30E-06
ENSG00000234997	AC016745.1	20.3699898	0	6.716642684	9.51E-07	6.45E-06
ENSG00000140600	SH3GL3	32.62234991	1.808672008	4.204599766	9.63E-07	6.52E-06
ENSG00000151789	ZNF385D	54.59208665	134.7167285	-1.300085554	9.89E-07	6.69E-06
ENSG00000135842	FAM129A	153.3277201	291.7103624	-0.925094475	9.90E-07	6.69E-06
ENSG00000204362	AL590644.1	11.08574143	52.11605609	-2.225265775	9.91E-07	6.70E-06
ENSG00000183513	COA5	314.0041736	182.8606097	0.778529555	1.02E-06	6.86E-06
ENSG00000132849	PATJ	120.9273953	226.0003592	-0.901831937	1.03E-06	6.94E-06
ENSG00000169738	DCXR	90.56567134	185.1755195	-1.033626437	1.04E-06	7.02E-06
ENSG00000139910	NOVA1	67.38706283	17.09207673	1.97676941	1.04E-06	7.03E-06
ENSG00000165202	OR1Q1	38.09117885	4.198469695	3.176261904	1.05E-06	7.07E-06
ENSG00000235173	HGH1	208.0286666	357.0425575	-0.778587405	1.10E-06	7.40E-06
ENSG00000170271	FAXDC2	262.4650981	148.2220017	0.825465423	1.10E-06	7.40E-06
ENSG00000184979	USP18	7.402879391	44.52549977	-2.580763374	1.14E-06	7.63E-06
ENSG00000148219	ASTN2	56.53842925	137.9380918	-1.281832258	1.14E-06	7.66E-06
ENSG00000042832	TG	37.42920434	97.94049717	-1.387740028	1.16E-06	7.77E-06
ENSG00000156876	SASS6	78.84838387	164.5086053	-1.062052288	1.16E-06	7.78E-06
ENSG00000116194	ANGPTL1	23.70786111	79.15981137	-1.731753029	1.19E-06	7.95E-06
ENSG00000100105	PATZ1	227.1485505	115.0094451	0.983241382	1.24E-06	8.32E-06
ENSG00000169621	APLF	144.8297379	62.60099583	1.213290315	1.30E-06	8.69E-06
ENSG00000267683	AC008991.2	0	17.91498034	-6.700257666	1.37E-06	9.11E-06
ENSG00000215790	SLC35E2A	225.3523439	123.2786146	0.870285138	1.39E-06	9.26E-06
ENSG00000239828	AC063944.1	3.652527893	33.28915786	-3.167175231	1.41E-06	9.35E-06
ENSG00000166897	ELFN2	13.13000196	55.75568029	-2.087984753	1.50E-06	9.96E-06
ENSG00000068831	RASGRP2	4.627271084	38.16848332	-3.037921701	1.55E-06	1.03E-05
ENSG00000050327	ARHGEF5	87.7004108	178.5361658	-1.027081223	1.64E-06	1.08E-05
ENSG00000141574	SECTM1	65.85838842	18.62813508	1.824295506	1.67E-06	1.10E-05
ENSG00000234459	AC002064.2	31.17150311	2.477266375	3.662683606	1.71E-06	1.13E-05

ENSG00000149554	CHEK1	288.8361245	169.963805	0.766737741	1.72E-06	1.14E-05
ENSG00000187398	LUZP2	0.602562096	29.52256723	-5.570614008	1.75E-06	1.16E-05
ENSG00000153233	PTPRR	0.304691634	27.12410754	-6.336365674	1.76E-06	1.16E-05
ENSG00000168389	MFSD2A	87.45028854	30.50833563	1.518810003	1.76E-06	1.16E-05
ENSG00000111981	ULBP1	61.87972838	143.210836	-1.207586733	1.79E-06	1.18E-05
ENSG00000080561	MID2	122.2455903	225.2967151	-0.882000145	1.87E-06	1.23E-05
ENSG00000235997	LINC01936	5.253346152	37.21541564	-2.817360335	1.93E-06	1.27E-05
ENSG00000141837	CACNA1A	138.6541455	54.84086004	1.340305354	2.01E-06	1.31E-05
ENSG00000111907	TPD52L1	7.8289076	41.76820804	-2.418743833	2.02E-06	1.32E-05
ENSG00000144218	AFF3	189.009443	325.1716617	-0.784321303	2.12E-06	1.38E-05
ENSG00000267248	AC025048.2	29.35932285	84.6512047	-1.529498672	2.17E-06	1.41E-05
ENSG00000130762	ARHGEF16	33.87522231	99.41081372	-1.556400176	2.20E-06	1.43E-05
ENSG00000169679	BUB1	160.4913248	74.26250212	1.112849033	2.21E-06	1.44E-05
ENSG00000123485	HJURP	144.7200656	62.76911205	1.20344801	2.22E-06	1.45E-05
ENSG00000176678	FOXL1	28.09120317	85.38674609	-1.60395743	2.25E-06	1.46E-05
ENSG00000124882	EREG	29.80644251	1.446937607	4.399278206	2.29E-06	1.49E-05
ENSG00000197410	DCHS2	35.8229151	4.57382227	2.974294179	2.31E-06	1.50E-05
ENSG00000105607	GCDH	126.5763412	232.5742663	-0.876155159	2.32E-06	1.51E-05
ENSG00000243449	C4orf48	130.0244225	228.3612685	-0.812522335	2.34E-06	1.52E-05
ENSG00000172548	NIPAL4	4.999452178	36.95836748	-2.889048187	2.35E-06	1.52E-05
ENSG00000012048	BRCA1	190.3963132	96.11695668	0.987712942	2.36E-06	1.53E-05
ENSG00000100297	MCM5	349.7364271	206.4111915	0.762578854	2.36E-06	1.53E-05
ENSG00000181619	GPR135	152.96683	72.13595907	1.086319311	2.54E-06	1.64E-05
ENSG00000154258	ABCA9	142.4649808	65.12436816	1.127414359	2.56E-06	1.66E-05
ENSG00000137070	IL11RA	209.5182737	359.0793441	-0.776768201	2.58E-06	1.67E-05
ENSG00000099957	P2RX6	48.42394334	9.350113536	2.36478657	2.65E-06	1.71E-05
ENSG00000187164	SHTN1	123.1139895	223.1386765	-0.856591294	2.78E-06	1.79E-05
ENSG00000128655	PDE11A	0.595740924	29.52656836	-5.57632976	2.85E-06	1.84E-05
ENSG00000119922	IFIT2	24.48937861	78.08551277	-1.671336016	2.94E-06	1.89E-05
ENSG00000060642	PIGV	350.4992747	207.2943725	0.759345772	2.97E-06	1.90E-05
ENSG00000126822	PLEKHG3	28.08123822	83.87656731	-1.574095466	2.98E-06	1.91E-05
ENSG00000130720	FIBCD1	10.28313247	46.77060527	-2.184301748	3.00E-06	1.93E-05
ENSG00000180346	TIGD2	143.7278181	65.57892916	1.13040469	3.02E-06	1.94E-05
ENSG00000160886	LY6K	17.83831052	0	6.528484158	3.07E-06	1.96E-05
ENSG00000158373	HIST1H2BD	71.58038907	19.67869024	1.863535179	3.08E-06	1.97E-05
ENSG00000170542	SERPINB9	11.61094344	61.69323063	-2.398051711	3.19E-06	2.04E-05
ENSG00000128849	CGNL1	222.0180598	385.8140629	-0.80023226	3.25E-06	2.08E-05
ENSG00000100092	SH3BP1	38.41004644	5.977851559	2.685607209	3.39E-06	2.16E-05
ENSG00000130962	PRRG1	279.0249749	155.9755293	0.84102148	3.39E-06	2.16E-05
ENSG00000159212	CLIC6	17.31688023	0	6.484780029	3.39E-06	2.16E-05
ENSG00000174938	SEZ6L2	145.4338147	69.76378068	1.058765485	3.41E-06	2.17E-05
ENSG00000148803	FUOM	82.29844338	165.9119748	-1.011991011	3.53E-06	2.25E-05
ENSG00000198937	CCDC167	71.53983524	151.2803937	-1.077109693	3.61E-06	2.30E-05
ENSG00000231445	TIMM8AP1	130.1326504	55.64222557	1.225621324	3.62E-06	2.30E-05
ENSG00000204442	FAM155A	215.9763708	119.7610007	0.849730026	3.88E-06	2.46E-05
ENSG00000284052	AC006460.2	76.75854192	23.44858497	1.709546429	3.89E-06	2.46E-05
ENSG00000162631	NTNG1	57.8592345	14.46225964	2.00217005	3.90E-06	2.47E-05
ENSG00000250602	AC093535.1	25.04733629	76.47295957	-1.612613566	3.98E-06	2.52E-05
ENSG00000230615	AL139220.2	36.47932432	5.216832344	2.80098957	4.07E-06	2.57E-05
ENSG00000089685	BIRC5	244.0605883	135.7023904	0.848447354	4.08E-06	2.58E-05
ENSG00000147408	CSGALNACT1	74.15990678	19.35696179	1.942057514	4.15E-06	2.61E-05
ENSG00000182325	FBXL6	137.0012737	235.9579862	-0.783834746	4.17E-06	2.63E-05
ENSG00000254887	AC010247.1	44.33039469	8.839132521	2.332061026	4.21E-06	2.65E-05
ENSG00000139173	TMEM117	153.6542013	262.7761888	-0.772671269	4.22E-06	2.65E-05
ENSG00000260196	AC124798.1	25.91680677	81.58345361	-1.660682946	4.25E-06	2.67E-05
ENSG00000080986	NDC80	141.4279602	59.21882153	1.260122857	4.37E-06	2.74E-05
ENSG00000146858	ZC3HAV1L	120.7482512	225.5952697	-0.898910938	4.39E-06	2.75E-05
ENSG00000188626	GOLGA8M	11.07829232	50.93181982	-2.211416562	4.40E-06	2.76E-05
ENSG00000100368	CSF2RB	5.730172021	36.65811573	-2.69445067	4.44E-06	2.78E-05
ENSG00000167034	NKX3-1	67.15900902	149.1373674	-1.150822056	4.64E-06	2.90E-05
ENSG00000153721	CNKSR3	114.1460026	48.22114225	1.241898771	4.65E-06	2.90E-05
ENSG00000118620	ZNF430	90.18389849	180.0827963	-0.994940004	4.74E-06	2.96E-05
ENSG00000082074	FYB1	13.2117617	54.45273786	-2.033708027	4.76E-06	2.97E-05
ENSG00000137033	IL33	27.24524567	1.054261007	4.695422772	4.79E-06	2.99E-05
ENSG00000115008	IL1A	32.92829742	2.737913915	3.56870333	4.84E-06	3.01E-05
ENSG00000241439	AC092903.1	28.58094913	0.678908432	5.353460839	4.95E-06	3.08E-05

ENSG00000105889	STEAP1B	116.7192369	52.21218679	1.16068888	5.01E-06	3.11E-05
ENSG00000262406	MMP12	38.10230535	5.640049397	2.759749455	5.06E-06	3.14E-05
ENSG00000128311	TST	167.3458383	286.0675994	-0.773406507	5.15E-06	3.19E-05
ENSG00000267339	LINC00906	0	16.72343884	-6.598892834	5.18E-06	3.20E-05
ENSG00000117152	RGS4	126.8739327	227.5527866	-0.844891548	5.21E-06	3.23E-05
ENSG00000205763	RP9P	139.234266	239.875582	-0.784388181	5.26E-06	3.25E-05
ENSG00000183718	TRIM52	207.9911381	114.9039554	0.85701949	5.30E-06	3.27E-05
ENSG00000145358	DDIT4L	4.748607659	33.76890141	-2.841764365	5.33E-06	3.29E-05
ENSG00000135525	MAP7	160.2086114	80.92421828	0.983778076	5.38E-06	3.32E-05
ENSG00000178026	LRRC75B	206.7590783	111.2667868	0.893497475	5.48E-06	3.38E-05
ENSG00000161912	ADCY10P1	98.0758959	36.54836686	1.420599949	5.54E-06	3.41E-05
ENSG00000171320	ESCO2	71.38744226	20.41412516	1.809921142	5.68E-06	3.50E-05
ENSG00000158220	ESYT3	21.85009942	72.82257448	-1.730225502	5.75E-06	3.54E-05
ENSG00000189057	FAM111B	65.63070781	17.94592254	1.875247359	5.96E-06	3.66E-05
ENSG00000119227	PIGZ	69.74381316	145.5183341	-1.061891757	6.09E-06	3.74E-05
ENSG00000072041	SLC6A15	69.43597775	21.33140095	1.701326644	6.11E-06	3.75E-05
ENSG00000244274	DBNDD2	3.638885549	32.4027178	-3.133367438	6.17E-06	3.78E-05
ENSG00000163507	CIP2A	180.1026822	90.9009729	0.98782298	6.17E-06	3.78E-05
ENSG00000222022	AC112721.1	0	15.05700349	-6.450187269	6.20E-06	3.79E-05
ENSG00000176928	GCNT4	18.07246324	65.58017947	-1.849851554	6.60E-06	4.03E-05
ENSG00000156313	RPGR	150.2463247	70.12305954	1.100301777	6.72E-06	4.10E-05
ENSG00000109272	PF4V1	15.81083612	0	6.353088626	6.76E-06	4.12E-05
ENSG00000085276	MECOM	13.31873366	59.88235327	-2.165674812	6.94E-06	4.23E-05
ENSG00000137745	MMP13	34.32853521	4.219097827	3.024840477	7.12E-06	4.34E-05
ENSG00000123572	NRK	58.57693993	16.23998945	1.853958827	7.13E-06	4.34E-05
ENSG00000227220	AL133346.1	1.637973574	25.46508388	-4.009623734	7.20E-06	4.38E-05
ENSG00000167178	ISLR2	0.335025778	22.28828094	-6.055334266	7.32E-06	4.45E-05
ENSG00000106565	TMEM176B	24.01255274	0.361734402	5.995449146	7.45E-06	4.52E-05
ENSG00000128228	SDF2L1	129.0101025	230.6761783	-0.836477054	7.71E-06	4.68E-05
ENSG00000158014	SLC30A2	0.304691634	21.78926605	-6.020816436	7.73E-06	4.68E-05
ENSG00000163517	HDAC11	137.8972123	240.0192819	-0.798333012	7.80E-06	4.73E-05
ENSG00000101888	NXT2	189.8902947	98.42424184	0.946309886	7.92E-06	4.79E-05
ENSG00000154240	CEP112	143.0892622	244.7310203	-0.774773193	7.97E-06	4.82E-05
ENSG00000167528	ZNF641	142.9127283	242.9664618	-0.766545187	8.00E-06	4.83E-05
ENSG00000103942	HOMER2	26.28414482	76.37928441	-1.534757442	8.18E-06	4.94E-05
ENSG00000224431	AC063976.2	75.05163436	25.44775986	1.557190154	8.25E-06	4.98E-05
ENSG00000188707	ZBED6CL	82.04517735	30.06984835	1.447305275	8.34E-06	5.03E-05
ENSG00000176533	GNG7	3.347836259	29.1711469	-3.102028732	8.35E-06	5.03E-05
ENSG00000101057	MYBL2	143.3230659	66.73126819	1.102375267	8.53E-06	5.13E-05
ENSG00000258754	LINC01579	0.602562096	24.73996303	-5.318520878	8.72E-06	5.24E-05
ENSG00000109944	JHY	137.6867612	234.8501011	-0.772124993	8.82E-06	5.29E-05
ENSG00000196867	ZFP28	121.2010305	213.5515897	-0.817431466	8.82E-06	5.29E-05
ENSG00000255717	SNHG1	254.0961144	144.8026787	0.813120645	9.06E-06	5.42E-05
ENSG00000255823	MTRNR2L8	30.46904496	3.102952424	3.279130052	9.07E-06	5.43E-05
ENSG00000185883	ATP6V0C	66.87657449	150.3767734	-1.172800732	9.14E-06	5.47E-05
ENSG00000054967	RELT	65.5500194	133.6883471	-1.028655015	9.43E-06	5.63E-05
ENSG00000141933	TPGS1	109.7311649	215.4713222	-0.974327856	9.45E-06	5.64E-05
ENSG00000129521	EGLN3	1.19830302	23.77482276	-4.268150681	9.54E-06	5.69E-05
ENSG00000273007	AC021205.3	30.25302856	3.101300371	3.268640712	9.61E-06	5.73E-05
ENSG00000165821	SALL2	93.7753849	37.46534737	1.323004521	9.65E-06	5.75E-05
ENSG00000130940	CASZ1	0.304691634	22.91271668	-6.095479468	9.75E-06	5.81E-05
ENSG00000112029	FBXO5	123.7887898	57.54743002	1.105374891	1.00E-05	5.97E-05
ENSG00000156049	GNA14	45.19071681	9.823248865	2.200676884	1.00E-05	5.97E-05
ENSG00000186417	GLDN	15.12714221	0	6.290073754	1.00E-05	5.98E-05
ENSG00000260077	AC104794.2	80.67508915	26.84147508	1.583834409	1.01E-05	5.99E-05
ENSG00000123353	ORMDL2	143.6293625	243.9548373	-0.76232121	1.02E-05	6.05E-05
ENSG00000119915	ELOVL3	16.23614206	0	6.39000937	1.03E-05	6.09E-05
ENSG00000115163	CENPA	61.82013141	19.233193	1.68234484	1.04E-05	6.18E-05
ENSG00000167130	DOLPP1	103.0363992	195.2389766	-0.919629341	1.04E-05	6.18E-05
ENSG00000130558	OLFM1	40.93248303	96.46632321	-1.237903197	1.06E-05	6.30E-05
ENSG00000065621	GSTO2	90.58971792	33.65254432	1.428765903	1.07E-05	6.32E-05
ENSG00000173436	MINOS1	141.127578	70.08921498	1.009633513	1.07E-05	6.35E-05
ENSG00000144354	CDCA7	41.28555081	8.772291965	2.236191747	1.08E-05	6.38E-05
ENSG00000261490	AC005674.2	36.58944007	90.06690666	-1.301467844	1.08E-05	6.38E-05
ENSG00000135617	PRADC1	120.1516035	217.2482936	-0.855778089	1.08E-05	6.41E-05
ENSG00000068489	PRR11	197.5539134	108.5093886	0.864183564	1.11E-05	6.58E-05

ENSG00000133135	RNF128	1.26579248	22.77007829	-4.185638165	1.13E-05	6.66E-05
ENSG00000154263	ABCA10	104.8247876	45.91015124	1.190591606	1.16E-05	6.82E-05
ENSG00000177570	SAMD12	114.7994567	46.8645757	1.291150323	1.16E-05	6.85E-05
ENSG00000101412	E2F1	150.0793124	64.39253262	1.219248159	1.17E-05	6.91E-05
ENSG00000168427	KLHL30	4.248268818	32.0926602	-2.894925242	1.20E-05	7.05E-05
ENSG00000095587	TLL2	8.293441321	42.65299604	-2.351651366	1.21E-05	7.08E-05
ENSG00000136999	NOV	124.729078	223.3474585	-0.838225527	1.24E-05	7.27E-05
ENSG00000180113	TDRD6	32.06681376	4.529261899	2.816507742	1.25E-05	7.35E-05
ENSG00000168329	CX3CR1	0	13.71651065	-6.314759919	1.26E-05	7.42E-05
ENSG00000227471	AKR1B15	14.9073541	0	6.267863993	1.28E-05	7.51E-05
ENSG00000102683	SGCG	46.08046215	105.4939048	-1.194515728	1.29E-05	7.58E-05
ENSG00000164167	LSM6	157.5073138	80.42314958	0.969249701	1.30E-05	7.59E-05
ENSG00000112379	ARFGEF3	0.335025778	20.86362352	-5.960298905	1.30E-05	7.61E-05
ENSG00000175463	TBC1D10C	15.61078926	53.84592142	-1.78805131	1.31E-05	7.68E-05
ENSG00000085999	RAD54L	49.35838743	12.29185323	2.009084638	1.31E-05	7.68E-05
ENSG00000135740	SLC9A5	131.8896333	59.51907328	1.150440818	1.35E-05	7.89E-05
ENSG00000116661	FBXO2	44.01143278	9.538669085	2.20934867	1.36E-05	7.92E-05
ENSG00000137975	CLCA2	60.7604847	18.95026527	1.681511591	1.38E-05	8.03E-05
ENSG00000173041	ZNF680	91.29657573	36.17106696	1.338416455	1.39E-05	8.09E-05
ENSG00000175315	CST6	1.191481848	23.41969657	-4.249487313	1.39E-05	8.11E-05
ENSG00000173227	SYT12	1.279434824	22.94666772	-4.19037793	1.47E-05	8.55E-05
ENSG00000125457	MIF4GD	53.56312315	119.1592469	-1.156002137	1.48E-05	8.60E-05
ENSG00000171115	GIMAP8	14.98157041	0	6.277556082	1.53E-05	8.90E-05
ENSG00000231560	CLEC12A-AS1	5.768677533	42.4986868	-2.86424595	1.54E-05	8.94E-05
ENSG00000152822	GRM1	23.66567821	1.39206317	4.0768771	1.58E-05	9.14E-05
ENSG00000168916	ZNF608	76.4196501	28.43941783	1.427782886	1.66E-05	9.60E-05
ENSG00000262966	AC005695.1	24.85716006	0.678908432	5.152624574	1.67E-05	9.65E-05
ENSG00000285895	AP003557.2	14.18955434	0	6.197907189	1.68E-05	9.69E-05
ENSG00000186684	CYP27C1	49.08959524	13.29990181	1.881966166	1.68E-05	9.72E-05
ENSG00000152784	PRDM8	197.113336	111.0968055	0.825916665	1.71E-05	9.84E-05
ENSG00000130224	LRCH2	144.1338221	69.92264434	1.044300472	1.71E-05	9.86E-05
ENSG00000267528	AC008991.1	0.297870462	19.56769105	-5.866859646	1.75E-05	0.000100728
ENSG00000146072	TNFRSF21	214.9893355	119.5885189	0.845159045	1.78E-05	0.000102418
ENSG00000163710	PCOLCE2	12.26663039	51.1987803	-2.051980848	1.80E-05	0.000103934
ENSG00000163808	KIF15	91.38767249	37.19934193	1.297918363	1.87E-05	0.000107761
ENSG00000259342	AC025580.1	13.96904397	0	6.17410805	1.87E-05	0.000107795
ENSG00000225855	RUSC1-AS1	126.8323778	222.9661947	-0.816774018	1.89E-05	0.0001085
ENSG00000061337	LZTS1	1.821328633	24.19102986	-3.705719945	1.91E-05	0.000109833
ENSG00000006128	TAC1	13.94930271	0	6.172387512	1.91E-05	0.000109887
ENSG00000214026	MRPL23	122.5003009	256.4675614	-1.06610284	1.98E-05	0.000113259
ENSG00000231123	SPATA20P1	53.69325903	15.43936602	1.799661063	1.98E-05	0.000113371
ENSG00000246430	LINC00968	97.1938544	41.50260434	1.228616251	1.99E-05	0.000113798
ENSG00000135540	NHSL1	64.04020357	131.5579466	-1.039489874	2.05E-05	0.000117462
ENSG00000160446	ZDHHC12	132.0823254	229.3697639	-0.796845534	2.06E-05	0.000117792
ENSG00000134072	CAMK1	22.9833345	66.44463462	-1.530216057	2.08E-05	0.000118857
ENSG00000123612	ACVR1C	30.04301675	4.254996185	2.828304196	2.08E-05	0.000118857
ENSG00000230928	AL139241.1	7.591705426	38.72578323	-2.361813825	2.08E-05	0.0001191
ENSG00000245571	FAM111A-DT	104.2741847	42.92715529	1.279856013	2.12E-05	0.00012072
ENSG00000165480	SKA3	72.45840412	23.2601359	1.643401629	2.13E-05	0.000121248
ENSG00000226445	BX322234.1	85.3341389	158.39902	-0.893844863	2.14E-05	0.000121941
ENSG00000090889	KIF4A	145.6658248	69.28073303	1.072594843	2.16E-05	0.000122871
ENSG00000122483	CCDC18	128.2595471	60.51504928	1.084155239	2.21E-05	0.000125768
ENSG00000151623	NR3C2	14.388251	53.03138455	-1.883675714	2.27E-05	0.000128722
ENSG00000171791	BCL2	173.689071	92.98130353	0.900946258	2.29E-05	0.000129602
ENSG00000198857	HSD3BP5	21.58668388	0.361734402	5.836450917	2.30E-05	0.000130211
ENSG00000225968	ELFN1	127.6413645	60.8133537	1.071223052	2.30E-05	0.000130211
ENSG00000283175	AC007920.2	79.35922127	29.27924372	1.441212737	2.31E-05	0.000130803
ENSG00000204388	HSPA1B	131.9793799	221.74606	-0.75033532	2.33E-05	0.000131634
ENSG00000134690	CDCA8	113.9848345	48.06363537	1.248104168	2.33E-05	0.000132019
ENSG00000106789	CORO2A	3.161431745	26.37009828	-3.071508941	2.33E-05	0.000132026
ENSG00000171241	SHCBP1	204.5722252	102.3519387	0.997649363	2.39E-05	0.000135233
ENSG00000167900	TK1	263.8854462	156.0141784	0.760029015	2.40E-05	0.000135778
ENSG00000160256	FAM207A	122.9116154	216.7594413	-0.817642023	2.45E-05	0.000138035
ENSG00000224259	LINC01133	4.406760709	31.04130156	-2.822639444	2.57E-05	0.000144545
ENSG00000273472	AC096733.2	57.22992132	17.48640539	1.709477267	2.65E-05	0.00014882
ENSG00000273179	AC092535.4	53.76901418	120.0753788	-1.157421049	2.67E-05	0.000149909

ENSG00000173376	NDNF	74.22183505	26.48675064	1.482085623	2.67E-05	0.000149992
ENSG00000167775	CD320	68.07908851	145.4597087	-1.092047877	2.73E-05	0.000153393
ENSG00000204991	SPIRE2	125.5290809	59.75568212	1.068785726	2.75E-05	0.000154548
ENSG00000159884	CCDC107	67.79935437	136.8498798	-1.009900831	2.78E-05	0.000155792
ENSG00000168781	PPIP5K1	68.9772882	138.5838978	-1.004898826	2.78E-05	0.000155883
ENSG00000146197	SCUBE3	77.47866888	147.598263	-0.930860804	2.79E-05	0.000156178
ENSG00000248927	AC114284.1	33.82128087	83.0360444	-1.293598651	2.80E-05	0.000156962
ENSG00000230183	CNOT6LP1	39.94032578	9.145992486	2.128659277	2.81E-05	0.000157206
ENSG00000006025	OSBPL7	143.6251515	72.62010548	0.983722331	2.92E-05	0.000163556
ENSG00000102349	KLF8	33.47512859	87.84287612	-1.398287085	2.97E-05	0.000166174
ENSG00000279400	AC008957.3	123.256233	58.68875797	1.069988273	3.04E-05	0.00016965
ENSG00000120162	MOB3B	3.496457523	27.32988064	-2.982419049	3.06E-05	0.000171085
ENSG00000197182	MIRLET7BHG	144.8894051	247.0095849	-0.769296968	3.13E-05	0.000174576
ENSG00000111341	MGP	0	12.22336062	-6.149903593	3.16E-05	0.000175833
ENSG00000266088	AC004585.1	0	12.55745693	-6.1890199	3.19E-05	0.000177751
ENSG00000179627	ZBTB42	21.99054932	63.64523805	-1.538291465	3.21E-05	0.000178772
ENSG00000069424	KCNAB2	203.285879	117.7428497	0.787102859	3.26E-05	0.000181406
ENSG00000198756	COLGALT2	14.31089092	49.70728233	-1.795645245	3.28E-05	0.000182355
ENSG00000125247	TMTC4	206.0575269	118.4891069	0.799909137	3.38E-05	0.000187795
ENSG00000117115	PADI2	1.218766537	22.38566196	-4.175955505	3.39E-05	0.000188086
ENSG00000236393	AC091806.1	44.78505779	11.88555846	1.912347972	3.47E-05	0.000192134
ENSG00000134201	GSTM5	90.4115791	35.68886787	1.339891177	3.48E-05	0.000192485
ENSG00000102575	ACP5	106.7319024	43.6666978	1.287498251	3.50E-05	0.000193739
ENSG00000134215	VAV3	37.69871879	6.274397459	2.585850925	3.51E-05	0.000193904
ENSG00000115107	STEAP3	196.0124132	109.0911289	0.847052764	3.59E-05	0.000198665
ENSG00000230715	AC018638.2	108.6803175	196.4224707	-0.85224963	3.59E-05	0.000198665
ENSG00000173715	C11orf80	49.20546084	107.672678	-1.128289555	3.61E-05	0.000199748
ENSG00000261371	PECAM1	23.70113426	1.709237201	3.768408206	3.73E-05	0.000205762
ENSG00000165507	DEPP1	38.27094674	101.6105554	-1.401650405	3.73E-05	0.000205762
ENSG00000141441	GAREM1	84.96491294	34.43253961	1.30456961	3.78E-05	0.000208374
ENSG00000157542	KCNJ6	10.91162906	47.29065004	-2.132269472	3.80E-05	0.000209407
ENSG00000114405	C3orf14	161.3563014	87.52940794	0.883011097	3.89E-05	0.000213981
ENSG00000138180	CEP55	183.7619369	98.07828587	0.908285864	3.90E-05	0.000214835
ENSG00000063180	CA11	41.26131557	10.13176088	2.024571668	3.95E-05	0.000217236
ENSG00000228561	AC026355.1	1.48935231	22.49911668	-3.872796125	4.00E-05	0.000219799
ENSG00000278962	AC092645.1	63.47237527	21.85115044	1.541925291	4.10E-05	0.000224781
ENSG00000079482	OPHN1	118.7917591	202.8539733	-0.773498907	4.12E-05	0.000225898
ENSG00000245711	NADK2-AS1	41.84583568	10.48152917	1.994300265	4.22E-05	0.000231234
ENSG00000051341	POLQ	113.8172644	53.82529329	1.077897643	4.29E-05	0.000235189
ENSG00000099869	IGF2-AS	22.47312503	0.678908432	5.012239008	4.34E-05	0.000237509
ENSG00000215399	HMGB3P7	1.249100681	20.37657475	-4.029137815	4.35E-05	0.000238063
ENSG00000022567	SLC45A4	78.20750081	150.9103991	-0.944472134	4.44E-05	0.000243025
ENSG00000173391	OLR1	0.297870462	17.09207673	-5.672088584	4.53E-05	0.000247647
ENSG00000094804	CDC6	152.2508197	74.94266087	1.024110384	4.55E-05	0.000248608
ENSG00000168679	SLC16A4	83.59331414	167.3636557	-1.005635019	4.62E-05	0.00025244
ENSG00000102362	SYTL4	157.0174735	83.32022244	0.913061894	4.68E-05	0.000255202
ENSG00000169750	RAC3	18.0885271	57.14950199	-1.65834965	4.74E-05	0.000258248
ENSG00000165209	STRBP	107.7568153	189.657659	-0.817737647	4.85E-05	0.000263814
ENSG00000127083	OMD	192.3180756	103.3600938	0.89425616	4.90E-05	0.00026657
ENSG00000250423	KIAA1210	23.56093911	1.393715224	4.073743144	4.92E-05	0.000267455
ENSG00000169851	PCDH7	49.36772445	106.3955086	-1.109436168	4.94E-05	0.000268199
ENSG00000267002	AC060780.1	170.0666927	86.43204981	0.977818146	4.97E-05	0.000270067
ENSG00000122952	ZWINT	201.5171375	114.2856811	0.820465744	5.08E-05	0.000275501
ENSG00000171889	MIR31HG	66.61711521	22.75936248	1.548938717	5.10E-05	0.000276517
ENSG00000161249	DMKN	47.80091772	102.5505131	-1.099651652	5.13E-05	0.000278379
ENSG00000184661	CDCA2	93.0337231	34.7069118	1.428495909	5.18E-05	0.000280683
ENSG00000171160	MORN4	110.4379284	189.9583125	-0.780449496	5.20E-05	0.000281581
ENSG00000153291	SLC25A27	191.213357	106.670389	0.843142658	5.25E-05	0.000283951
ENSG00000169660	HEXDC	162.2118506	85.93002608	0.917886375	5.28E-05	0.00028529
ENSG00000178852	EFCAB13	89.7862263	39.60150748	1.180649481	5.50E-05	0.000297107
ENSG00000228784	LINC00954	55.80438108	18.25443456	1.611361176	5.52E-05	0.00029834
ENSG00000100036	SLC35E4	77.80920068	145.3631763	-0.902364691	5.62E-05	0.000303535
ENSG00000105255	FSD1	44.31360857	10.82964539	2.031698737	5.67E-05	0.000306012
ENSG00000185585	OLFML2A	66.71349429	135.5614041	-1.022581371	5.73E-05	0.000308794
ENSG00000214455	RCN1P2	131.8764344	59.12658549	1.153138238	5.77E-05	0.000310745
ENSG00000235501	AC105942.1	60.43290803	125.1617277	-1.048651108	5.85E-05	0.000314615

ENSG00000133119	RFC3	113.4744405	52.04260732	1.122812797	5.86E-05	0.000315432
ENSG00000276365	MIR145	10.56673266	43.48610725	-2.056741623	6.08E-05	0.00032625
ENSG00000152402	GUCY1A2	106.0342171	4.776291266	4.46388207	6.12E-05	0.000328405
ENSG00000163694	RBM47	37.73389597	8.791268043	2.103694503	6.21E-05	0.000332635
ENSG00000167723	TRPV3	90.14709224	36.52608667	1.299336157	6.26E-05	0.00033502
ENSG00000121775	TMEM39B	210.9549404	121.5313188	0.796684802	6.28E-05	0.000336107
ENSG00000081913	PHLPP1	184.5775362	103.8139578	0.829807459	6.35E-05	0.000339851
ENSG00000162840	MT2P1	35.21460316	7.047784537	2.322654762	6.37E-05	0.000340938
ENSG00000129173	E2F8	38.66466268	8.155267927	2.252624524	6.39E-05	0.000341811
ENSG00000284391	AL139398.1	0.335025778	16.15417281	-5.589269692	6.51E-05	0.000348044
ENSG00000198168	SVIP	51.22315894	110.898891	-1.11497146	6.57E-05	0.000350942
ENSG00000267199	AP001029.2	13.69163702	47.12727704	-1.783396849	6.78E-05	0.000361444
ENSG00000138380	CARF	201.2406173	118.1870967	0.766373035	6.82E-05	0.000363766
ENSG00000124635	HIST1H2BJ	38.36364843	8.827166402	2.126136422	7.05E-05	0.000374602
ENSG00000257365	FNTB	108.0257019	193.3357886	-0.83970879	7.23E-05	0.000383935
ENSG00000197233	OR1J2	11.63005676	0	5.90744026	7.25E-05	0.000384785
ENSG00000196754	S100A2	59.8713673	18.59924668	1.687088327	7.28E-05	0.000385962
ENSG00000162390	ACOT11	24.00824742	69.07951434	-1.531042144	7.36E-05	0.00039007
ENSG00000251136	AF117829.1	135.0733222	64.42784043	1.068973108	7.42E-05	0.000393052
ENSG00000187607	ZNF286A	143.2695678	74.11861341	0.951891172	7.44E-05	0.000394232
ENSG00000162267	ITIH3	1.828149805	61.02698533	-5.048423437	7.44E-05	0.000394245
ENSG00000110876	SELPLG	19.36796193	56.76413062	-1.552656844	7.73E-05	0.000408903
ENSG00000223653	AL078459.1	9.308389283	38.81244843	-2.057666915	7.77E-05	0.000410985
ENSG00000260966	AP001486.2	40.81114646	10.12309887	2.008464856	7.79E-05	0.000411953
ENSG00000272269	AL138724.1	24.37046356	2.17040641	3.516429166	7.89E-05	0.000416157
ENSG00000093134	VNN3	11.70741684	0	5.915840231	7.90E-05	0.000416827
ENSG00000180035	ZNF48	73.59082261	143.3877207	-0.959661111	7.97E-05	0.000420022
ENSG00000260439	LMF1-AS1	12.40915274	43.77068703	-1.815876425	8.00E-05	0.000421521
ENSG00000143028	SYPL2	6.951988014	33.861728	-2.291996316	8.04E-05	0.000423449
ENSG00000110042	DTX4	52.0721659	116.5481929	-1.165834769	8.10E-05	0.000426253
ENSG00000230918	AC008063.1	118.5076253	59.29586969	0.99769235	8.29E-05	0.000436248
ENSG00000267787	AC027097.2	26.38061823	3.89160973	2.766809375	8.33E-05	0.000438179
ENSG00000131941	RHPN2	11.4300099	44.48725234	-1.969543132	8.40E-05	0.000441641
ENSG00000197959	DNM3	81.32674965	146.7840214	-0.852013957	8.55E-05	0.000449366
ENSG00000173077	1-Dec	130.0955894	67.83008957	0.9386296	8.85E-05	0.000464637
ENSG00000177432	NAP1L5	114.469274	194.158139	-0.764413211	8.91E-05	0.000467589
ENSG00000120647	CCDC77	120.8596269	59.46409238	1.020286761	9.09E-05	0.000475979
ENSG00000185507	IRF7	41.00814386	93.95345375	-1.195441566	9.15E-05	0.000478672
ENSG00000236304	AP001189.1	39.1291964	88.10403187	-1.168720934	9.15E-05	0.000478672
ENSG00000153933	DGKE	216.7134684	123.5419065	0.80961778	9.21E-05	0.000481325
ENSG00000169083	AR	76.47824593	29.59641775	1.371733001	9.30E-05	0.000485721
ENSG00000184949	FAM227A	75.43799141	29.37196384	1.361383072	9.43E-05	0.000491652
ENSG00000039068	CDH1	0.944409046	19.41833797	-4.374042447	9.43E-05	0.000491731
ENSG00000013563	DNASE1L1	102.7291015	177.7996322	-0.792677961	9.54E-05	0.000497452
ENSG00000156675	RAB11FIP1	32.50343486	78.90812111	-1.285559798	9.63E-05	0.000502017
ENSG00000146232	NFKBIE	153.0653759	86.2198573	0.827846189	0.000100819	0.000524079
ENSG00000230623	AC104461.1	4.102069075	27.15534501	-2.737507487	0.000104235	0.000540485
ENSG00000231584	FAHD2CP	4.559781624	28.16845622	-2.607620934	0.000105	0.000544319
ENSG00000174371	EXO1	70.02561976	27.829002	1.334324348	0.000107979	0.000558789
ENSG00000178531	CTXN1	39.95638965	91.00315852	-1.192281756	0.000108486	0.000561275
ENSG00000187957	DNER	11.05100763	0	5.83293395	0.00010867	0.000561948
ENSG00000008300	CELSR3	163.6689809	83.48455047	0.973606739	0.000109735	0.000566752
ENSG00000258441	LINC00641	192.636221	113.3644865	0.764889104	0.000110365	0.000569862
ENSG00000187049	TMEM216	140.9619861	75.16175687	0.906663221	0.000112083	0.000577877
ENSG00000130052	STARD8	27.14302241	69.24453939	-1.350838573	0.000112148	0.000578067
ENSG00000173207	CKS1B	147.0999798	79.48729946	0.890614799	0.000113734	0.000585808
ENSG00000005379	TSPOAP1	74.29982307	136.3544643	-0.876717892	0.000114093	0.000587512
ENSG00000085840	ORC1	44.86618959	13.31351998	1.752339202	0.000114868	0.000591068
ENSG00000188662	HILS1	3.154610573	23.60164391	-2.91187878	0.0001149	0.000591086
ENSG00000016402	IL20RA	3.624615271	29.59516744	-3.065981229	0.000116176	0.000597205
ENSG00000006747	SCIN	9.43654703	37.0248063	-1.979058074	0.000116971	0.000600849
ENSG00000180190	TDRP	130.5358879	69.4771471	0.911046988	0.000117345	0.000602593
ENSG00000183421	RIPK4	0	10.17632125	-5.884533473	0.000117932	0.000605339
ENSG00000160447	PKN3	64.53686502	131.700585	-1.033115407	0.00011872	0.000609081
ENSG00000176485	PLA2G16	94.91455848	167.7214954	-0.822396989	0.000119887	0.000614769
ENSG00000255440	AP001085.1	0	10.15404107	-5.881815342	0.000120272	0.000616287

ENSG00000168646	AXIN2	80.78315658	147.6011653	-0.868179654	0.000124438	0.000636072
ENSG00000123219	CENPK	123.0626582	60.39168223	1.02456207	0.000127679	0.000651519
ENSG00000229373	LINC00452	19.51281147	56.0245881	-1.527282061	0.000129822	0.000661485
ENSG00000185442	FAM174B	40.58076546	87.70599737	-1.112401242	0.000132015	0.000672002
ENSG00000151500	THYN1	160.3023143	91.41731182	0.809610728	0.000132464	0.000673955
ENSG00000269899	AC025857.2	9.545591457	37.52557971	-1.967075174	0.000135749	0.000690167
ENSG00000171448	ZBTB26	201.3127853	119.3725832	0.754580663	0.000136902	0.000695348
ENSG00000237596	AL138828.1	18.23095513	0.713154737	4.697682302	0.000139573	0.000708223
ENSG00000145247	OCIAD2	88.15130218	153.8623464	-0.803378128	0.000142076	0.000720575
ENSG00000262003	AC087392.1	13.37330304	46.42073051	-1.795298884	0.000142984	0.000724826
ENSG00000274307	AC023449.2	10.5233841	39.75251261	-1.912318044	0.000143451	0.00072702
ENSG00000066382	MPPED2	28.5543867	71.76625967	-1.333196183	0.00014634	0.000741122
ENSG00000276980	AC008760.2	10.8274478	0	5.802071266	0.000146456	0.000741345
ENSG00000177679	SRRM3	23.05315116	66.41574621	-1.527064375	0.000148669	0.000752184
ENSG00000154040	CABYR	42.41815795	93.70312028	-1.147743336	0.00015121	0.000764483
ENSG00000258875	AL135818.1	50.27667744	16.44741461	1.608764506	0.000152805	0.000771237
ENSG00000169220	RGS14	11.52541198	40.6314345	-1.81760987	0.000155047	0.000781799
ENSG00000197385	ZNF860	0.944409046	17.69393701	-4.236036994	0.000156479	0.000788444
ENSG00000124596	OARD1	166.7922803	94.07972317	0.826407464	0.000162803	0.000819121
ENSG00000242960	FTH1P23	98.93602936	43.14841156	1.202022248	0.00016711	0.000839375
ENSG00000164430	CGAS	120.1028783	63.78387532	0.912513209	0.000168174	0.000844303
ENSG00000186185	KIF18B	101.5252332	48.10323958	1.079354259	0.000171153	0.000858441
ENSG00000232882	PHKA1P1	5.679374361	28.89192502	-2.354249573	0.000171596	0.000860458
ENSG00000087586	AURKA	166.381688	97.98340548	0.765358519	0.000171736	0.000860949
ENSG00000198046	ZNF667	153.8939193	86.07681715	0.838741733	0.000171922	0.000861679
ENSG00000248213	CICP16	23.68434814	3.1817591	2.905497459	0.000175316	0.000877845
ENSG00000161981	SNRNP25	110.2529684	187.7690365	-0.766361174	0.000177318	0.000887439
ENSG00000170989	S1PR1	130.2394619	61.15541501	1.094179651	0.0001789	0.000895145
ENSG00000198807	PAX9	2.186688555	19.58130923	-3.165029799	0.000181057	0.000905285
ENSG00000144481	TRPM8	37.55736209	8.635306745	2.11050865	0.000181778	0.000908673
ENSG00000154839	SKA1	49.49786033	15.52518266	1.678203929	0.000181902	0.000909075
ENSG00000224945	AL353150.1	22.48164547	2.398459699	3.208431379	0.000183539	0.000915938
ENSG00000064692	SNCAIP	113.0510885	57.89844861	0.964421447	0.000185412	0.000924496
ENSG00000196584	XRCC2	53.61284948	18.49280191	1.534280221	0.000186589	0.000929824
ENSG00000153558	FBXL2	57.64312377	113.9143747	-0.985049446	0.000186973	0.000931484
ENSG00000225937	PCA3	2.866610738	22.70158568	-3.003419122	0.000190467	0.000947789
ENSG00000258773	AC087636.1	0	9.495760768	-5.784211899	0.000192033	0.000954897
ENSG00000269902	AC234772.2	26.97016591	4.935556672	2.455440223	0.000192747	0.000958218
ENSG00000229657	AL391822.1	7.511295883	35.4768883	-2.241538439	0.00019646	0.000975516
ENSG00000176371	ZSCAN2	76.14161522	31.67564962	1.266928765	0.00019689	0.00097742
ENSG00000143554	SLC27A3	75.98240107	141.3343684	-0.896253645	0.000206862	0.001023753
ENSG00000138658	ZGRF1	135.8524182	76.10171459	0.835564249	0.000208064	0.001029211
ENSG00000171873	ADRA1D	2.203380354	20.10436283	-3.196929241	0.000208783	0.001032281
ENSG00000204642	HLA-F	58.12497723	112.3155834	-0.949609809	0.000209607	0.00103611
ENSG00000167799	NUDT8	20.77932621	58.3187633	-1.493298765	0.00021045	0.001040029
ENSG00000128602	SMO	143.9635758	79.24882564	0.859581058	0.000211173	0.001043105
ENSG00000070193	FGF10	0.609383268	16.46268483	-4.729276156	0.000214757	0.001060309
ENSG00000262001	DLGAP1-AS2	61.364397	23.78308303	1.367594328	0.000215419	0.001063323
ENSG00000186862	PDZD7	8.05246743	33.00993599	-2.030800316	0.00021807	0.001075392
ENSG00000240666	MME-AS1	68.23767473	28.7838282	1.246041172	0.000218679	0.00107814
ENSG00000177398	UMODL1	38.29418084	9.780340548	1.965160422	0.00022015	0.001084314
ENSG00000166292	TMEM100	21.40845074	2.465300255	3.120325635	0.000222599	0.001095134
ENSG00000140022	STON2	2.491380189	20.2841499	-3.024915474	0.000223082	0.001096996
ENSG00000237489	C10orf143	59.05888771	20.39184498	1.53501138	0.000226737	0.001113917
ENSG00000169067	ACTBL2	0	9.797262829	-5.830336335	0.000231345	0.001135218
ENSG00000186205	1-Mar	8.231422838	33.43150099	-2.031071077	0.000232089	0.001138601
ENSG00000152229	PSTPIP2	190.0470874	109.7105471	0.791451525	0.000235518	0.001154267
ENSG00000162241	SLC25A45	103.4417793	181.5876995	-0.814395775	0.000245683	0.001202181
ENSG00000118507	AKAP7	95.61959857	44.34531096	1.109686507	0.000246062	0.001203471
ENSG00000133116	KL	0	9.502770726	-5.785151527	0.000246228	0.001204
ENSG00000112319	EYA4	14.18210523	0.351420336	5.231283831	0.000247321	0.001208493
ENSG00000164306	PRIMPOL	130.0211844	70.07064064	0.892718942	0.000248143	0.001212228
ENSG00000088882	CPXM1	40.78458403	11.89752457	1.776421881	0.000248346	0.001212931
ENSG00000249661	TNRC18P1	9.907901924	0	5.679045426	0.000252171	0.001230487
ENSG00000115896	PLCL1	50.05814519	99.4512665	-0.989552506	0.000253407	0.001235627
ENSG00000125434	SLC25A35	23.83979057	61.03109292	-1.352461558	0.000255654	0.001246293

ENSG00000205746	AC126755.1	73.02963094	155.9917708	-1.095408868	0.000257956	0.001256923
ENSG00000148735	PLEKHS1	17.87860962	1.402377236	3.671033944	0.000258593	0.001259439
ENSG00000179967	PPP1R14BP3	86.78391438	148.5910413	-0.775025475	0.000258982	0.001260744
ENSG00000066583	ISOC1	143.5854803	77.05478226	0.896320717	0.000264713	0.001286243
ENSG00000179862	CITED4	23.83539092	60.88515042	-1.355134581	0.000265142	0.001288024
ENSG00000152253	SPC25	46.94581185	14.32527442	1.712558688	0.000273072	0.001324696
ENSG00000197077	KIAA1671	48.17174862	97.050753	-1.012478619	0.000274427	0.001330032
ENSG00000172322	CLEC12A	37.27303964	84.07833929	-1.172281678	0.000275073	0.001332543
ENSG00000268575	AL031282.2	58.7497021	20.41988481	1.526214444	0.000276286	0.001337797
ENSG00000100342	APOL1	42.1041293	88.91981906	-1.075745903	0.000277392	0.001342841
ENSG00000006062	MAP3K14	51.07749281	102.5628809	-1.003060315	0.000277578	0.001343429
ENSG00000186765	FSCN2	17.44808743	55.36545923	-1.665922371	0.000281492	0.001360794
ENSG00000143126	CELSR2	17.89970107	53.17002182	-1.564475792	0.000283179	0.001368
ENSG00000251348	HSPD1P11	31.70522556	6.660064099	2.249378188	0.000283344	0.001368478
ENSG00000141542	RAB40B	84.10855119	146.9939021	-0.807317958	0.000286684	0.001383327
ENSG00000162511	LAPTM5	135.3901414	66.378007	1.032483598	0.000293651	0.00141498
ENSG00000158691	ZSCAN12	126.7992731	70.57967433	0.846125138	0.00029777	0.001432566
ENSG00000159905	ZNF221	80.77067989	36.08194622	1.159961632	0.000297781	0.001432566
ENSG00000280219	AC093908.1	39.82141073	11.64553905	1.77633651	0.000299637	0.001440498
ENSG00000143061	IGSF3	63.83028608	26.22775515	1.280604006	0.000302475	0.001453805
ENSG00000088808	PPP1R13B	45.18757302	92.28176697	-1.030914404	0.000302665	0.001454381
ENSG00000224039	CDYLP1	9.510857662	0	5.615700298	0.000315648	0.001513628
ENSG00000109794	FAM149A	16.41339821	48.49540797	-1.5606899	0.000316546	0.001517588
ENSG00000138439	FAM117B	116.4813125	59.15733888	0.979569218	0.000318451	0.001526018
ENSG00000125703	ATG4C	183.5702218	105.3009013	0.803772376	0.000325407	0.001555777
ENSG00000197301	AC090673.1	133.4087862	73.11912037	0.865924114	0.000326076	0.001558616
ENSG00000219736	AL356473.1	0	8.792920097	-5.673251767	0.00032709	0.001563103
ENSG00000165028	NIPSNAP3B	73.18327979	31.8694566	1.198937547	0.000327278	0.001563645
ENSG00000196074	SYCP2	81.49143478	36.97169038	1.140912242	0.0003291	0.001571271
ENSG00000159314	ARHGAP27	68.21120663	126.8904943	-0.897143871	0.000335944	0.001601381
ENSG00000162415	ZSWIM5	9.278055139	0	5.584514237	0.000337212	0.001607057
ENSG00000007968	E2F2	29.44557656	4.66995297	2.673406804	0.000338495	0.0016128
ENSG00000104983	CCDC61	77.91384545	137.0469909	-0.816547257	0.000339378	0.001616517
ENSG00000093009	CDC45	47.30362834	16.16118277	1.552728318	0.000344242	0.001637565
ENSG00000179598	PLD6	24.09000716	61.00305309	-1.342277003	0.000349698	0.001662803
ENSG00000249673	NOP14-AS1	72.97201211	133.0872903	-0.869251562	0.000351269	0.001669848
ENSG00000105711	SCN1B	100.7094252	169.2060899	-0.750898284	0.000355047	0.001687037
ENSG00000153044	CENPH	74.20379305	29.72298243	1.3167423	0.000355368	0.00168818
ENSG00000108771	DHX58	85.96011964	160.9089629	-0.905676054	0.000359529	0.001706394
ENSG00000185634	SHC4	7.788702829	33.43190273	-2.10050289	0.000359697	0.0017068
ENSG00000234279	LINC01937	9.240899823	0	5.579447435	0.000360506	0.00170986
ENSG00000282849	AL359834.1	0.304691634	12.32444748	-5.198286704	0.00036392	0.001722529
ENSG00000136274	NACAD	71.39838421	135.4980565	-0.925411657	0.000376306	0.001779136
ENSG00000115392	FANCL	156.8414732	87.09403596	0.851244061	0.000376941	0.001781328
ENSG00000169946	ZFPM2	14.20319668	43.3627402	-1.605441889	0.000377193	0.001782116
ENSG00000139146	SINHCAF	148.0869208	85.31113705	0.79548772	0.000378148	0.001786222
ENSG00000243055	GK-AS1	24.56368925	4.596102455	2.424903362	0.000392255	0.001849507
ENSG00000152977	ZIC1	13.41727953	41.23524211	-1.621365574	0.000394805	0.001861108
ENSG00000157654	PALM2-AKAP2	65.31416742	27.6697366	1.239147446	0.000395889	0.001865796
ENSG00000279881	AC041040.1	38.58353087	11.31805095	1.775150997	0.000413059	0.001943206
ENSG00000183891	TTC32	94.35417929	43.57221916	1.113114622	0.000417226	0.001961478
ENSG00000034053	APBA2	23.13195577	4.210435815	2.457719855	0.000422646	0.001984724
ENSG00000099994	SUSD2	3.594281127	24.77380759	-2.817618567	0.000423379	0.001987272
ENSG00000166831	RBPMS2	21.6493303	3.1817591	2.774637132	0.000424036	0.001989905
ENSG00000187243	MAGED4B	34.24793701	8.768987857	1.968113388	0.000425812	0.00199779
ENSG00000126709	IFI6	234.9142822	502.8910822	-1.098989395	0.000429861	0.002015425
ENSG00000165490	DDIAS	47.22259086	16.47670475	1.519870959	0.000433493	0.002031997
ENSG00000158050	DUSP2	10.85231097	39.05617369	-1.845725772	0.000442524	0.002071538
ENSG00000103316	CRYM	4.650784055	23.51747933	-2.333927045	0.000448237	0.002096867
ENSG00000105464	GRIN2D	74.63654811	133.1892257	-0.838225449	0.00045819	0.002139111
ENSG00000271614	ATP2B1-AS1	63.68471427	27.34720466	1.219061817	0.000462416	0.002156427
ENSG00000152284	TCF7L1	44.92613562	92.17252631	-1.037315379	0.000464979	0.002166925
ENSG00000077274	CAPN6	0	8.708755516	-5.661239705	0.000465734	0.002169956
ENSG00000134222	PSRC1	101.9649037	53.10688712	0.943385885	0.000469413	0.002185634
ENSG00000056558	TRAF1	122.5373619	68.26732654	0.843144664	0.000470301	0.002189278
ENSG00000108381	ASPA	114.1802028	61.87081235	0.882250937	0.000471978	0.002196105

ENSG00000128271	ADORA2A	21.51057967	2.820024699	2.938042227	0.000476662	0.002214933
ENSG00000249790	AC092490.1	3.794327985	23.41114103	-2.632873541	0.000477919	0.002220278
ENSG00000254102	AC090136.3	9.142448285	0	5.559889426	0.000482198	0.002238165
ENSG00000279030	AC007336.3	102.7684897	51.16949016	1.006023999	0.000482748	0.002240218
ENSG00000112796	ENPP5	18.85020902	50.94514272	-1.429150665	0.000483187	0.002241755
ENSG00000248323	LUCAT1	29.92168017	6.958663796	2.09914393	0.000484295	0.002245399
ENSG00000133874	RNF122	74.48927704	32.49925025	1.195028187	0.000485038	0.00224834
ENSG00000254810	AP001189.3	26.03742108	69.59515624	-1.425476709	0.000491485	0.002277718
ENSG00000123700	KCNJ2	40.1100385	84.17902441	-1.06825524	0.000496796	0.002299267
ENSG00000138395	CDK15	11.99299516	39.48474865	-1.712185721	0.000511636	0.002362706
ENSG00000170962	PDGFD	89.34564894	158.8131733	-0.834425694	0.000518274	0.002392297
ENSG00000166803	PCLAF	36.43229837	10.09586252	1.849307618	0.000522357	0.002409546
ENSG00000167487	KLHL26	77.23697272	137.1966392	-0.83069363	0.000523807	0.002415699
ENSG00000174963	ZIC4	23.3888992	57.34690835	-1.294933332	0.000528704	0.00243714
ENSG00000279086	AC073130.3	16.43691118	47.89581442	-1.539135963	0.000528807	0.00243714
ENSG00000203814	HIST2H2BF	27.10011725	4.481397421	2.581034608	0.000536823	0.002471353
ENSG00000099282	TSPAN15	4.494713685	23.79009299	-2.425290992	0.000538125	0.002476799
ENSG00000073150	PANX2	89.18221968	43.00772049	1.054609381	0.00054221	0.002492303
ENSG00000251615	AC104825.1	40.6935816	88.30364359	-1.116313619	0.000542825	0.002494578
ENSG00000132881	CPLANE2	40.61792077	83.11525282	-1.03403519	0.000548082	0.002516643
ENSG00000112182	BACH2	39.46799389	11.53909428	1.773191091	0.000552359	0.002535596
ENSG00000185811	IKZF1	9.104570708	0	5.554535007	0.000553099	0.002538435
ENSG00000159307	SCUBE1	30.05360964	6.233542938	2.26070994	0.000554785	0.002544492
ENSG00000183454	GRIN2A	15.09375862	0.690874552	4.434736793	0.000554917	0.00254454
ENSG00000174749	FAM241A	81.29103886	140.3533673	-0.787282831	0.000555896	0.002548469
ENSG00000260075	NSFP1	2.464095501	25.12107525	-3.342615925	0.00055754	0.002554879
ENSG00000099251	HSD17B7P2	27.77519639	65.80668717	-1.244080422	0.00055907	0.002561329
ENSG00000241886	AC112496.1	21.09011676	3.497281077	2.587852363	0.000561207	0.002569989
ENSG00000163545	NUAK2	110.2345532	57.99487459	0.923662483	0.00056894	0.002604832
ENSG00000279672	AP006621.5	53.30752991	103.7685488	-0.961013005	0.000583506	0.002666654
ENSG00000265112	MIR3153	21.37192336	2.376179513	3.144493482	0.000587367	0.002682129
ENSG00000158856	DMTN	4.227805301	24.18772575	-2.494586958	0.000588236	0.002685507
ENSG00000156299	TIAM1	3.655577349	22.79896669	-2.624652912	0.000609787	0.002780854
ENSG00000261220	AC103706.1	43.08506573	14.08485327	1.615046272	0.000611893	0.002789848
ENSG00000285796	AL162458.1	135.4912734	71.2615916	0.927513285	0.000615366	0.002804456
ENSG00000198246	SLC29A3	128.8131051	73.12863095	0.816308203	0.000618617	0.002818041
ENSG00000128683	GAD1	90.70137252	155.1205396	-0.775984198	0.000620496	0.002825979
ENSG00000174343	CHRNA9	34.11842907	9.113398235	1.9053253	0.000628609	0.002857935
ENSG00000256195	AP002518.1	3.632064377	22.8707634	-2.633282847	0.000629362	0.002860738
ENSG00000064787	BCAS1	10.73644537	35.742492	-1.742893618	0.000633408	0.002877247
ENSG00000166823	MESP1	0.297870462	12.53392643	-5.224648559	0.000635913	0.002887033
ENSG00000108984	MAP2K6	155.6770213	87.3244834	0.834857885	0.000636518	0.002888227
ENSG00000196544	BORCS6	54.08599796	103.2574613	-0.934687221	0.00063816	0.002894418
ENSG00000215158	AC138409.2	71.41139862	32.35991596	1.142251099	0.000641831	0.002909801
ENSG00000259583	AC015712.2	81.39936102	38.36900499	1.083421621	0.000642256	0.002911095
ENSG00000175772	LINC01106	26.69033745	5.647059355	2.248802345	0.000642648	0.002912237
ENSG00000157456	CCNB2	138.2289339	76.60693595	0.85140245	0.000643919	0.002915463
ENSG00000185875	THNSL1	71.54027863	31.71525383	1.174384823	0.000647641	0.002930405
ENSG00000224081	SLC44A3-AS1	6.942117386	28.66706937	-2.054439937	0.000658914	0.002975648
ENSG00000259943	AL050341.2	44.57441804	13.18159739	1.765895397	0.000664967	0.003000985
ENSG00000198283	OR5B21	9.097749536	0	5.553628994	0.000665361	0.003002117
ENSG00000110076	NRXN2	76.91389003	37.17045353	1.049461316	0.000665702	0.003002837
ENSG00000223947	AC016738.1	23.63534406	3.924203981	2.600970932	0.000665809	0.003002837
ENSG00000170873	MTSS1	21.15590696	55.55350657	-1.392658752	0.000684736	0.003084195
ENSG00000100784	RPS6KA5	48.31929856	17.64401874	1.458629057	0.000692853	0.003118064
ENSG00000229619	MBNL1-AS1	82.75166196	141.2810637	-0.770175929	0.000698071	0.003138838
ENSG00000236453	AC003092.1	23.18643082	4.143595258	2.474695844	0.000701156	0.003151348
ENSG00000230606	AC092683.1	61.40325157	25.82847034	1.246933958	0.000712193	0.003198195
ENSG00000188185	LINC00265	83.48866936	40.60790401	1.037156094	0.000720196	0.003232047
ENSG00000244137	AL512328.1	0	8.527316395	-5.627382049	0.000726679	0.003255537
ENSG00000198729	PPP1R14C	26.81176835	63.36601617	-1.241246454	0.000732261	0.00327843
ENSG00000170915	PAQR8	131.2568314	75.65221622	0.796513672	0.000734508	0.003286371
ENSG00000257642	KCCAT198	12.71007266	40.43336837	-1.668039682	0.000740005	0.003310258
ENSG00000214243	AC004980.2	8.289669605	0	5.423275819	0.000741496	0.003316215
ENSG00000270127	AC027020.2	20.52678244	2.796092459	2.874003754	0.000754191	0.003368657
ENSG00000243116	AC092943.1	0	7.762591328	-5.492821497	0.000757029	0.003379884

ENSG00000214193	SH3D21	33.63792581	73.56968024	-1.127580717	0.000759696	0.003390339
ENSG00000064886	CHI3L2	8.147241579	0	5.394547739	0.000763063	0.003403908
ENSG00000153391	INO80C	40.9429816	82.65533392	-1.011487674	0.000777129	0.003462208
ENSG00000242113	RN7SL124P	11.74592236	0.339454216	4.96317209	0.000777824	0.003464567
ENSG00000143036	SLC44A3	6.458470344	27.93875088	-2.102417963	0.000778228	0.003465626
ENSG00000138435	CHRNA1	0.63289624	13.89464566	-4.474830666	0.000782454	0.003482955
ENSG00000173320	STOX2	8.116907435	0	5.389874728	0.00078555	0.003495247
ENSG00000197989	SNHG12	83.37298831	147.6838908	-0.821446553	0.000793429	0.003526115
ENSG00000238103	RPL9P7	133.9381992	76.21506284	0.813580857	0.000802984	0.003566725
ENSG00000165272	AQP3	2.142712067	17.03555024	-2.977679582	0.000804603	0.003573156
ENSG00000266852	MIR4482	47.40324553	17.44349707	1.437585752	0.000808673	0.003589702
ENSG00000171951	SCG2	1.912331065	17.27226554	-3.196252486	0.000832435	0.003688115
ENSG00000272563	AC016745.2	14.07063929	0.678908432	4.336223224	0.000840386	0.00372097
ENSG00000272316	AL021368.2	78.28127783	37.26328012	1.072098231	0.000840926	0.003722569
ENSG00000135451	TROAP	102.2560473	55.00167103	0.894217092	0.000843611	0.003733666
ENSG00000256083	AC090673.2	15.91925261	1.415995409	3.499560932	0.000861599	0.003807617
ENSG00000106560	GIMAP2	14.12753586	0.713154737	4.325406856	0.000864135	0.003816397
ENSG00000083782	EPYC	1.211945365	14.73116745	-3.576366285	0.000882962	0.00389295
ENSG00000104848	KCNA7	8.638337727	30.49141335	-1.813137221	0.000890201	0.003923207
ENSG00000167749	KLK4	3.496457523	20.59636777	-2.573468341	0.000894751	0.00394076
ENSG00000026297	RNASET2	75.73227881	131.4004848	-0.794311399	0.000896372	0.003947068
ENSG00000091622	PITPNM3	36.53631521	77.28595086	-1.081103935	0.00090828	0.003997811
ENSG00000250889	LINC01336	1.272613652	14.70022525	-3.5494628	0.00090923	0.003999463
ENSG00000239857	GET4	52.22751401	96.89324612	-0.892723228	0.000910255	0.00400228
ENSG00000204311	PJVK	63.45335627	27.41569727	1.21250466	0.000946566	0.004154927
ENSG00000130702	LAMA5	21.10438704	51.5914569	-1.28698563	0.000951468	0.004173808
ENSG00000230067	HSPD1P6	7.8289076	0	5.337127236	0.000960464	0.004209729
ENSG00000154133	ROBO4	19.56118761	2.739565969	2.817998588	0.000964404	0.004225222
ENSG00000196666	FAM180B	0	7.390542861	-5.422474836	0.00096812	0.00424061
ENSG00000114854	TNNC1	5.57168013	24.4859237	-2.131533786	0.000982725	0.00430097
ENSG00000002933	TMEM176A	7.791752284	0	5.331153407	0.000985313	0.004310966
ENSG00000111728	ST8SIA1	23.56103343	4.856749996	2.268803647	0.0010021	0.004379328
ENSG00000276012	AL160153.1	5.367861555	25.3055232	-2.244880644	0.00101103	0.004415802
ENSG00000065328	MCM10	63.406236	26.50367292	1.257623332	0.001011505	0.004415802
ENSG00000165879	FRAT1	57.40519934	23.32727173	1.293967659	0.00101249	0.00441918
ENSG00000197457	STMN3	9.566054974	33.4846169	-1.802871804	0.001014895	0.004427823
ENSG00000233858	LINC02599	14.7914885	1.054261007	3.810743352	0.001028269	0.004485231
ENSG00000256660	CLEC12B	1.540149971	15.31970483	-3.305153277	0.001030473	0.004493906
ENSG00000241399	CD302	123.9243966	70.92272793	0.803808573	0.001045689	0.004553595
ENSG00000204851	PNMA8B	46.84843164	91.0819652	-0.963214291	0.00105402	0.004586999
ENSG00000042781	USH2A	7.697700397	30.11235493	-1.96073837	0.001057832	0.00460263
ENSG00000204618	RNF39	46.06072089	17.26330826	1.41890781	0.001060842	0.004614764
ENSG00000277945	AC107308.1	14.56855661	1.085203205	3.779135583	0.001062688	0.004621828
ENSG00000219565	ZNF259P1	23.59136758	4.880682235	2.266846731	0.001068142	0.004642644
ENSG00000026559	KCNG1	65.11277036	29.38888612	1.14534012	0.001071005	0.004653735
ENSG00000148426	PROSER2	9.389521087	31.49615783	-1.749003394	0.001074152	0.004664877
ENSG00000256616	AP002414.2	11.83010362	35.68596551	-1.592724074	0.001083916	0.004702384
ENSG00000188611	ASAH2	47.53985351	14.59954013	1.702035855	0.001085292	0.004707371
ENSG00000239268	AC092691.1	0	7.656146563	-5.475541355	0.001121367	0.004855762
ENSG00000073849	ST6GAL1	15.01127662	1.393715224	3.422207766	0.001133808	0.004905561
ENSG00000177465	ACOT4	15.29927645	42.28960959	-1.466316642	0.00113496	0.004909527
ENSG00000131127	ZNF141	129.5728089	76.65355012	0.756008155	0.001136793	0.004916436
ENSG00000180884	ZNF792	66.61469369	26.67890557	1.316178403	0.001137894	0.004920176
ENSG00000257732	AC089983.1	0.97474319	12.99138979	-3.777605778	0.001139217	0.004924873
ENSG00000119703	ZC2HC1C	40.59745726	14.37809506	1.497088388	0.001139751	0.004926161
ENSG00000232034	AC092168.2	13.88109099	1.052608954	3.723907749	0.001142259	0.004933987
ENSG00000260822	AC004656.1	23.27985477	4.945870737	2.237998776	0.001142271	0.004933987
ENSG00000178966	RMI1	134.9509143	77.38498392	0.804236317	0.00114618	0.004947794
ENSG00000128645	HOXD1	4.001196016	21.40855557	-2.41039026	0.001148529	0.004956908
ENSG00000277758	FO681492.1	2.53158496	20.41151807	-3.024073107	0.00115176	0.00496777
ENSG00000279198	AC008894.3	65.20512299	115.360759	-0.824350379	0.001159647	0.005000755
ENSG00000266835	GAPLINC	2.129069723	16.71672416	-2.954640514	0.001166321	0.005023909
ENSG00000143494	VASH2	24.17723787	4.469431301	2.420854948	0.001172013	0.00504469
ENSG00000174007	CEP19	91.73508474	48.09953373	0.930875091	0.001174292	0.005051368
ENSG00000261716	HIST2H2BC	127.3255464	66.1029378	0.947862246	0.001177513	0.005062094
ENSG00000271857	AL096865.1	45.96289729	17.93891258	1.36025133	0.001190775	0.005110676

ENSG00000167136	ENDOG	44.60412425	88.14198403	-0.985546621	0.001198506	0.005136454
ENSG00000161888	SPC24	62.32307632	27.09356708	1.20618277	0.00120747	0.005173808
ENSG00000256667	KLRA1P	60.49429858	25.41321828	1.255587875	0.001225067	0.00524331
ENSG00000177990	DPY19L2	63.14489292	28.19073641	1.166587758	0.001237303	0.005291848
ENSG00000078295	ADCY2	0.900432558	12.64162151	-3.772500632	0.001268651	0.005412601
ENSG00000164406	LEAP2	10.55748996	0.361734402	4.808406816	0.001268956	0.005412792
ENSG00000120262	CCDC170	95.61035588	51.21239848	0.901081149	0.001281251	0.005458599
ENSG00000258667	HIF1A-AS2	51.62862931	19.45093222	1.404060298	0.001283069	0.005465166
ENSG00000115339	GALNT3	11.22072035	34.55289783	-1.623838845	0.001303844	0.005549123
ENSG00000164604	GPR85	97.07198011	49.23094936	0.98336753	0.001304346	0.005550127
ENSG00000260025	AC009414.2	28.98544249	65.40729589	-1.16975083	0.00131542	0.005592683
ENSG00000227744	LINC01940	40.10061126	12.31082931	1.701194688	0.001317026	0.005598366
ENSG00000185480	PARPBP	86.64767959	45.30344127	0.935339925	0.001322723	0.00562144
ENSG00000004660	CAMKK1	74.20747044	125.6171373	-0.762001512	0.001328279	0.005643767
ENSG00000258920	FOXN3-AS1	16.38548559	44.68230964	-1.451943488	0.001328519	0.005643767
ENSG00000153363	LINC00467	26.69958014	59.1485704	-1.146022441	0.001333684	0.005663714
ENSG00000002016	RAD52	154.4580702	89.95862103	0.781014217	0.001334029	0.005663714
ENSG00000156521	TYSND1	123.9356174	72.08974664	0.784026154	0.001335071	0.005666986
ENSG00000227036	LINC00511	69.99528562	118.5694592	-0.759297761	0.001351284	0.005733352
ENSG00000238287	AL603839.3	0.944409046	13.16456864	-3.812983475	0.001358503	0.005760581
ENSG00000171790	SLFNL1	14.34122506	40.46856972	-1.495806797	0.001378952	0.005836611
ENSG00000109061	MYH1	0.595740924	12.63666535	-4.357686314	0.001381969	0.005848194
ENSG00000203727	SAMD5	4.211113502	25.09068635	-2.552433319	0.001385787	0.005860782
ENSG00000180353	HCLS1	67.51217112	122.2188892	-0.855771092	0.001391714	0.005881079
ENSG00000232320	AC009299.2	191.4138914	596.9331935	-1.64033566	0.001402563	0.005923323
ENSG00000246090	AP002026.1	22.18135348	53.49119698	-1.264879214	0.001403508	0.005926113
ENSG00000269845	AC092364.2	7.653723909	0	5.308537459	0.001408486	0.005944723
ENSG00000101251	SEL1L2	1.502994655	15.97468102	-3.381346371	0.001410624	0.005952543
ENSG00000149243	KLHL35	14.48437535	41.09095166	-1.497735662	0.001416988	0.005974559
ENSG00000254631	AP001372.1	0	7.020146447	-5.348858189	0.001420169	0.00598676
ENSG00000168453	HR	22.90534648	57.8475753	-1.340714172	0.001432408	0.006037135
ENSG00000253837	AC090197.1	69.85276326	29.82476631	1.223887128	0.001437937	0.006056763
ENSG00000139266	9-Mar	69.60757837	30.422519	1.190597989	0.001468077	0.006171252
ENSG00000279041	AC102945.2	51.47938011	21.17224201	1.285012133	0.001468635	0.00617235
ENSG00000158352	SHROOM4	52.31914438	94.31489288	-0.84971279	0.001486218	0.006239962
ENSG00000157368	IL34	29.77000945	8.331750888	1.832123928	0.001498384	0.006288509
ENSG00000258429	PDF	24.71535996	57.8143905	-1.226351732	0.001500144	0.006294628
ENSG00000143228	NUF2	88.08651311	42.9069289	1.037510759	0.001501272	0.006298093
ENSG00000170264	FAM161A	75.54713016	36.666376	1.042725235	0.001502085	0.006300238
ENSG00000076944	STXBP2	18.29539513	48.68961669	-1.411564735	0.00150251	0.00630075
ENSG00000273012	AL353600.1	7.689529029	0	5.307483704	0.001506404	0.00631454
ENSG00000271605	MILR1	129.0608058	75.82168922	0.769138012	0.001515043	0.006349475
ENSG00000228741	SPATA13	32.53197542	10.22088163	1.6751139	0.001521883	0.006374299
ENSG00000213760	ATP6V1G2	31.04020158	8.439847707	1.883737534	0.001525023	0.006385239
ENSG00000129158	SERGEF	46.94068994	87.5653063	-0.898582476	0.001534092	0.006417701
ENSG00000173531	MST1	100.0226819	54.18548211	0.884353216	0.001542255	0.006447896
ENSG00000253406	AC012613.2	19.75064158	2.821676752	2.813126926	0.00156879	0.006548401
ENSG00000236136	ADORA2BP1	7.300656136	0	5.233922729	0.001581061	0.006598301
ENSG00000092295	TGM1	18.24701899	45.84701654	-1.334572393	0.001601624	0.006677436
ENSG00000237499	AL357060.1	25.88100165	4.330498754	2.596405181	0.001619474	0.006742417
ENSG00000188859	FAM78B	7.17931956	0	5.212822213	0.001623467	0.006756346
ENSG00000255366	AC120036.4	1.502994655	14.32322062	-3.221430964	0.001638565	0.006811022
ENSG00000197977	ELOVL2	18.83351722	3.111614436	2.58885969	0.001643924	0.00682785
ENSG00000221852	KRTAP1-5	2.714940019	19.15078694	-2.793035272	0.001656166	0.006874591
ENSG00000171729	TMEM51	77.98708476	38.88174453	1.004581404	0.001667661	0.006916797
ENSG00000155875	SAXO1	0.914074903	13.23841916	-3.835154401	0.001680096	0.0069656
ENSG00000185386	MAPK11	50.2426659	96.23691315	-0.943562639	0.00171886	0.007114691
ENSG00000235910	APOA1-AS	7.876561478	0	5.351062644	0.001719469	0.007114691
ENSG00000140961	OSGIN1	43.70494756	83.86555622	-0.937509653	0.001742908	0.007205956
ENSG00000139890	REM2	7.091366584	0	5.197299678	0.00174418	0.007209784
ENSG00000205106	DKFZp779M0652	0	6.689354243	-5.278589231	0.001745303	0.007212995
ENSG00000158106	RHPN1	1.844841605	14.3421967	-2.947353425	0.001750412	0.007229813
ENSG00000261189	AL031058.1	48.87590604	91.20317198	-0.903230597	0.001767473	0.007295944
ENSG00000274265	AC245297.3	92.55931875	48.11675128	0.943110479	0.001768726	0.007297198
ENSG00000110203	FOLR3	55.2755919	22.39968187	1.305675992	0.001768827	0.007297198
ENSG00000245975	AC090515.2	6.113573939	25.5368806	-2.050876823	0.001779623	0.007338832

ENSG00000198848	CES1	7.459775962	30.042612	-2.009164444	0.001781323	0.007343363
ENSG00000154102	C16orf74	23.60878164	4.097382834	2.507904326	0.001786427	0.007361066
ENSG00000206127	GOLGA8O	14.47315452	39.37015008	-1.448071816	0.001809919	0.007449036
ENSG00000123685	BATF3	15.53207897	1.719551266	3.155286981	0.001831815	0.007530233
ENSG00000164318	EGFLAM	1.302947796	13.78830737	-3.440978935	0.001833133	0.007532679
ENSG00000131797	CLUHP3	120.8255211	69.61317729	0.792697424	0.001841279	0.007563175
ENSG00000273888	FRMD6-AS1	114.4539324	66.76852333	0.777633267	0.001848072	0.007588087
ENSG00000223813	AC007255.1	1.205124193	12.96745755	-3.395921094	0.001850354	0.007595962
ENSG00000260593	AC009097.2	0.297870462	9.478436744	-4.820191105	0.001857732	0.007624748
ENSG00000203565	AL450313.1	7.825135883	0	5.336610577	0.001862051	0.007639466
ENSG00000163006	CCDC138	35.85315492	11.19633596	1.681603592	0.001865506	0.00765063
ENSG00000205609	EIF3CL	221.5396344	35.80437639	2.629515697	0.001874814	0.007684269
ENSG00000222032	AC112721.2	3.989975192	24.44176507	-2.651450992	0.001888411	0.007735437
ENSG00000235652	AL356599.1	51.54686957	22.02267724	1.224652077	0.001901581	0.007784797
ENSG00000196581	AJAP1	21.35702514	4.222401934	2.336295326	0.001902909	0.007788704
ENSG00000087589	CASS4	2.410248385	16.45607662	-2.745085035	0.00190439	0.007791704
ENSG00000064989	CALCRL	12.68960914	37.2781486	-1.55019	0.001924279	0.007863823
ENSG00000162777	DENND2D	42.03233452	16.09434222	1.38204951	0.001936093	0.007907452
ENSG00000102445	RUBCNL	12.66851769	38.01222675	-1.594679528	0.001939244	0.00791722
ENSG00000157111	TMEM171	118.9291595	68.83949493	0.787702298	0.001945307	0.00793731
ENSG00000147234	FRMPD3	1.979820524	15.70166562	-3.032954786	0.001950925	0.007957117
ENSG00000182389	CACNB4	11.53223315	36.75949787	-1.674792002	0.001957002	0.007980343
ENSG00000181804	SLC9A9	83.70056498	44.47683181	0.912114097	0.001958226	0.007983773
ENSG00000267009	AC007780.1	16.97808273	2.096555895	3.016331295	0.001974234	0.008041175
ENSG00000114547	ROPN1B	6.928475042	0	5.159813106	0.001983377	0.008075249
ENSG00000004777	ARHGAP33	45.03662457	88.58076658	-0.97472296	0.001983762	0.008075249
ENSG00000205300	AL356414.1	6.90496207	0	5.155561149	0.001990532	0.008100669
ENSG00000253414	AC124067.2	0	6.66376995	-5.273796194	0.001999625	0.008135059
ENSG00000132481	TRIM47	117.7955754	69.34192041	0.76634412	0.00200427	0.008150777
ENSG00000247095	MIR210HG	9.240271889	32.35165569	-1.818594354	0.002012186	0.008179776
ENSG00000279528	AC115618.3	27.77654659	60.60707238	-1.121078197	0.002017253	0.008198776
ENSG00000259370	AC103740.1	67.18494351	115.7963439	-0.787054555	0.002021405	0.008209931
ENSG00000108823	SGCA	14.00314983	37.59737643	-1.423235545	0.002069187	0.00840003
ENSG00000172183	ISG20	14.0266628	38.52786865	-1.458033305	0.002079694	0.008438089
ENSG00000235387	SPAAR	2.521714333	16.66515383	-2.734790546	0.00208693	0.008460528
ENSG00000233461	AL445524.1	29.40024988	9.087813942	1.690925538	0.002108722	0.008541678
ENSG00000168421	RHOH	12.08399759	0.723468803	4.096885969	0.002110022	0.008544174
ENSG00000178568	ERBB4	0.304691634	9.89834969	-4.881546298	0.002110938	0.008546226
ENSG00000184792	OSBP2	22.75672522	53.75349657	-1.241199296	0.002133099	0.008624223
ENSG00000187951	AC091057.1	62.40725758	28.10697357	1.153966665	0.002156143	0.008708945
ENSG00000175106	TVP23C	111.394096	64.7576776	0.780681973	0.002184007	0.008809547
ENSG00000101938	CHRDL1	1.60763943	14.55633654	-3.201690104	0.002196767	0.008852452
ENSG00000176595	KBTBD11	23.9822186	55.92184919	-1.215913964	0.002211314	0.008902473
ENSG00000156427	FGF18	0	6.902137305	-5.327324538	0.002218346	0.008929059
ENSG00000100629	CEP128	83.08678206	44.38976486	0.902239637	0.002241624	0.009014052
ENSG00000213316	LTC4S	3.983781955	21.81855619	-2.442308143	0.002246062	0.009025735
ENSG00000084734	GCKR	2.210201527	14.70062699	-2.744591355	0.002246624	0.009025735
ENSG00000148288	GBGT1	21.53795868	50.67943255	-1.236029903	0.002262328	0.009081543
ENSG00000179388	EGR3	1.60763943	13.65627831	-3.113371326	0.002288134	0.009176302
ENSG00000096654	ZNF184	141.6155304	78.69959719	0.850312355	0.002318519	0.009289223
ENSG00000157404	KIT	15.16671905	42.02690825	-1.475290241	0.002319674	0.009292066
ENSG00000285053	TBCE	36.83786307	12.94147152	1.509502119	0.002320662	0.009292452
ENSG00000104221	BRF2	61.87155701	113.2290469	-0.872492213	0.002324631	0.009306558
ENSG00000280077	AL353763.2	72.98628239	36.98696061	0.982707481	0.002334438	0.009338648
ENSG00000251179	TMEM92-AS1	48.44170646	19.94304363	1.276850223	0.002336903	0.009344924
ENSG00000188766	SPRED3	64.75620974	110.6356363	-0.770527096	0.002373289	0.009470444
ENSG00000004846	ABCB5	0	6.60724346	-5.263064525	0.002441469	0.00972948
ENSG00000159055	MIS18A	88.53640335	47.69614132	0.892433588	0.002453661	0.009770596
ENSG00000205683	DPF3	9.491744342	31.18724406	-1.706359993	0.002502367	0.009949343
ENSG00000183378	OVCH2	0.335025778	9.036243613	-4.75326203	0.002504687	0.009956669
ENSG00000227925	LINC01655	22.8197207	52.3116216	-1.197937671	0.002516805	0.009997215
ENSG00000178999	AURKB	91.55433575	50.86137988	0.849555573	0.002517904	0.009999675
ENSG00000130193	THEM6	36.74003946	73.41662131	-0.993900773	0.002538588	0.010077981
ENSG00000106123	EPHB6	68.17575057	32.43046236	1.072467099	0.002564691	0.010169989
ENSG00000237234	Z99289.1	3.743530325	18.71189792	-2.320424522	0.002566755	0.01017624
ENSG00000130775	THEMIS2	105.5696834	61.74920382	0.775174996	0.002567286	0.01017641

ENSG00000111679	PTPN6	6.56311512	0	5.083086628	0.002568419	0.010178966
ENSG00000121743	GJA3	7.135343072	30.50988122	-2.095762627	0.002570713	0.010186122
ENSG00000285641	AL358472.6	6.992915046	0	5.171900032	0.002575939	0.010204888
ENSG00000255203	OR7E2P	0.335025778	8.835828414	-4.717437531	0.002576465	0.010205034
ENSG00000259367	AC087286.2	2.758916507	16.45772867	-2.56413548	0.002616643	0.010350404
ENSG00000175643	RMI2	66.16308005	32.61355354	1.021583707	0.002617138	0.010350404
ENSG00000121653	MAPK8IP1	93.07562713	49.19699833	0.918892395	0.002619471	0.010357665
ENSG00000132141	CCT6B	67.59723505	31.17237558	1.116380579	0.002627388	0.010383064
ENSG00000214278	AC010442.1	6.71918549	0	5.121134307	0.002639985	0.010430868
ENSG00000104880	ARHGEF18	50.7395119	92.86835702	-0.871571954	0.002657353	0.010495513
ENSG00000257438	AC011595.1	1.821328633	15.00418285	-3.022027732	0.00267906	0.010571235
ENSG00000156968	MPV17L	16.46347361	2.818372645	2.550839071	0.002686052	0.010596817
ENSG00000280094	OR1B1	6.549472776	0	5.080444791	0.002701668	0.010656411
ENSG00000138483	CCDC54	1.540149971	13.31847615	-3.102228841	0.002738207	0.010782174
ENSG00000177191	B3GNT8	34.50937442	69.98317196	-1.015351425	0.002740163	0.010787838
ENSG00000228343	AC115618.2	46.29352342	85.16314075	-0.881140757	0.002741423	0.010790762
ENSG00000234156	AL162254.1	42.2756356	16.13394643	1.388802923	0.002745838	0.010804063
ENSG00000188015	S100A3	99.83942116	54.86068469	0.862499327	0.002784523	0.010937698
ENSG00000132874	SLC14A2	0.914074903	11.8496601	-3.67545486	0.002803702	0.011008887
ENSG00000261578	AP003119.3	40.25938202	15.3279651	1.38926649	0.002807384	0.01101648
ENSG00000197766	CFD	39.19982964	83.04745722	-1.086097118	0.002807749	0.01101648
ENSG00000238278	ALG1L6P	99.95277091	54.1540317	0.882177199	0.002832892	0.011113038
ENSG00000132570	PCBD2	50.49853801	89.93623438	-0.832014045	0.002851238	0.011182901
ENSG00000230018	PPIAP39	0	6.373832266	-5.207905385	0.002879262	0.011284324
ENSG00000165475	CRYL1	53.27710144	97.17481706	-0.865378305	0.002884084	0.011298976
ENSG00000101187	SLCO4A1	9.231029196	0.351420336	4.616454602	0.002898835	0.011354631
ENSG00000276649	AL117335.1	11.25410395	33.03386823	-1.554556216	0.002915711	0.011414301
ENSG00000220494	YAP1P1	1.526507626	14.16240963	-3.195332847	0.002963044	0.011580032
ENSG00000103522	IL21R	13.00049401	36.04399406	-1.480754461	0.002973257	0.011613415
ENSG00000276223	AL118522.1	0.304691634	8.463779946	-4.655798525	0.002980699	0.011640304
ENSG00000272654	AL358472.2	33.95940357	11.22192025	1.595509033	0.003037322	0.011850329
ENSG00000159214	CCDC24	34.03739159	66.749949	-0.971895419	0.003038143	0.011851317
ENSG00000181274	FRAT2	112.2610506	63.36921381	0.822099267	0.003051379	0.011898496
ENSG00000153132	CLGN	48.63628234	21.69849325	1.163338469	0.003061127	0.011932043
ENSG00000258231	AC020637.1	0	6.235194992	-5.180237497	0.003084614	0.012007881
ENSG00000274276	CBSL	1.309768968	12.76498856	-3.324859443	0.003090972	0.012028139
ENSG00000273319	AC058791.1	26.51317563	6.447682781	2.051999555	0.00311445	0.012099178
ENSG00000005471	ABCB4	31.44343908	67.57810404	-1.099455345	0.003124292	0.012135151
ENSG00000234869	AL021392.1	0.297870462	8.878736731	-4.723657087	0.003151067	0.012225485
ENSG00000262185	AC005736.1	10.43175374	33.44802153	-1.687015197	0.003164339	0.012270127
ENSG00000229848	AC139149.1	8.093394463	28.30944256	-1.80630604	0.00316664	0.012276504
ENSG00000188396	TCTEX1D4	12.65864706	35.31927495	-1.489018085	0.003221268	0.012472321
ENSG00000188747	NOXA1	9.271233967	29.49738468	-1.664464927	0.003257509	0.012598602
ENSG00000271204	AC016831.4	19.83724436	3.83508324	2.365038539	0.003269074	0.012640984
ENSG00000100206	DMC1	18.24836919	3.452720706	2.392529046	0.003297282	0.012738247
ENSG00000236753	MKLN1-AS	60.26938856	27.90285252	1.114035907	0.003313567	0.012796421
ENSG00000255201	AC087623.1	37.79833598	13.24998354	1.508874465	0.003343276	0.01288728
ENSG00000188916	INSYN2	14.93696598	38.33281135	-1.357987889	0.00342043	0.0131555
ENSG00000003249	DBNDD1	10.37108545	30.90471808	-1.579292075	0.003445741	0.013238197
ENSG00000107551	RASSF4	94.7451005	52.40764583	0.851014565	0.003462075	0.013296052
ENSG00000258947	TUBB3	7.944773199	28.17040355	-1.817030021	0.003511594	0.013468862
ENSG00000104177	MYEF2	15.0552531	40.04935379	-1.416239627	0.00351959	0.013492086
ENSG00000215845	TSTD1	0.335025778	8.103697598	-4.593270089	0.00354801	0.013591036
ENSG00000111665	CDCA3	103.9558507	58.83976311	0.824974282	0.00358205	0.013713873
ENSG00000142856	ITGB3BP	76.94799589	39.80738705	0.95315189	0.003702888	0.014147923
ENSG00000111261	MANSC1	18.88431488	43.84453754	-1.211456167	0.003762762	0.014360898
ENSG00000208005	MIR503	0.63289624	10.18167916	-4.02336009	0.003833455	0.01460664
ENSG00000061918	GUCY1B1	34.24425962	67.04804048	-0.969960503	0.003837772	0.014620416
ENSG00000260750	AC092720.1	20.3866816	46.48466871	-1.193107861	0.003881296	0.014772732
ENSG00000273129	PACERR	6.644874858	0	5.106445579	0.003898506	0.014830115
ENSG00000163449	TMEM169	3.62219375	18.64175325	-2.341642	0.003899539	0.014830474
ENSG00000237807	AC022034.1	3.625243205	19.08229433	-2.374570608	0.003900023	0.014830474
ENSG00000282164	PEG13	11.0745206	33.34432757	-1.599945216	0.003905418	0.014848281
ENSG00000163624	CDS1	2.126020267	15.11352998	-2.809577327	0.003937775	0.014957662
ENSG00000177614	PGBD5	18.87130047	3.121928502	2.592614645	0.003938877	0.014959123
ENSG00000186088	GSAP	92.29285376	50.89386767	0.85610328	0.003942331	0.014969514

ENSG00000184374	COLEC10	17.43651754	2.775464328	2.647109358	0.003953056	0.015002043
ENSG00000231574	LINC02015	0.297870462	8.062441335	-4.587053231	0.003955129	0.015007176
ENSG00000253559	OSGEPL1-AS1	0	6.001783799	-5.121705998	0.003993985	0.015131512
ENSG00000185904	LINC00839	31.08597166	10.57230196	1.557088667	0.003994429	0.015131512
ENSG00000273381	AL158071.4	8.70205547	0.361734402	4.529255951	0.004040952	0.015293857
ENSG00000254951	AC044810.2	1.19830302	12.1206217	-3.306494655	0.004053747	0.015336713
ENSG00000101695	RNF125	1.211945365	11.9127948	-3.272116551	0.004077555	0.015414612
ENSG00000223403	MEG9	40.99063547	80.58351375	-0.971023009	0.004115988	0.015546804
ENSG00000285737	AL138920.1	11.25787566	1.042294888	3.4233618	0.004121077	0.015563204
ENSG00000147804	SLC39A4	50.77459476	88.87866926	-0.809587437	0.00413349	0.015601606
ENSG00000260941	LINC00622	8.384443753	0.339454216	4.475928779	0.004147384	0.015651216
ENSG00000158714	SLAMF8	13.10576672	1.405681343	3.222345063	0.004153143	0.015670112
ENSG00000273213	AC239798.4	0	6.192286675	-5.171275668	0.004170935	0.015728704
ENSG00000065361	ERBB3	6.576757464	24.2977699	-1.89035427	0.004197543	0.015820459
ENSG00000130590	SAMD10	23.77535057	51.4664378	-1.109954791	0.004208148	0.015854695
ENSG00000256325	AC025423.1	15.43425537	2.78412634	2.462487658	0.004231057	0.015926618
ENSG00000175305	CCNE2	34.7943248	11.86658238	1.552215087	0.004237003	0.015943243
ENSG00000165682	CLEC1B	2.112377923	14.42430748	-2.746086482	0.004248336	0.015980121
ENSG00000161692	DBF4B	96.66453161	53.01681134	0.865140209	0.004261119	0.016022421
ENSG00000255031	AP002807.1	36.27182835	13.44554904	1.435889966	0.004263399	0.016028104
ENSG00000132321	IQCA1	12.06290614	33.43315304	-1.47933638	0.004264973	0.016031132
ENSG00000177875	CCDC184	19.06569181	3.150816903	2.592884047	0.004273711	0.016058186
ENSG00000134830	C5AR2	0.297870462	8.472040215	-4.657280401	0.00429455	0.016127768
ENSG00000164078	MST1R	19.80898268	47.88043773	-1.270773587	0.004336256	0.01626974
ENSG00000256001	AC079949.1	6.163649337	0	4.994914538	0.004345703	0.016296385
ENSG00000158528	PPP1R9A	11.51859081	31.25078051	-1.438741771	0.004364169	0.01635975
ENSG00000158825	CDA	18.81242577	44.0673394	-1.229398558	0.004375645	0.016399821
ENSG00000161544	CYGB	34.82088723	12.2745292	1.507012241	0.004378659	0.016408168
ENSG00000279838	AL356273.3	6.079468078	23.09386053	-1.91101417	0.004390613	0.01644705
ENSG00000146700	SSC4D	9.084829453	29.47004187	-1.702832348	0.004419229	0.016540043
ENSG00000099812	MISP	0.304691634	7.709368946	-4.522504182	0.004488219	0.016782527
ENSG00000198056	PRIM1	38.41184003	12.90927901	1.567397204	0.004498917	0.016813481
ENSG00000283627	AL137785.1	11.96131082	1.085203205	3.489239432	0.004537596	0.016951957
ENSG00000233581	AC069155.1	1.205124193	11.2256261	-3.188754481	0.004558285	0.017017053
ENSG00000268621	IGFL2-AS1	0	5.659025475	-5.036399659	0.00457004	0.017057883
ENSG00000035499	DEPDC1B	22.73698396	5.319571259	2.102693744	0.004589482	0.017122702
ENSG00000281383	FP671120.4	28.9961999	5.275010888	2.454222249	0.004589869	0.017122702
ENSG00000116883	AL591845.1	18.58886594	44.4809394	-1.264061951	0.004638414	0.017291425
ENSG00000236049	LINC01920	6.360646741	0	5.043042198	0.004648236	0.01731875
ENSG00000203644	AC083799.1	62.47034739	31.56259664	0.98584072	0.004704412	0.017509281
ENSG00000276116	FUT8-AS1	16.22322198	3.192073166	2.353808084	0.004723545	0.017561681
ENSG00000263293	THCAT158	11.12217448	0.702840672	3.987601571	0.004731022	0.017580075
ENSG00000267321	LINC02001	57.87341045	28.6588091	1.012774765	0.004739698	0.017606042
ENSG00000186481	ANKRD20A5P	14.98471419	36.87721174	-1.299505076	0.004776095	0.017719142
ENSG00000281883	AL512506.3	12.03015047	1.381749104	3.110905432	0.004801947	0.017799216
ENSG00000228242	AC093495.1	56.83664878	27.33854265	1.05513824	0.004836534	0.017924232
ENSG00000179869	ABCA13	9.138676568	29.70576488	-1.704362023	0.004844559	0.017950782
ENSG00000179082	C9orf106	1.540149971	11.60097868	-2.902121911	0.004886107	0.018098299
ENSG00000255495	AC145124.1	11.30930126	32.53680066	-1.514867495	0.004893436	0.018122226
ENSG00000136231	IGF2BP3	95.86497211	49.00433518	0.965325636	0.004920976	0.018211279
ENSG00000253194	AL137009.1	20.51106764	5.251078649	1.96162295	0.004945569	0.018289307
ENSG00000136842	TMOD1	18.22350602	3.55050346	2.366719402	0.004973126	0.018384695
ENSG00000227107	AC104667.1	2.552048477	14.9064001	-2.565316811	0.004978261	0.018400415
ENSG00000281026	N4BP2L2-IT2	50.04692437	23.2345516	1.108108148	0.005034786	0.018596154
ENSG00000203760	CENPW	69.03176325	36.4659608	0.922719639	0.005080298	0.018737699
ENSG00000258955	LINC00519	21.21720318	5.276662942	2.012625066	0.005093975	0.018784822
ENSG00000100767	PAPLN	43.26842079	77.66130343	-0.842534754	0.005150506	0.018983215
ENSG00000074527	NTN4	46.61078607	85.35775122	-0.869476543	0.005168689	0.019040134
ENSG00000180828	BHLHE22	5.825574104	0	4.914635368	0.005186723	0.019093074
ENSG00000204758	AC008429.1	8.177575722	25.77194385	-1.663005583	0.005214422	0.019188261
ENSG00000167077	MEI1	4.085377275	17.47278721	-2.102996212	0.005222344	0.019214023
ENSG00000187672	ERC2	12.88050763	1.369782984	3.2161941	0.00525181	0.019315617
ENSG00000239590	OR1J4	21.14396388	3.968764352	2.428348816	0.005253428	0.01931816
ENSG00000250132	AC004803.1	62.55830036	31.35682354	0.9990839	0.005314885	0.019530377
ENSG00000272734	ADIRF-AS1	48.95291706	89.91475768	-0.874365729	0.005349453	0.019643555
ENSG00000044524	EPHA3	6.292529346	23.66507389	-1.917679895	0.005368746	0.01970052

ENSG00000243364	EFNA4	65.44358103	32.50380467	1.01057173	0.005370915	0.019705012
ENSG00000169126	ARMC4	31.53676871	11.30443278	1.486438669	0.005407542	0.019828922
ENSG00000125454	SLC25A19	35.77283971	71.93708945	-1.003428792	0.005414066	0.019849356
ENSG00000269843	AC008537.2	60.68787333	29.37526795	1.044645028	0.005419444	0.019862087
ENSG00000225828	FAM229A	39.73480795	16.84504737	1.239700754	0.00545188	0.019963417
ENSG00000261485	PAN3-AS1	29.25027842	9.833562931	1.571389842	0.005513602	0.020176322
ENSG00000151418	ATP6V1G3	0.304691634	7.820769873	-4.540403276	0.005513894	0.020176322
ENSG00000231789	PIK3CD-AS2	7.581834798	24.23228612	-1.683753838	0.005531259	0.020232763
ENSG00000188092	GPR89B	48.73922786	90.89197053	-0.896432917	0.005556295	0.020320778
ENSG00000259091	LINC00517	19.56800878	4.880682235	2.000276874	0.0055625	0.020336335
ENSG00000182621	PLCB1	27.27862926	57.50462817	-1.07183212	0.00557712	0.020379065
ENSG00000100024	UPB1	5.662682562	0	4.868971694	0.005597232	0.020438228
ENSG00000274204	AL138689.1	1.546971143	11.57003648	-2.896403691	0.005624514	0.020530659
ENSG00000175426	PCSK1	15.01809779	38.65619186	-1.36527822	0.005710385	0.020806545
ENSG00000115556	PLCD4	77.45873897	41.84000475	0.891309905	0.005711072	0.020806545
ENSG00000159961	OR3A3	5.852858793	0	4.92048802	0.005723021	0.020839153
ENSG00000200033	RNU6-403P	8.726290703	26.80733525	-1.617387802	0.005761431	0.02097169
ENSG00000235890	TSPEAR-AS1	7.76141814	0.351420336	4.364554463	0.005778692	0.021027178
ENSG00000277763	AL138995.1	5.828623559	0	4.915080049	0.005803673	0.021103345
ENSG00000285637	AL161449.2	5.927169424	0	4.936499837	0.005832575	0.021197349
ENSG00000279243	AP000769.3	0.304691634	7.803847592	-4.537707028	0.005925652	0.021507719
ENSG00000172020	GAP43	0.595740924	9.43222432	-3.939483109	0.005943618	0.021559576
ENSG00000137868	STRA6	0	5.849528351	-5.08896447	0.005978904	0.021664953
ENSG00000228782	MRPL45P2	60.43847334	31.19755813	0.952515475	0.005992395	0.021706294
ENSG00000149634	SPATA25	29.50866637	10.14537906	1.537256212	0.006014685	0.021775689
ENSG00000284713	AP003071.5	7.825858145	0.339454216	4.374867463	0.006045209	0.021874801
ENSG00000146054	TRIM7	8.025905003	27.03898792	-1.749643516	0.006224169	0.022456121
ENSG00000275183	LENG9	52.69204773	92.23606275	-0.807718071	0.006237232	0.022495466
ENSG00000235098	ANKRD65	10.81443339	30.54133163	-1.492908511	0.006280837	0.022640983
ENSG00000152760	TCTEX1D1	40.2402687	14.25648653	1.505430055	0.00629716	0.022684136
ENSG00000224397	SMIM25	63.90352539	33.63892615	0.926879217	0.006308531	0.022716361
ENSG00000176399	DMRTA1	0.63289624	9.346809429	-3.902365838	0.006330575	0.022784826
ENSG00000183778	B3GALT5	30.55215489	10.88451983	1.492315102	0.006334654	0.022795572
ENSG00000139044	B4GALNT3	4.643962883	18.5460243	-1.993834828	0.006387633	0.022970359
ENSG00000268041	AC010616.1	0.304691634	7.303074173	-4.445996852	0.006403166	0.023018279
ENSG00000100162	CENPM	48.48461162	22.2952909	1.118341326	0.006414147	0.023049805
ENSG00000221091	MIR1302-5	5.774148509	0	4.89396584	0.006436183	0.023117039
ENSG00000250208	FZD10-DT	7.93050292	0.351420336	4.391598135	0.006456534	0.023178156
ENSG00000196689	TRPV1	70.96490689	34.99108983	1.022605091	0.006463425	0.023194908
ENSG00000183397	C19orf71	34.23061728	64.61037831	-0.916929127	0.006483308	0.023258253
ENSG00000144893	MED12L	1.218766537	12.04482386	-3.284300583	0.00648716	0.023268068
ENSG00000265666	RARA-AS1	8.133599234	24.62496272	-1.599323948	0.006495594	0.023290308
ENSG00000204634	TBC1D8	33.98722187	65.15200625	-0.935203653	0.006510066	0.023334172
ENSG00000133985	TTC9	4.587066312	20.62690822	-2.158323845	0.006530948	0.023404997
ENSG00000131969	ABHD12B	1.83119926	12.71547202	-2.777934323	0.006541515	0.023428035
ENSG00000144031	ANKRD53	48.54527991	23.46425695	1.048733404	0.006545549	0.02343718
ENSG00000283267	FAM237B	7.875933544	0.361734402	4.382795797	0.006572848	0.023518771
ENSG00000169908	TM4SF1	5.090454609	19.51116456	-1.952688726	0.006595247	0.023586777
ENSG00000143217	NECTIN4	3.238791832	16.53488329	-2.375932887	0.006663295	0.023793406
ENSG00000278784	AL136295.7	37.48745111	15.20966068	1.307579279	0.006706154	0.023938248
ENSG00000200769	RF00019	5.834816797	0	4.907509734	0.006730562	0.024010849
ENSG00000258101	AC010173.1	7.673465164	0.361734402	4.350287549	0.00674154	0.02404398
ENSG00000167889	MGAT5B	50.57284864	23.45599668	1.107345288	0.006767459	0.024128166
ENSG00000158406	HIST1H4H	69.82799442	36.36777631	0.938786529	0.00683642	0.024340736
ENSG00000278998	AC099552.4	2.135890895	13.0000518	-2.588893699	0.006899206	0.024534957
ENSG00000100902	PSMA6	75.22439653	42.05609192	0.839445148	0.006907714	0.024556834
ENSG00000266043	MIR3649	2.105556751	13.56385346	-2.663101415	0.006936525	0.024638219
ENSG00000189221	MAOA	3.757172669	16.0856802	-2.101786603	0.006937697	0.024638219
ENSG00000280339	AP001528.3	0.97474319	9.77703644	-3.365026784	0.007034756	0.024931949
ENSG00000127533	F2RL3	19.04585623	4.615078533	2.052740802	0.007053868	0.024986941
ENSG00000160298	C21orf58	104.4116795	61.98437354	0.754153513	0.007126558	0.025227978
ENSG00000235852	AC005540.1	17.31930175	42.47350425	-1.298381351	0.007141968	0.025256112
ENSG00000171505	OR1N1	5.751263472	0	4.897925028	0.007219454	0.025499849
ENSG00000230454	U73166.1	27.02033564	8.858108599	1.613093532	0.007239671	0.025566927
ENSG00000198546	ZNF511	84.86350627	49.85663541	0.766620747	0.00726888	0.025658016
ENSG00000275713	HIST1H2BH	11.51554135	1.424657421	3.028802749	0.007269156	0.025658016

ENSG00000169247	SH3TC2	1.838020433	11.8496601	-2.67620715	0.007290439	0.025720076
ENSG00000178772	CPN2	27.33759829	8.479050173	1.696068663	0.007293653	0.025727061
ENSG00000117600	PLPPR4	7.886526432	25.10129569	-1.6781852	0.007296842	0.025733959
ENSG00000234617	SNRK-AS1	12.5100258	2.105217907	2.570758379	0.007309538	0.025765659
ENSG00000183801	OLFML1	46.57837947	21.10334766	1.144629721	0.00732697	0.02582274
ENSG00000225231	LINC02470	0.63289624	9.007355212	-3.848809143	0.007347095	0.025876171
ENSG00000241684	ADAMTS9-AS2	27.43029998	9.550635205	1.527235402	0.007354113	0.025893662
ENSG00000239739	AC026316.2	5.490548326	0	4.830022851	0.007416817	0.026099686
ENSG00000254192	AC011365.2	0.90725373	12.59881966	-3.76828756	0.007494596	0.026360042
ENSG00000166106	ADAMTS15	0.297870462	7.243243575	-4.435617569	0.007571156	0.026602395
ENSG00000167513	CDT1	69.08561036	37.62831863	0.879724719	0.007599251	0.026683122
ENSG00000233143	DIRC3-AS1	0	5.184238092	-4.914390479	0.007657767	0.026867242
ENSG00000112541	PDE10A	12.55400229	2.094903841	2.578032609	0.00765813	0.026867242
ENSG00000253767	PCDHGA8	7.751547513	23.85157564	-1.618233979	0.007677074	0.026915588
ENSG00000219626	FAM228B	38.58048142	69.63545747	-0.851699072	0.007702085	0.026994198
ENSG00000249341	AC124017.1	5.511011842	0	4.834703793	0.007735393	0.027106379
ENSG00000147394	ZNF185	16.46652307	41.31775464	-1.326231836	0.007762003	0.027190486
ENSG00000176692	FOXC2	0.914074903	10.70102691	-3.526226856	0.007862669	0.02750615
ENSG00000129354	AP1M2	0.914074903	10.10452454	-3.447035657	0.007876535	0.027540793
ENSG00000283930	AL117339.5	16.51427127	38.78036239	-1.229926313	0.007913747	0.027650939
ENSG00000218510	LINC00339	38.7815996	16.46804274	1.232301454	0.007920207	0.027665659
ENSG00000149633	KIAA1755	14.48984632	36.50476153	-1.337788972	0.00799609	0.027912017
ENSG00000280414	AC018470.1	13.61669845	2.434358058	2.473859973	0.008041818	0.028052853
ENSG00000237515	SHISA9	1.249100681	11.02150505	-3.139721399	0.008063324	0.028123169
ENSG00000238072	AC009244.1	70.95943592	39.44525091	0.849691916	0.008073112	0.028152599
ENSG00000124406	ATP8A1	46.18520125	21.76368176	1.08390288	0.008103423	0.028248851
ENSG00000144331	ZNF385B	0	5.182586039	-4.913903636	0.008140308	0.028367946
ENSG00000278266	AC079949.2	15.11045042	3.159478915	2.261134542	0.008189268	0.028519499
ENSG00000085871	MGST2	48.25495288	84.86379895	-0.812724599	0.008200697	0.028554531
ENSG00000260517	AC009093.2	82.14335001	43.76748939	0.90954177	0.008204647	0.028563516
ENSG00000238832	RF01233	3.110634085	14.39376703	-2.208336958	0.008239493	0.028670467
ENSG00000151117	TMEM86A	56.74941806	28.53845089	0.993854345	0.008297177	0.028842304
ENSG00000183840	GPR39	16.60590164	3.510899251	2.245339669	0.0083415	0.028977053
ENSG00000196656	AC004057.1	111.4170512	60.6520345	0.873806403	0.00835178	0.029007933
ENSG00000279312	AL136164.4	16.21335135	37.16509562	-1.196907974	0.008362711	0.029036223
ENSG00000246985	SOCS2-AS1	13.13610087	2.149778278	2.627099009	0.008365584	0.029041362
ENSG00000153094	BCL2L11	75.20170015	41.64854684	0.851457149	0.008396559	0.029144042
ENSG00000081853	PCDHGA2	45.78941286	78.62148753	-0.779288892	0.008401501	0.02915634
ENSG00000257221	AC007569.1	0.639717412	8.400243498	-3.74129021	0.008416032	0.02919705
ENSG00000165244	ZNF367	66.10385629	34.97075697	0.918556675	0.008424016	0.029219885
ENSG00000238273	AC108058.1	27.51825296	53.3968248	-0.95731083	0.008435128	0.029248699
ENSG00000163485	ADORA1	13.92883919	2.420739884	2.515887697	0.008460126	0.029325623
ENSG00000136828	RALGPS1	16.77193696	37.94549266	-1.175348704	0.008466131	0.029341562
ENSG00000231672	DIRC3	5.7444423	21.6060684	-1.895581958	0.008482681	0.029389149
ENSG00000198225	FKBP1C	35.41295076	65.52941262	-0.886423745	0.008520246	0.029508269
ENSG00000161914	ZNF653	27.78336776	57.08450229	-1.036688788	0.008546509	0.029585708
ENSG00000204253	HNRNPCP2	49.00639098	21.84248843	1.166167118	0.008567142	0.029642368
ENSG00000271709	AC017033.1	0	4.956184803	-4.844877943	0.008607663	0.029767751
ENSG00000172733	PURG	12.61772003	1.788043876	2.831970072	0.008615184	0.02978882
ENSG00000170175	CHRNB1	25.80974047	51.69449109	-1.00206179	0.008666337	0.029940865
ENSG00000168952	STXBP6	29.56009197	11.1913798	1.400337789	0.008840423	0.030506921
ENSG00000205002	AARD	4.93878389	19.09961836	-1.943186183	0.008888459	0.030647323
ENSG00000177675	CD163L1	46.74504273	22.35677355	1.064117929	0.008907096	0.030706508
ENSG00000283959	AP002851.1	27.85705045	9.254089306	1.596989205	0.00892945	0.030778481
ENSG00000248491	AC093772.1	5.300372095	0	4.77517865	0.008936113	0.030796356
ENSG00000242110	AMACR	27.22343195	53.3262784	-0.971689316	0.008941072	0.030808148
ENSG00000165807	PPP1R36	13.15351493	32.80075231	-1.321959476	0.008952596	0.030832775
ENSG00000250133	HOXC-AS2	53.03631621	89.05144637	-0.750476624	0.008981324	0.030916396
ENSG00000269069	AC007842.1	24.68637602	8.033552935	1.619903317	0.009009251	0.030997174
ENSG00000188985	DHFRP1	94.78728341	55.06014485	0.781165877	0.009020341	0.031025091
ENSG00000156787	TBC1D31	96.98673163	55.78372013	0.798134751	0.009032325	0.03104071
ENSG00000213214	ARHGEF35	17.80734844	41.6946528	-1.230657014	0.009059203	0.031122817
ENSG00000172264	MACROD2	29.55704251	57.16136164	-0.95056161	0.009094282	0.031209302
ENSG00000213057	C1orf220	32.42365325	12.90557316	1.326131098	0.009094854	0.031209302
ENSG00000259847	LINC02126	0.297870462	6.622513687	-4.304441084	0.009101637	0.031227435
ENSG00000250155	AC008957.1	27.41665764	10.1746692	1.430561766	0.009139791	0.031337715

ENSG00000091262	ABCC6	18.29099548	4.518947833	2.00881471	0.009202568	0.031542586
ENSG00000139410	SDSL	24.07636481	48.82135048	-1.020790597	0.009287327	0.031806958
ENSG00000210174	MT-TR	31.67416915	12.35869378	1.360024619	0.009294257	0.031820241
ENSG00000036672	USP2	4.464379541	17.65598486	-1.99585552	0.009343133	0.031961332
ENSG00000204314	PRRT1	49.69646266	23.40277429	1.087112291	0.009373872	0.032045456
ENSG00000001626	CFTR	7.047390096	0.361734402	4.227921368	0.009537948	0.032505103
ENSG00000172554	SNTG2	23.73451786	7.001572113	1.760079826	0.009543498	0.032513386
ENSG00000144485	HES6	7.893975539	25.38763399	-1.67039821	0.009563559	0.032565771
ENSG00000136122	BORA	56.37481545	29.52957719	0.936001809	0.009595377	0.032658115
ENSG00000285051	AC026316.4	7.199783077	0.361734402	4.254636443	0.009660464	0.032863546
ENSG00000283498	MIR1244-2	24.94134132	8.105349652	1.621403048	0.009668183	0.032884441
ENSG00000243742	RPLP0P2	27.37268115	9.754756255	1.485457447	0.009688048	0.032934441
ENSG00000185666	SYN3	0.914074903	10.34784805	-3.484035709	0.009689202	0.032934441
ENSG00000128917	DLL4	7.558949761	0.339454216	4.330817024	0.009716731	0.033006492
ENSG00000267052	AC005050.1	0	4.892648355	-4.828822627	0.009765937	0.033168232
ENSG00000230596	GPAA1P2	11.12899565	1.381749104	2.998769401	0.009772609	0.033185487
ENSG00000008118	CAMK1G	5.090454609	0	4.713266113	0.009803669	0.033274704
ENSG00000163145	C1QTNF7	0.602562096	8.057485174	-3.706600477	0.009865242	0.0334619
ENSG00000176401	EID2B	45.19062248	22.12872027	1.031082829	0.0098759	0.03348171
ENSG00000265100	AC005332.1	5.172214348	0	4.744233361	0.009910758	0.033583504
ENSG00000104783	KCNN4	0	4.865412008	-4.821770504	0.009943244	0.033683974
ENSG00000127399	LRRC61	73.29097402	41.95375475	0.803312931	0.009966473	0.033750359
ENSG00000173947	PIFO	2.417069557	13.28793569	-2.43713731	0.010006334	0.033874342
ENSG00000185669	SNAI3	10.49242202	28.93112749	-1.471646123	0.010032177	0.033946798
ENSG00000101144	BMP7	4.999452178	0	4.690553289	0.010074351	0.034082463
ENSG00000258376	AC004846.1	10.83794636	29.73905615	-1.453614186	0.010186125	0.03442151
ENSG00000285018	AC245140.3	5.157944069	18.93829915	-1.896121149	0.010240095	0.034592674
ENSG00000228192	AL512353.1	14.84219184	3.169792981	2.232111908	0.010299664	0.034760115
ENSG00000197768	STPG3	9.422904686	1.052608954	3.161047392	0.010342333	0.034881533
ENSG00000160190	SLC37A1	4.390068909	16.43544849	-1.909156122	0.010370747	0.034960401
ENSG00000128262	POM121L9P	2.210201527	11.90453453	-2.439338336	0.010412028	0.035082544
ENSG00000164045	CDC25A	55.94888156	28.20960601	0.985571833	0.01041762	0.035095714
ENSG00000260193	AL138781.1	25.92362794	7.65820036	1.752985814	0.010436528	0.035153733
ENSG00000223695	FO393418.1	0.914074903	9.492456661	-3.355359646	0.01048318	0.035288071
ENSG00000224687	RASAL2-AS1	24.93587034	49.25818571	-0.981040131	0.010519858	0.035400102
ENSG00000217236	SP9	76.71060506	40.16776467	0.933569286	0.010678094	0.035868899
ENSG00000253485	PCDHGA5	30.87838137	59.10536681	-0.940405604	0.01069609	0.035911991
ENSG00000234141	AC006042.4	0	4.832817757	-4.813324194	0.010699699	0.035912543
ENSG00000248971	KRT8P46	4.792584147	17.57717818	-1.889468689	0.010705796	0.035921444
ENSG00000162373	BEND5	6.810187922	0.351420336	4.176473146	0.010729677	0.035994787
ENSG00000278934	AC117489.1	27.94123171	8.484006335	1.722105258	0.010806204	0.036223369
ENSG00000120949	TNFRSF8	11.45487307	1.776077757	2.701247872	0.010841731	0.03632494
ENSG00000127564	PKMYT1	74.235034	38.56222142	0.949748757	0.010872991	0.036417969
ENSG00000124191	TOX2	44.97567741	19.75419281	1.186143047	0.010946635	0.036623451
ENSG00000152939	MARVELD2	11.96266101	31.04695474	-1.369148498	0.011017242	0.036818319
ENSG00000188501	LCTL	42.38845175	19.87455102	1.089933699	0.011024394	0.036830415
ENSG00000232702	AL158050.1	6.857936127	0.339454216	4.185246385	0.011070038	0.036957161
ENSG00000115353	TACR1	2.528535505	13.60180561	-2.437601218	0.011123205	0.037118891
ENSG00000021645	NRXN3	0.900432558	9.828606769	-3.415938007	0.011183258	0.037277528
ENSG00000164087	POC1A	79.62273114	44.46897328	0.840425111	0.011209032	0.037357466
ENSG00000100399	CHADL	6.884498554	21.29920844	-1.632866956	0.011284826	0.03757563
ENSG00000105011	ASF1B	75.07810246	40.73817454	0.880293346	0.011322917	0.0376768
ENSG00000081181	ARG2	29.19948076	9.948267965	1.558398955	0.011414992	0.037964998
ENSG00000129195	PIMREG	67.79423246	35.77548799	0.922891802	0.011428948	0.038005353
ENSG00000164683	HEY1	10.65289205	1.39206317	2.934661356	0.011435165	0.0380139
ENSG00000170689	HOXB9	39.71506669	69.64206569	-0.808756363	0.011438722	0.038019663
ENSG00000153234	NR4A2	49.98858328	23.84827153	1.067207709	0.011451353	0.038049514
ENSG00000013588	GPRC5A	4.620449912	17.3733524	-1.90570317	0.011474372	0.038119927
ENSG00000139200	PIANP	54.39678851	28.42704996	0.935832659	0.011584691	0.038453306
ENSG00000137463	MGARP	24.4100404	51.06480392	-1.070683779	0.01162041	0.038562079
ENSG00000231160	KLF3-AS1	31.40358338	13.01532203	1.270440835	0.01163693	0.038605756
ENSG00000163995	ABLIM2	0.304691634	6.890171185	-4.363393222	0.011732438	0.038887809
ENSG00000254343	AC091563.1	3.090170569	15.29371879	-2.303857549	0.01179386	0.039081689
ENSG00000261150	EPPK1	0	4.582484282	-4.732593853	0.011845062	0.039232651
ENSG00000258976	AC013451.2	17.63826366	4.889344247	1.850213363	0.01184965	0.039241615
ENSG00000285441	SOD2	9.863925436	1.018362649	3.24952123	0.011859024	0.03926642

ENSG00000215493	AC007731.2	36.27729933	15.83369468	1.194706735	0.011925445	0.039480078
ENSG00000257894	AC027288.3	0.670051556	8.103697598	-3.664509687	0.011929426	0.039486986
ENSG00000168237	GLYCTK	53.0877418	90.32109753	-0.766746143	0.012024038	0.039774899
ENSG00000213793	ZNF888	60.43237442	32.61520559	0.888606889	0.012047631	0.039840303
ENSG00000131153	GINS2	51.11455379	24.32140686	1.076509489	0.012054152	0.039855546
ENSG00000119514	GALNT12	1.942665208	11.20499797	-2.557020038	0.012061287	0.039872352
ENSG00000278330	AC018529.2	0	4.551542085	-4.724206015	0.012099988	0.039975399
ENSG00000134470	IL15RA	59.3087311	29.57248551	1.001797311	0.012164499	0.040175797
ENSG00000270124	AC092127.2	12.54718112	2.478918429	2.34554885	0.012235674	0.040391674
ENSG00000275226	LINC00547	6.743420723	24.33366825	-1.844841664	0.012256916	0.040455388
ENSG00000040608	RTN4R	0.639717412	7.685436706	-3.612880955	0.012404947	0.040892196
ENSG00000163633	C4orf36	0	4.563508204	-4.727486769	0.012486672	0.041122589
ENSG00000136856	SLC2A8	15.1544269	34.76538561	-1.193423103	0.012554427	0.041339198
ENSG00000226017	PRICKLE2-AS3	13.08288169	2.466952309	2.415732888	0.012574757	0.041399602
ENSG00000224228	AL031599.1	0.967922018	8.859760653	-3.22225675	0.012584163	0.041424029
ENSG00000266127	ZNF415P1	6.488804488	0.351420336	4.107182721	0.012604841	0.041485547
ENSG00000127954	STEAP4	0.97474319	8.717417528	-3.198588773	0.012634061	0.041568592
ENSG00000215068	AC025171.2	38.40394753	66.22689539	-0.787153873	0.012664182	0.041654549
ENSG00000163354	DCST2	3.767043297	21.15055237	-2.495645021	0.012682142	0.041693893
ENSG00000224057	EGFR-AS1	15.03551185	3.121928502	2.259880986	0.012684307	0.041694434
ENSG00000154153	RETREG1	19.41803732	42.48496216	-1.133490775	0.012752365	0.041911541
ENSG00000278727	AC000403.1	10.59769473	27.64870672	-1.384018986	0.01277091	0.041965875
ENSG00000174292	TNK1	18.75615714	4.577126378	2.039690582	0.012798428	0.042048672
ENSG00000131944	FAAP24	29.42511305	55.11968018	-0.902116259	0.01280014	0.042048672
ENSG00000055163	CYFIP2	34.93477469	15.77882024	1.146498	0.012802428	0.042049564
ENSG00000158402	CDC25C	31.18200168	12.69649595	1.301943442	0.012811637	0.042073183
ENSG00000277948	RF02271	4.725094687	0	4.607587388	0.012831453	0.042131623
ENSG00000271265	AL355297.3	6.684451695	0.361734402	4.144126373	0.012841015	0.042149741
ENSG00000171931	FBXW10	8.401135553	0.702840672	3.581685214	0.012856959	0.042182154
ENSG00000268658	LINC00664	7.612796876	23.98224792	-1.645575569	0.012864186	0.042199227
ENSG00000281332	LINC00997	61.70247223	33.91689771	0.863542851	0.013026824	0.042679019
ENSG00000139133	ALG10	55.67694559	29.89131159	0.897809459	0.013132308	0.042997587
ENSG00000285653	AC006238.2	13.33614773	2.761846154	2.261543149	0.013146252	0.043036483
ENSG00000185818	NAT8L	0.670051556	8.564866807	-3.738745677	0.013168809	0.043096793
ENSG00000279491	AP003733.4	19.82190276	6.283059471	1.656423745	0.013188648	0.043149445
ENSG00000249138	SLED1	4.785762975	0	4.623747458	0.013189037	0.043149445
ENSG00000147231	CXorf57	8.513951696	28.05044708	-1.709245503	0.01319698	0.04316866
ENSG00000234693	AL353653.1	8.604231866	0.723468803	3.60480074	0.013306949	0.043487442
ENSG00000285881	AC105206.3	12.68278797	2.148126224	2.57817444	0.01331957	0.043521868
ENSG00000188488	SERPINA5	4.711452343	0	4.603925268	0.013360972	0.043643469
ENSG00000276805	AL133216.2	44.24549117	20.90487979	1.078735498	0.01338659	0.043699764
ENSG00000145536	ADAMTS16	0.937587874	8.367649246	-3.161543944	0.013445767	0.043865473
ENSG00000107611	CUBN	54.37157628	93.07629039	-0.773952285	0.01352473	0.044093212
ENSG00000155980	KIF5A	48.46047072	24.58866262	0.978857053	0.013533607	0.044103736
ENSG00000237945	LINC00649	26.1645075	9.245427294	1.50843485	0.01356321	0.044186394
ENSG00000127472	PLA2G5	0.304691634	5.964233386	-4.151944643	0.013597779	0.044278259
ENSG00000162669	HFM1	9.219808372	1.030328768	3.138823736	0.013604708	0.044293905
ENSG00000198885	ITPRIPL1	54.69088725	28.80981424	0.924989185	0.013629527	0.04436778
ENSG00000078018	MAP2	8.974085766	28.89882851	-1.685112103	0.01380285	0.044847969
ENSG00000128203	ASPHD2	69.68996605	40.76551736	0.773936059	0.013844625	0.044969688
ENSG00000179766	ATP8B5P	0	4.628696707	-4.745360118	0.013862928	0.045022127
ENSG00000183668	PSG9	22.7028781	46.61834982	-1.036980569	0.013872299	0.045040091
ENSG00000136237	RAPGEF5	6.674581068	21.20843564	-1.680435209	0.013885122	0.045073143
ENSG00000150054	MPP7	8.374573125	24.23393817	-1.529729832	0.013887384	0.045073467
ENSG00000226153	Z93242.1	4.829739463	0	4.63557683	0.013890722	0.045075496
ENSG00000157601	MX1	176.7041426	345.3144125	-0.966796271	0.013950329	0.045242549
ENSG00000234840	LINC01239	0.297870462	5.977851559	-4.154768389	0.014047753	0.045523105
ENSG00000233452	STXBP5-AS1	26.81104609	50.46374712	-0.912650425	0.014055178	0.045533011
ENSG00000114757	PEX5L	9.165961257	1.030328768	3.131726565	0.014124185	0.045735247
ENSG00000243547	HNRNPKP4	92.35173257	52.17228731	0.822798699	0.014236599	0.046070632
ENSG00000251537	AC005324.3	4.850830914	0	4.652172291	0.014273439	0.046182682
ENSG00000226312	CFLAR-AS1	43.82125655	20.7057149	1.079117665	0.014380214	0.046492086
ENSG00000264107	AC138207.2	8.37322293	0.713154737	3.563292416	0.014497437	0.04684202
ENSG00000245275	SAP30L-AS1	13.69468648	31.90410465	-1.219448387	0.01450019	0.046843657
ENSG00000135917	SLC19A3	4.637141711	0	4.583898568	0.014527614	0.046907498
ENSG00000227811	INKA2-AS1	4.637141711	0	4.583898568	0.014527614	0.046907498

ENSG00000278989	AP001148.1	2.314846302	13.21408518	-2.551763846	0.014614882	0.047163023
ENSG00000180767	CHST13	0.304691634	5.921325069	-4.143138177	0.01473885	0.047518947
ENSG00000227199	ST7-AS1	30.58868227	12.53352469	1.283048424	0.014744618	0.047530191
ENSG00000173662	TAS1R1	11.38361189	2.094903841	2.441782064	0.014801244	0.047683241
ENSG00000260781	ARHGAP23P1	5.58837193	18.7651203	-1.742733184	0.014856125	0.047845259
ENSG00000177144	NUDT4B	21.04309082	6.651803831	1.658937715	0.014889514	0.047937557
ENSG00000280099	AL603750.1	13.73866296	31.9676411	-1.218712235	0.014957361	0.048111843
ENSG00000232004	CAP1P2	20.288858	41.31570084	-1.027052619	0.014969085	0.048134706
ENSG00000255423	EBLN2	47.11964534	24.54079814	0.940722872	0.015060516	0.048413779
ENSG00000250979	AC022905.1	9.453866764	1.064575073	3.164869461	0.01510647	0.04851662
ENSG00000144395	CCDC150	37.47013137	16.56252138	1.179896547	0.015113333	0.048531186
ENSG00000258537	FRMD6-AS2	0	5.158653799	-4.907285555	0.015147413	0.048625646
ENSG00000255158	AC131934.1	7.903218232	0.701188618	3.491468589	0.015173589	0.048694678
ENSG00000062524	LTK	7.913811121	25.62640309	-1.701599701	0.015186217	0.048727705
ENSG00000153902	LGI4	2.805942451	12.90887727	-2.20329369	0.01520475	0.048767797
ENSG00000256713	PGA5	6.244781142	0.361734402	4.049661301	0.015205729	0.048767797
ENSG00000179083	FAM133A	0.304691634	6.216218914	-4.214750294	0.015244846	0.048855678
ENSG00000160200	CBS	62.7369769	111.1636912	-0.829474889	0.015290429	0.048985687
ENSG00000198133	TMEM229B	4.25508999	16.39789807	-1.932151602	0.01549433	0.049601838
ENSG00000093100	AC016026.1	6.92165387	20.70736695	-1.586020425	0.015531281	0.04969724
ENSG00000236397	DDX11L2	75.33837833	43.03495683	0.808589958	0.015544245	0.04973109
